# Identification of *WRKY* Family Members and Characterization of the Low-Temperature-Stress-Responsive *WRKY* Genes in Luffa (*Luffa cylindrica* L.)

**DOI:** 10.3390/plants13050676

**Published:** 2024-02-28

**Authors:** Jianting Liu, Lijuan Peng, Chengjuan Cao, Changhui Bai, Yuqian Wang, Zuliang Li, Haisheng Zhu, Qingfang Wen, Shuilin He

**Affiliations:** 1 College of Agriculture, Fujian Agriculture and Forestry University, Fuzhou 350002, China; ljt338625@163.com; 2 Crops Research Institute, Fujian Academy of Agricultural Sciences, Fuzhou 350013, China; ljt18396513693@126.com (C.B.); lzuliang@163.com (Z.L.); 3 Fujian Key Laboratory of Vegetable Genetics and Breeding, Fuzhou 350013, China; 4 College of Horticulture, Fujian Agriculture and Forestry University, Fuzhou 350002, China; plj_678@163.com (L.P.); 17689628171@163.com (C.C.); 15136739206@163.com (Y.W.)

**Keywords:** *Luffa cylindrica*, WRKY transcription factors, abiotic stress, expression analysis

## Abstract

The plant-specific WRKY transcription factor family members have diverse regulatory effects on the genes associated with many plant processes. Although the WRKY proteins in *Arabidopsis thaliana* and other species have been thoroughly investigated, there has been relatively little research on the WRKY family in *Luffa cylindrica*, which is one of the most widely grown vegetables in China. In this study, we performed a genome-wide analysis to identify *L. cylindrica WRKY* genes, which were subsequently classified and examined in terms of their gene structures, chromosomal locations, promoter cis-acting elements, and responses to abiotic stress. A total of 62 *LcWRKY* genes (471–2238 bp) were identified and divided into three phylogenetic groups (I, II, and III), with group II further divided into five subgroups (IIa, IIb, IIc, IId, and IIe) in accordance with the classification in other plants. The *LcWRKY* genes were unevenly distributed across 13 chromosomes. The gene structure analysis indicated that the *LcWRKY* genes contained 0–11 introns (average of 4.4). Moreover, 20 motifs were detected in the LcWRKY proteins with conserved motifs among the different phylogenetic groups. Two subgroup IIc members (LcWRKY16 and LcWRKY31) contained the WRKY sequence variant WRKYGKK. Additionally, nine cis-acting elements related to diverse responses to environmental stimuli were identified in the *LcWRKY* promoters. The subcellular localization analysis indicated that three LcWRKY proteins (LcWRKY43, LcWRKY7, and LcWRKY23) are localized in the nucleus. The tissue-specific *LcWRKY* expression profiles reflected the diversity in *LcWRKY* expression. The RNA-seq data revealed the effects of low-temperature stress on *LcWRKY* expression. The cold-induced changes in expression were verified via a qRT-PCR analysis of 24 differentially expressed *WRKY* genes. Both *LcWRKY7* and *LcWRKY12* were highly responsive to the low-temperature treatment (approximately 110-fold increase in expression). Furthermore, the *LcWRKY8*, *LcWRKY12*, and *LcWRKY59* expression levels increased by more than 25-fold under cold conditions. Our findings will help clarify the evolution of the luffa *WRKY* family while also providing valuable insights for future studies on WRKY functions.

## 1. Introduction

Luffa, which is widely cultivated all over the world, especially in China, is a diploid species (2*n* = 26). The main cultivated luffa species are *Luffa acutangula* Roxb. (ridged) and *Luffa cylindrica* Roem. (smooth). Suitable temperatures for *L. cylindrica* seedling growth are 22–28 °C during the day and 17–18 °C at night. The temperatures during the flowering and fruit-bearing period are relatively high, typically 25–29 °C during the day and 18–22 °C at night. In contrast, temperatures below 10–12 °C have inhibitory effects on *L. cylindrica* seedling growth. According to field observations, the low temperatures and low light intensity that are common in spring and autumn affect *L. cylindrica* seedling growth, photosynthetic activities, and dry matter accumulation, resulting in insufficient nutrient levels, curled leaves, deformed fruits, and other undesirable traits, ultimately leading to decreased yield and quality, which has seriously restricted the development of the *L. cylindrica* industry.

In plants, low-temperature stress can activate stress signals, which are then transmitted to the nucleus through signaling pathways involving Ca^2+^ messengers, a reactive oxygen species (ROS) burst, abscisic acid (ABA) and other hormones, and various kinases. The integration of multiple transcription factors (TFs) results in a regulatory network important for the reprogramming of gene expression required for the plant response to low-temperature stress. The WRKY TFs were initially identified in sweet potato. On the basis of whole-genome sequencing and functional genomics studies involving various plants, the WRKY family has been identified as one of the most important TF families in plants [1,2,3,4]. The typical feature of WRKY proteins is one or two highly conserved WRKY domains comprising approximately 60 amino acids and the N-terminal consisting of highly conserved WRKYGQK domains (with a few variant sequences, including WRKYGKK, WRKYGMK, and WRKYGEK). The C-terminal contains a motif encoding a zinc-finger structure (CX4-7CX22-23HXH/C) [5,6]. The WRKY TFs are broadly involved in the regulation of plant growth and development as well as responses to biotic and abiotic stresses because they bind to the conserved W-box element [(C/T)TGACC(A/T)] in target gene promoters and activate or inhibit gene expression [7,8,9]. Generally, WRKY TFs mainly control the transcription of the downstream target genes in the nucleus. However, recent studies showed that in pepper (Solanaceae), CaWRKY27b (with a WRKYGMK variant sequence) in the cytoplasm enters the nucleus after being phosphorylated by CaCDPK29 and then combines with CaWRKY40 to activate the transcription of target genes [6,10].

Earlier research indicated that WRKY proteins are important for decreasing stress-induced damages and increasing stress tolerance via signal transduction pathways and the production of a series of proteins mediating plant defense responses. The WRKY responses to abiotic stress may depend on ABA [11,12] or they may be mediated through signal transduction pathways associated with different phytohormones, including salicylic acid (SA) [13,14] and methyl jasmonate (MeJA) [15]. The WRKY proteins can bind directly to the W-box element in the promoter of their own gene or in the promoter of downstream target genes to positively or negatively regulate gene expression, thereby increasing or decreasing plant tolerance to environmental stresses [16,17]. According to studies on rice, after low-temperature stress signals are transmitted to the cell interior, the Ca^2+^ channel (i.e., CNGC) alters the concentration of Ca^2+^, which is the second messenger in cells, after which MAPK, MAPKK kinase cascades, and CDPK as well as other protein kinases affect the binding between WRKY TFs and target genes, resulting in abiotic stress responses that modulate the low-temperature tolerance of plants [18]. Notably, defense responses to abiotic stresses, such as low temperatures, can protect plants via the synthesis of osmoregulatory substances (e.g., soluble sugars and proline) and certain proteins with protective effects against cold stress [19]. In *Cynodon dactylon* (L.), CdWRKY2 positively regulates the cold stress response by targeting the *CdSPS1* and *CdCBF1* promoters and activating expression to coordinately mediate sucrose biosynthesis and the CBF signaling pathway, ultimately leading to cold tolerance [20].

There has been considerable research conducted to clone and functionally characterize *WRKY* genes in model plants and some agriculturally important crops. For example, the functions of WRKY TFs related to plant responses to abiotic stressors, such as low-temperature conditions, have been reported for *Arabidopsis thaliana* [21], rice [22], wheat [23], soybean [24], tomato [25], cucumber [26], autumn eggplant [27], bamboo [28], banana [29], and centipede grass [30]. Some WRKY TFs are positive regulators [20,25], whereas others are negative regulators [11]. A WRKY TF can regulate the expression of hundreds of target genes [31,32,33] as well as its own gene [34]. Moreover, WRKY TFs can be activated by other TFs as part of a cascade or regulatory network that controls plant defense responses to low-temperature stress. In rice, the OsWRKY63–OsWRKY76–OsDREB1B module helps regulate cold tolerance via the optimization of the response to low-temperature stress [22].

The sequenced genomes of many Cucurbitaceae crops, such as *Cucumis melo* [35], *Cucumis sativus* [36], *Cucurbita maxima* [37], *Cucurbita moschata* [37], *Cucurbita pepo* [38], *Benincasa hispida* [39], *Citrullus lanatus* [40], *L. acutangula* Roxb. [41], and *L. cylindrica* Roem. [42], are useful resources for genome-wide analyses of Cucurbitaceae species. In this study, we performed a genome-wide analysis of the *LcWRKY* genes in *L. cylindrica*, resulting in the identification of 62 *LcWRKY* genes. A comprehensive analysis involving bioinformatics techniques and methods for examining gene expression patterns was performed. On the basis of the tissue-specific *LcWRKY* expression profiles as well as the expression patterns in response to a low-temperature treatment, several *LcWRKY* genes were identified as candidate regulators involved in the abiotic stress response of *L. cylindrica*.

## 2. Results

### 2.1. Identification of LcWRKY Genes in L. cylindrica

The HMM software version 3.0 package (i.e., hidden Markov model) was used to identify the putative WRKY proteins in *L. cylindrica*. The candidates were then compared with proteins in the NCBI and Pfam databases to confirm the presence of the WRKY domains. The 62 *LcWRKY* genes with complete WRKY domains identified in the *L. cylindrica* genome were named *LcWRKY1*–*62* according to their order on the 13 *L. cylindrica* chromosomes (Supplementary File S1). The length of the coding sequences ranged from 471 bp to 2238 bp. The analysis of the conserved domains indicated that 11 LcWRKY proteins (LcWRKY10, LcWRKY13, LcWRKY27, LcWRKY28, LcWRKY35, LcWRKY36, LcWRKY41, LcWRKY43, LcWRKY55, LcWRKY56, and LcWRKY60) contained two conserved WRKY domains, whereas the other 51 LcWRKY proteins had only one conserved WRKY domain (Supplementary File S2). The molecular weights ranged from 18.35 kDa (LcWRKY40) to 82.10 kDa (LcWRKY10), with an average of 40.12 kDa. Only 12 LcWRKY proteins had molecular weights exceeding 50 kDa (LcWRKY27, LcWRKY20, LcWRKY41, LcWRKY51, LcWRKY37, LcWRKY13, LcWRKY11, LcWRKY55, LcWRKY18, LcWRKY24, LcWRKY56, and LcWRKY10). The isoelectric point (pI) ranged from 4.70 (LcWRKY5) to 9.87 (LcWRKY12), with an average of 7.41. Of the identified LcWRKY proteins, 43.55% (27/62) had a pI greater than 7.0.

### 2.2. Chromosomal Locations and Duplication of LcWRKY Genes

**Figure 1:**
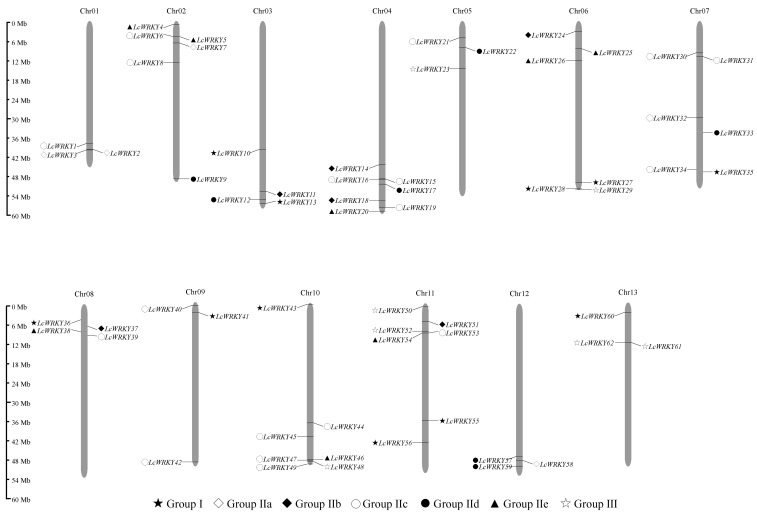
Chromosomal distribution of the *LcWRKY* genes. The chromosomal position of each *LcWRKY* gene can be determined using the scale on the left.

The 62 *LcWRKY* genes were mapped to the 13 *L. cylindrica* chromosomes (Figure 1). Chromosomes 4, 10, and 11 contained the most *LcWRKY* genes (seven each), whereas only three *LcWRKY* genes were located on chromosomes 1, 5, 9, 12, and 13. The other chromosomes contained 4–6 *LcWRKY* genes. The 62 identified LcWRKY proteins were classified into three groups according to the number of WRKY domains and the zinc-finger motif structure [43]. Group I consisted of 11 LcWRKY proteins. Group II, which was the largest group and comprised 44 LcWRKY proteins, was further divided into five subgroups, with subgroups IIa, IIb, IIc, IId, and IIe consisting of 4, 6, 19, 7, and 8 LcWRKY proteins, respectively (Supplementary File S1). Group III included seven LcWRKY proteins. Most of the LcWRKY proteins contained the conserved WRKYGQK motif, but LcWRKY16 and LcWRKY31 had slight variations in their signature motif (i.e., WRKYGKK) (Supplementary File S2). Similar results were reported for *Liriodendron chinense* [44].

### 2.3. Analysis of LcWRKY Cis-Acting Elements and Gene Structures

**Figure 2:**
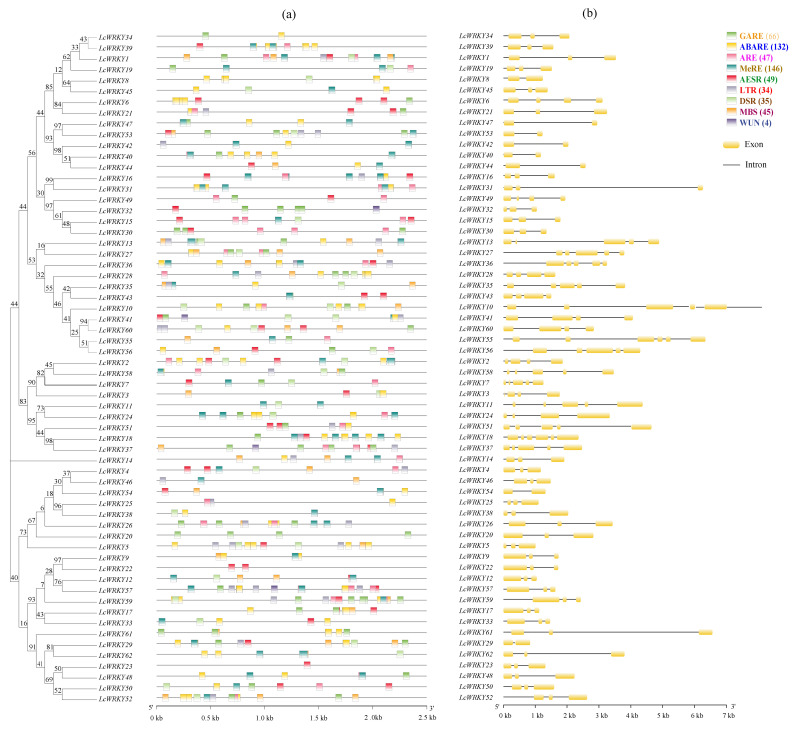
*Luffa cylindrica LcWRKY* promoter elements and structures according to phylogenetic relationships. The phylogenetic tree was constructed on the basis of the full-length *L. cylindrica* WRKY protein sequences using MEGA (version 7.0). The proportional lengths of the *WRKY* genes are presented. The groups are differentiated by color. (**a**) The promoter elements were analyzed using the TBtools software (version 1.6). (**b**) The *LcWRKY* gene structures were examined using the Gene Structure Display Server 2.0 software.

The *LcWRKY* promoters (2.0 kb upstream region) were analyzed using the PlantCARE database (Supplementary File S3). A total of 6324 (61.78%) known cis-acting elements were detected in the *LcWRKY* genes. These cis-acting elements were associated with abiotic and biotic stress responses as well as physiological and developmental processes. More specifically, 146 MeJA-responsive elements and 132 ABA-responsive elements were detected in 41 (66.13%) and 45 (72.58%) of the *LcWRKY* promoter regions, respectively (Figure 2a); these were the most abundant cis-acting elements among the nine analyzed elements. Additionally, 66 gibberellin-responsive elements were detected in 59.68% of the *LcWRKY* promoter regions, 49 SA-responsive elements were detected in 54.84% of the *LcWRKY* promoter regions, 47 auxin-responsive elements were detected in 51.62% of the *LcWRKY* promoter regions, 45 MYB-binding sites associated with responses to drought were detected in 50% of the *LcWRKY* promoter regions, 35 defense and stress-responsive elements were detected in 48.39% of the *LcWRKY* promoter regions, and 34 low-temperature-responsive elements were detected in 40.32% of the *LcWRKY* promoter regions. Four wound-responsive elements were detected in only four *LcWRKY* promoter regions. The presence of such versatile cis-acting elements reflected the functional diversity of the LcWRKY TFs in *L. cylindrica* (Supplementary File S4).

The structural features of the identified *LcWRKY* genes were examined using the GSDS server. The examination of the exon and intron regions (Figure 2b) revealed that 50% of the 62 identified *LcWRKY* genes had three exons. Eight *LcWRKY* genes contained two exons, eight *LcWRKY* genes consisted of four exons, eight *LcWRKY* genes included five exons, four *LcWRKY* genes had six exons, and three *LcWRKY* genes contained seven exons. In contrast, *LcWRKY47* had no introns. The identified *LcWRKY* genes also differed in terms of size, ranging from 879 bp (*LcWRKY29* in group III) to 8348 bp (*LcWRKY10* in group I) [45] (Supplementary Files S1 and S5). The *LcWRKY* gene sizes in group I ranged from 1578 bp (*LcWRKY43*) to 8348 bp (*LcWRKY10*). The *LcWRKY* gene sizes in group II ranged from 1205 bp (*LcWRKY5*) to 6681 bp (*LcWRKY31*). The *LcWRKY* gene sizes in group III ranged from 879 bp (*LcWRKY29*) to 6956 bp (*LcWRKY61*).

### 2.4. Phylogenetic Relationships and Conserved Motifs

**Figure 3:**
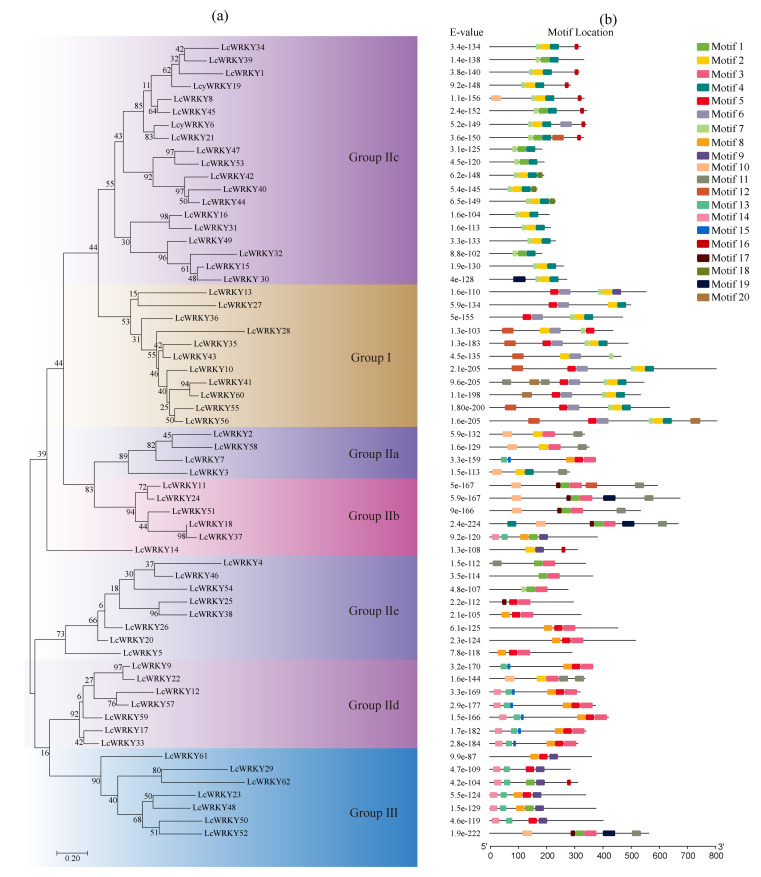
Conserved motifs in *L. cylindrica* WRKY proteins according to phylogenetic relationships. (**a**) Unrooted phylogenetic tree constructed on the basis of full-length WRKY protein sequences using MEGA (version 7.0). (**b**) Distribution of conserved motifs among WRKY proteins. Different motifs are indicated by different colored blocks as indicated at the top of the figure.

On the basis of the phylogenetic analysis, the 62 *LcWRKY* genes were classified into three groups. Group I was composed of 11 *LcWRKY* genes. Group II contained 44 *LcWRKY* genes, which was more than the seven *LcWRKY* genes in group III. Group II was further divided into subgroups IIa, IIb, IIc, IId, and IIe, which consisted of 4, 6, 19, 7, and 8 *LcWRKY* genes, respectively (Figure 3a). The typical feature of the encoded LcWRKY proteins was one or two highly conserved WRKY domains comprising approximately 60 amino acids. The N-terminal of this domain included a highly conserved WRKYGQK sequence or a slightly variant sequence (WRKYGKK; only in LcWRKY16 and LcWRKY31). The C-terminal contained a zinc-finger motif (CX4-7CX22-23HXH/C) (Supplementary File S2). To further investigate the diversity among the motifs in the LcWRKY sequences, the conserved motifs in the 62 LcWRKY proteins were predicted using MEME. Among the twenty predicted motifs, three WRKY motifs (i.e., motifs 1, 2, and 5) were broadly distributed, whereas the other motifs were limited to certain phylogenetic groups (Figure 3b and Supplementary File S6). These three WRKY motifs are involved in DNA binding as well as protein–protein interactions. Relatively little is known about the other motifs.

### 2.5. LcWRKY Expression Profiles in Seven Tissues

**Figure 4:**
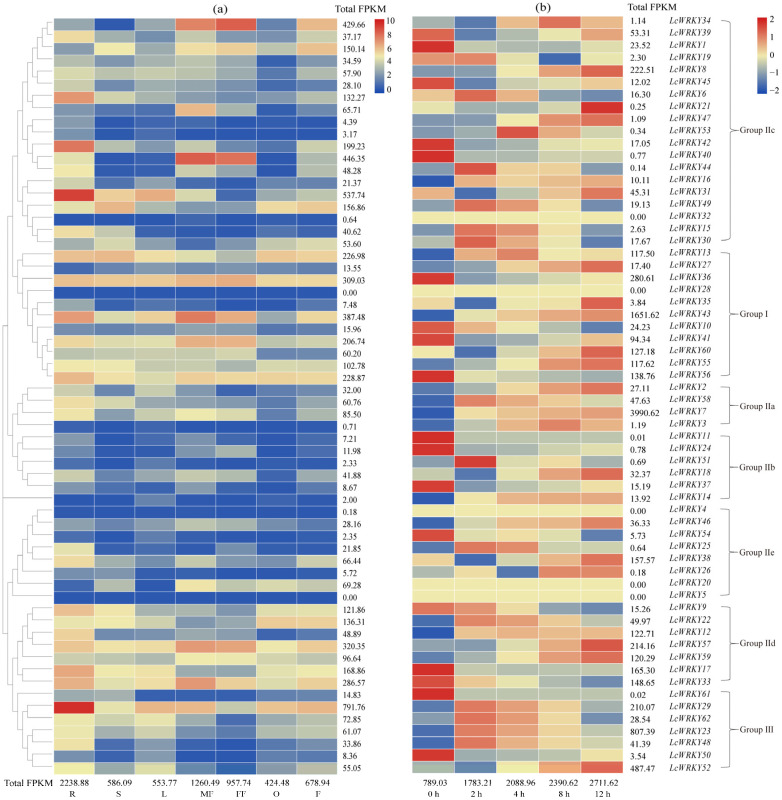
Tissue-specific and low-temperature-stress-induced *LcWRKY* expression profiles. Transcriptome data were used to determine the *LcWRKY* expression profiles. The color scale represents the FPKM normalized log_10_-transformed counts, with blue and red indicating low and high expression levels, respectively. (**a**) Tissue-specific *LcWRKY* expression profiles. (**b**) *LcWRKY* expression levels in the leaves at five time-points during a low-temperature treatment.

The expression levels of all 62 *LcWRKY* genes were thoroughly examined by conducting a rigorous transcriptome analysis of seven *L*. *cylindrica* tissues (root, stem, leaf, male flower, female flower, fruit, and ovary) on the basis of publicly available transcriptome data. The fragments per kilobase of transcript per million (FPKM) values determined on the basis of the transcriptome sequencing data revealed differences in *LcWRKY* expression among the selected tissues. Specifically, *LcWRKY* expression levels were highest in the root, followed by the male flower, female flower, fruit, stem, leaf, and ovary, with FPKM values of 2231.04, 1254.83, 954.89, 669.01, 583.42, 555.61, and 422.34, respectively (Figure 4a). Among the 62 *LcWRKY* genes, *LcWRKY5* and *LcWRKY28* had undetectable expression levels (FPKM value of 0) in the seven examined tissues. The FPKM values for 13 *LcWRKY* genes (*LcWRKY4*, *LcWRKY32*, *LcWRKY3*, *LcWRKY14*, *LcWRKY51*, *LcWRKY54*, *LcWRKY53*, *LcWRKY47*, *LcWRKY26*, *LcWRKY11*, *LcWRKY35*, *LcWRKY50*, and *LcWRKY37*) were less than 10 in the seven examined tissues, whereas the FPKM values for 28 *LcWRKY* genes (e.g., *LcWRKY2*, *LcWRKY7*, and *LcWRKY8*) ranged from 11.98 to 96.64 in the selected tissues, with an average of 45.66. The FPKM values for the other 19 *LcWRKY* genes were also determined for all tissues. Furthermore, seven *LcWRKY* genes were most highly expressed in the root, especially *LcWRKY29* (FPKM value of 554.42) and *LcWRKY31* (FPKM value of 407.36). Five *LcWRKY* genes (*LcWRKY33*, *LcWRKY40*, *LcWRKY41*, *LcWRKY43*, and *LcWRKY57*) were predominantly expressed in the male flower (FPKM values of 92.78, 244.61, 60.78, 148.05, and 88.66, respectively). The *LcWRKY34* (FPKM value of 237.85), *LcWRKY36* (FPKM value of 65.23), and *LcWRKY40* (FPKM value of 175.89) genes were preferentially expressed in the female flower (Supplementary File S7).

### 2.6. Analysis of LcWRKY Expression under Low-Temperature Stress Conditions

The WRKY TFs are well-known regulators of abiotic stress signaling pathways. In this study, an Illumina transcriptome sequencing (RNA-seq) analysis was performed to determine the *LcWRKY* expression patterns at specific time-points during an exposure to low-temperature (5 °C) stress conditions. According to the heat map (Figure 4b), the expression levels of the 20 stress-response-related genes identified in *L. cylindrica* varied among the selected time-points (0, 2, 4, 8, and 12 h). The FPKM values indicated the *LcWRKY* expression levels gradually increased, with FPKM values of 789.03, 1783.21, 2088.96, 2390.62, and 2711.62 at 0, 2, 4, 8, and 12 h, respectively (Figure 4b and Supplementary File S8).

**Figure 5:**
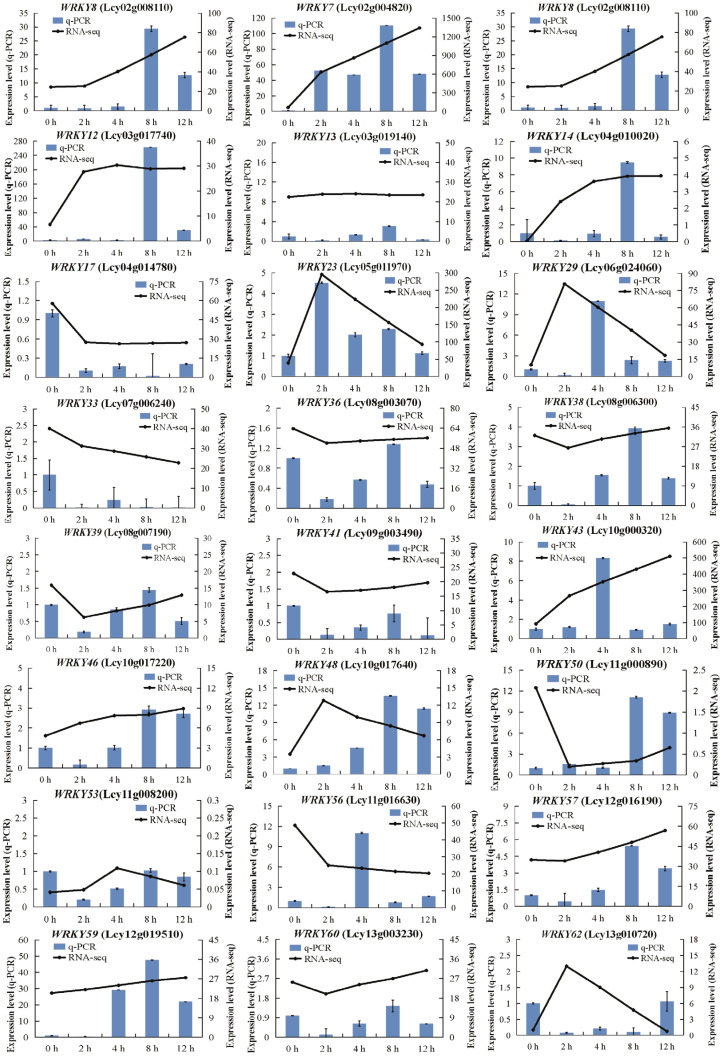
Quantitative real-time polymerase chain reaction analysis of the expression of selected *LcWRKY* genes associated with the *L. cylindrica* leaf response to low-temperature stress. The 18S rRNA gene was used as the internal control. Error bars represent the standard error of three biological replicates.

**Figure 6:**
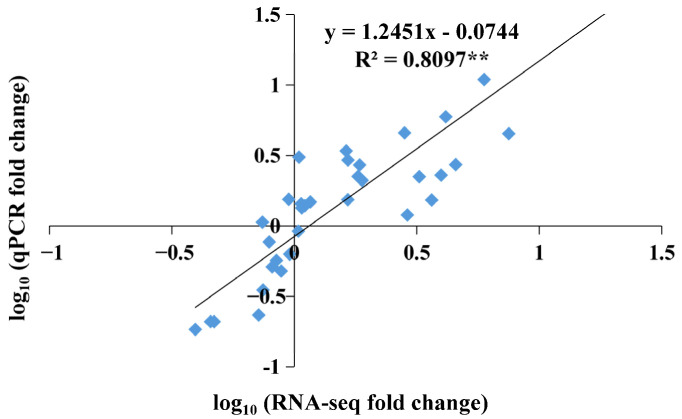
Regression analysis of the fold-change values determined on the basis of transcriptome sequencing (RNA-seq) and qRT-PCR data. For the RNA-seq analysis, the FPKM values at 2, 4, 8, and 12 h were compared with the FPKM value at 0 h to calculate the fold-change. For the qRT-PCR analysis, the expression levels at 2, 4, 8, and 12 h were normalized against the expression level at 0 h to calculate the fold-change. **, significant correlation (*p* < 0.01).

To verify the RNA-seq data, 24 *LcWRKY* genes related to abiotic stress tolerance (6 from group I, 12 from group II, and 6 from group III) were selected for a quantitative real-time polymerase chain reaction (qRT-PCR) analysis (Figure 5). There were significant differences in the expression levels of these 24 *LcWRKY* genes, many of which were highly expressed during the low-temperature treatment. Specifically, the *LcWRKY2*, *LcWRKY7*, *LcWRKY8*, *LcWRKY12*, *LcWRKY14*, *LcWRKY36*, *LcWRKY38*, *LcWRKY46*, *LcWRKY48*, *LcWRKY50*, *LcWRKY57*, and *LcWRKY59* expression levels peaked at 8 h, whereas *LcWRKY29*, *LcWRKY43*, and *LcWRKY56* were most highly expressed at 4 h. Notably, *LcWRKY23* was highly expressed only at 2 h, while *LcWRKY13*, *LcWRKY33*, *LcWRKY39*, *LcWRKY41*, *LcWRKY53*, *LcWRKY60*, and *LcWRKY62* expression levels were almost undetectable throughout the low-temperature treatment period. There was a strong positive correlation (*R*^2^ = 0.8097; *p* ≤ 0.01) between the qRT-PCR and RNA-seq data, even for the *LcWRKY* genes that were generally expressed at low levels (Figure 6).

### 2.7. Subcellular Localization of LcWRKY Proteins

**Figure 7:**
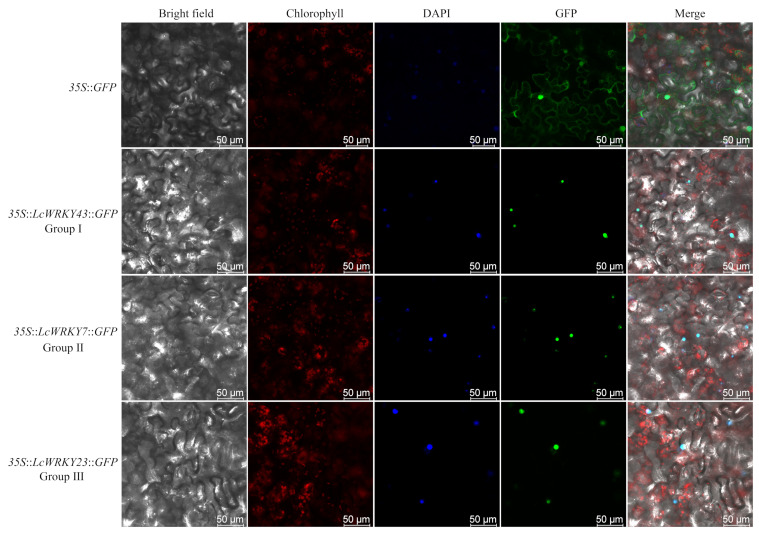
Subcellular localization of three LcWRKY proteins in the lower epidermal cells of *Nicotiana benthamiana.* The green fluorescence, visible light, and merged green fluorescence and visible light images are presented. *35S*::*GFP*: *Agrobacterium tumefaciens* strain carrying the empty vector (pCAMBIA1300-*GFP*); *35S*::*LcWRKY*::*GFP*: *A. tumefaciens* strain carrying a recombinant vector (pCAMBIA1300-*LcWRKY7*-*GFP*, pCAMBIA1300-*LcWRKY23*-*GFP*, or pCAMBIA1300-*LcWRKY43*-*GFP*). Scale bars = 50 µM.

To examine the subcellular localization of the LcWRKY proteins, constructs were generated for the expression of LcWRKY43 (group I), LcWRKY7 (group II), and LcWRKY23 (group III) fused to the green fluorescent protein (GFP). The constructs were inserted into *Nicotiana benthamiana* via an *Agrobacterium-tumefaciens*-mediated transformation for the subsequent transient expression and subcellular localization analysis. In the control cells containing the empty vector (*35S*::*GFP*), green fluorescence was distributed in the cell membrane, cytoplasm, and nucleus, whereas in the cells containing a *35S*::*LcWRKY*::*GFP* construct, green fluorescence was detected exclusively in the nucleus. Accordingly, LcWRKY7, LcWRKY23, and LcWRKY43 were localized to the nucleus, which was consistent with the predicted subcellular localization (Supplementary File S1 and Figure 7). The nuclear localization of these three LcWRKY proteins is in accordance with their putative roles as TFs.

## 3. Discussion

### 3.1. Characterization of the LcWRKY Gene Family in L. cylindrica

Among the plant TF families, the WRKY TF family is one of the largest and most important, with a broad range of functions that affect plant growth, development, signal transduction, and biotic and abiotic stress responses. Thus, in this study, we conducted a whole-genome analysis and detected 62 *LcWRKY* genes (*LcWRKY1*–*62*) in *L. cylindrica.* These genes were divided into three groups (I, II, and III) and five subgroups (IIa, IIb, IIc, IId, and IIe) on the basis of the presence of conserved WRKY domains and a zinc-finger-motif-like structure. The phylogenetic analysis and resulting clades supported the classification of the *LcWRKY* genes into three groups (I, II, and III). There was a close phylogenetic relationship between the *WRKY* genes in subgroups IIa and IIb as well as between the *WRKY* genes in subgroups IId and IIe. Interestingly, in contrast to the other LcWRKY proteins, LcWRKY16 and LcWRKY31 were revealed to contain a non-standard conserved WRKY domain (i.e., Q-to-K substitution in WRKYGQK during evolution, resulting in WRKYGKK). A similar mutation was also detected in the genome of other plants, including *Prunus mume*, *Elaeis guineensis*, and *Fagopyrum tataricum* [5,7,15]. Our phylogenetic analysis revealed the close relationship between the two subgroup IIc *LcWRKY* genes, which belonged to the same branch of the phylogenetic tree (Figure 3a). Variations in the WRKY motif can alter the DNA-binding activity, which may help to explain the functional diversity among the WRKY TFs [6]. Earlier research showed that Cucurbitaceae originated approximately 80 million years ago, with at least four whole-genome duplication (WGD) events occurring during the evolution of cucurbits [46]. These WGD events are the main factors that have contributed to the morphological diversity of cucurbit plants; however, the genome size and the number of genes in these species decreased to the corresponding levels before the WGD events [47]. In the current study, *WRKY* gene duplication events were not detected, which is consistent with the findings of previous studies on *C. sativus* [48] and *L. cylindrica* Roxb. Because of the lack of gene duplication events in the *LcWRKY* family, the group III *LcWRKY* genes, which are most active during evolution, likely have relatively conserved functions.

The conserved WRKY domains of the LcWRKY proteins were analyzed in this study. Multiple sequence alignments revealed a change in the WRKY domains of LcWRKY10 and LcWRKY37 (subgroup IIc). Most characterized WRKY proteins preferentially bind to their cognate cis-acting W-box element via their WRKY domain. Hence, it may be worthwhile to further investigate the binding specificity and functionality of these two LcWRKY proteins. An earlier investigation involving broomcorn millet (*Panicum miliaceum* L.) indicated that five PmWRKY proteins (PmWRKY2, PmWRKY15, PmWRKY23, PmWRKY24, and PmWRKY28) contain the variant sequence WRKYGKK, while four PmWRKY proteins (PmWRKY5, PmWRKY6, PmWRKY8, and PmWRKY20) contain the variant sequence WRKYGEK. Changes in the WRKYGQK motif can modulate the binding of the WRKY TF to target DNA sequences. For example, in pepper, CaWRKY27b, which contains the WRKYGMK domain (i.e., Q-to-M substitution in the conserved WRKYGQK sequence), cannot bind to W-boxes in the nucleus, but it can combine with CaCDPK29 to form a complex that regulates the CaWRKY40-mediated defense response to biotic stress [6].

### 3.2. Analysis of L. cylindrica WRKY Gene Promoters

Various types of cis-acting elements were identified in the *LcWRKY* promoter regions, suggesting the encoded TFs may be involved in diverse biological processes influencing plant growth and development [2,45,49]. An earlier study demonstrated that the expression of the cucumber gene *CsWRKY46* can increase the cold tolerance of transgenic plants, which may be related to the associated positive regulation of the cold signaling pathway in an ABA-dependent manner [26]. Salicylic acid is a key regulator of plant responses to various pathogens because of its effects on multiple mechanisms that induce defense activities. In *A. thaliana*, the expression of *MiWRKY53* affects the regulation of plant defense responses involving SA-mediated mechanisms [13]. In oil palm, *EgWRKY59* and *EgWRKY65* contribute to similar regulatory mechanisms involving ABA-, SA-, and ROS-mediated signaling pathways during an exposure to drought or other abiotic stresses; these two genes may be useful for enhancing the abiotic stress tolerance of plants. A recent study showed that *OsWRKY24* and *OsWRKY70* expression levels are upregulated by low temperatures, SA, and MeJA but are downregulated by ABA [50]. In *Dendrobium officinale* seedlings, the expression levels of nine *DoWRKY* genes are significantly affected by cold and MeJA treatments, suggestive of their contributions to stress tolerance [51]. In banana fruit, *MaWRKY26* expression is reportedly induced by cold stress or MeJA, thereby enhancing cold tolerance [29]. In the present study, various cis-acting elements responsive to phytohormones (i.e., MeJA, ABA, and SA) were identified, implying that the LcWRKY TFs likely help control various hormone signaling pathways related to the abiotic and biotic stress responses of *L. cylindrica* (Figure 2a and Supplementary File S4) [12,13,14].

### 3.3. Expression Profiles of LcWRKY Genes in L. cylindrica and the Changes Induced by Low-Temperature Stress

RNA-seq analyses are usually performed to study gene functions and structures and to reveal the molecular mechanisms underlying specific biological processes. In the present study, we used transcriptome data for seven *L. cylindrica* tissues (root, stem, leaf, male flower, female flower, fruit, and ovary) to explore tissue-specific *LcWRKY* expression patterns (Figure 4a). Many of the selected *LcWRKY* genes were highly expressed in the root (51%), whereas a few *LcWRKY* genes were highly expressed in the male flower (12.90%), leaf (3.23%), fruit (4.84%), female flower (1.61%), ovary (1.61%), and stem (1.61%). These results are consistent with the findings of earlier studies involving other plants, including *Acer truncatum* [52], *Pennisetum glaucum* [2], and *Melastoma dodecandrum* [45]. Moreover, the gene expression data indicated that the *LcWRKY* genes have tissue-specific expression profiles, with potentially important roles in root responses to external stresses. The *LcWRKY34*, *LcWRKY40*, *LcWRKY41*, *LcWRKY43*, and *LcWRKY57* expression levels peaked in the flower, suggestive of a key role in the mechanism mediating *L. cylindrica* flower formation and development. In addition, a few *LcWRKY* genes were expressed at low or undetectable levels (e.g., *LcWRKY3*, *LcWRKY4*, *LcWRKY5*, *LcWRKY14*, *LcWRKY28*, *LcWRKY32*, and *LcWRKY51*). These results suggest that LcWRKY TF genes are expressed at various levels in different organs or tissues to regulate diverse biological and physiological processes in *L. cylindrica*.

Low-temperature stress can substantially alter plant growth and development. Previous studies on the mechanism by which WRKY regulates cold stress responses mainly focused on model plants [11,53]. In the current study, we explored the regulatory functions of LcWRKY TFs in *L. cylindrica* Fusi-1 seedlings that underwent a low-temperature treatment (Supplementary File S8). The analysis of the *LcWRKY* expression levels (i.e., FPKM values) under normal conditions (Figure 4a) showed the *LcWRKY* genes (except *LcWRKY61*) were expressed at lower levels in the leaves (553.77) than in the other examined tissues, with the exception of the ovary (424.48). However, the *LcWRKY* expression levels increased 3.44-fold from 0 h (789.03) to 12 h (2711.62) during the low-temperature treatment, indicative of the *LcWRKY* gene responses to cold conditions. Furthermore, 24 *LcWRKY* genes representing all three groups (I, II, and III) were selected for the qRT-PCR analysis. The expression patterns of the examined *LcWRKY* genes, including *LcWRKY7*, *LcWRKY8*, *LcWRKY12*, and *LcWRKY59*, suggested that the encoded TFs are likely involved in the *L. cylindrica* response to cold stress. Recent research showed that the overexpression of *CsWRKY46* and other cucumber *WRKY* genes can increase the viability of transgenic *A. thaliana* seedlings incubated at 4 °C [26]. Another study involving an analysis of transient gene expression showed that CsWRKY46 (group II) is a nuclear protein [24]. In rice, the overexpression of *OsWRKY76* reportedly enhances the cold stress tolerance at 4 °C, whereas OsWRKY63 negatively regulates chilling tolerance through the OsWRKY63–OsWRKY76–OsDREB1B transcription-regulating module [22]. In the present study, the expression levels of a few *LcWRKY* genes decreased during the exposure to cold stress (e.g., *LcWRKY13*, *LcWRKY33*, *LcWRKY39*, *LcWRKY41 LcWRKY53*, and *LcWRKY60*). We speculate that these genes may play a role in *L. cylindrica’s* responses to other biotic and/or abiotic stresses. Interestingly, a W-box element (WRKY TF binding site) was detected in the region upstream of certain *LcWRKY* genes, including *LcWRKY7*, *LcWRKY8*, *LcWRKY12*, *LcWRKY23*, and *LcWRKY43*, suggestive of the auto-regulation of the expression of these genes under stress conditions (Supplementary File S4).

## 4. Materials and Methods

### 4.1. Plant Materials, Growth Conditions, and Treatment

*Luffa cylindrica* Fusi-1 seeds were washed three times with distilled water, soaked in 2% sodium hypochlorite for 15 min, washed three more times with distilled water, and then sown in plastic boxes. The seeds were placed in an incubator equipped with an LED cold light source for an incubation under the following conditions: 16 h day (28 °C)/8 h night (20 °C); light intensity of 300 μmol photons m^−2^ s^−1^; and relative humidity of 70%. Twenty-day-old seedlings were incubated at 5 °C for 0, 2, 4, 8, and 12 h under a light intensity of 80 μmol photons m^−2^ s^−1^. The stress treatment was initiated at the beginning of a photoperiod. Images of the chlorophyll fluorescence of the leaves were captured using the IMAG-MAX chlorophyll imaging system (blue light source) (Walz, Effeltrich, Germany) to determine the maximum quantum efficiency of photosystem II (*Fv*/*Fm*) (Supplementary File S9). All control and treated samples were examined using three biological replicates. The leaf samples were combined, immediately frozen in liquid nitrogen, and stored at −80 °C.

### 4.2. Total RNA Extraction, RNA Sequencing, and Gene Expression Analysis

The total RNA extraction, mRNA purification, and cDNA library construction steps were completed by Guangzhou Gene Denovo Technologies Co. (Guangzhou, China). The cDNA libraries were constructed as previously described [54] and then sequenced on an Illumina HiSeq 2500 instrument. High-quality reads were aligned to the *L. cylindrica* cultivar P93075 (ASM1713956v1) reference genome using Bowtie2.

For the qRT-PCR analysis, total RNA was extracted from each sample using an EZNA Plant RNA kit (Bio-Tek, Beijing, China). These experiments were completed using three biological replicates and three technical replicates. The RNA samples were quantified and checked for quality using a NanoDrop ND-1000 spectrophotometer (Thermo Scientific, Carlsbad, CA, USA) and a 2100 Bioanalyzer (Agilent Technologies, Santa Clara, CA, USA).

The read count represents the number of reads mapped to the reference genome. However, the FPKM value may be used to represent gene expression levels because it is calculated after considering the sequencing depth and feature length. Notably, the choice of computational methods for analyzing RNA-seq data can influence the estimated gene expression levels in the transcriptome [18]. To assess the differences between the read counts and FPKM values, we calculated the FPKM values for the identified genes in each transcriptome.

The tissue-specific *LcWRKY* expression profiles were investigated using our publicly available Fusi-1 (PRJNA1044273) transcriptomes for the following tissues: root (SAMN38393632, SAMN38393633, and SAMN38393634), stem (SAMN38393635, SAMN38393636, and SAMN38393637), leaf (SAMN38393623, SAMN38393624, and SAMN38393625), male flower (SAMN38393626, SAMN38393627, and SAMN38393628), female flower (SAMN38393620, SAMN38393621, and SAMN38393622), fruit (SAMN38393617, SAMN38393618, and SAMN38393619), and ovary (SAMN38393629, SAMN38393630, and SAMN38393631). The effects of low-temperature stress on *LcWRKY* expression in the seedling leaves were examined at the following five time-points: 0 h (SAMN38393638, SAMN38393639, and SAMN38393640), 2 h (SAMN38393641, SAMN38393642, and SAMN38393643), 4 h (SAMN38393644, SAMN38393645, and SAMN38393646), 8 h (SAMN38393647, SAMN38393648, and SAMN38393649), and 12 h (SAMN38393650, SAMN38393651, and SAMN38393650).

For the qRT-PCR assays, primers (i.e., *LcWRKY*-Fq and *LcWRKY*-Rq) were designed according to the sequences determined in this study (Supplementary File S10). The thermal cycling program was as follows: 93 °C for 3 min; and 40 cycles of 93 °C for 5 s and 72 °C for 30 s. For the control, the 18S rRNA gene (GenBank accession: KM656452) was selected as the reference gene, and the *18S rRNA*-Fq and *18S rRNA*-Rq primers were designed (Supplementary File S10). Relative gene expression levels were calculated using the 2^−ΔΔCt^ method.

### 4.3. Identification of LcWRKY Genes in the L. cylindrica Genome

The *L. cylindrica* WRKY proteins were identified using two methods. Specifically, we searched the *L. cylindrica* genome database (https://www.ncbi.nlm.nih.gov/datasets/genome/GCA_017139565.1/ (accessed on 22 September 2023)) for WRKY proteins on the basis of the sequences of conserved WRKY domains in *A. thaliana*. All *L. cylindrica* peptide sequences were downloaded from the *L. cylindrica* database. The HMM software package and BLASTP were used to obtain candidate WRKY TFs, which were subsequently validated using the Pfam (http://pfam.xfam.org/ (accessed on 22 September 2023)) and SMART (http://smart.embl-heidelberg.de/ (accessed on 22 September 2023)) databases.

### 4.4. Bioinformatics Analysis of the LcWRKY Gene Family

The *LcWRKY* gene structures were visualized using GSDS, which aligned the cDNA sequences to the gene sequences [55]. Phylogenetic trees were constructed according to the maximum likelihood method (bootstrap: 1000 replicates) using MEGA (version 7.0). The *WRKY* genes responsive to stress were identified. In addition, heat maps, phylogenetic trees, and cis-acting elements were visualized using the TBtools software (version 1.6). The theoretical molecular weight and pI were calculated using ProtParam (http://web.expasy.org/protparam/ (accessed on 23 September 2023)). The subcellular localization was predicted using WoLF PSORT (https://wolfpsort.hgc.jp/ (accessed on 23 September 2023)), with plants selected as the biological type. The conserved motifs in the LcWRKY proteins were analyzed using the following optimized parameters of MEME (http://meme-suite.org/tools/meme (accessed on 27 September 2023)): any number of repetitions; maximum number of motifs, 25; minimum sites, 2; and optimum width of each motif, 6–100 residues [56]. The MAST program (http://meme-suite.org/tools/mast (accessed on 27 September 2023)) was used to screen protein databases for the detected motifs. The cis-acting elements in the *LcWRKY* promoters were analyzed using the TBtools software and PlantCARE (http://bioinformatics.psb.ugent.be/webtools/plantcare/html/ (accessed on 30 September 2023)). The PlantCARE results were used to visualize the predicted cis-acting elements.

### 4.5. Subcellular Localization of LcWRKY Proteins

To investigate the nuclear localization of the LcWRKY proteins, the full-length sequences of three *LcWRKY* genes (*LcWRKY7*, *LcWRKY23*, and *LcWRKY43*) without the stop codon were amplified by PCR using the primers listed in Supplementary File S10. The PCR products were inserted into separate pCAMBIA1300 vectors for the expression of a GFP fusion protein. The generated recombinant plasmids were inserted into *N. benthamiana* seedling leaves for the subcellular localization analysis. The empty vector (*35S*::*GFP*) was used as the control. The leaves were examined for GFP fluorescence using a TCS SP8 confocal laser scanning microscope (Leica, Wetzlar, Germany), with 488 nm argon excitation and a 505–530 nm band filter.

## 5. Conclusions

A total of 62 *LcWRKY* genes were differentially regulated under low-temperature stress conditions according to the Illumina sequencing data. A comprehensive analysis of phylogenetic relationships, chromosomal locations, gene structures, and conserved motifs was performed. On the basis of the diverse tissue-specific *LcWRKY* expression profiles as well as the expression patterns in response to the low-temperature treatment, several *LcWRKY* genes (e.g., *LcWRKY7*, *LcWRKY8*, *LcWRKY12*, and *LcWRKY59*) were identified as candidate regulators of the cold stress response of *L. cylindrica*.

## Data Availability

The transcriptome sequencing data are available in the NCBI database (project ID PRJNA1044273). Further inquiries can be directed to the corresponding authors.

## Supplementary Materials

The following supporting information can be downloaded at: https://www.mdpi.com/article/10.3390/plants13050676/s1, File S1: Table S1. WRKY family genes identified in *L. cylindrica*. File S2: WRKY protein sequences. File S3: Promoter region 2.0 kb upstream of *LcWRKY* gene sequences. File S4: Identified cis-acting elements in the *LcWRKY* promoter regions. File S5: Full-length sequences of 62 *LcWRKY* genes. File S6: Table S2. Consistent sequences of the predicted WRKY motifs in *L. cylindrica*. File S7: Tissue-specific *LcWRKY* expression profiles. File S8: Low-temperature-stress-induced *LcWRKY* expression profiles. File S9: Figure S1. Effects of low-temperature stress on 2-week-old luffa seedlings. File S10: Table S3. Primers used in this study.
