# Supplementary material for: Identification of WRKY Family Members and Characterization of the Low-Temperature-Stress-Responsive WRKY Genes in Luffa (Luffa cylindrica L.)
Source: Plants (Basel). 2024 Feb 28;13(5):676. doi: 10.3390/plants13050676 (PMC10935285; doi:10.3390/plants13050676)
Supplement: Supplementary file 1 [file plants-13-00676-s001.zip › Supplementary File S1.pdf]

Table S1. *WRKY* family genes identified in *L. cylindrica.*

Gene name	Gene ID	Position	Coding sequence length/bp	Protein length/aa	WRKY location	Relative molecular weight/kDa	Theoretical Isoelectric Point (pI)	Group
LcWRKY1	Lcy01g013860	Chr01:35317600-35321483	882	293	146-205	32.245	6.31	IIc
LcWRKY2	Lcy01g015520	Chr01:37098283-37100632	933	310	145-205	34.05	8.62	IIa
LcWRKY3	Lcy01g015530	Chr01:37109189-37111044	789	262	87-146	29.30	8.61	IIa
LcWRKY4	Lcy02g000520	Chr02:886468-887701	942	313	146-206	33.89	5.46	IIe
LcWRKY5	Lcy02g003300	Chr02:4395721-4396925	810	269	64-124	30.44	4.70	IIe
LcWRKY6	Lcy02g003370	Chr02:4472340-4476225	951	316	144-203	34.68	6.63	IIc
LcWRKY7	Lcy02g004820	Chr02:6294188-6296022	936	311	157-217	33.98	8.63	IIa
LcWRKY8	Lcy02g008110	Chr02:11911386-11912916	930	309	154-213	34.74	6.67	IIc
LcWRKY9	Lcy02g018410	Chr02:45569374-45571776	1,017	338	270-330	37.70	9.59	IId
LcWRKY10	Lcy03g007290	Chr03:37041515-37049862	2,238	745	260-318;484-543	82.10	5.28	I
LcWRKY11	Lcy03g015080	Chr03:49118873-49123457	1,650	549	233-293	59.67	6.41	IIb
LcWRKY12	Lcy03g017740	Chr03:51487311-51488955	891	296	217-277	32.17	9.87	IId
LcWRKY13	Lcy03g019140	Chr03:52697842-52703679	1,536	511	199-257;372-431	55.37	6.32	I
LcWRKY14	Lcy04g010020	Chr04:41393674-41395779	864	287	120-179	31.70	5.62	IIb
LcWRKY15	Lcy04g013590	Chr04:45650053-45652243	726	241	160-219	27.31	9.16	IIc
LcWRKY16	Lcy04g013690	Chr04:45775210-45777257	588	195	102-161	22.26	7.04	IIc
LcWRKY17	Lcy04g014780	Chr04:47142779-47144474	948	315	242-302	34.14	9.68	IId
LcWRKY18	Lcy04g020100	Chr04:51709386-51711938	1,851	616	343-403	66.24	6.56	IIb
LcWRKY19	Lcy04g022670	Chr04:53830845-53832838	798	265	121-180	29.15	5.41	IIc
LcWRKY20	Lcy04g023770	Chr04:54892243-54896234	1,434	477	238-298	52.35	5.12	IIe
LcWRKY21	Lcy05g005160	Chr05:4760592-4764317	921	306	144-203	34.48	6.35	IIc
LcWRKY22	Lcy05g007820	Chr05:7688892-7691373	1,044	347	279-339	38.61	9.56	IId
LcWRKY23	Lcy05g011970	Chr05:13724246-13726258	945	314	109-171	35.32	5.87	III
LcWRKY24	Lcy06g003090	Chr06:2919058-2922682	1,869	622	267-327	67.00	7.17	IIb
LcWRKY25	Lcy06g008190	Chr06:7989644-7991320	825	274	65-125	29.92	5.35	IIe
LcWRKY26	Lcy06g010380	Chr06:11318747-11322452	1,260	419	212-272	45.19	6.01	IIe
LcWRKY27	Lcy06g021780	Chr06:46758360-46762744	1,386	461	194-252;389-448	50.86	9.31	I
LcWRKY28	Lcy06g023690	Chr06:48356425-48359744	1,212	403	167-225;316-356	44.46	6.56	I
LcWRKY29	Lcy06g024060	Chr06:48619644-48620522	798	265	119-181	29.69	5.43	III
LcWRKY30	Lcy07g002360	Chr07:9134090-9136383	759	252	168-227	28.74	9.41	IIc
LcWRKY31	Lcy07g002580	Chr07:10143214-10149894	600	199	123-182	22.29	5.46	IIc
LcWRKY32	Lcy07g005230	Chr07:27913508-27914770	516	171	93-152	19.65	9.62	IIc
LcWRKY33	Lcy07g006240	Chr07:32119638-32121520	867	288	215-275	31.08	9.62	IId
LcWRKY34	Lcy07g013580	Chr07:42704310-42706648	894	297	169-228	33.03	5.90	IIc
LcWRKY35	Lcy07g014420	Chr07:43494758-43498837	1,359	452	174-232;328-387	49.87	8.93	I
LcWRKY36	Lcy08g003070	Chr08:4389112-4392520	1,305	434	109-167;281-340	47.27	5.95	I
LcWRKY37	Lcy08g004870	Chr08:6315882-6318558	1,563	520	281-341	55.29	6.84	IIb
LcWRKY38	Lcy08g006300	Chr08:7998714-8001062	900	299	74-134	31.99	5.32	IIe
LcWRKY39	Lcy08g007190	Chr08:8985724-8987601	927	308	168-227	34.55	8.14	IIc
LcWRKY40	Lcy09g001010	Chr09:974949-976801	471	156	77-136	18.35	5.42	IIc
LcWRKY41	Lcy09g003490	Chr09:3028755-3033619	1,515	504	230-288;390-449	55.00	7.72	I
LcWRKY42	Lcy09g020790	Chr09:46205239-46207860	534	177	97-156	19.69	9.05	IIc

Gene name	Gene ID	Position	Coding sequence length/bp	Protein length/aa	WRKY location	Relative molecular weight/kDa	Theoretical Isoelectric Point (pI)	Group
LcWRKY43	Lcy10g000320	Chr10:403137-405176	1,752	583	233-291;408-467	46.47	7.02	I
LcWRKY44	Lcy10g008570	Chr10:34586277-34589174	651	216	137-196	24.40	9.56	IIc
LcWRKY45	Lcy10g010610	Chr10:38559010-38560834	957	318	162-221	34.91	6.55	IIc
LcWRKY46	Lcy10g017220	Chr10:45285901-45287860	1,014	337	161-221	36.97	5.82	IIe
LcWRKY47	Lcy10g017240	Chr10:45320615-45323824	516	171	92-151	19.82	8.93	IIc
LcWRKY48	Lcy10g017640	Chr10:45669746-45672602	1,044	347	117-179	38.89	4.84	III
LcWRKY49	Lcy10g018700	Chr10:46586602-46588639	651	216	142-201	24.55	8.53	IIc
LcWRKY50	Lcy11g000890	Chr11:858082-859913	1,116	371	128-190	40.74	5.53	III
LcWRKY51	Lcy11g005270	Chr11:5134386-5139438	1,482	493	237-297	55.09	5.68	IIb
LcWRKY52	Lcy11g007870	Chr11:8150570-8155637	1,059	352	130-192	39.40	5.65	III
LcWRKY53	Lcy11g008200	Chr11:8595284-8596734	537	178	99-158	19.80	8.81	IIc
LcWRKY54	Lcy11g008210	Chr11:8608668-8610091	771	256	121-181	28.78	5.65	IIe
LcWRKY55	Lcy11g015160	Chr11:33909996-33920087	1,767	588	228-286;404-463	64.17	6.15	I
LcWRKY56	Lcy11g016630	Chr11:40122538-40127220	2,232	743	323-381;538-597	80.50	5.99	I
LcWRKY57	Lcy12g016190	Chr12:44128923-44131108	1,041	346	268-328	37.28	9.60	IId
LcWRKY58	Lcy12g017550	Chr12:45195090-45198986	978	325	164-224	36.44	5.78	IIa
LcWRKY59	Lcy12g019510	Chr12:46827117-46830417	1,167	388	315-375	41.94	9.54	IId
LcWRKY60	Lcy13g003230	Chr13:2740565-2744212	1,485	494	204-262;385-444	53.61	8.74	I
LcWRKY61	Lcy13g010710	Chr13:11470918-11477873	1,002	333	163-225	36.59	7.22	III
LcWRKY62	Lcy13g010720	Chr13:11493288-11497467	867	288	120-182	32.00	5.78	III
