# Supplementary material for: Identification of WRKY Family Members and Characterization of the Low-Temperature-Stress-Responsive WRKY Genes in Luffa (Luffa cylindrica L.)
Source: Plants (Basel). 2024 Feb 28;13(5):676. doi: 10.3390/plants13050676 (PMC10935285; doi:10.3390/plants13050676)
Supplement: Supplementary file 1 [file plants-13-00676-s001.zip › Supplementary File S10.pdf]

Table S3. Primers used in this study

Name	Sequence (5'-3')	Function
LcWRKY2-Fq	GTCGCCTAGAGCTTACTATAAATG	qRT-PCR
LcWRKY2-Rq	GGTTAGATCGAAGGTTACAACAG	qRT-PCR
LcWRKY7-Fq	CCGGAAACACGTAGAACG	qRT-PCR
LcWRKY7-Rq	CGAGGATGAGATTGGAGGT	qRT-PCR
LcWRKY8-Fq	TTCCCACAGTCGCAATACC	qRT-PCR
LcWRKY8-Rq	ACGATCATATCTTGTAGAAGCCC	qRT-PCR
LcWRKY12-Fq	CACTGAGTCCACCACGAC	qRT-PCR
LcWRKY12-Rq	CCAGGAGTATTCATCGGAAGG	qRT-PCR
LcWRKY13-Fq	GAATGTAAGCCAGAACCAAGC	qRT-PCR
LcWRKY13-Rq	CAGAAGCAGGTGTCCTAACAT	qRT-PCR
LcWRKY14-Fq	CAAGAGCCCAACTTTCATCTC	qRT-PCR
LcWRKY14-Rq	ATCATCCAAGGGACCATATCC	qRT-PCR
LcWRKY23-Fq	ACAACACGAACACGACAACCCTT	qRT-PCR
LcWRKY23-Rq	CGGAGACAACTCACTTTCCCTAAA	qRT-PCR
LcWRKY29-Fq	GATTCTAAATGCCAAGTACCCTC	qRT-PCR
LcWRKY29-Rq	GAAGTTGTCCTGTGCGAC	qRT-PCR
LcWRKY33-Fq	CGATGCCACTGCTCTAAGAAG	qRT-PCR
LcWRKY33-Rq	CAACTGATTGAGAGTGATTATGGTC	qRT-PCR
LcWRKY36-Fq	TAGCCATGATACGGTGGTTC	qRT-PCR
LcWRKY36-Rq	GCAACTCCTGGACTGGAAT	qRT-PCR
LcWRKY38-Fq	GATTCGCTGATGATAAAACCGT	qRT-PCR
LcWRKY38-Rq	GTAATTCCCCAAGATCCGCA	qRT-PCR
LcWRKY39-Fq	CTATGCTTTCTTGACTAAGAGCG	qRT-PCR
LcWRKY39-Rq	CAGAATGTCCTCGGAGTGTAG	qRT-PCR
LcWRKY41-Fq	TGGTCTCAAACAATCCAAGC	qRT-PCR
LcWRKY41-Rq	GTGAATTAAGCAACCCAGAGG	qRT-PCR
LcWRKY43-Fq	TGTCGGAGAAGAATATGGTGAAGC	qRT-PCR
LcWRKY43-Rq	CAACGTCTGAGATTGTTGAGGGTAAT	qRT-PCR
LcWRKY46-Fq	TCGGAAGACCAAAGTACGATC	qRT-PCR
LcWRKY46-Rq	GGTCGGAAAAGCAGTCGT	qRT-PCR
LcWRKY48-Fq	ATCACCGAGTTGACTCAGG	qRT-PCR
LcWRKY48-Rq	GTTGAAGATGAGTTCTGAGTTGG	qRT-PCR

LcWRKY50-Fq	TGAGGGCTCTCTTGATGATG	qRT-PCR
LcWRKY50-Rq	GCAAGTATGGTTTCCTCGGTA	qRT-PCR
LcWRKY53-Fq	GTGCTGAGGATGTTCTTGATG	qRT-PCR
LcWRKY53-Rq	ATGTGTGCTATGTTTGACAGC	qRT-PCR
LcWRKY56-Fq	ATGGTATCGGAAAACGCCC	qRT-PCR
LcWRKY56-Rq	CCTCCACGGAATTTCTGCT	qRT-PCR
LcWRKY57-Fq	CAAGTGCCATTGCTCAAAAAG	qRT-PCR
LcWRKY57-Rq	CTGACCGTACTTCCTCCAC	qRT-PCR
LcWRKY59-Fq	GTCGTCTGGATTTCAGTTCAC	qRT-PCR
LcWRKY59-Rq	CGACGAGCCACACTTCAT	qRT-PCR
LcWRKY60-Fq	AGCAGGCTTTGGCTCAG	qRT-PCR
LcWRKY60-Rq	CTGATCTTTAGTCTTCTCGCCG	qRT-PCR
LcWRKY62-Fq	ACTCTCGCAACGATCAGTT	qRT-PCR
LcWRKY62-Rq	GCTCCCAAAATCCAACTCC	qRT-PCR
Lc18s rRNA-Fq	GTGTTCTTCGGAATGACTGG	qRT-PCR
Lc18s rRNA-Rq	ATCGTTTACGGCATGGACTA	qRT-PCR
LcWRY7-GFP-F	gagctcggtacccggggatccATGGCTGCTTATTCCCCTTGG	subcellular location
LcWRY7-GFP-R	gcccttgctcaccatggatccATGCGGGAAAATTTTTCCCG	subcellular location
LcWRY23-GFP-F	gagctcggtacccggggatccATGGAGTTTGTGGGTATAATCCAAG	subcellular location
LcWRY23-GFP-R	gcccttgctcaccatggatccACAGAACAACTCCAAGTTATCAAAACTA	subcellular location
LcWRY43-GFP-F	gagctcggtacccggggatccATGGCCTCCTCTTCCGGG	subcellular location
LcWRY43-GFP-R	gcccttgctcaccatggatccACATAAGAGAGATTGGAAGAACGTGT	subcellular location
