# Supplementary material for: Identification of WRKY Family Members and Characterization of the Low-Temperature-Stress-Responsive WRKY Genes in Luffa (Luffa cylindrica L.)
Source: Plants (Basel). 2024 Feb 28;13(5):676. doi: 10.3390/plants13050676 (PMC10935285; doi:10.3390/plants13050676)
Supplement: Supplementary file 1 [file plants-13-00676-s001.zip › Supplementary File S2.pdf]

>LcWRKY1

MDPDPPPDFSPADSSTDWTNSLASADPAYFFSADRESTILTEFGWNFHSHDLQPPRFLHSDNPDLPGTSAPPID
DGVSGGLQSPDAPATAGSLNDAVASNPSLSSSSGEPPEKQPEIPRKVKKKGQKRIRQPRFAFMTKSEVDHLED
GYRWRKYGQKAVKNSPFPRSYYRCTNSKCTVKKRVERSSEDPTVVITTYEGQHCHHTIGFPRGGLNMAHEAAF
GAQFSPQMAQFYYPEAQPLPRNNPPNAHQPQNLPTMASSNAAFEQKEAANSQPVPSDEGLLGDIVPPGMR
RR

>LcWRKY2

MEPSSTINTCLDLNFNPPPYTADESPATNTVTQLKEEAPAVLAEKLNRMSSENQKLNQMLGLVVDSYNVLKDQ
VIDLMIKSRKRKAGCDDCNFNRSGSGANAFTDQYCGCCSDDDSCHKRPRESSKPKVMRVLVPTPISDASLVVK
DGYQWRKYGQKVTKDNPSPRAYYKCSFAPSCPVKKKVQRSAQDPSYLVATYEGEHNHMKPNSGIEYQLVGPIH
LGGSKLDSSVPSPSSSVKSPSIPSVVTFDLTKSQSTDTVKASVPELERPSSAPSQQIIVQQMASLLTRDANFTRALA
TAITGTMVEKEIWR

>LcWRKY3

MGSEVECLRIELERLRKENEALKFMLRVVSMRNLVSQVGAFVEHHNDMNQNSNMRARADLQVPLALGASST
TQAYVRTNFKDPALMVKDGYRWRKYGQKITKDNQSPRAYFRCSSPGCPVKKKVQRSLKDKSMVIVSYDGHHN
HENDSPSPVSTSEQLSSSPSSQTVIHRLPQPPPPPLLEESNRVVQPASLDLDLDLDLTLSRGNKEDRKKPSNLKEEL
EGETLNIIKDCAESLTQNSGFTVALAAAVARSISDQPRSMES

>LcWRKY4

MAAGNDDWDLSAVVRSCNSASSTDPTAAAAAETALSCLASLTFDDDPNDVAFSFSDILQPQQQPNGFHELHQ
AFVSFLPNPAASAAVTTALPEIPNPTPSRHFRQGVKPIRPQPDPGAAALQPHHRQPPKNQQKRTVCHVTADNL
STDMWAWRKYGQKPIKGSPYPRNYYRCSSSKGCGARKQVERSNADPETFIITYTGDHTHPRPTHRNSLAGSSR
NRSSSSITTKNTTTGDPNRSLTATAVIGSCSSPAASPMTPLDGGGEKEAEMFEDMVIDSDDDDDDDDILIPNLSV
RDEIFVGFEELGCSPRRRPS

>LcWRKY5

MEVSSDHSLSLKPNPEEDDNLSAQLTPEATSKKRKVLQKTVVTVKIGSGKAAIGIGKMKNEGPPPDLWSWRKY
GQKPIKGSPYPRGYYRCSTTKGCSAKKQVERCKTDGSMFIITYTSSHNHPGPNISTLHLDQSQEEIQPQPLDQD
QEQDQNNLHPNQGLEKQDQDKNDEKNSIIPSDDEEEVEEEEEEIEEEEEEEKKAMECLEEPKVSSCSHELINLSA
TNKSELENHDHFFDELEELPIPPPFSSLMRSSCYFFDEVIRIPAAPS

>LcWRKY6

MEKKKEEIIKMEDTSTSGGGGGGGGGYLSFSDNMIANSFLDFSDGEKCSVGFMELLGLNNHEFKDYCSEVFNP
PATPNSSSVSSASSDAVNDDPTAADHKADDHDLQQQQQHKPTKQLKAKKTNGKREKEARFAFMTKSEVDHLE
DGYRWRKYGQKAVKNSPFPRSYYRCTSAACNVKKRVERSYADPTIVVTTYEGQHTHPSPVLTRSALGISVPPPP
STDISGGGCVGIAAMPWLKASNNSHEGNITAISHKYLQNPTFFTAQHMDADYNRNLIGAANMAGFLQEKRFC
NPNPAFLADHGLLQDVVPPHMLKQE

>LcWRKY7

MAAYSPWLDSVDTSLDLNIHPLRFSGEAPKERNYLDVERKVSVKEETGALMEELKRVSAENKKLTEMLTVVCEN
YNTLRGHLMEQMNKNGEKEISSSKKRKSESSNNNNITGVNGNSESSSTDEESYKKPKEETINKSSKTTRIQVKIG
ASDSNLVVKDGYQWRKYGQKVTRDNPCPRAYFKCSFAPSCPVKKKVQRSVDDQSILVATYEGEHNHPNPSQM
EATSGAARCVSMTPAALTAAAGGSSSAAAAVSLDLAKPKSSTEGKTTSSPRFESPELQQFLVEQMASSLTKDPNF
TAALAAAISGKIFPH

>LcWRKY8

MFVNCMEEIEKKKKEELKKRENLNWMGNSGMLFSDSIPSGMFDFPLDSTEYSSFKPSTFSDLLAIQDYNPSLFD
LFSPPSLPPPLPESSEVLNTPPTPNSSSVSCSSNERPLDADEVDRDNSPFNKQLKVKKKNQKRAREPRFAFMTKS
EVDHLDDGYRWRKYGQKAVKNSPYPRSYYRCTTAGCGVKKRVERSSDDPSTVVTTYEGQHTHQSPIMPRGAL

SSSISPAAAAFTATPPLVFPQSQYPHHHQQYTYTPAPPMGFDPVFHSLGEERRRIGGSSSNSDSFKDHGLLQDM
IVPSSSSLHIPKEE

>LcWRKY9

MEEVEEANRAAIETCHGVLNLLAHQPLQDQVQLSNLMVETGEAVFKFRKVLSLLNSGFGHARVRRFNKIPPPL
PNLPQRALLDCPNYRPDPPSKNLHPFQPGLNGKVSIFMGNPDLELGQNDKNSLQIPKQAAPSLNFSFPQQQQ
QQHQQRLLAHQKQMKQQAEMVFLRSNSGMNNLNFDSSNCTLTMSSARSFISSLSMDGSVADGSSFHLIGPS
STSADNKRKFPGRGDEGSLKCGSTGKCHCSKKRKHRVKRSIKVPAISNKLADIPSDDYSWRKYGQKPIKGSPHPR
GYYKCSSMRGCPARKHVERCLEDPSMLIVTYEGEHNHPKMSTQSAHT

>LcWRKY10

MQFMEDYGDHTRPSTSSTPKFADDEVLVDANPGSFAQRRPSKRGRNGFNAPKNNMPTPRFRTTSPPLASPM
VPSPCLTIPPGISPTVLLDSPIMLLNTQDLPSPTTGTFPIHPIKDEHSLLNPVMPEDGSCHGSEDSYFRFAPHGDSL
QNLLRLENQEADIDYQAFEPEKTLMDFEFLADFPKEASVLKYEVASSTDNDLFDGKIVNDNCDDMESCLTSIAN
NQSSIPEESTKREDIETQHPLEGEQKGSYIPMGILRTSEDGYNWRKYGQKQVKGSEYPRSYYKCTHPNCLVKKK
VERSLDGQITEIIYKGAHNHAKPDPNRRAVLGSVPTPDDTPEIGEGGGNRAKVEAGLTWRNTQYGVKDIKLISD
WSVGGLERTSSVSVVTELSDPLLNPQGKTVGAFESVGTPELSSTLASHEDGGGGDDDDDLTTQGSISVCMEAD
DVEPELKRRRKEGSSIETNLASRSVREPRVVVQIETEVDILEDGYRWRKYGQKVVKGNPNPRSYYKCTSAGCLVR
KHVERASHDLKCVITTYEGKHNHEVPAARNSSQVNSSNGNAQPSASHVQPNMGLSRNSNVPKSETQIQDLAA
QFYPKPEFNHDYQRSGCFDSFSNDMKLGAPSFCQMKFPPLHNTLPYSSFGLNSKHTATSISGSLASMVPDFPISL
PLNQNLSAAGFDFANGRPIPPFQVFLAGQQLRETDRFLTPKQEHDEENICASFQPIVDSSSGSSSSSVSSVYQQI
MGNFP

>LcWRKY11

MEAEPLPTKDDRVQPIGGDHEEAAAKQDVLHKVTFMADLEKPSMEPLSVASASSTWKEEDDHHHEQRIKMA
KVEMSQVREENQRLKKSLDQMMKDYESLKRQFHDITTQREGKKSTQTSASTINNDEVEEVDDMVSLTLGRFSS
CDQNKNNTSSERKLDHKLLELKTPSNNDHIQSPTDSEAKDQEEAGETWPPSKALKGLPAPATGEDEVSQQNPP
KKTRVCVRARCDTPTMNDGCQWRKYGQKIAKGNPCPRAYYRCTGAPSCPVRKQVQRSVDDISILITTYEGTHN
HPLPVSAMAMASTTSAAASMLLSGPSSSSTSRPGLNPSSSISTAANLHGMNLYLSNNTNTKQFYLQNSSMLSSS
LNHPTITLDLTSNPPSTSSSSSPFQYKIPSTYPPKYPFTSLDFGSSQPNNFMSWNNNNNNQPYNITKNAIGIASSD
LSKQLPLHSNIYQACLQHFAKPSTPPLPLPPPSPLPDTIAAATKAITSDPSFQSALAAALSSIIGGEGIQPVSNVCGS
SMGFQGPPSLICSSSKSPSSSPGDSRDNNTK

>LcWRKY12

MAVDLAAFPPSFDHQAAIQEAASAGLQSMEHLIRLLSKQQSSPTLNHPHLDCSQVTDFTVSKFKRLISLLNRTG
HARFRRGPSPVSDSPNPVLNSLDPPLKSPNPTSTVDFLKPNLTPPPKSPDSRAPTESTTTSSFMSTVTADGSVSN
GKLGLSLFTTPAVSSGKPPLSSKRKCEDSSRLACKISSKPCHCAKRRKSGDKKTVRVPAISSKIADIPSDEYSWRKY
GQKPIKGSPYPRGYYRCSTVKGCPARKKVERARDDPAMLLVTYDGDHRHPQATVPRAVTDARVGFVSQKC

>LcWRKY13

MAEREGFETDRLGSSKATAGQEDDEEEEMEVSEDESEVELEEGGGVSELQPTELRAGSSVSEAAVRGSPSETLA
APSANRSSENGQSDGLPVNSSAQSLEGAELKQAPSSRNEPLAAEATQTDQVQEQSQLQVSTCKGTDSGRSPTS
VTQSISSSTSPSLSEHKLSPKKVHKECKPEPSQKSSSDHKTALSVPNVRTPASDGYNWRKYGQKQVKSPKGSRSY
YKCTYSECCAKKIECCDHSGLITEVVYKSQHSHDPPRKINNPKESKLVPYVEPVVKKIIAEHSRRIINDSDPPTPSKE
TVRETALVLERKRQHSNDSDGNDEFKIKDENDNDSETKQKVKKSSGGYSGTPLKPGKKPKFVVHAAGDVGISG
DGYRWRKYGQKMVKGNPHPRNYYRCTSAGCPVRKHIESAVENPNAVIITYKGVHDHDMPVPKKRHGPPSAP
LVAAAAPASMSNTQPKKTDAVQSQISSTQWSVDAEGELTGEALDLGGEKAMESARTLLSIGFEIKPC

>LcWRKY14

MESLAAIPWSETYNATSEDDLLGLTDLLHDDASPLFLLPQDVDDDRPTKLPVPGGPAYFGPTIEDIENALSTAPRS

KDLQSNTHISHAVTGFSIVERGSLNKVEHKYSLRIKSCGGNLVADDGYK**WRKYGQK**SIKNSPNPRSYYR**C**SNPR**C**
SAKKQVERSIEDPDTFIITYEGL**H**L**H**FAYPFFLMGQTPQAQSPTKKPKTINPDPEAQFHEAHKSPTFISPGPLPPD
DPKEETGPQGLLEDMVPWMIRNPSTNNNTLSNSSSCSSYRSPPTSPPSPSTCPTFLASCF

>LcWRKY15

MGSPASDEIMVNQLVSLLQAEHAAAAITTNFNFFSFPENTTAVDLHQQSSLKPFFCTSTEPPHYLSQQSQPPLLF
SLKPPSLLHFPSSLPSTTATSSDLWDVGEGFVRGRNGSGDSGGDEDRKPPRLRVSATAKMKRIKGRKKVREPRFC
FKTMSDVDVLDDGYK**WRKYGQK**VVKNTLHPRSYYR**C**TQDN**C**RVKKRVERLAEDPRMVITTYEGR**H**A**H**SPSH
DNLEETMGQLPSGHFNNFFW

>LcWRKY16

MNMNSPQNPTFFNHHQFQDSSSSFMDLLDFSGYPLPDFCLEPETATLSEPIIDGRSRSMEATSTDNNTMDGW
CEMKGVKRKKERGGGCNRIAFRTKSELEILDDGFK**WRKYGKK**SVKNSPNPRNYYK**C**SSGG**C**GVKKRVERDRE
DSSYVITTYEGI**H**N**H**ESPFLVYCNDPIFHPHATWPSSLYHSSPPYSSTTTL

>LcWRKY17

MAVELLPHAHPLSSTHNMEANAVQEAASGLESVEKLIRLLSNAHQQHHSLPSIQSPNSNSNSALDFPTDCRAA
ADAAVSKFKKVISLLGRSRVGHARFRRAPLPLPQTPQVHYATPIQQIPPHRHHPPGDLDKKEPLTTSVNFSYSSA
HNSFISSLTGDGDSKQPSSSSAFQITNLSQVSSAGKPPLSTSSLKRKCSSDNLGSGKCGAASSSGRCHCSKKRKLR
VKRVVRVPAISLKMADIPPDDYS**WRKYGQK**PIKGSPHPRGYYK**C**SSVRG**C**PARKHVERAVDDPAMLVVTYEGE
HN**H**TLSVPETSNLILESS

>LcWRKY18

MDKGWGLTLRDSDQSIGFFSNKPPPPTVNSFQRMFQGIEFSGKLGRTDDDNPAPLPSDENRLVVNEVDFFSDK
KRVVDDTREDQDSKSSINIIATAVNKDDKNLTAPRTGFNLVNTGLHLLTANTGSDQSTVSDGISSDGEDKRAKNE
LAQLQVELQRINAENHKLRDMLSHVSNNYNTLQMHLLTLMQQQQQQNQATEPAHEREIGERKSTEKHEVG
RVVVPRQFMDLGPSGNTAETDELSHSSSDERTRSGSPLNNAETGSKKSTGRDHEIAPSDHENSNFRDGKRSSTA
REESPESESHAWGPNKAPRLNSSKPLDQSTEATMRKARVSVRARSEAPMISDGCQ**WRKYGQK**MAKGNPCPR
AYYR**C**TMAVG**C**PVRKQVQRCAEDRTILITTYEGN**H**N**H**PLPPAAMAMASTTTAAASMLLSGSMSSADHNLMN
PNLLARAILPCSSSMATISASAPFPTITLDLTHTPNPLQFQRPAATPFHVPFAGGQPPSAAAAAQLPQVLGQALY
NQSKFSGLQLSHEMGANSSHLGHPQIAQPAAASQPGGASFADTLSAATAAITADPNFTAALAAAISSIIGGAHS
NNNTTTTTNTTSTTTNNNNGSNSSKISSFPGN

>LcWRKY19

MSDEMFRDVFFSDSFRDGWLGDLESGMDEYDESLARAFGVSGSSNNNEISGTPMNSCGSLSSSDAGAEEDD
SGKEKDKQIKEMDDDSGQTSKPAAKSKKKGEKKEREPRFAFMTKSEVDHLEDGYR**WRKYGQK**AVKNSAFPRS
YYR**C**TTQK**C**GVKKRVERSFEDPSIVITTYEGQ**H**N**H**PIPATLRGNLSAAAAFPPSMLAPMPVVGGGAAFLPELLT
NTSGNNQAVGGSVYSQSSFGYPYNGRQPEYGLLQDIFPAPSSFYNRQP

>LcWRKY20

MCSLFEMENYQGDLTDIVRGRSTFGCKTEDPFSSEWQFSSEMAMNFSSSSELQEQAATAREEEYSFGDPFCAA
AMRDPLLQELDICGNNNNNSSSSSSSSAFFNGGGNLEDKSGGGGSVFGVSSCLHEDELIKRPCNIFSRMLQISP
ATITNNKFPISASPCDSPLLIPNCNSPTHPLIHANNSDLHHFVDNNPSALQISSPRNNPAGIKRRKSQARKVVCVP
APVAASSRPNGEVIPSDLWA**WRKYGQK**PIKGSPYPRGYYR**C**SSSKG**C**SARKQVERSRTDPNMLVITYTSE**H**N**H**P
WPTQRNALAGSSRSQQSKNNTPNSSKLSSTAQPQKPTTTTQEGSDKTKEDQTNNNNGTRSPAASNNVKEEAV
EEIEKPFETEEGDQFSEGFQYRVAMGSNNNQSDDFFADLEELETDPLNLLFTQGQKLDQISKGGGLDDVVAFN
NLFDWAAENNNSNNNSNNSFEEQPPPATASKRGFY

>LcWRKY21

MEELAASDSSRCPFPYAFECAEDGKSRLGFMELLSIDQDFSSQFDMFSTSSLSSSLIPNLNIDSDNLEIWNRWPA
TPNSSSISSTSSEVVNDELTELNLEGGQKQHQQETVKTDKQSKTKKTNHKKKEQEPRFAFMTKSEVDHLEDGYR

WRKYGQKAVKNSPYPRSYYRCTSVACNVKKRVERCLKDPSIVVTTYEGQHTHPSPVMARSTFFPPPISAALYGG
SPSFTTATVSTATPSLFHYQNAHNSDFISHPNGFMASSFHHERRPWSVASYDRATHFLAAATDHGLLQDVVPTN
MMSWHGEMV

>LcWRKY22

MEEVEEANRDAVESCHRVLNLLTVASPQDHSKLRSCLMAETGQAVFKFRKVLCLLDSSGLGHARVRKKKVKSF
QFNSSSPFPLPQSMFLETFSPNCRMDLLQGRNLQMGPLSLGSPSLELSSNGKSCSIQQQQQQQPQSVGHYHH
HFLQNRVLLNSNPNINPNASQPEMVYLRSNSGINLNFDSSSCTQHTMSSTRSFISSLSIDGSVANLDGSAFHLIG
APRSSDQNSHHKRKCSGRGEDGSVKCGSTGRCHCSKKRKHRVKRSIKVPAISNKLADIPPDDYSWRKYGQKPIK
GSPHPRGYYKCSSMRGCPARKHVERCLEEPSMLIVTYEGEHNHPRIPSQSANT

>LcWRKY23

MEFVGIIQELNQGKQLAQQLRNHLRPSPSPSSSDGILLIDKILRSYENALSVLAGGCGGDEELAAVKSAVAPMN
AAVKEGDVSKKRKVMAKWSERVKVPSGSAIEGPLDDGFSWRKYGQKDILGSKFPRGYFRCSHRFSQGCLATKQ
VQKSDNDPTMYEITYKGKHTCNRVLHSNTPKEEQKPLLQHPNQPKQEQKPLRQQHEHDNPSCFTFSSDAIRVK
SEKLDDVDDGLSRPFRTLSSSWFGSEVRDDQSPFRESELSPTFESSDMFGLCRDGVRDWDFVTEFVSVPNSVT
NISIGDLEEYYSFDNLELFC

>LcWRKY24

MEAAAAFGRPRPVVKTEKPTGDVDGDGDSPSKQRLLVKMGNNYGKQEDKTSNSSDQKDLSCTKPQEDQLES
ARAEMGEVREENQRLKMSLSQIMKDYEALRTQFLGIVRREGKKLSEDDDKLNNEQHHDDDDRNQTDETELVS
LTLGRFPTTEKNKNKAADEKLDQNNKSSQGREMIGEEQGVKETLSLGLNCKFEQESTVVKEVDSPNRSLTNSFD
HEAKEEAGETSWPPSKGVKTMRSGDDDVAPQNPPKRARVCVRARCETATMNDGCQWRKYGQKIAKGNPCP
RAYYRCTGSPTCPVRKQVQRCADDMSILITTYEGTHNHQLPVSATAMASTTSAAASMLLSGSSSSSSTSATANLH
GLNFYLNNPTNNNNPKPNFYLPNNSSIISSTSPTHPTITLDLTSNPSSSSTSISAHLGKFPTGFSNPRYPFTSQLDFG
SSRNNVLSWNNGLLSYNRNNNPTATSTTNAIYQNYIQQQQQRNNNLNHHGVVAAAATSLPHQQQPLPDTIA
AATKAITADPSFQSALAAALTSIIGTGSGAGVIKSSARGEQPLFQLTSTAANKGNGCGTSFLGNIAAATATTTKSNS
PPAGNVVFVPASSLPFSNPKSASASPGDHIDLTN

>LcWRKY25

MDASFNNNHQFYSGDPLEDSDEKAPDSPPPNSTAKKGRRGMKKRVVSVRINGDSPRNSSGSTTPPSDSWAW
RKYGQKPIKGSPYPRAYYRCSSSKGCPARKQVERNRLDPTMLVITYSCEHNHSGPVSRNNNNNNNNIHHHQA
AVTKPGSPETVPVQAEAEPEVEEKFVDIGEESLITGDEFSWFGEMETTSSTVLESSIFSGRSSGVGDSISSDVAML
FPMGDDDVDESLFADLGELPECSVVFRRGGGRGLAVEEQPAAGQRRITPWCGTTT

>LcWRKY26 Chr06:11318747-11322452 PREDICTED: probable WRKY transcription factor 14

MFCSLFEMDNNSYQQAGDLTDVIRAGSAAAGLSSEFSLDPSSGDRRLWPHLPADDPSMSFGDPLLFSTGAAR
DPFLHPHFSAFNFGSDGGGGVGGLAAAAERPCNIFSRMLQISPSSDGVISSPPCESPVVGCSPSSTSTKALACG
GGGGGGSIVISSGGSNPLCSMDNSGLQISPPRNPTIKRRKSQVKKVVCIPAPAPANSRSSSGEVVPSDLWAWRK
YGQKPIKGSPYPRGYYRCSSSKGCSARKQVERSRTDPNMLVITYTSEHNHPWPTQRNALAGSTRSHPSRTTVTA
HKTSPKHEHTNENPTTAAVKEEEMDEDQTTEKATATTTTNDHKKEGDPQEFSYDLIFAEFEDQINDNHYHHHH
HSADPLNNNLVLFSSHGFSTGNDQERSKDPFLELYDWAENSNESLFKEAKGG

>LcWRKY27

MDNKAAERVVIAKPVASRPTCSSFKSFADILACAFNTSPPKTSSETMVSAIRPKTARFKLKDNPAPSPRAKISETLP
GTTSRSSSENLTLSDSKSTVLFKPLAKHVSKRTVSQLSLMGNTNLQNHLPPPPVEARIECLNQGKDNIKSALNSN
LPRNITSAVENSQSIESSRVTLNYSKEDPTSLCPRISCAQPSYDGYNWRKYGQKQVKGSEYPRSYYKCTHPSCPV
KKKVERSLDGKIAEIVYKGEHNHPKPQPLKHNSLATQGEGSISNGTARDTNSELWLHYLNGQIEGCESRLENQID
KTCQGRVTQSFVPVAIREVNTGCGISDNSCGLSVEYEEGSKVLVPMGDKLRSKRRDGKNPTNEADTSSEGVKEQ
HAMARGCTDIEISGKGIRWRKYGQKVVKGNLYPRSYYRCTGLKCKARKYVERASDDPDSFITTYEGKHNHGISL

GNANPLTPEME

>LcWRKY28

MNNINQAINALAGGSSDNRTNNFAMEAPKFKSLQPPSFPLSPSSYLSAFSSGLSPTELLNSPLLFSFGVFPSPTTG
ALNSRNDFDGGEQQEEMKGDFKNYSDSLFNPQTGSSVSSYFQSSSSPLNPGGLSCDESGAKSEFVASEMAAA
QTKQISQLPLYNREQHKSENDGYN**WRKYGQK**QVKGSENPRSYYK**C**TFPS**C**PTKKKVERSLDGQITEIVYKGS**H**N
HGKPQPTRRSGGSAVYDPAAAESAVLQEDSSVSVGDEELEPNSPFSNSVDDNENEPVAKRWKGENENEGFSG
GGSRTVKEPRIVVQTTSEIDILPDGYR**WRKYGQK**VVKGNPNPRYTSHSSMFHLFTVFAKCNFFTFSLLFKVKFYF
TFNSNSLSTLLQFLIIVFLISIPLNSSILFLPFG

>LcWRKY29

MDCSWPETTPFDRRKAADELLRGREFAQQLGALLRRNSSSGGAASQEDLLSRILTSFSKTLSILNRCDSDDINGSI
VDSPDDARKSEESGDSCKNNDRRGCYKRRKSSHSWARESCSLVDDGHA**WRKYGQK**VILNAKYPRNYYR**C**THK
FDQG**C**QATKQVQRVEEDPPKFRTTYYGH**H**T**C**TNFLKASEIVLGSSNFNDSCGVLLSFDTPTVAQDNFFLPADDD
VKKELAVCSPSDYMSAGPSPDHLSEVIMGSVDFEDDVLQFDF

>LcWRKY30

MGSKSQVLLNPQALFEDQAPSSEAAAHSQMGFFSFPSSNLTFFQLPSMPQTHSSSSSPPFEPPNFSTTSNININ
NNTLSETLLSSSLLPLKPSLLSPSSHFAPQHLLSLQTSTPNLWPWGEVGERLMSGKRNEYQLGVSTMKMKKMK
GRRKVREPRFSFKTMSDVDVLDDGYK**WRKYGQK**VVKNTQHPRSYYR**C**TQDH**C**RVKKRVERLAEDPRMVITT
YEGR**H**I**H**SPSHDSEDSEAQTHLNNFFWYPLSLSL

>LcWRKY31

MASFPNDDPNPNPDPNPNLNYTHFPPILDPNSFFDFELSDFLVFGDDENVDQTASTSPSIASSEKITGVDSGNS
CSAVDSGSSTVVSSGTSTSSIRSKNEEKKRKREMVFRVAFRTKSEQEIMDDGYK**WRKYGKK**SVKNSPNPRNYYK
CSSEG**C**CVKKKVERDREDASYVITTYEGV**H**N**H**ESPFVVYYNQMPSFASTPT

>LcWRKY32

MGSSSEITVNQLVLRSPLQPQPAADASSSSPIIQQSLPRNVEESDSNDNGDHNVCKRQLKVSTKEMKVRKREG
RKKVAEPKIYVIIKSDVDVLEDGYK**WRKYGQK**VAKNSLHPRNYYR**C**TQEN**C**KVKKRVTRLDKDPRMVLTLYQGR
HI**H**SIPLQETMGQPPSDHLNNFFC

>LcWRKY33

MAVELLTAFTNAQLSAPMDQDSAVQEAASGLDTLKKLVSLLSHSPPSNLDSDCQAVANAAVSHFKKAISLLGRS
PRTGHARFRRAPLDSSKVYNATPIQQIPPPLERRESATTINFSYSSAPSGSFLTSLAGADSEIKVQHQPSSSFQITDL
SRVSSVVSKPSSGLKRKCGSENLGSGKCSGSSGGRCHCSKKRKMRLKRVVRVPAISSKNADIPPDDYS**WRKYGQ**
KPIKGSPYPRGYYK**C**SSLRG**C**PARKHVERASDDPSMLIVTYEGD**H**N**H**SQSVAEASSLILESW

>LcWRKY34

MSNEDHQKDSCYQKFDPFTHNFHHHKPFEAPLLSPYDAFDPSYVGFFSDFLHASADYDYNNILSAGLEMSCSSS
EVISPIDEASKKSMGVGESVVTGEHPSTPISSTASSSDEAAPGGGDSAKSGEEVKEFEDKSGENCKKGNGRKIKK
EKREKGPRFAFLTKTEIDHLEDGYR**WRKYGQK**AVKNSPFPRSYYK**C**TTQN**C**SVKKRIERSTEDPCFVVTTYEGK**H**
N**H**HCPITLRGQNAAGVLPASASPPLLPGLAPANFFSGEVYEGVDLLQQYYQVPDYGLLQDLINPLSFNRKQEP

>LcWRKY35

MEFFFSASVEDPTYGRRRLPERTAPPMSKLISTENPPLLPALSPPLFSPSSFFTIPPGISPTQLLDSPVFLNPSNIPPS
ATTGAISAGDLTGNRNNSGHHQQNIKQEHNNLPDFSFSRTHPTKSSSMFQSFSTQQNRPWSSQPEPPVSFPPE
TTISGGSKRDHRPQSQSRNRASDDGFN**WRKYGQK**LVKGSENPRSYYK**C**THPN**C**PVRKQVERSLNGQITEIVYK
SK**H**N**H**PKPEFTRRSSSSSCSSSSSWLSEALPQTMAAEPKHPAASDSMPTPENSSITIGDDESDEADAKRWKSES
ENEMVSAAGGKTVREQRVVVQTISNVDKLDDGYW**WRKYGQK**VVRGNPNPRSYYK**C**TYAG**C**GVRKHIERAS
HDLRAVITTYEGK**H**N**H**EIPAARGGGGGRPVLNNHGSSNDGASNNGNGDRRPSSEISGGDNGLSNMLWRTKE
EAADDIFQSLLD

>LcWRKY36

MVSSGDQVDNEVDSDRLEHEQSSDSQSQASQEDPGGTNASKSDIKCTAAASKTLEEAVNLPDVTIEQVDRGGI
SNIVSEKVTHKPTTADQDSHPDLKVSITSTIREKVSEDGYN**WRKYGQK**LVKGNVFVRSYYR**C**THPT**C**MVKKQLE
RTHDGKITDTVYFGQ**H**D**H**PKPQPHIPVAVGVAMVEEKLDEHASRSSQDKTSIALGQTPHQTGPVDTLQLSSVT
ASDNVKDEALKRSRINDEDDSDDTPDLKREKKRCNIDVTVADKSTVESRVVVQTPSEVDILNDGYR**WRKYGQK**
LVKGNPNPRSYYR**C**SSPG**C**PVKKHVERASHDPKVVLTTYEGQ**H**D**H**DMPPARTVTLNSVGAAAAHSDEIKTKSV
GSSISHDTVVHASKDPRSDSSSEGKLIEKNGTSNATGASDGIVLDMVVNSSPGVASGQNKQLKVAIES

>LcWRKY37

MDAAPSPPPPTRHFPVNLNSTLHDSDPPPPPPPPPPPPPSSYEMNFFAADDKSRVLLAKLPASDNLDFNVNTGL
NLLTTNSSSDQSMVDDGVSPNQEDKRAKNERAVLQAELERINAENQRLKEMLNQVTSNYQALQMHFTTLIQ
NQKAGDAGDPMDEKAGGGGGGGQEKVRHGGGGGNNNSNKLVPRQFMDLGLATNTDTDEPSMSSSEGRS
GERSRSPGTTGEVASSKRHSPDQGSNWGSSKVPKFSSSSSSSGKDVDQTEATMRKARVSVRARSEAPMITDGC
Q**WRKYGQK**MAKGNPCPRAYYR**C**TMAAG**C**PVRKQVQRCAEDKTILITTYEGN**H**N**H**PLPPAAMAMASTTSSA
ARMLLSGSMSSADGLMNSNFLARTLLPCSSSMATISASAPFPTVTLDLTQTPNPLFQRPAAGHFPIPFAAGPPQ
SFPQIFGHALYNQSKFSGLQMSKDMEVPPPPPQNPLADTLSAASAAIASDPNFIAALATAMTSLIGGSHHQKEN
SNGNNNVDNNTTSNSQQ

>LcWRKY38

MDRRVRTNPFLSEQEDPEATSDDGLPESPSDGNDSKPAAGPPPKKSRRGVQKRVVSVPIGDVEGSKSKGEAYP
PSDSWA**WRKYGQK**PIKGSPYPRGYYR**C**SSSKG**C**PARKQVERSRVDPTKLVITYAFD**H**N**H**QLPATKSHHHHHHN
SSPSSASIAAAVAVPDDSSPGSTTTSSSTSSGDNTNATPTSPTAAKFEEAAAVFASQPELELGGDSLMIKPCIGDF
GWFADVAYDRILEGPICGGGDIFDDADVMVLSTRGDDEEESLFADLGELPEGSVVFGRRRAVEPDGPNRTCGR
VLDC

>LcWRKY39

MSNDEGKNNLYQQYDPFHYNQLDMNRSIFQQESLDPAFMSFTNFFDTSLDYNSLSRAFDVSCSSSEVISAVDD
MSKIKKAAAAAASSGGGKSSLNNNNQSSTPNSSVSSSSNEAVAEEDSVKSNKDDTKGSQNKDEEKSSKKQNIAK
KKEKRQREPRYAFLTKSEIDHLEDGYR**WRKYGQK**AVKNSPYPRSYYR**C**TSQK**C**LVKKRVERSYQDPSVVITTYEG
Q**H**N**H**HCPATLRGHSAGMMSSPFYASASTSVTAASSGPTLPQELFSHLLPITNCPTDPASIMYQNLNLQQHLQM
PDHYGLLQDLFTQK

>LcWRKY40

MDNYQIFFPASSSSNNSVPMGMELLTEEDHQEEEEEEEEEEEDDEIKNNNGGKLKGETKKIRKPRFAFQTRSQV
DVLDDGYR**WRKYGQK**AVKNNKFPRSYYK**C**SHQG**C**KVKKQIQRLTNDEGVVLTTYEGV**H**S**H**PIEKPQDNFEHIL
THMQIYPSF

>LcWRKY41

MAKKDDSARAPLQRPTITLPPRPSIEAFFAGGPTGVSPGPMTLVSSFFADAAVDSPSFSQLLAGAMGSPMAMG
FLGTGSTPNYYAKDGGASELEFGLKQSKPLNLVVARSPLFSVPSGLSPSGLLNSPGFYPPQSPFGMSHQQALAQ
VTAQAALAHSHMHMQQAEYQHSSVPAPTETLTRDPSFTLDEASQQSILPSTSDTKNLIAESTEVSHSDRKYQPP
PPHASDKPADDGYN**WRKYGQK**LVKGSEYPRSYYK**C**THLN**C**PVKKKIERSPDGQITEIIYKGQ**H**N**H**EPPPANKRA
RDNSEPTGCTNSLMKPESASQNQAGILNKSSENVQLGSSDSEEQADTEITDDRDEDEPNPKRQNIESGTSAVAL
SHKTLTEPKIIVQTRSEVDLLDDGYR**WRKYGQK**VVKGNPNPRSYYK**C**TSAG**C**NVRKHVERSSTDSKAVVTTYEG
K**H**N**H**DVPAARNSSHHTANNTVPQIKPHKVVAQKHPLLKEMEFGSNDQRPAVLRLKEEQITV

>LcWRKY42

MENYQMFFPCSGGGGLSAAYHQADMSSGGGSDMFGSFHGGDLQGSGFLGLKTEEVDAAAVEGGEQYQIG
GGGGKKKGEKKVRKPRYAFQTRSQVDILDDGYR**WRKYGQK**AVKNNKFPRSYYR**C**THQG**C**NVKKQVQRLTRD
EGVVVTTYEGM**H**T**H**SIDKPTDNFEQILSRMQIYSTPF

>LcWRKY43
MASSSGSLDTSANSHPSFTFSTHPFMTTSYSDLLASGTNDPPSSAAALRGSGTGVPKFKSLPPPSLPLSPPPLSPS
SFFAIPPGLSPAELLDSPVLLSASHVLPSPTTGSFPSQSLNWKSNSGYNQQSIKEENKFLSNFSFQTQSSKPPPTSF
QPSSTTAPTTQGWSFQEQGKKQDGFLSEKNMVKPEFGSMQSFSPEYGVVQNQSQNNGGGEFQSDYGNNYP
QQSQTLNRRSDDGYNWRKYGQKQVKGSENPRSYYKCTFPNCPTKKKVEKSLDGQITEIVYKGSHNHPKPQST
RRSSLSSAGSSHAMAASNPAANDMGDQSFTTQGSGQFDGVATPENSSISIGDDEFDRSSQKSKSGGDDFDED
EPEAKRWRRDGDNSEGISAAGSRTVREPRVVVQTTSDIDILDDGYRWRKYGQKVVKGNPNPRSYYKCTNPGC
PVRKHVERASHDLRAVITTYEGKHNHDVPPARGSGSHSLSRPFPQSEPPAAAIRPSAMTHQSNNGGNNNNN
NLLQGLRLQQSSENQMPFTLEMVQNPNGFSFPEFGNSMGMGSFMNQTQSNDNLFSRAKEEPRDHDTFFQS
LLC
>LcWRKY44
MMENFPNFRSTSSSSSTSFSLQQMLISRASEIAGIHGGHFGQTPISDGFPLFRDVSTNDMSVDATRSTKDDVVS
MSKSFFHVDGSAEIGVDRLEKKAVAGGATTAVTMTTGRGDYDKKKKTRSRRFAFQTRSQVDILDDGYRWRKY
GQKAVKNNKFPRSYYRCTHQGCKVKKQVQRLTRDEGVVVTTYEGMHSHPIEKSTDNFEHILSQMQIYTSY
>LcWRKY45
MEKKFEDSLMGSENSAGFYSPALFSDDFPATGFGFSGMFDMPCDDDHKASCSSNSFGIGIHDLYNPSSLFDLLS
TAAPPLQQPLSSPASTVPESSEVLNAPATPNSSSVSNSSSNEAAAIEEVDKNNNTDKTSKVLKPKKKNQKKQREP
RFAFMTKSDIDHLDDGYRWRKYGQKAVKNSPYPRSYYRCTTAGCGVKKRVERSSGDHSIVITTYEGQHTHQSP
VMPRGSIRVLPESTNCLVANHDAATPGLLFQHNTPQPFMYSPPPPFLTIDSSSVSPHPPSSLPPRSSRDSLLRDH
GLLQDLVPLQMRKEPKDEQNG
>LcWRKY46
MKMEVDWDLHAVVRGYSAVSSAASTAVTAAVPSSDFYSVPSISNNSIPLAFGRDPTNQTNHLFSLQDPFQGPN
CNSTEELHELFKPFFHKSQQPSPPPPPPPPSAPPLLSSPAGKILTHQKQQQNTHLPKQLHSSPVSAPRSKRRKNQ
LKKVCQVPAESLSSDIWAWRKYGQKPIKGSPYPRGYYRCSSSKGCMARKQVERNRSDPGMFIVTYTAEHNHPA
PTHRNSLAGSTRQKPITPTPAAAEPGSGKPDPKQPGCSSEDQSTITESKEEKEELLAEDEEDDDLGVSDLIVNDD
FYVGFEELDSPIADDCFSDQFPANFELPWLFNNAAAAASSI
>LcWRKY47
MEEHDQQFPPPPPFPPPSCTDPHAASLEIDWIAVLSGQVTGDFSPAAATCESSEMKTDEEKGNSMRNGGRR
WRRDARRRRFEFQTRSAEDILDDGYRWRKYGQKAVKHSLYPRSYYRCTHVTCNVKKQIQRLSKDTSIVVTTYEG
IHNHPSHFLMQTLTPLLKQIQFLSTF
>LcWRKY48
MEHKSLITELTQGKEMAIQLRTHLQPSSSSPEACFFLTEMIQSSFEKALLLLNYYSNPSDSIMAADPSPKVSFVEEE
STKNKRQSSKSSDVSKKRKLLPRWTEEVKVCSGTAPEGPLNDGYSWRKYGQKDIHGATFPRCYYRCTHRHVRG
CLATKQVQRSDNDSNIFEVTYRGRHTCNQSSNLGSTSISAQNEIPEETNQNQPNPISEILFNFGDQQHFNVKTE
DHFENLFPPFSFPSSSIGSAIGDENVFYEPTMEPAFITDTGLSAVTVSPAASEKAWDWSFNDGGDGGIQRVQSS
EQSDMTEIISATTSVTNSPINNGDWDFSLDKVDFDQNFPFDCLDLDFIC
>LcWRKY49
MEGSERSGVHNYQVQVSFLSNAPHPQPIHEMGFVQFEDHNQVLSFLAPNNAATTATAPTATMPAAFPSPHAH
LVSSRPSWSNEQVGTLDPKPAHDENCTGSASDGSNSWWRNSNADKNKVKVRRKLREPRFCFQTRSDVDVLD
DGYKWRKYGQKVVKNSLHPRSYYRCTHSNCRVKKRVERLSEDCRMVITTYEGRHNHSPCDDSNSSEHEPFTSF
>LcWRKY50
MENLGEWEQKNLKNELLKGKELAKQLQIHLNVRPSSSSMAASSSSSSSSHDGGEILVQKILSSYEKALSLLCSNG
IQRSESPSSLNGSPRSEDSDHELRSASRKRNILPTWTQKFQVSPGMALEGSLDDGFCWRKYGQKGILGAKHPR
GYYRCTHRNLQGCLATKQVQRSDDDPTVFEITYRGNHTCSQVSNLSTPSAITPEFQQQNNRIESNLVQSHPAVH

DQKVQNQQTSPDALLNSWASLRVITENLDTTHEPTLFPPFSYDPTSNYEAADRVESTSAVDVNFAEFSPSFLSPT
TSGSGLSYFSASSSGLSEGFVGNQNLQGRKSELSEIFSSPTSGVNSQTLGLEFPFGGLEMEPSFTFDNTNFFS

>LcWRKY51

MEIDLSLKIDHHREKSQQKDEENQEEEEEKNHQLQEKEEMGVEEEEAEIDLAAASGLKVFLPHNINAGEISELQ
MEMDRMKEENKMLRKAVEQTMKDYYDLEMKIAIIQQNNLHKKDSHNFLSFHGNENKRQEEPKPDHLELGET
AKRRRVRSPSKEDEMRERQLGLSLGLHTDNDLEQENHKEEEETREKNKELDPTTNFNSIQNKPQRPELQGMAP
PQNRKARVSVRARCEAATMNDGCQWRKYGQKIAKGNPCPRAYYRCTVAPGCPVRKQVQRCLEDMSILITTYE
GTHNHPLPVGATAMASTASAAASFMLLDSTNLSLPNPQNPNILNSSSYSPTFLGSANPNEPSKGLVVDLTSNNF
YSPMASSSSSALAQPSNWMKTNSHSYYQRNFQANLLSPFDGRTWKPAEDNKPPLTAESVSAIASDPKFRVAVA
AAISSLINKENNHPTAHPVEHSSFGPNKDGEGGGGGGDNGGGNKKWVVESLSTNGN

>LcWRKY52

MESGWSWDQKSLIGELIQGMELTKQLRAELSSASAEESRGSLVQGILSSYEKALLILKWNGPMSQPQTVEPTPG
LPGSPISVNGSPSSDDSGRGLMGNQDPRKESKKRKTQPRWTEQVKVNSETGFEGPHEDGYSWRKYGQKDIL
GATYPRSYYRCTFRNTQNCWAVKQVQRSDEDNSMFEITYRGKHTCSQGNYLAQSCHSPEKKENDHDHDHHQ
KQPLQENLLSNQTIENIEKLETKASTFCFGSTSVGYKDIVNGGFSHLAIDTHTALGSFSQSFISPTTPDSNYFTPSSC
QRSNVGGTHNVQHPESDVHEIFSANNSATNSPILDWDFPFDSDQINPNFPFNSSGFFY

>LcWRKY53

MANSCSSSSSLMMMMNCSTDQVIDGSGCCEIDWAGLLSGSCLSLEMEKKESGSGAAMETHEDYHGGKKISN
KGKMVMGSKRSAAMPRVAFQTRSAEDVLDDGYRWRKYGQKAVKHSTHPRSYYRCTHHTCNVKKQIQRHSK
DPTIVVTTYEGIHNHPSEKLMETLSPLLKQLQFLSGI

>LcWRKY54

MEDWDLQAIVKGCSSRPNSDPFYSSFLSEEDDFSCVYPQLFETTATTTSSSSSHEFEGFGRSIYPLIPHPNNSSSIS
DLLREFKEPEKLHQKKQIAATKQKQSKKSRQNRVVKEVKADSVCSDSWGWRKYGQKPIKGSPYPRSYYRCSSSK
GCSARKQVERSLSDPDVFVVTYTAEHNHAEPTRRNALAGTTRKKFPAPENPSFDVVLSPNNSTSVASIEEEQPM
EGVAEGEVLMNMPFEIFSDDLFTGLEDLLFG

>LcWRKY55

MDPTDSHWTSTDPNLADPSHARPPPSSEPSSAAAAGAKYKLMSPAKLPISRSPCITIPPGLSPTSFLESPVLLTNL
KVEPSPTTGSLTKLPMAHDSSSSAIYSMTTMAFSNTNASDEGKSSYFEFKPYVGPNMVPADLSHRKGEQSKEV
QVQGQPLPFTAPPMTKSEINVISNDLSRSTQMDTVASGASVPEVDGEDFNHNMNTSTRVQPPQSDPKGSGIS
VASDRLSDDGYNWRKYGQKHVKGSEFPRSYYKCTHPSCEVKKLFERSHDGQITDIIYKGTHDHPKPQPSRRYTA
SASVNVQEDGSDKPSHLTGQDDRSCGIYAQTVHTIEPNGTSEPSLAANDSIAEGAGTTLPCKNHDEVDDDDIFS
KRRKMELGGFDVCPMVKPIREPRVVVQTLSEVDILDDGYRWRKYGQKVVRGNPNPRSYYKCTNVGCPVRKH
VERASHDPKAVITTYEGKHNHDVPTAKTSSHDVAGPSTIAPSRYRLEESDTISLDLGVGIGTGGENRSNEYRQAL
HPQLVENQTPNGNFNFEVVQENSAPTYFGVLNRGINQHGSRESLSESHPMEIAPLNHSSHPYPPSIGRILTGP

>LcWRKY56

MGRTDDNVAIIGDWVPPSPSPRTFFSAMLGEDVGSRPAMDTTISDKTEELFLRPREHMVSENALARGGIPGIH
SSDRSMDLGSFSEQKFRGGLVERIAARAGFNAPRLNTESIRSTSTDHSLNSDVKSPYLTIPPGLSPTTLLDSPVFLS
NSLAQQSPTTGKFPFLPNVSNRSLTMMSEANNKGSDNPFNDNSTSFAFRPGVESGSSFFLGAASKAASATILPQ
SYPRIEVPAPRSENSFQSHLVEPSLSLPQNRISHHPQVGLSTSCAERDDGGKTVSDDQRPFDSLCGGGEHSSPLD
EQPDEGEQRGSGDSMAGGGCGAPSEDGYNWRKYGQKQVKGSEYPRSYYKCTHPNCQVKKKVERSHEGHIT
EIIYKGAHNHPKPSPNRRAAIGPSDSQLNMQLEIPAQAGQQSAEGPLWEDSQKGIPTGAPDWMHDNLEVTSS
ASLGPEYGNQPNPLQAQNGSHIETVEAVDASSTFSNDEDEDDRGTHGSITLGYEGEGDESESKKRKLDAYVTE
MSGATRAIREPRVVVQTTSEVDILDDGYRWRKYGQKVVKGNPNPRSYYKCTNPGCTVRKHVERASHDLKSVIT
TYEGKHNHDVPAARNSSHISSGTSSPVTVQNSTAAIQSHVHRPGPPQPQNTIPRFERPPFGIAGRQQMGTAHA

FSFGMNHQPGLGNLTMAAVGQAKLPVLPMHPYLAQAHHVNEMGFLLPKGEPNVEPTSDLGLNFSNGSTVY
QQIMSRLPLGPEM

>LcWRKY57

MAVDLMSFPKMDDQMAIQEAASQGLKSMEHLIRLLSHKQPSSHVDCSDLTDATVSKFKKVISLLNRTGHARFR
RGPVSNSTSSSSSGSVPQNQATNLTPTPFTSPATVSAPPFTAPATVAQPQAKVAAAANFLQSQPQSMTLDFTRP
NILNSNPKGTDLEFSKETFSVSSSSSFMSSAITGDGSVSNGKLGTSIFLAPAPTASGGKPPLSAAPYKKRCHEHDH
SEDLSGKFSGSTSASVKCHCSKRRKNRMKKTIRVPAISSKIADIPPDEYS**WRKYGQK**PIKGSPYPRGYYK**C**STMR
G**C**PARKHVERDPNDPAMLIVTYEGE**H**R**H**TQSSLPESMAAGVSLVFEST

>LcWRKY58

MDIFLDLNVDPTSSYANPAMDEGLDSSKREFEAGEIYWDKEKLSLSLANKGSDSNSTLEEELDRKIQENGKLSQ
MLRVMYEKYMNLQKQVMYLLSQQKQNSEMEAVSRKRKAEGEEDYENLEGICSSSRDEDFNRWLKRPRLNGN
SKVSKVFVQKDASDPSLVVKDGYQ**WRKYGQK**VTRDNPSPRAYFK**C**SSAPN**C**PVKKKVQRSLEDPTILVATYEGE
HS**H**ASHFQTELSLRSINGGKGSAVPVLATIKPSCATVTLDLIHEDGLFKSPKDYASSESVVSTEAAVWQEFLVQQ
MASSLKKDPEFAGIVAGAISGKVLANQTNRE

>LcWRKY59

MLLLHNNMAVELMVGFGDAATNFTPKMEENAAVSAVQEAASAGIQSVENFLRLMSHTPNQHHSEDHSSTST
APNATAGYEAVADSVVNKFKKVISLLDRNRTGHARFRRAPVVTTTSPPPPPPPPKVKTQHQDPSSSSPISTPQIQ
VKKQESVSAFKVYCPTPSSVVRLPPLPHNNPHQPSSNPPNTFQAQQNSSSVVLKNGSADRKDSTTTINFAASPP
ISAANSYISSLTGDTESLQPSLSSGFQFTHMSQVSSAGKPPLSSSSLKRKCNSMDDSAMKCGSSSGRCHCSKKRK
NRIKRVIRVPAVSSKLADIPPDDYS**WRKYGQK**PIKGSPHPRGYYK**C**SSLRG**C**PARKHVERALDDPTMLIVTYEND
HN**H**AHSTETPAALVLESS

>LcWRKY60

MGDDEEQLPPSSAPPPPSKSKALPLRPTINLPPRTSMESLFSGGPGLGSGFSPGPMTLVSSFFSDSDDCKSFSQL
LAGAMASPVAVPPRASELKGPAGLLDSPGLFSPSQGPFGMTHQQALAQVTMQAAQTYSHKQIQGESFSSLVP
PSASSSELLTTFPGEKTKDQPVPPSLLNSAVASKESSDISQSDQRLQSSSCNVDRPADDGYN**WRKYGQK**QVKGS
EFPRSYYK**C**THPN**C**PVKKKVERSLEGQVIEIIYKGE**H**N**H**KRPQPNKRAKDVGNSNGCSSIHGNPKLSSQVQSGYL
NKLNDQQTAVSSIAKKDQESSHVTHEQLSSTSDGEGGSEIETGVSRKDEDEPDAKRRNTEVRVSEPASSHRTLTE
SRIIVQTTSEVDLLDDGYR**WRKYGQK**VVKGNPYPRSYYK**C**TTPG**C**NVRKHVERASTDQKAVITTYEGK**H**N**H**DV
PAGKISSHSTVNSNVPQLKSQNIATDNNTDPGNSRQQPVGLLRLKKEQIT

>LcWRKY61

MASQRKLHKDEQDPAMEDAISLILRGCSLARELEFNVLNSANNVHFPQPQMVARSCDQILSVFSAAKDRLSGC
ERPPLTAVLREVGLEDWLRSSQAMDQLAQMQMRSPPPPPPSAVEDSGKLVGMGFGSSSSSSTKPRRRKDDM
EKRTVRVAAPRIGNTELPPDDGFT**WRKYGQK**EILGSRFPRGYFR**C**THQKLYH**C**PAKKHVQRLDDDPHTFEVTY
RGD**H**T**C**HMSATAPSAPPPPPPDVAGQHMAQFRPPSAGWLSMEFVGPASGSGSGSSSGGPAAVRYGKDVAD
QFPVLDMADAMFNSGTGGGNSMDSIFASYVAEEHHHHKWEKDDKKN

>LcWRKY62

MELADNSRRRFPAEARRRITSPLVRGRESTVRLQILLQSETATDHDRRALAAEILSCFTEAISVLHSSESAGDEFGC
PDHSLCSDLDSGDSRRSPAGKDLGRGNKRRRLANTKIVSSAMIEDGYA**WRKYGQK**AILNTTHPRSYYR**C**THKY
DQG**C**GATKHVQRMEGNSEMYKITYTSN**H**T**C**GPTSPIAAVVIEPPPSASNSYNICFSDDSRNDQLVESTGSFFWS
SKNNNVAKGEETTTTSGVTDEMDLWSELDFGSLQETAATPMTTNQYYFLNGDDADSSHMFWGGF
