# Supplementary material for: Identification of WRKY Family Members and Characterization of the Low-Temperature-Stress-Responsive WRKY Genes in Luffa (Luffa cylindrica L.)
Source: Plants (Basel). 2024 Feb 28;13(5):676. doi: 10.3390/plants13050676 (PMC10935285; doi:10.3390/plants13050676)
Supplement: Supplementary file 1 [file plants-13-00676-s001.zip › Supplementary File S3.pdf]

>LcWRKY1 + Up_Stream_Len 2000

aaacgcacctgtagttatcggagaaggagaagatggaggagcatatcggaagaagcattttgctgcagtgtggccaaatttggtacatagttggcac
tgaactctgttccggttattccaagagcgacccccctcggtttgaaggaccacctctgcctccacgacctcccttatttccatttccattattctgagaaaa
ctgagattgattagttttcgagagataagaatcaactggtttagagtgaatggtgagattcaccgaaggaatagcaccttcgggttgagccaacttactt
tcatttctattttcttgagttaataaaagtgacatcacctcatgtactgattgaggacccaccttagcagaaattacagataccatcgactcgtaatcggat
ccaagaccggccaaaatgaagagaatatggtcttcaacttctacgggttttccaacagctgctagggcattacatatttttgtattttagaaaaatattcttt
gagagacagacatacctcccttttgtatagtctgaagcttggtcttaatcttcatcatttgagcaagatttctggttgtgaatatctgactcaaacaggtcc
agatctcctttgttgaagaacagtgaatcatttggtggagtatttcttctgacatcgttcctactagccatgaggaaattaccttgttttgccgcttccattga
aaataagctggattaggaacggacgcagtagctgatgttgcgacagaagaagactcagtgccagtgcttgtattcgcttgaactgttaatgacttcgg
cggagggtcatcatttaagtgagattcaagcccatagccttccaatgtcgttagaatctggaatttccatagaagaaaattttcgtcggtgagtttaacaa
ctgaaattttgttaccggggctaaaagattggaccgaagccacgcttaaatcttctacaacaccactctcggagctttctggtgtcgtcattcagaacag
cagagaagaaggatgagaatcgagctcagtcgctagacaagaggctctgatatcatttcaaaatcacaaacaataacagaaaatcacaaaattaga
gagaagatgtgcaggagaaaaccctatgcatttatatatgattatgatcaagaatcagccatatataaaggtctggggagaaaaactcctacaaaacc
attcacgatttcttttcttctggaattttatttttgccctaaccaactctacagacgagaaagagaggaagaggatataaagaatgttgggccgaaacata
atgagccaaaatacatgaataatcatgaatacaaaaaatcagaagatgggctttagagggctatcttaacagaaccaaccttcaaagttcaaaaccaa
tcgaaacttagttcaactagtttaacacattatatcttcgacaaaaaaattagagattcaaatcttcatcttcacctactattacactcaagaaaaaacaaaa
aaaaaaaaaaaatcaaagtcagaaaacttattagaaacgactctatataagaatctatcgaatacaatcgtgaaagctcgagaaccaagataggtaat
gtaagagtgattttgtgttttcctaattaccatagatgcgcgtgggtcccacgctccttccatgtccccggttccatgcttaaatatcagcctcccgtacaa
attgagaagttgacgcccatttttctaacttatctcttttttttttcttttttcttttttatttccctctctattcccttcccattttacgcctctctctctctctctgca
actgtatattgacgcgacatagctttcgaaaacctttcaaaaagttcaaaaactacgcgtcctttccatttctcaacgtaccctcaccatttccttctccctc
acaaaccctaattcctcatctctcatccaaatctcccttccgatttccactcgatttctccagatctttcttctccgatt

>LcWRKY2 + Up_Stream_Len 2000

gtatttattattatttttttataatatgtgagatgagagatttgaacctctgacctcttggtagagagtacaagttttacgccagttaaactatgttgtcgttgac
acttatatcagtatttatgacagctatgattttagtaatgattgagtaatagttaatctccaagcaacgtggtatacttctcccatcttgtcaaatgaatattca
ttgaactcagtgaacctaaaaatataaaacaaaatcttgattgcctaccaaatagaccattagcttatcgttgtacttaaaaaaaaaaaaaagtattttact
agctaaactcaagctctaacgtgctcattcttttaaaaaatctttgaaagttgagtttatttattaattactccttcctttactttttcatcttttttttttctaaaaaa
acaacatgctttgaggttcaaatctataacttattaagtttgggaagagaagtaaataaaatgccataattcttcattgagtgaataggcaattaaatagcc
aatgtacagagatctgttagagaaactaacagagaactaactaacaaagtaactgatcctatttacatcttaatattcccccctcaagatggtgctggag
tcaagtccagcagagcttggagagtcggtttgtgtaatggtttggtgaacacgtccgcaagctgtaggtgcgtgcgaataggtagaagtttgacatct
cctttggcgattctgtcacgaacaaaatgataatctatttcgatatgctttgttcgttcgtggaacattggatttgttgcgatgtgtacagctgcaatgttgtc
acaatagactgtggaaggggaagaagagcccacgcgtaactcggttaataattggcgaagccagacgacctcacttgtggtagctgcaagcgctc
tatattctgcctcagcagaggagcgagaaatagttgtctgtttctttgatttccaggagatgagcgattcaccaaggaaaacacaaaggccggtcgtg
gactttcgagaatcaacacataaagcccagtcggaatcggtgaaggctcgtagttgaaaggaggaagaggcatggagaaaaactccctggccag
atgttcctttcaaataccgaaggagggagtgtgctgcagacaagtgaggtttgcggggctgagccataaactggctgagtgcaaataaggattgga
gtacaattcaagaattgagcggctagatgaggagtttgttgagttcgtagcagccataggtgctgtaggatcagaggtggtgacggaatcaaacatg
gcgcgatgaagcaggaagagacgcaaaacgcgggaaggaaaattcactctctgaagcaattaaaatctgataccatattaagtttgggaagagagt
aaataaaatgccttaattcttcattgagtgaataggcaattaaatagtcaatgtacagagatctgttagagaaactaacagagaactaactaacaaagta
actgatcatatttacatcttaatataactatctataattaatgatatatttttttttcattctaaaaaaaaaactaaactagtctattcacatttttttaatttttaaaatct
cagacaaatttcatattaaaagaaagcatataatttcgtaaaagaatatagagtacaaaagagctaaaagaaaaagaaaagaaaatgttaaattcgag
aaacaatttccacctcgaactttaaaaataaaaataaaaatccaatatattaaacacgtagaaaataaaaccatatttcttacatttttctttcgaaggtttttc
gtcattatcgtgacgtcattatcgcgaaaataagaaaagggattatattcataattccttcccacaatttccaaaacgatttcctatataaagggagttaatt
cgtgcctattcattcagtccaagaattttatttcctggtttaccaagtcctctcaacttgaaattcttccttcttctagac

>LcWRKY3 + Up_Stream_Len 2000

Tttgatatcttacccctgaggtttgcggaaatggtttttttagtccctgccgttaacaagggctgactggattttctgagacatgcttttgtttttttgcccaaa
aaaagagaatttgcgatgggtggcaaataagaattatataattgcaaatattgcacattgtaaaaaaaaaaattgcaaatatagcaaaatcaatcaaagta
tattatatgctattaatttggtccaatttgccatctatgcaactgcctaaaaataaaactacttctttattagtccgaccaacaaacgcaacgttttaatacca
tatcaatcttaacatcttttacaaatacaaggaagagtgattatataaaaatatttcatgtggaacaagagtgagactttttcacaaagggctaaaagccc
aagccattcccaaccaaagaaagaacaagacggtggcgatgagtcttgatgatggcgaaagggacgattgatggtcagaatggggaaaaataaat
tgaaggattagggaggggagtaaaggaaaagagtattttaaaaaattacttaaaaaaaaaaataatgccatgtcagcttaagaatattttttttaaaaaaaa
atcagttagctttctgatagctacaaacggcatggatcaaagaaccattatttttccaaacctcaaccgcgttaaaatgtcaagtttgaaaattcaagtcct
tcataatcatttggtttttggtttttggttttttgaaaattaagtctataaacactagttccacctacaaatttctttgttttcctaatacattctttctatgtcttaaaa
aaccaagccaaattttaaaaactaaaaaagtagttttcaaaaacttgtttttgtttttggaatttggctaggaattcaaatgtgcccttgataaagatgaaaa
ccatggtaggaaaattgatagaaaagaagcataattttcaaaaactaaaaacttatcaaacgggttctcaggaacccaattacaaccaaacacaaatc

tcaaagaccaaaagtgtaatttttcagaagaaaaataaatgatattaaattataattaaaagataataaaaaaaaaaaaagacagatatttaatctaaac
acgattatattatatctacaatctaacactctccacatattgtccaatatatatatatatatatatatataacactccccctcaaggtgggttgtatatagaa
tacaagtcaagcttggagttgatttcatcaaagctctttggtaatgttatggtgagaaatgtaagcaatttaatgtctagatggaacatagtttagtcccact
atgctattgttgaccttcttaaacatgaaataacgatctatctctatgtgtcatcttgtcatgatacacaaaattctttgctatatattgattgttgtttgactatc
acaaaacattttgaatgagctctaagtttttattctcccatccttttatgacatgtttggaagtaattcttgaattaccaaaatcactttgtatgatgtccaacta
aaaattccaaggaccaattggtaatcttacttctttaaaagtcccctaaaaaaaaacatcacagcacacaagtccattattatttactcgtaggtttttaggt
taaaagtcttttccatgtttaaaaactaggatttctttttccacaaaaaccctagacttatgattccccgtcgtaatccaaaggcatttaataatgttagagaa
atccaaatccatgacatgcacgttcgttcttcttcaacctcgtggctttgaattccttggcctacgattccaattcccagcttgagaaatccaaatccaagt
ccaactcaacaccaaaatttcccatcaaaccacttgttaattataatgccccttcccttttttaccatttcttaatgatattcaatgatcataataataatccata
taacctaaactttcatttctttcttcatttctttactttctcttc

>LcWRKY4 + Up_Stream_Len 2000

aacatcaccgacataatcagatcgatggtaaaacctgaaagttcagggactaaatttgcccaacgagtaaaagattcgtttgagttgatgtgagagtg
ggatgtaaggataaaagaaatgatttgtcaaacatgcaatgaaaaaggagcacatgtggagaagaagtggagttcacgaggagagaaagcgcgt
ggagtaaacagagctccctaatccctataaacctacggaccaatgaaaggaaagagatgggaatcgtacagtcaaacttgtaacgcctttgccacct
tccctcctcttcctttgtccccatttgacattttctaaaagtcaatcaaaaaacaaaaaaacaaaaaacaaaacaaaaatcccctctctctctctttctccat
ttttttctttctttctctctctacatgcatcttctttagatttatggatattgttttcttcatatttgagtagtttgtatactctgttttgatgacgatataggacgagg
ccgagcagaaagaggcgtaatttcaggattaaagctttacgactttcaaatcactgtcctacgaactaaatttttttacaactatttattttttttaaaaaaaaa
atgaatgacaattcctttccctctcttcttttttgcttaagattttttttcttgtaaattgaggtaaattatgttggaattgtttcttcttgttttgtcttaaattcaaaa
aatatgtgaaataaaattaagggtttaaagtgtttttttctctcatttatctccttgaaattagagaatttcctatatttttttcgcttcctagtgatggtttttttttttc
ttcttctttttaagtagtataattcatttttttaagaatataattttacaagggttaggaaaggggcttgaatttataatctagggagtattgatgtctaaccactc
aaattatgttcgaaactaataatcattagttaatttgagcataactttatactggaatatagatgtttgaatgttaacccacattgaactaaacaataataata
ataaatttgtaataagtaagcttatttattaaaaataaaataagtatcaaaccgattacgaagcaattatttttaggaaaaaaaaaaacaaactcattctttttt
gttgaaagatttgaatcacaaatcttgattattagtacaagttttatgccagttgaactatactcatattcaactcattcaatatgcccaaatttctttcataggt
aattttatagtttaaaaggtgttgttataacttgttttcgtgtttgaaacttagataacattttgaaaaacaattttcgagaaataacgaacaaagacactctaa
ctaacgaaaaacaaaatatctattcaataagccattaattttctatcattagtggtagaatataatcattatttttacaaaagtttcaactctaactagtataga
accaagatagtaggagtctcggaaaaattactcaaacataaatggaaataaattaaatacgacgatataatacgattaatcaatttaagatttagatatttt
cacaacctaatcacttaatttacttaatttccaaaattttccaaaaaatcatgccaaattgctcaacacccaatatcaaagataaaaacaaaatgtcaaga
gataataccaaatatatatcaattttttaaaaaaaaaaaaatagtgaaattattttttttcattttacataaattgaaaatattatgattttctgtgtttataaatacata
attaagaattagtgaatttcaaaagtcagtgctctttctctttcatttctttctctctctctctaaacacatcaaaaagtcagcgccgtcgtttctgccttctc
ttctttccctttgtccccctttttaccaaatacctaaaatttttcgtcttttttttgtttcggtttttttttttttttttttttttgtttttttttcatttcgatttgtgtataatccc
accc

>LcWRKY5 + Up_Stream_Len 2000

gggccttaaatgtgaggtttttcctttttttttagttcaacatgtaaatgttgagggattcaaacctatgttcttttggttggggtatatatctcaatctgctaaat
catacttaggatcatacttagattgataggtatatacacgtttttttttttttttaattcggtaccttattttaacattttttttaaattatatgtgagcaattatttata
gttcaattatatggtactagttgagttggctgaaatttaaacaaggtatgaaaccatttaagagaataaatattgttataggtaattttggaccacacagat
gcacaaggagctgacaaggacaatcgggcagaggtaggaccagaaaaccgacccagagaaagacctgatcaaagggtcgggccatgtatggt
cggcctcggcaagaggccgaggccgaccatccggcccgcgcaccccccctttcggtcccaaatgccccgaatcgccccggttttgcccggttcgtc
ctgaaacacctccgaattcctaaaaaccctaggaggacgagcaggtatttaaacccctcttcatcactgaagaagggttccgaaactctattctcgact
tcttcctttactccctatcttctcctcggttttctgacttaagcatcggaggcggtgtggcctacaccacaccggtgtgcagcggttttggctggtcttgca
ggtcacgtcttctccagtttctacaaattcactgttggtgtcacgtgaaggtcaggtgagtcctctatccgaattttggtatcaacaaatatcaaattttctat
cgacttcttcaaaatttagaagaggtattagaaattattctctcttagtattaaaattcctccaattaagggtccgtttggaatacattttaatttaaacgtttttc
aagaatgcatttttttaaaaaacattttgtttaaaaaattgtttagaattcaaacactcatatatttggttacatatttccttgtaagtgtttttatgcacaatttattgt
atacttaaaaagtctttttaaaaaagttgatttcaagtgtttctctcaaaatgatttatctatgaatctcgttttctaacaagtcatttttagtggttgtcaaatact
agaaatttttttgaaaatgatttttttttttcaaaattaaacacttgaaaagttaaaccaaacccaccctaaatttctattgtctcactaaatacatatggtataatt
gaaaatttcttaactaaattaaaaactttgtaaacaataaagactaaattgaaaagatatctaaacattagagattaaaacatatatttaaccttttttttaact
aataatgactctaatgtattactacatgtttgggagtgatttcaaaattgttaaaatcatttttgtcaggttcgaaatcaccccgaaacatacttttaattattc
aaaattatcaatttcatgtttggttttacacttctaaacgtgctttttacaccatcaaaattgattttgattttgattttttttttttttttgacaatatgggggtgggg
gatttgaaccatagatctcgtgattatcagtacaactttatgccagttgaactatacttttgttcgcaattgattttgaattattaaagacacgttttggactga
ttttgaatatgcaaaagtgattttaaccatttgaaaatcacactcccaacatatgtttaatatataaacctgccctctctctctctcttactttctctctctaactc
cattaaatgagtgatgagaaacaaaagatagcaacttgggagggtgattttgtcatctcctctctctctctcttcataacagatgggctctgtgtcttctcc
atcctagtcctttctttaatagcaaacaacccctctgtctttatttctctccttcaaatgctcaagaagaagaagaagaagagaaaaccaaaagaaagaa
gcaaatttagccatagccatagcc

>LcWRKY6 + Up_Stream_Len 2000

Gcaaagaaaaatgggtttctaaaaagaaattaactaaataaaaaagaaaagaaaaagaaaaatagaaagtaaagtgaaaaagaaagtgagagag
ggaagaaattactcccaatattttcaaaaagaaatatatttatttattgctctagatgcacgtgcgaggtttttatgagtaactttcagccgttagctttgcac
gtgtgaagagagataaaaacgtgttcacacttcgcatctcagccggtggccggtcaagtaattcatatttacttttgacccaaaattttctatttattttaaa
ttataacctttaggttactttattttaaaaagattgattcgattcatatataatttggttgaattaatcctaaaaatgtctctattattactaattgtaattaattttttt
aggtgaattgtttttagacatggttaaaaataaaagaagataagctcaatatgtataaaaggtaaaataatatatagagattactttttcaaaaaaggtat
atagaagttaaattaatacctttgatgatttgacctaatttttataataatgctttagagggttgctttgaaattattgacataagtcggtcagtataaacatag
ctcaattattaagatatttatcaatttttttggaattagaagttcaaattctcatctactaaaataattttgtttaggaaaaaaagaaatataagaaaaaaaaac
ttactatagttcaacttattaaatcaatagtcaaagcgaacttagttcaacggtaattagcatatacccttgaccatgaggtcagaggttttaatccccaaa
attaaaaatttagtgatttttttagggaataaatttagtgattgaacttttaagattgtatctaatatctaatctatcgttaaacttgaaaaagaaatatttaata
aattcttaaactttttgtgtataatagattattaaactttaaaaatgtcaaatacacaattttgtgtctggtaagcttctaaattttgtgtctaataaatcttcaacc
ttttaattgtgtgtctgaaatgtccataaattttaaaaattttatatatatatatatatatattgacagtgtataaggtagggatttagacttttgacctccaatcg
aaaaaaacattctttattttaattgaactatgctcttgttaaagaaaaaaatatttaatatattagaaacttatgacatacaaaattgaaaattttaaaaattatt
aaatattttaagaccataaatttatggttaaattacaagtttagtccatgaactttcaggattgtgtcaatttggtacctaaactttaaaaagtgtcaaataagt
cctcgaaactttcatctttgtttcaatagatccctgaactttaaaaaatatctaataggtccttaaaccttcacttttgtgtccaatagatcatttaacattcaat
tttgtgtctaataaatccatgacatttttgacactttttaaaatttacaggcttaatagatacaaaattagaagtttagaattctattaggtataaaattcaatttt
atataaaatagatggattatttttaaaaatcttgaatgtgtcaggaatccattagacacaaaattgaaacttagtccattagatactttttaaaattcatgaat
ctaattgatataatgtcgagggactaaacttgtaataaatactaaatttattaaacatgaaatttaaaaaattattaaagaggatttgaagagaaaaaaggt
ggcatagaaactttcaccgactctgtcatttccgtacgaaccgatgacctacaaattccagtcagttcccctaccttccattctctcccattttcttctctctc
ttctttctctctcctctttttttcttcaccttattctctctctcacttcaagtttttttagaaagaaaaaaaaaaaaaacaaaatccaacagacagagagccgcag
cagcagcagcc

>LcWRKY7 - Up_Stream_Len 2000

aatttatccaatcttattctcatcctttatttttcgctcatccataataagaaaaaaaaaatgaaacatattatatgctctcccttttaagaagccacaaatggaa
atggagtacaagtggattgattgagattaattctcaatcaaaattgatgttgaattaatttagaaattactaaaagaaaactaaatactcaaccaaacatca
taaaagtttgaccacattataagtcaaccccaacttgataaaatattaattaaaataaatgtcaacctttaaaaatacatgaaattgaatttgtcgttgtttatt
aaccatttggtcataatttctaactttattgcaaatagaaaaatatttcatgccctaagattgtagcatatattatttaccctatgacaataaatgaatcctcaa
ctctaggattccattacttctcaaaaaaaaaaaaaaaaaaaaaactctaggattccattacctttccctaattactttatttactaaaaaaaaaaaaaaaaactt
atagacttgaaaaagttccattgaaataatattgagggcttaaattttcgtttgaattacaagtttagtccatggacttcaaatgtgtagtcttcactaacttta
aaaaatgtttaatagatctttgaaatattaattttatatctaagaggtctttgaataactttaaaaatgcctaaaaaaattgagaccgcataatattaaaagttt
agagatttattcaatataaatttacaaattcaaggatctattcgacacttttttttagagagttctatcagacagttttaaaagttcaatgacctataaacacaa
tattaaaaataaaactaaaacgattcaattttctgtaataataagtccatgaattgttaaaatgtcaaatataatataccattagacacaaaattgaaagttc
agagacctgttagacaatttttagatttttttaaagtttaaaaatctactacaactaaagttcttattattattattattttgagttcaacaatgattgggggtg
ggaggatttaaacctctaacctcttgatcagtgatatatgtcaattaccactaagttagactcactttgactactacaactaaagttagagacctaaacttg
taattttttttaatctccaaattgtcacatgctacccaaaaggctttgaattcttcccatgaaaaaatgtgaatacaataaataaacccaactatagaaaaca
ccaatccctccaaaaaaaaaaaaaaaaaaaaaaaaaacttgcaaactcaatgaaattcaagacttccaactataaaaaactaaacctaaactctcaagt
ctctcaaaacccctcgatcgtcccactgtggctctcactcctttaaattatgatttttttgtttctttctcctatattcaatggtataaaaccgtcagtcacattac
accagtctcctcaaataatttctcctttctcacaccgctagaggccccactctatcaaatctcacaaaaaaaaaaccattaattacaaatcaaattcaaca
atgaagcacacaaggtgttgcttgactttgaatccatcctcccaaagcccaaaaaaaagaaaagaagtttcaaatttcaactccctcaaccgacatgg
ccccctctctcttccttcctttcaccaatcagaaatctcaaatatgccaaaaagcgcacaccaaatcagaactccctccatccatgtattttcctcttctaat
ttccccaatttcttctgtcaaattgccagcctttgcctttattatcctatttctctctctttccatggactttcctctctttttctcccaacccctcctctatatatcc
ccttcctttccctctcttccctctcggaagcgcttttcctctctataatcacttctgggtctccctcaatccgaggaaaaatcaaaccttttcttgttttctctctt
gggtttttggtcgtttcttcttagcct

>LcWRKY8 + Up_Stream_Len 2000

Tcctattactttatttttaaaattgtctagcattttgttatataattttaaaaatttgaaggaaaagaaaagagtgagattaaattaatggagttgatttgaagt
tgtattatttattttggtatatggcttaaaatgattaatttaataaataatcgatgtatttagacaaatacatatgtatatatgatttgactaattcacgagattttg
gaacaaataattgaatataacttttaagagacaatgtaagggtccgattaattggaccatatttaaatttcgtgtttatttagataaattaagcattttcacaat
tattcgttgtagattagtctaatttgatttttttttattaatatgataaattattataccaaaaaaacgtgataaatctttcaaaatttgagaaggaaattaaagca
ttcaatctgataaaaatatcaaaatcaactaataattttaccaaatatagtgaagtggttagatggtcaaggatattgatgaggcggaggaaatgtcagc
aagaacatccacgtgtcattcatcttcttcctccagctagccgttaagggaattaacgatctctgtgttcaaaaattggtgtacgcataatgttttaaaatta
cgagtcctgttgtttaaggtttgatttatttatttatttattattataatttttttttatatccgtgagtatccgaaccagcttacgcgcacctcgactaatcttacag
gacaaccgctcgaccctactatatttggttgtcaaggaaacttgtagaatattaaatattctaggtaggtggtcactatggattgaacccattccctctaat
taaggtttgatttactaatttataataatatcaagcaaaagaaataaaacaatcatttaaaagaagtaaagtccattatgtaatcataattattgacttttctat
atagatgctatataatttttctatatagttcggtaaacaagcaaatctacatagcttttatccttcatcaatgctaaaaaaacaatcttttaactattaaatttcta
tggccatttagatcaaaattttcatttagcttaatagtacgtatgtgtattaaattggtgatataaagaatcagaaaaaaaaatactcagattctacgtaata

acaacgtataaaaaagtataaaaatgtgatctttgattaagaaaacttgttcaaagaggtgaaatagtatagagattgataattttatatcattaaataatat
aaatgaaagaaacaaaaattataattaaccatgcatgaaagactttaggggaagagaattaccataatgtagcagttagtcggtgatattgaataatatt
ttattgagtggtgtataatcaaacatacttccttaggaagcacgaaaatcgaaaaaaaagggtctaaattttgactgaaggttagtttatgtagagaaatg
gtgtggagtaagggactagttgaatttcaaatttcgaaattgaacttgtatttatttaaattaataggagagagaggaattgcattggatttgtaaatattatt
ttggtgtgactgtgagcgactatagtatatagtataaggaaagaaaatgagtgaaggatggtaaattggtaatttaggcggaagcgtaaaagaatgga
ataaggaggaatcaggtgtggaggagagtattatattatttagtgaaagccacgtggaaactgatgtaggggatgattgggtaaaaaaatccgcgta
agagagaaagtggagggccacctcagcggtggtcctggtgcatttaatgttaaacaccgtccctcagtcgacatattccgtacgcagaacaaccga
cagctgccggtcttccttggccttcttgggtttttttccttgaaatttccaaaaacaaaaacccctctttcacagttcacactctcaaacactcggcgccca
ctctctctctttctatcttctatctgtttttatatatgtatatat

>LcWRKY9 - Up_Stream_Len 2000

atggtgatggatcaaattgaaagatgttgccaataagagtacaacttaattggcataaagcagccaaagcgaacttagctcagcggtaattggcatgt
gccctcgaccatgaggtcaaaggttcgaatccccccacccccaaatgttgatgtactcaaaaaaaaaaaaaaaaaaaaaaaaaaaaaaaaaaaatt
ggcataaagcactattgaccaagaggtaaaaggttcaaattttccaccccaaatgttgtaaaaaaatgttaaggccccatttgataatcatttggtttttta
ttttgggtttttgaaaattatgcttgatttctcctaatttccttaatatggttttcatctttgtcaaagacactttagatttctagtcaaatttcaaaaacaaaaaca
agtttttgaaaactattttttttttagttttcaaaaaaatgacttggtttttttaaaacatgggatgaaggtagataacaaaacaaagaaatttggaggtagaagtc
aaatttagatttctagtcaaatttcaaaaacaaaaacaagtttttgaaaactattttttttttagttttcaaaaaaatgacttggtttttttaaaacatgggatgaagg
tagataacaaaacaaagaaatttggaggtagaagtagtgtttataagcttaattttaaaaaaccaaaaacaaaaaaccaaacggttatcaaacggggc
ctaaattatcaaaactttccctgaactatgggattggtttcaattaaactcttaaactttcaaaagtttcattttagcctttgaaggttttttttccattcaattcata
gacgaaaaaacgatgtgtttgcactttgcatgtgtattgttgtttatatgaatagatttgaacacatacgtggaaaaacaataaaaacacctgcaaacac
gtcattgtttgtcattaagtttaacaaaaaatgtcttcaagtgccgttttgaaacaaacttcaaacttcaagggttaaaataaaacatttgaaagttcaagag
tataattgaaaaccaatcttatagtttaggagtattttttataatttaataagaaaagaaaattaaaagacgtttttcaccattttctaaaaaaaaattagatttgtt
ttttcatttttttcaaatgttaggataaggatttatagttttagagagagagagagagagagagagaaagagaagaattttgaagtgggatgatgagtgtgtgt
gtgcatggtgagtgtgtgtttggcaagtccaaaatgcggctctaaaccaacaaaaaaacaaatcaaacaaaaattcaaacacaactaatcagaaaaa
gacatctaaaattaaattcaaacagtacacctcaattttcaaaaccttctccaattcaattcaattcaatttctttctttcttttttttttaatcaattcaattttaaaa
agcgatttcttttttttttcacaaaaagacactaatacgtgtatttctcatctccctcttctctcaagcaaagcaactggcaaaccccactctgtgggtcccac
tccaaattttttttttaataatttttattttctctcaaggaatttccaaaccatttaaataagcaaggtgggaaaaagagaaaaaaaaaaaaaaaaaaaaaaa
gtaggaaagatggaaactttgtagtttttggctctggcttcttcagggggttgggttctcttctcaagctagacaaaagaaaaaagcaaaaaaaaaaaa
aagtcttctttttcacattcagaccaaaatcatggaagttttagtttagggtttcagaaacttacacgctctgtttttgttgtttttttcttcttcatacatacttctg
tccatttctctctgtttttttgtttctttttcccaacttcttcaccttctcatgattccctcttggattttacctctttttgttcttttgggtattttctaaaccatttttctct
tctcaaaattctgtttttctgtgggtttttttgcaa

>LcWRKY10 + Up_Stream_Len 2000

acaatatgacttttaatttttttcaatgaatgggaggagaggattcaaatttccaacctcaaaagtatgaaataacacattaatcattatgtctaaaaaaaaa
aaacacattaatcattaatggtgcgtttggtttaacttttcaaatgtttaattaaaaaaaaaaaaaatcattttgagaaattttctagtgtttgacaaccactaaaa
atgacttgttagaaaatgaaattgataaataaaccattttgagagaaacacttgaaaccaagtttttaaaaaaagagtctttaagtatacaataacttgtgtat
aaaaacacctatgagaggaaatgcaaccaaacatatgagtgtttaaattctaaacaatttttatacataatgttttttaaaaaatgcattcttgaaaaacattt
aaattaaaatgtattccaaacggaccctaagttatgttcatgtgttttactttttagttccaacaatacgtggagtggaaggatatgaacctttaattagcga
gtcatgctcatgtcgacgataacaacactgtaaaatatgtgttttagcatgctaatatcattacattctaaaattacctctctccctctctatatatagaagta
ataaggctacctctaaaagctaaagatgggtattagctattaagtgattatttttttggatttgaatatctaaccttttggtctaggttaaaaattcaacaacat
gtgggggtaaaaactcgaacatctgatcttttgataaaagttaaatgtttttttttagttcaatagcacgtgtggggttgaggatttgtaactctaacattttgg
tccataaatatattttaattaattgaccatattcaaattggcaagtgaaaagttactaagtgattgaatgacaatgaagattaaaatagtgaattttagaagg
tgagaacactaaaatcctatttgataaccattaggtcccgtttgataaccatttggtttttgaaaattaagccttgttttctcccaatttccttagaatgatttgc
atctttgtcaatgacacatttgaattactagccaaattctaaaaacaaaaataagtttttgaaaactagtattttttagttttcaaaatttgacttgtttttgaaaa
catagaagaatgtagataacaaaaccaagaaacttaaaaggtggaagtagtgtttttaggcttaattttcaaaaaccaaaaaccaaaaaccaaatacc
aaatggttatcaaacgaggccttattttttggtttttggtttttttgaaaattctttattttgttatctaccttcttttctgattttcaaagctcaagccaaattttgaaa
ggtaagaaaagaagttctcaaaacttagtttttatttttgaaatttggctagaaattcgaacgtgagtctttcacgaagatgaaaatcaaagagaaacaaa
gcacaattttctaaaactaaaaagcaaaaaatcaaaaggacctaaaatagtgatttcaaatattgtttggacaagaatctaaaaacgtagatgaagggt
gaaataaaaaaaaattgatgatatagtgggtcccaccgagtcgcacgtggtgcagtttgggattgtattttgattgatatctatgaatctttttaatgcaaag
agaaaaaaaggaaggaaaggaaggaaaaagaagaaaaagaagaagagaagaagaagatcttaggaagctattttaagaaaggaaaatgcacct
aacagaatttacgcttctttctatcttccttccaatttttttattttactttttttgtaatttttctgtttcgattttgaagtatttcatcgtttaatctcccttccgatgtc
atccccaatttctccatcttccagttggaggtacacacaactccatgatcatcactgcaactcaagctcaattcaattccccaccccaatttcaaaaatgc
aattttagattatgttaagtgttgccactgtgtaatga

>LcWRKY11 + Up_Stream_Len 2000

Tttatccatcgacaataattgctggtcaacggccttgatcccgaggtcggccagcacaatatacggcaaaactttgttgccctggctgaccgggtcg

ggtcgggtcatttccctcaagttccacaaggatttttatattttaatatccatctaatatatatacacgatcattcaagttccaccgttaacttttatttttattttt
cttagaatttttgtagaaactcaacgtacattcatttttttcctcaaaagaaaacactttcaatggttcaaagacctacctttctcataaaaaatacaccaaa
ggcttgttcggtaggcaattcagattctattttttgttttcgaattcattgaattcttgtgaataaattgtggaaattctaaaaacatttttaaaaatgattttttat
atcaccaaattttgaatttgaaaattttaaatgcaagattaagtttaaaaaaataaaaactttaataaatatagtatttcacattataaactcaattcatttgtgt
gaaattaaaatttgtataatataatgtattataaattatatgatattgtaaaataataattataaaaaatttataacatataacatgtttataaattaattaataata
gctatatagtttatatattcaacctagcatttagttgataactataaatatatattggtttatggttataaatatattatcaaatacatcttcaaccatggtataaa
ttgggatccaatttccgaatttactgtcaaacacatctaagaacatgaaatacaacgaatcccttgtgtttcaaatgtctgtttttagatcgtcaaccaaata
taccctaaatttgtagaactagtatcttttatgtcaaaatattattatgaaaaatcaaaccttgtagatacctcaactagttaatatatatattattaacaaaaa
aaaaagttagagatttgaatcttccaccccatatatattgaattaaaaagaatatattatcattaaaagatgatttttctttttttagttcaacaagatatgagtg
aagatcccttagatattttgatcgagaatatagtatcccgaatagttgaattgtttttaggttagttatgaaaataggtgatttaaatctggaaagtagaaga
agaaacctcagtgttgtgggttgggtatagggtttgatttgagaagaagataggcattaatattgcatcttaagaaagtagagatatatatatatatatata
tatatatattggatatgacgttaaaaaaaggtcaaaggcatagaccaaaattatgagaagggcatttggtaagactcaaatgttttgaccttagcttagct
tacacacaaattaattagaaggagagagagactacaataatataacataacataacataacataacataacataacaacaagccaattagggatatga
gagaaaactaaggtcacccattgtagcattgcacacgccactgcctaaaactttgacctttcaaccaatccccatgtgacatttattatttacaattattttt
ttatttatttatattctaccctcttcattcaaaaacttgaaaccccttttaattatcaacatgttatatatatatatatatatatatatatatcaaacacttgcaac
tctttataattctccatccctccggccattgcgccataacccactactctcttcgaagatttctacaaaatgaagtgaattggaacagttttataatgtaaga
tgcagagtttgtgaaaagggttgtgtttggatttgtgtttggatatgaggatgacaagtaattaagagttgaatctgagtcttctttttttaaatatgtaatttt
gttcttttcttagaaacaaaggggagaggaagagagagtggtcaaacactgctgctctctagctcagatttctattataattaaggataagcaagagct
caagaagaaaacaagaggcttttc

>LcWRKY12 - Up_Stream_Len 2000

Atatattttttatttaaattttattaattataaatttatagacccttgtacaaatgctatttttctaatatttttctcaaaattttattgctataaaaatttatcattatgt
ttttccttttttttttttttggtaaaatcataattcatttttttagtacatcaatatttgatcgaaggtacatatcaattatcgttgaactatgctcgttttgagagtaa
aatcatagttcaatgacaaatttcatcacaataacaataataatagtaataaaaaagtttcatctctaaattttggaagttatgtctattaaattcatgtaatttt
ttcactaaaaaaaaaaagttcatgtaattttaaacgtttaaatttttgagttggtctatttttttcttatactttaaaaggtttgtaatcattttcaaaattctcaatttg
ataggtaataggtaatcattgctaacttcgtcaatctttttttagtacaatagatgtggagatgagaatttgaatctctgacccaaaaaaaaaaaagagtaaat
gttttaaatactgagttacactcatgttgtgttaactttgtcaaatatatatatattttttttggacaatatgtagggagggagtttcgaaaaagaccttgtggt
tatcagtataacttcatgtcaattgagttaagctcttgttggcaactttgtcaatttaatacttccgtgtcatgtcttttaaattatttaaggattacatggtaaaa
ttgatttcgagtaggttcaaatatttgttggtgtaaacaaaaatgaaggtagtgataaaaacctataaaaataaaacttaaaatgatcaattattttaaaacg
tattaatttaattaatacacaaccatcaaactaattttagagatttactaatatcatcctaattcaatggttaagatattctcttaacttaacaaatctccactca
aaatttgttatactaagaaaataaataaccaatcttacaaatgtaatacaagctagcaatttagttataaaaaaatcaagccaacaggagcatagctcaa
ctgacataaagttggactgataaccacgaggtctgtggttcaaatcctcacccccacatgttgtaaaaaaaaaagttataaaaaaatcaacaaattgaa
ttggcactttttttctagttaaaaaacatttgggggtgagagtgagacattcgaacatttgagaacctctttgttgagagtaaattaattatggttgagctatg
ctaaaaaatgacactttttttgttttcctttttatcttctatgtcttccactccatgaaaaatacaaatgaatttttattcaactgttttttttaaggttattaattagtcta
gtcactaaactttcagatctgtatctctttgatcttgaaaattttaaaatatctaatagatcattggattttcaattttatgtcaaataggtttttgaactttaaaaa
tgtttagtaagttcttaaaccttcgtatatctaacaaatctctaacctatataaaatttttaaaaattaactatctattagatttgaaattaaattttatgtcaaagg
aatcctacccctccaatcttatgtttgataggttcatgaatttcaaaaaatctcaaataggtcaaagacctacaagacacaaaattgaaaattgacttgtta
gacacgacatcaaaaattgaaagacttatcgaacccaaaattaaaagtccaaaaacttactaaacatttttaaaagttgaaggaccagatagaaaaaa
aaattaaaagttgaggattaaaaaaaaaacagataaaattatgtagggaaggcaaggtcatagctctttgagttgacgaaacatccattgactttctcct
cccattccttttataaaccccccctttctcttcttctcccttaacctctctccctctctccacttctctcttccactctccgcaacattccgatcatcttcctatcc
c

>LcWRKY13 - Up_Stream_Len 2000

actctgtagctttctgcaacgttgtaaatcgtctcatttattttcatgccaatagcttaaagatgggttcgctgaaggaaaagatcttgtcctaaccgtcatg
tctgcaatgggagaggagcagatctgtgctctcaaggacattggtcctaaaagttaaacacaaagctttggctacgtctgatggctcagaatcccca
aaccaaagttctatcaagtatgtttatgtaataaaaacatattatcagcagtgtacttaaaagccgaaacacagctcgtaacagatgggaaaaatattat
agtaaatgttgaacctcccgatccttgcacactgctgaaaggattggatacatatgttacccttgctttatatagtacaatacttataaaatcatctgtcatc
aacgttatcttctgaaccctcttgaatttgcttaagatcttggattctgcttgtccaggttgttagcctagaaaagaaataggaagataaacaaacagttgc
gctggtgttctggtctctttccctttattcttttgtacggagggtgttattaaggaacatttaagctcatcgttgatgagtggctgttgtttccttgaaccaaaa
gaggatgacaagttcgagggcttggcccgaccgatatgactggctctgtaattattgtcctaaaggcagacaacacaacgtagttggacgtgtctcc
aacaagctgctgaacgcctgaactgaaccacacaaatctttgtagtccaacaagctgctactcttagtttcaaacatcaaatagtgcaatcttatcatcgt
atgaggggaattatgtatgttttatgtgtcctcatacttatacccaatatttagatttaatgcaataaatgtttcatttgatgtgctagatattttaggtactacta
tgacagtgagcctcattttcttgaaggtggtcaagttctttttctttttttgagtttaacatgtagggtcgggattcaaatccctgaccttttggttgaacgtcc
atgtcttaaccagtcaaattatgcttaggttgacctaaatgtggtcaggttaaattagaattgaattggttaaaaaagcattagtaacatcgttgaaggttg
aacattttgatttttaacttaacattttcaaaataattatttcatgctattcgaatggaagtgttaactcaaataaatatttttaaattcgagtaggttgtccactt

ataatttttaccacttcaaattaaaaaatgttcacttataaaccaaagtcttgacttctgagggacattttccttcaaattggccacttatttattaaattagag
attaaaataaagacagtatcacatgaataaaaatcgtttttaaaataataccaggggcattaataattcatcaaaaacatagctgcaaatatcctattatg
attttaatgcacaaaatctttgtctacctaaattaaatacgaagtatgaactaaacaatacattttttccccctattttaaaatagaagaataaaagaacaga
atctggggaaaaacaaaacctgtaatgaaaaataattaaatattgggacaatggaattagtcactgtaattgagtcaaaagagaagccccatgtggcc
acaaacgccgtgtaagaaaaccgtcgaccaacagaatccaacggcgatccaaccatccaagaagatcggacggctatgtagtgacggacaaact
gacggtgtccgttaaccaaaaccacctgagtcaactacagtcaaagacacttatgcccatttcgctttaaagcggcttcttctctctctctctctaaaacc
tttctctcacctaatctattcttctatgtcactgagacgcagactccgatactcgatttttgaatcggcgttggtttcggatttgcttcgaacactataaaaga
acttgcaacagttggcgactttactctctctctctctctctcttctagctagaatcggcc

>LcWRKY14 + Up_Stream_Len 2000

Gaggaatgaaaatgtaataatgagcgttaaaatttagttgattgagatgtatatctcccatcaagaggtcagagtttagaggttctaatcttcctcctttgt
tatcttattggactaaaaaaacataactaaataacgaggtggctagtaaactaactctttttttttttttttttgaagcaagtaaactaactaaaatttaaaac
ataaaaaaatagaaacaagtcattatgaaaagcaatgttaaataagacattgaattcaaacatcaagtaataatatataaaattgattattattatatcttgtt
tagtggttgactcttgctttagttgaagttggaagaggggttcatgggtcaggtagggtttggtccactttaattcatccatcacatcaaaacaaaataac
cagacaatgccaacactaaatgataaatttcagagccaataaaaacaagctaaaaagaaaaaagtacttcaatataatgaaataaattatgtgacacc
cctctctttttttctattcttcttcacacccctccttctcattaaatgcatccattaattttattataatccaaagtcacaaaaaaataatattatcatgcataaaaa
caagtaaaagtggagaaggtcaactgtgactattgctacccttgggacctaaacttcttttcaaattcatttttgtaccaaactttttatttgtttatgcaaag
ggtagatttgtcatttcatctttatatcggatcttaaatttgattttgaatgttcaatgtgtgatatagataatcaaagtttatatagtacatgtcaatatatatca
ctcttattaaaattgacttgatccatccgtaaccttacggttttgtctaaatttttcttactttagaatatatcaagttaatttatttatcctatattttcttttacaa
ataataatttttgaataacataaatatatttttgttcaattacaatagacgtgagagtagatatttgaacattcaactaatttgaaatactttaggcccgtttgat
aaccatttgatttttggttttcggtttttgtctattaagcttatacacacatacttccacctataagttacttggttttagtatcaatcttctatattgttttcaaaaa
ccaagtccaattttggaaaaaaaaaaatagttttcaaaatcatgtttttgtttttaaaatttgcctaggaattcaaatgcaaccttaactaggatgaaaaccttg
aaaatttaggagaaaacaaacataattttcaaaaaccaaaaaccaaatggttatcaaatgtgaccttaacagttgagcttaaatgtttcaaacacttgtgt
taacaactacatttatatatgtttgactataattttgttgttattgattttttttttttcttttgtttattagtagaaaaacttttcatttgaacctctaaactcattttaag
tcatgtactttgaatgacgtttgggatgacaaaaatgtttttagcctaatgaaaaatacttttcatttttttttttttaagaaaatgtttggacaatatactcaaa
gaagaagcatttaattaatattttttaattaaaaaattcttcacttaagaaatctacctcacgagaaatcccaaactcgctataaaatgagtaagctagca
aaattaaaaaaaattcaaaagagaaacaaacattgacgacgatgtaaaatacactttaaccaaaagaaaaattaccactcaataaaagacaagaacta
aaaaattcaaaagtcaatgtacaaaaaaaaatatattttttttgcaatattttgaaaaaaaaaaagggcaatcatagtgatattactataaaaagtcaagat
gccaacgacgcacgtaaaagcctctccccatatcctccattctcatctactccactttatcctctcttcttcttcttcatcttcaaaaattcaatctctcttttctt
cttctttttc

>LcWRKY15 - Up_Stream_Len 2000

tgagaatcacacattaaaaagatgagatcataatagtataaattatttgatatacataaccttgtgcaccatcttgaacgacatgttgatgatctcatattga
aaatatcaatatgaggtcataatattacaagttaaataaataaaatatttgacatgcatcgttacttgtgtagaacataccttagctagaattaaaaaaaag
atatttaaaaaaaaaaacttcaaaagaaaaatcaatgataaaaaacggaaaaataaaaataattattcaaaatgtcaataataattcaaacaaaaacttgg
ttctatacttctgttagaatctctacatgtaaataataaataataataataataatcataaatagtaggggtataagctaaaaggggtcaaaacgaggtaa
agtaaaattgatttagatttagttatgattatatggcgagatattttgattttcaaaagatatagttatatttagataaaaggttgctatataatttatttttaatttt
cccctcaatttattggaaaattgtataaaataatttcagtattttgattggataactatttttgaaaaatagattgaaggtattttatccataaatagtgatttc
aagcacataataatatttatttctaccaaccaaaaagagaaaattttctctacatttttctttacacacgaattggtaggaaaaattatgtaaagatttgcgg
ctgaaaataaaatcattataatctgatctttctttgttggagtcaaaaacctttctccttttccctttgtttcctcgtgtatcgtttttacgccaaaattccacttaa
acaagaaacatggatttttaatcattttatgaaaaatggtaggaatgggaaattgaggagcggtagagaatacaattctaatcttgtttgtcactagatttt
gaaccagtctttgagttgactaattgtgcatatgttagtctagttcgaggtgagttcacgaatcacgggtcttgattgatagcaagagtatggaatgtttgt
tagactttagtctgacgctcaagttagtttacaagtctaaaatttgataaagtttcgctaagttgcgagagagtacttacctatgcgtctcttctctttggattt
atagagatactagaggtggaaggagcttactattaggagattgtgggacatgctcctagtggttaagttggatgagttagcattagatagttggcaggt
gcaagcaatcatcacttcactaacttacctcaattttggagacgacctcgaagctagctcccatcctatgtcgttcactctggaccgatcgtccatcacc
catgagatccccaagcctaggcttagtgttcacagactcaaggtcgttgtttaggatagtctagctcattagccaaattgtcatcccatcgataaactga
ccgttagaatatttctaatgataatatctttgaagaaatattaatgtaaaaatcacataaaatattaacataaatactaattataagacttttatcattagtattta
tgttaatatatatgctatatttagaaattataggttaatcccttgtatcacaaggatcaaaagaaaatcaagaaaacaattaaaaacaagttaacaatcagc
atataataaaattagtcaataaataaatataaaaaaagaatatgaaatactaccatactggttttttttttaaaaaaaaaaaattatttaattctcccttttattttgg
atcaggaaaaaaaaaaaaaaaaaaggaggggcaaaagtgtaagaaagtggaaacagaaagaaaaaggtgggtcagttaatttgttatatataaata
tacacacagattagatgattcctgaatttgcagaaaattctctctcgctctaaaaagtttcaagttaagcagtcagaatcattctattctggaatgaagtttc
ttcttcttcccgatccatctctaacataaattaaggaaacaga

>LcWRKY16 + Up_Stream_Len 2000

Agtaaggctagttccttcttgaacaagaaaaggaaaaaaaaaaatctcaaaattaaatatcttattcttatgcaataataatgtggatgcgttgaataaaaa
atattaaaaaaataataataataataatgtggatgcgttgaaggaggataattttgtgtcgtcgtgattcttattgtgtattaaatttgtaaaaaatgaaagaaa

atgaacttattaattccttaggatagaaacgaaattcttattcaacggtgggaccccaccaacttttcttttatctaattttttttattctctctagtttttatatttc
acatcactttgaatttgattcatgtcccgacccgtagtaaacattatcaacaatatttatgaatttcattccatttttttatttaaaaatagttttaataaacgcag
ttttgaaagtaatttattctcattttttttttaacttctggccattattaggacaagtcgtggagtcactccctacacacatctattattgtgggaacgcttttcttt
agttaaaaattgagtttttatcctgcttatttagttcatctatttttcatagaattgtgaaggttttgaattgtaccatttatttcgcctatataataaaattttaaaa
attaataagttaagttactatcaacatcatatattaggttaatgccattaaatactataatttgttcaaaatcgtcaaagcggacacaactcaacagtaatta
acacacatcatcaactaagaggtcaaaggttcgaatcactcacttcaaatgttgttgaactaaaaaaaaaaatttgttcgaaatcaattcactatatttaaat
aatcaatatgccacctcaatgtatttaaataatcaatatagttttgaagatcaaagttgtccaaaaaaaaaaaattgagacaattgaatggatgatccatttt
gagaggtcaaaatgaaaaatgtcccaccttttcagaataaggatatatgttcttttgcttacgtcatcatcctagcaaaattaccattttattttccatatgaa
aacgctcattttaggcatttaagagcgagattggagagagaaattagagagagagtaagagattggagggagtaattggagagaaagagcgataat
aagagcgagagcgagattggagagagaaattggagagagggctattagattacatgaggttttatctcaaaaccaattggcaatgagtggagtatcc
tcaacatgtcccctcaagatggtgcctctttgggttcaccattcttggatcggatcccaatttttttttattagaccgaatacccgtttgggtttaatgggctc
tgataccatattagattacatgatgttccatctcaaaaccaattgacaatgagtggagtatcctcaacaagagcgagaagctagagcaagagtagcga
gagagagagagtgagattggagtagagagtaaaatggtaattttactaggatgctgacgcaagcaaaaaagtcatatattcttgttttgaaaaagtgg
gacatttatcattttcgcgtattttagagggtcatccgtacaaaaatctcccgaaaattgaatatcaaatagtcataatcgtctttttctatttctttttctttttc
tttattctttttaaatttttctggaaaaaaaaaagagagaggtcgaagaaaaacacagtaggcggatcccacttcagaaactcaaagtgacgtcacaa
ttatgacctcaccacaaccaataccacgtgtcaagaaagagaactttcctgacaggcagctcgaatcgaaaaggaaggtgagtaggaaatagcga
actggaaaaaaaaaaaaaaaaagatattaatgaattaatttaaagaaagaaaaaaaaaaaactttatataatgtaaagcaaagcaaatccaacaaggaa
ttcccttgcaccttgctcccccattttcctcctcttctaaagcatttactttatcc

>LcWRKY17 - Up_Stream_Len 2000

ttgtattggttaaatttatcaactttataacatagccaccattaagaagatgactaattggacaaaaatcacacttattaattactctaaaataacaaagaga
tccattgtttagataactaacaatatttggtcaaaataggtctctgctagtatttatttgaatgtatatcccgtaactttacagtttattctcgagtcaagataaa
tcttaacaaggaatttcaaatagaaaaaaaatctgataattcaaccctgtcattcacattttctaccacaaccaaccataaaagaagagttatttctacctc
ttctaaatattcctaggttcacatccttactttcatactttattaaattatttattctaacctgattatgatacacggttgtaaaattgattttaaaaatacaaatat
acagaaaaatgataaattaaaaatagtacaagagcaggggaacaagaaagtcaactgaagggtgctgtatttgacagggagtgagttggcatggat
cgatcacacgtggccaaatttataagtagtcaaagatcaaacggctaggatcacatctttgacctaatcaccatctttgtccttttgttctttcccatctaga
ttctagaccagattaacttttgtggtccccactttaactgcaggtcaaggttctcgacatcaagcatcggattccgagtttctttttcctcctttcaattttatt
tttctgcacattcatccataattttggcactttcgtttttaaactgcttctttcatttttattttttttaattttgaattatactagaaaatgaaaatcgagagcaga
taaatttatcttttaagaacaggatagttacaaaacgccaaggttcatctgttgttaatcgggtttctcactcagacaccattttggttatattatttgtcttata
aaattataaatttggaaggttttatctatttaaaaagtctttaagtatattctattgaggatcccacattaaaaacatgagtggagacctcacaagttataag
aaagtgagttactccactcattgtcaattgattttaagatggattccatataatcaaatatattcttgtcctaactaagtatatagatgtagattcaaatattata
tattgttgagaaaaataattgagtattgacaatatagatgacaatcaagtaaataaattaaagatagagataacaaataatattgtaattgttgtgttggatg
ttgttagaatttttttgtccgggttaattgaattaggtttgtgaaggaaaaaggattgaaggttttgtttttttttagaaagatgaggaggagattggaatgagg
acaagtaatttgagttcacatctaaaaaagcattaagtgaaagtgttttcttcgtcccgactagaattcaaaataaagagcctacaaggatccgattctat
ctaatttttttgctgtccattagtcaacagccgacgatgcatgtctaatggggaactgagctcactaaccaaaattactaatttctccctttatcatccatcca
cgattcacattcacctacctctctaatgtaattaacaataaaaaaagaattttagacacaaggttaaatgttttgagactattatattggttttgatatgaatttg
aaaattaaacattttttttaaaaaaaaaaattaaaattaaaatgatatattagagagacatgtgggagttgaatggctaattttgtaatttgataaaagaaaac
aaataaaaaaacagaaaaaagaaagtcagtagcgtgagaggggtgagtgaggcagtgggtgtatttagactttaatgggggaaattgagaaaccatt
ttgaccagagagactctgtttctcctcctttatcaatcaaacgttagtctgcttaattgtttacttttgccaactcttctttatctttttttgccctttttttctttctcag
gcccaagtgtttgatgggtctgtagat

>LcWRKY18 + Up_Stream_Len 2000

actatggtgtatttttagactactgtgttattctttttttatagtttcttgcctactattaattagtatatatatataattaattcgtttggcgaagaaattatcctgatt
gtgcattttgaaataaattaggaggcttagtgttagatgcttaaattatttcttctcctattgacttcatctagaacaagccccccactatatattaattgagg
cgaggacaatttgggcattatggtagctagcttgcatatataagtctattttattttttatttaaatttttttcctttcttcttccttcctagttattcgaggtctttga
aagaagaaagaatgaaagtgatttataaaccaaaatatatattaaatgaatcaattaaacaaagacctagagtatatctaatatcataataggtagttgaa
gaaaatgattggtgtgtcacaataacaagttaacctcctctaaatccaatctcatttacattaatctgttttttttttttaattatatataaataaagatttacatttt
taatctgtttaatttttttaaataacatataatacatatagttaattaatttatttctttaattattttaattaatgtattcaaggaactctcttttgtttttcgtctttaatttt
tatttatgtgtacgatataggataattttcaaaattcctttttgtaattttatctatttaaaaaaactatttcacattgtttttttcttcatttaagaatatatgaagacg
aatatttgaacctctaaatttttagtcggtgggataacaacttaatcaattgaattatacaaaataatttgggtgtttttttttttaatacaacttgcgaaataatt
aacaaaagaaaggttagtaaatttgattgtggcgaaaaagattaattagaaagaaaataacagaagcaataaaaagaaatatttgtgaagaaaagga
gattgattagataatttagattagatgtgtaggtgaattaggtctaatacgaaaggaaaaatgatttaggtccactgttatgaaagagtcgtgcagtagta
aacaaaatgacgacatttaatttttttaaaaaatatcaattaaattaggcaactaatttttcgggagtttgtaatattggaaaaagaaaaccaatcagaaga
cgaaatcaagctaaaatggtatttttatttttttatttattaaaaaaacaaaaaaaaaaaaaaagcaaaaaaagtcaaagcaaatgactcggtcccacgggt
ggacagtcaacatagatcccgcgggtacagatctgaaacgtggtgtcttgtaaagtgaaagggagcaaggtgggccccatcagagaccaggccc

actccccgttgccttcagagagagagccgggccgtcaaattcgttgacttggctctctctctttacgagaccttcaaccttttcttttttttcttgtttttcctcac
gtggaacgcgccgcaggattcggggcggtggatcggtttcttcttccttcggatttaatgaacaagtcaaactcttttcttgacgaaatgaatcccaaac
gaaaaagaagaaacaaagaaggaaatcccacccaccctcctttttcctttttttttttgttttcccacgggtcggtcctctgaaaccgggttcgggttgt
taaaaaaaaataaaaataaaaaagacccgtatggacgcgaaatagagagtgggaccaaaaaaagtgccacctggcaactgacgcactatctgatgt
aattaaactaaaatcttacctattccgtagaaaatccaaatccattttggtcttcgtccaaccctttttatagccgaacgcattgcaccggtaaaagacttc
acagacttcacccacacgtacacacctcttttacaatttccttctcttctcttcctttcttttctccttctctttctctttctcttcagcttcactctcctcttttacttc
tcccatctctctctctctttgattt

>LcWRKY19 + Up_Stream_Len 2000

Cctcaaactctctctttgctttaattttgtttttctttattcttttttcttttttttggagtttcaccaaacattccctacgatcaaaattaaaatattattttcgattga
aatatggatatcccaattttacgaatctggttggaaatatctatatcgaccaatattttttatactagtaaaatttaaactttatttaatatttaatttgataaaca
ggttattttcacttatttataaattaaatcttatattcgcatttgtattccaattaatattttccatatattattcatattaagatatttctatatctcttgtttttaaatgt
taatctaaatataacttaactaattaaaatgttaattaccactaatctcctttcaaagtgggtaaagttggggaaacatttctattttagagataaatgtattttt
tttttttgtttttaattaattcatacaaaaattctaaatcgtttataaagatacaaaattttaaaattaaaatcaaatcataataaaaataagttatggtcgtcaatt
ttttgttttcttttatttgactaaaatttcaaaattattttaaagggctaagagacaagacaaatgaaatatagatataaataatatttataaattcagtttttaata
attagatcattatcgaacgggtttagattatcaaacttattacaaaggaaaagtcaacgagaatttttttaaaataggccgatgatattcacgaaataaagg
gagaaaaaaaattgtttgcattcaagtcatttagtcaagtttatgtgtgtgtgatcactctacctctttttaggtctaataaaaactaattagtcttaattagata
gcatatatgtagatcatacaccaaatgtactaaaaaaatcacacaccaaacttcaactttctaattttttttctcataatattaattatttcctaactccacaca
ataattctaaatcatcttgacatgaatttaaaataaaaaaatatcgacaaactaaaaaaaaaatctaaagactaaacgttaaacatttgtaatgtaattgcatat
atattacatcttgcctcttcaatcgtaaattagatatgatgtaagggtggacgactttctctttttattcaacaaattggacgaaagacgacgatataaatat
attaacttatattgatatgacattactaattaacttgaatcggactttaattagtcccttccattaaaaaagccttcaattctaatatttagaatttaaaaaaaaa
actctttctctagcaattaaatttcttgttaacctaattaaaaattaattagagctactagtagatgataaattttaatatttattacactctcaacaatatctctc
acattattgatctcagatttaatttcgacttacgcaaaaaccaacatataaattattaacatatgaaatgtatgggcagagattgaagatttaggatgtacta
agctaatataattaaattatttgactaacataatttttcttagtttcgaatatgaatacgtctgaagaaatatacatatttttaaggaaaaaatacacttttagtcc
ctgagtttgagttaggtttcattttggtttctgaggtttcaaattcaacaattatgaagtcgaccacactattattgcttttagcagctataataatagtaaatta
attaattaataaattaattaaattgtgtagctgaaaaaaatagaaacccagttcgcgtcaaaggtctttatagaatgattattattactggtctcaaacctaaa
cacttttcatttttctaacttatttatttatctatttatatatatatatatatatataagaagaagaagaggacgacgatctaatccagaagactggacctaaagaa
aacactgaacccacacccacagcttctgcttcttcttcttcttctggttcttctggttcttggagacgtcgtcgttttggtattgtgattcc

>LcWRKY20 - Up_Stream_Len 2000

ttatcaaatatcgatcgagttgatggtggaacgattctgttgaggactcttgtaaaataattgtacgttttggtcctttatttttcgataaggttgctcaatttttt
ataagatctatgatttaatcgactgtttgatgatttgattcaagatttaatgatatttattttcgatgtaaactattgccctatttttacaattggattcctgaggtta
aatccaaagacttgatcaattgtttcaagaaataatgtcggttagtcatagtttcatttttttacaatgaaattgtcatataacatggtaaatttcgtgttttgaaa
aatatataataataataatggcaaaataaaataaaataaaaaattgtggattttttttttttttttttttttttttttttttttttgtatttatagaattcttctatacaaatttat
tgtataaaattgaccttatgcatatatatcctttgcttaaatatattagtgaagcacaacatattcctattatatcaccaataaattttaggaatgagaaaagta
taattcgacataaaagagatcatataactttatcattttatttatttatttattttgatatgttgagctttcttaatcaagagtaaacactttatgtcaattgagctat
actcttgttgacatacaactttatcattgaattagtatttccaataacatttcaaaaattattttttaggattaatatagaaaaatcccattcgacaatattaaata
taccatattattctccttcctttttagtacaatatttatttattaatattaaaaaataaaccaaatttaattatctatgagctagcttttgcgaactcaaaactaaca
aatttttactataactttatgttagaacctaattaaataattacaactcattaaaaaaaaacgtatctgataataaaccgaacattcttatccaattaagcatacc
aaatataaatatcaaatttaggtcgaattccattacttaactaaaactatttaaatggaacaaatttatatcccaaataattgaattaggtctaaaatagatat
caaattgccactgttcaaactggcagcagagggaaagaaacaagtgcaaatggaaaaaaaaaaaaaagacacaaaaacgaaccgttttatgagaat
tgtggctctatttggctttgcctatttggtctctatgtcctcaccacaacatcactttgttattattatttttaaaaaaaatttaaaaaaaaaaaaccacttttttttt
ccttgaaagctctcaactttaattaaccctttgcgtgtaagagcaaatagagattaaaaaaagttaattactcctaattaaaaatgatagaaatttgactaa
gtgatgctagttcatacaagtccacacgcagctcaattccctttttgtttaattaaatgatggcatgtttatagttctaattggggtaaatttcattgatttattat
aacccatgatacaaaattaattgatgtatatgaagctgaagtgaaaagtgaaaagcaccttgctgattaaagtaaatccaagaaaagatatataattaaa
tatatttttttttaattaataaagaccccaataggttctgttctgtcacctctattttttaagtttaaaattagttaactatcaacctcctttccttttttccctcctcca
ctctcctctatgtagaatagaagagctataatttgtgttaaaaagtcagcgactttctctctctctctctctctctcaatttttgtttgttttagaccaagaaaaa
tgccctttaaagcctgttttctgtgtgccttttgtgcttctcttcaataaccaaaatcatcatcagcaacaacttctcttcttagcttactacttgcacaagaag
ggaaaagaaaagaaaaaaaatcaaccccccacgaggaaaaattgaatctttggttgttgttgatgaatttcttctagagtgggattgagagaagt

>LcWRKY21 - Up_Stream_Len 2000

caaaatcttcaaacaccatatctctatagtcaaagtgagcatagttcagcggcaattgtcaattgacatgcatccttaatcaaaaggtcagatgtttgaat
catctcaaaaaaaaaaaaaaaaaaaaaaaaaaatttatatgtattattcaatctttttaaaattaaaacatgagaaaacaattcttccaaaccgaatgacttga
caagtttcttatagattcaaaggcaaaagagaattattcatcttttaagcccactaccccaaaaaaaaaaaagaaagaaagaaagaattttttgtatttaa
aatgagataaccattatcaagtcataaaactgtcaaaatgagtgacttcaaaaatttcaaacttcaaactcattcacatttggaacctttttcgataaagta
aaagaagcatacaaaataattcaaactgttctatcttctagcaatataaaataagagagtgagtgttaaaatcaagatcaaaatgatggatcatgttccta

aagattaaaggcaattgattgagaaataggaaaaagacaaaatatttcaaagaaaaatgttgaggggaagaaatcacatggattatagtagtaaatttt
tgggcatgttatttggttgtttgtaagtcttataatcattgcaataagagaatatatattattggctttaaatttaatttgatactttgcatttaatttaattacctgt
ttgacaaaattaaatgacaagacttctcattgccctcacctatattcagctcatctttcccatcatattatattagcttagccaccccacccaaataaataac
tatatatatatatatatatatatttattgtatttaatactaaaaaaacatgttacaatataatccttttaacacatcatttagtttggaaaaaaatcattttttttgtg
tacacctagctcaatttttcaacttttaatcttaaaagtttggatatttcacatcaagtaaactttgaatttgattaagtggtgcaacaaatatggtaattagttc
gtaggttaaaacatttttatctttcgtaaaggaaagaatggtcattaattaagttcaaatctcccagtgtaatttttttaaaattttatcattactatactatttttaa
ttgttaaaaatattattatgataccgactatttaaaaaaaaaaactaaatattaagaaaattaaagcaaacaaagtggagtaagttgtttaagcatctctatt
ttatttaagagattattagttcaaatcttcatcttttatgcctaagataatatattaaaaaataaagatgaaacttataatatatttctaaattcaagaagtaaat
gaattattagtaaataatttacaatttttaaaataaaactattacccaaccgaatctttatacacgacttttgaaaagatgaattgaattcgtccaatatctat
ctaattacaatgattttgattcattataataaaaaacacaaaaacattaattttcgtaaagatttgaatatcttttattaaatccattttctttaaaagaaaaaaa
aaaaaggaaaagattgaaagagattgtgaaagatggaggagacaatgaagcaatttcgggattgaggaaagcaaaaagcaaaaaagaaaaaag
aaaaaagaaaaaaaaattatatttattacgcatgcttacaaaacgacggcgtattcatgatttaagccgcatatacttcacgtgggtcaaaaccccgtcc
agaccgaccggctaccggccaagtacgacgtttcacaccttctgttctgtcggtccacttacttgtttacaactcccactgtctcgtccgtacaaaactat
gacctacaattgccggtcaccacccccctaaaagaacaccctcctcctctcccccaatccccatttcccattattccccaaatcccaacccccaagtttct
ctctctataaaaaatcccattctctctctaggtttttgtaagaag

>LcWRKY22 - Up_Stream_Len 2000

catgattttcatcctggttaaggatccatttgaatttctagccaaattctaaaaacaaaaacaggtttttgaaaactactttttttttttttctcaaaattggactt
ggtttttgaaaacaaggaagaagattgatactcaaacataggtggaagtaggtatgtataagcttaattttcaaaaaccaaatggttatcaaacgggac
ctaagagattcaaatctccgctccatgtgttgttgaaaatcaaaacaataatgatgataaaatcattgaaacttctaaaaacattcgaccaaattgtaacc
aggtccaaattgaagccattatctaaggtcgaggtccgaattttctatcaatgctgcaaatcgaccaagccataacattaaagttgtgaatataaattttgt
ttaaaaaatagaagaaatataaaagagatttttttaaggagaaaaatataaaagagattatagagaaggaacattgcatataaacattactaaaagtaa
caatattgggaagaagctaagcttttcaaactaaaatcaattaaactttatttatttattttttttgaattaaactttagttggatatagagatttgaatctctctc
atttcaatgttaataacatttatttactgtaaaagatattatatcaaatttagggtgtgtttggtttaacttttcaagtgtttaattttgaaaaaaattttagtgtttg
gaaactactaaaaatggcttattagaaaatgagatttataaataaaacattttgaaagaaacacttgaaaccaacttttctaaaagagtctttaagcataca
atagtgtgtaaaaaccctacgaggaagtgcaaccaaatgaacaatttttatacaaaatgtttttttaaaaaattcattcttgaaaaacatttaaattaaaatgt
attccaaacggacccttgatacttacaattttgtattttacacatcgattgaaaaattcactcgattcttttttagttcaacacgacgggggggggggggggg
gggggattcgaacctctgacctcatggtcgagggtacatgccaattaccgctgagctaagctcgctttggcttagttcaacacgacaggatgaagac
aggatgaagatcaaaccatcgacgcttaaaatggtaatccatggtggccacctacctaggatatttaatatcctacgagtttccttgacaaccaaatgta
gtagggtcggacggttgtcccgtgagattagtcgaggtgcgcgtaagctggcctgaacactcacggatatcaaaaaaaaaaaaatggtaatcaaggt
cttaattaaaataaaaataaaaattataaaataaaaaataaattaaaatcaaataaatcaaattgaattttacaccaccattaaaaaaaaaaaatggggag
agtagtacagtaacatggtggaaatggtgggacccacctgcaaatctgcgacagagacggcagtttcatttctctgcttcctgtttcctcctctgccctg
ccatctgcttctctccattttaattttctttaaaaaaaaaatccctttcttctttcttctctctcaatttctccagttcttcacagtttcaatcaatccccgtctctgata
aattcttccactcttcctctttctctcacatcccacttcttttctctctctacaaatcatctttccaaaaagaaaacccaaaaaaaaaaaaaaaaattaaagac
gacggatggatgatgtgcgctttttattcttttgggtctctcaaaattcgcttcagtttacactttattaaatcatggcagaagaatctagggttccctctctct
gttttccgatcccaatttgcccaaactctccttcttcttcttcttcttcttgatcgcctctttctcttccagtttttcgcccccattatgctcaatttcagctagctc
cttcagatttcgtgatttcgcccttttggggctctctcttttcaagc

>LcWRKY23 - Up_Stream_Len 2000

ataaaaaaaaaaaaaaaaaaaaaaaaaaaaaaaaaaaaaaccttccaacatgcttttagctaaagtacttcacctagaaagactccaaaagtgctag
aattcaagtatatattgaacaaagtttggtgataaacaacctattactttctttaaaggtgatcattcatctctactccaactttctttagaaaatgcattttgaa
ctttcaattttatgtataattaaatttcaaactttaaaatgtatttaattaagtcataagcgcttttaagtttgtgtataatagtaataggatcttaacctattcaac
aattttaacttattatacacaaaaatgaaagtttaggaaattattagacgtttttttaaattttagaaaccaatcacatacaacttgaaagtttagaaactaaatt
cataatcaaactttttttgctctattacttaaaaaaaaaaatattttgatctctcattcaaacatatatatacatagaacaatacttggttatacacggagtatctt
cttcctacacccaccaccattaagggcttgtttggaatacaatattttagtttaaatgtttttcaagaatgcatttttttaaagaacattttgtataaaaaattgttta
gaatttaaacactcatatgtttagtgacattttctcacaagtgtttttatacccaagttagggtgtgtttggtttaacttctaaagtgtttaattttgaaaaaaatt
ctaatgtttggcaaacattaaaaatgacttattagaaaatgagattgataaataaatcattttgagagaaacacttgaaaccaactttttaaaaagactaat
aacttgtgtataaaaacatttataggaaatgcaaccaaacatatgagcgtttaaattctaaacaatttttatataaaatgttttttaaaaaatgcattcttgaaa
aacatttaaattaaaatgtaatccaaacaaacacttattatacacttaaaaattgtttctcaatctcattttttaataagtcatttttagtggttgtcaaacactaa
atttttttcaaaatgattttttcttaaaaaataaacacttgaaaagttaaaggttacacttattgctaggagtgagaaggaaagaaaataagaaatttgaac
ttttttatgaaaaaaacttggcccattgagatgttaacaatatgagagataaatctttttctcattttctacatttctatcaaaattttaagagttacgtagagaa
aatctcatattcagtcaagtttttatttatttattattatttttttagctaacgaaaccatcataggttggccctaatggtcaataagggccatagaaaaaataaa
ggacttcaagggaatgggttcaatccatggtggccacttacctaggatattcggtgcgtgtaagctggcccggacactcacggatatcaaaaaaaaa
aaaaaaaaaaccgatggtttttaggaagatattcacgcgtgtaggataatgaatgacaactaccgaccaatgaaataccaatcctctgaaacccattaat
atgatccaatatgtatatatatatatttacatatatttgtcgctgtcaaatttgtgaagaaaattctaaggaatcttctcagtctcttccgcgttgctacaacat

caaaatttcaaatccaagagagaaacaaaagaaagaaagaaaaaaaaaaagtgaaattgtttgatgaaatttgaagtggtcataaattggaccttttcc
aatccccaaacaaaaaattcctcactgatttccattcattatttttacccaatcttttcttcttcttctttccttgtcttttcctctcaatttctccaatcgcaatcat
ggagtttcagtctctctctctctctggtttttccccttcctttataacccttctcttctccatttctttcaatttcactctctcttccatgcttccacgccca
Gacccttctcaccttcgatt

>LcWRKY24 - Up_Stream_Len 2000

Cttatacaagacgagatcgcagaataactaaagaactaaaataagtaaataagtatttaaaaagcttaaagattgattaaaaaacgcattttatttgaatt
gccacggtggatattattcagctttcatgtgtcattaaatcatcgtcatatataatttgttctcttaataataataataataataattttgtttgattaaaaaagaa
aataaaagtcaaatgaatggaccaatcagcagcttctaaatacatactaaaaatttgacctcaccgttattaaaatttgtaaattaaatgtagagagacaa
aaatgaaatggacaacggtggcacgccggtggctaaccctaatctaacaattttgacccttaattataattataattaaatccacttcccatctgacttgtt
tatttgtattttctcaaaaaacccaaaaaaaaaaaaaaaattgtttatttgtattttgtacgtacgaaacttccacccctctctctacccttttacgtaattcctttct
ctttcctctcaaacctaacttcctttctctttctaaaaaaacccattaatttaactaacgagctcccactttttaattcattattccaatcattttcatttttaataca
aaataggtattttttttttacgaacatgtggggtggaaggattcgaaccatagacttcttattcaccgatacaagttttatgtgtttaatttatttttaatttacag
gacaatttttaaaaggtattcaaataatagttattaaacacaaaactattatgttataagtaattttggaccactctgatatgcaagaaatcgatgaggaca
gcgcaatactagcctaaggagacagtcaggaaacggaacccagaggaagaacagaccaaagggtcgggccaaaggcgaagggatcaagcttt
tggcccgaccccctgctcttccgcctccgttcggtccctggtgcccccgactacctcggttcaacccgaatcgccttcgaattcctaaaaatcctagg
gtaagaacatgtatttaaacccttcttcatcactgaagaaaggatcccgaactctattctctaactcttctcttgcccttactctcttgcttcctattgttctgct
tgctgacttaagcatcgaaggcagtgtggcaaacaccacaccggtgtgcaggttttactgtcttgcaggtcacgtatttcccccctctcaaacaaatgta
ccgttggtggcacgtgaaggtcaggtgagttcctcctgtccagattttgccatcaacatattatatataattcacctaaatttattagacatgcatattgtaaa
gttgaagaatattctatgggatatatatacaacatcaaaagttgggaacctattagatatttcaaccaattaaatagatctatagtttttaaccaattatttatt
atctgacaagattatatcgacatattcgtttaattttttcgttgacgatttatatcgccgtcgtcgtctttacttgtctgataagaaaagggaaaaaatggatt
ataaaaggaaaaaaaaaaaaaaaaaaaaaaaagggaagaaagggaagaaaaagtaataagagagacttgttgttgggcacacattttatccaaag
agttgacgtttgaattctttttgggaggtagtttaggtttgattaatattttaatacataattaaaataaagtcttctctacgtccgataatataatgtgacaaa
aaaaataataatgcaagttgggggctttgattggactggcaaaattgagggtttgacatttaatggagaaaggggtgcgttgagatttgaaatccaaa
gcccttcaaattactcacatgtgattggctgagagctatggcttagtcaaatacagccttctcttctctttcatttcccctttatatatatcttcttttgcaacttc
cattaaaacctcattgaaagcacatcaaagaacaagactagggttctttcc

>LcWRKY25 + Up_Stream_Len 2000

tgtgaattataagaagataattatgtttttcgtgaacttttagaattgtgtttatttaggccccgtttgataaccatttggttttgagtttttggtttttgaaaattaa
gcttatacacacctattcctacctatagtttctttgttttagtatctaccttgttctttgttttcaaaaaccaagtcaaattttgaaaaccaaaaaaagtagtttcc
aaaaacttgtttttgtttttgaaatttgacaaggaattcaaatggttccttaacaaagatgaaaaccatggtagggaattttggagaaaaacaagcataatt
ttcaaaaaccaaaaaccaaaaaccaaatggttatcaaacggggccttaatttttgtagttttaaaaattcctaatcagtcgttgaattttcgtattgtgcctg
gtcttttcggtcagttatgttttaaagaagtttatattaattacataaattacaattctttttgtgtttaatatatattccattgttaacttgtttagttcgatgcgtaa
cctgacatattgattatattatttaagaattatcaagggtgggctgactgccaaagcgagcttagctcagcggtaattggcatgtatcctcgaccatgag
gtcagaggttcaaatcccctcactcccaaatgttgatgtactcaaaaaaaaaaaaaaaaaaaaaagggtgggctgactttaataagttcgcgtatttagt
agggtgaacattaaaatggcagagtaaataacataaatctattaaccagaaagaaaataaaaggaaaaaaaaaaaaactcagagagactgttataaac
atggccttaaaattttcaaaaatcagacttataatttgaattaattatgttttcaaaaaaattgtgatggtaacacataagtctaatccatactttatgtttattta
gcttttttcgtttaaataaaaaaattctaattctaattacatcttgaaatttcgtggttgtatctatctaattttgtatctaatagatccataaatttttagaaagtatc
aaatatgtcaaaaactgattagatacaaaattgagagtataggaactcatgacaaaattctaaactttaagaaataatacaacattgaaattttgtaaacct
taagaaatacaatattaaaaatttaagtgcttaattattagacatttttttaaggttcaagtagttaagacatatacttcttcaacctcatgttcgaaactcttatc
ctaaatgttcacaattttataattatgagaataaaatttgaatttaatgagaaattaaaaaaaaaaaaaaaaatcagagagcgtgttataaacataaccctaa
attgttcggggataaaacttataatttaaattaattacatttttaaaaatatgcaacggtaacaaacaaatgagtgaagcctataagctgagtttttaaaatc
aggattctttaccattatcatttaccataaaattatcctctcttttctaatgaaggtaaccacaaatatagaaaaacattaaaacgaaaaaaataaataaata
aataaataaatccagatcaaatatatatagcaaggataaaatttttgcaataggtagaaaatcccacataatttattattcctaattgtccaaattaaaaccc
cacaaataaacccaaaaaaaaaaaaaaaaatcaaaatccagtgaataattttattcaaattgaaattgaaaaacacaaatttctccctattgtcacaatca
acagcgacaaattgtcaattttaatatcaccaaattgaaagagattagatagttattaagaagtagtagtcgcacgtccccccattaatatttgtcaactct
acaccccatttttttaaaaccacattaaatttcttgcttctctcttcaaaattctctgatacaaacttttccgacagccatttccttaacaaaacaaccaccctct
acggctactccggtggcggccatagtaac

>LcWRKY26 + Up_Stream_Len 2000

ttctttattttttttttaaatatgagatgggttgaatgctttcagtttagatagatttagtctttttataatctagtttgagtttattaaataacatgagatttcatatca
aaactaattgacaatgagtggagtaatccatggatcttataaattgtgaagttttcacttatcttttcaatgtgagatcctcaacagagttgctttaccggctt
aacttcgtgcttaggttggatttagatttgtttttcatgattttgagttttttattagtttttttcttgttatttattaattaatttaattctcatccagatatactaatttt
gctactttgtacgatttcaaacacagacacatatatccaacttatagagacacaaagaaggaaagaaatcaataaagcatgtcataccaacattttgcct
cacaaattttcactttacactaaaatttgtattgttataggtgaatgtgaaccatcccaaaaggcaaggagattacgaggacacgcgacatcaacccga
gacacatcctagaccaaacaataggaaaggaaaagggccgaggggggttgggtcggagcccaatccctaacctcgaccttgaacggaggtcaac

cctaggctcatgcccgaggttggcccgatttttccgatgtgcggtccctgaagcccagttgacccgaaacctaattctaaatcgtcccttaacgaaga
agaaaaccctaaaaaaacaacccaggtataaatacccttctgaacggctggaaaggagggggacactttgattcttgtcacttcctacttcctgttcgc
taacttaaacatcggagtgactgtggcaagcaccacatgcaccaatgtgcagtttttactagttttgcaggtcacgtcttccccttcaaacaaattcattgt
cgttgtcacgtgaaggtcatatgagcttcttgtccgaattttggcatcagcatgtataacatcattctttcctttagagttattatcacttattattattttttaaa
gtacatcaacatttgagggtgggggggattcgaacatttgaccttttggtcgaggatatatgtcaattaccgctgagttatgctcgttttgacagttattatc
acttattaggtcattgagacatcatcttcaattctattcctcattctcctaattatctctatactgtctaaatcaaatatgcatcttacactatgacgttgtaataa
atatatcaaagtacttccattatagtaagaatcaatttgtcaagaaaacaaaaatcaatcgctctagcaaacccaatttgcaaaatcgtcatttctataaatt
tgaaaatggaaaattatgaaaactaatatataccatgaattatctttttaattccatccaattaaaaaaaaagtgtgaaaattaatatatatccaaaaaaaaa
aaaaaaagaaataataattgaacggagagaagggaatcgaagcgttctccgaaaaatgcggctacttgccgccttctttacttttccatcacttgttttc
caaaaatgacttttcttccagtctttctttattattattattattattatacattattttaaaattcaatattgatcatattaaaaaaaaattataataaaataaaataa
aaaccctccaaatcccaaatcaaccccataattttcccaaatttcagaatcaacccccaaaactgcttaaatttgactcttcacgcatctcagttcaaccc
ctccacttctaatttatttattatcactctctttctctctcctcatgttgcgtatggaaggaagagaactggtttttgtgagctagagagagaaagtcagcgtt
ctatttctctctctcaaaatttctattttccatttaaccaaaaatcccccatttccaatttctctctctcaactcgccggcgacaagaattctgcatgttttccgg
tgggtctccggccaacgcagagacgaccctcacaac

>LcWRKY27 + Up_Stream_Len 2000

tatgaagtagcaatgttttaaccaccaaccaaggtaaatgttggtaaaattttcaatttttgatcaattatcctttttttaatttaaatttttaataattataaattt
aaagcaagtagatttcttatttctactactgaaaactactttccccccctcaggttcgtcttaaatttaaattactttctttcaaattaaataatttgcagagttttc
tcagtaataattcgactgtgattcttcagactcatccctttcgctgcagcacgtgcatcgcccaattcaactaccattcttcagagtagaatttcagttgga
ggctcttgaggtcgggcgttggcgggattggccaaaggccgctgttcctcttcgtccccaccctccaactaactcaaaaccccacaatgtccaaaac
ctctcgctcatctccatacctttaatctcatgaaaatggcgatgacccaattttctttcagtcgactcgggtttctgttcttcgtgttcttgatcttcgcttactc
ttgagattgtttcagcaaatggggttgtcgttgttttccattttacaatcatctgttgtagaaagctaatttgaaataattccactggaagtttgaagatcgtct
tggagcttgaggaatgagtggttcgagaatccatggatgggcttgaaggattgcggtttgtctttgtcctggttttcttctttctgaagtttgaatgtttcga
tcaattgttccccctttgtgtttttcttttttttgtttattttctcctcaaggactcgcagattggttgaacttggaccttagatcttgatgggtcgtttttttttagtatg
attattggtagaagcaacagagggagttatagagagttatgttctggtgtctggatttttcactgttctaatgctttattgattgtgtacttcagttcatgtatt
cattatagtgttgaacagttgttactggtatattcagaacattgaaacatttcataaactagtttcaaatgttaagatattattgttgggggttttggaactgtt
cttgcaggctaggaagctttcagcaagtgtttactacttggctggtggtctgtgatctagtggaaaatccaacatgcaacaattatcaaactctttgcgat
tactaataagttggataaggatgataagcatcataggctttttcttattgaaaccgattggctgctttataattcattatgggttactaagaaacaaatgttg
gatgcctaatactgacatgtttgttaggcatgtgaatataatcctacattggttagaggtagaaaaattcatgtcgtacgcgtaaggatgagatattgatt
ggtacaagactatttggatagaagcaattaataaatgtatgtggacttagaccaaaaacggacaatatcaaatatcatgatatcgttgagatatagaga
gacccattgtcctaatagtgattgtgccattatacaaattgatgtagcaataggtgtttggaagattagaaagggacatttcttattttgtgatcctccatga
atataatggtatggcattttctcacatcttctttgattatttgtcatcttatataaaattttatatattgtgggtcttgccatgctttgtgtgatccaaagcgctgg
aatcttggtgtgacactttttagactgttaaataccttttcttgtcttgagctgaaaagactaatatgtcatcaatttcagttgtgaacattgagcaatacaatt
atattctctgtgtattataggtatggagttgaacctgatttgaccataaccaatgtagaaaagccattgcttaagcatctaccagtcatgactctttattggg
ctctttcaagcttgtaattcctaatgctattggatacagcagtttgggaaataattatttggtcatcaaagttttcttgaatattcgtaggcttctcatttctgac
aagaacataactactcgtacatt

>LcWRKY28 + Up_Stream_Len 2000

Tccatggttgccctgatcttgaaatgataaatatagcatattgtcgagacatcaccgacgcgtcgttttcatccttacgaaaatgctcaaggctgaagac
aatcgaagctcgggggtgcccgcatatcacatcttgtggcttagcaatagctgttgcaggatgcaagctactaaggagattggacttaaaaaagtgtt
gcaatgttgatgatgctggcatgatccaccttgctcatttctctcaaaacctcagacaggtgacaattgctctcttttcactatttcttagagttaaacaggt
agaacaaaatgtaacttctctaatttcttggcagataaatttgtcgtatagctcggttaccgatctcggcctcttgtctcttgccagcctttgctgccttcag
catctgactatattgcacacaaatagattaactccaagtggagttgcagctgccttgttggcaaatagttccttaacaaaagtgaagctccatgcattgtt
tcaagctctgctacctgaacgtcttcttcaacacttagaagcacggggatgtacattcgaatggagagagaaaatatttcaggtacctgacaatgcattt
tgatttcttcgtcacatatcgataccttaggccttatttgacattcatttgattttttttttaagtttccaaaactagacatgaatttcaaaaacatttttaaggagt
agataacaaaacaaagaaacccataggcaggttaatttttaaaaaccaaatagttataaaatggggtttcgtttttattttcggagtcgactgtgggaaca
tgatacttttgccccccaaaaaggaaacattctcggactcgaatcgatgttaagctattaaagagagtcctactttttttggttgcatacaaggagtgggg
agcctggatcaatgtttaacttttgacaatcttgtgcagatggatatagtacacatatactatatgtatgtactcctatggaatcctaagtagctttttacatg
attggcaggctgaattggatcccaagtgctggaaattgcagttggaagatgagatgccaattgtccaataggaaccagccatctcatatatgtagaga
tctctaacaagttgagaagcctctctttctggtgaattcaaaattttcatcagctctgctccaaatgggaatgctttttttctctcagtttttgagtttagacaat
gtatagaatgaagctgtgtgagtttttcttctcaaaccttgaaaaagaggaacgtggctcattccaaaactggatgggcccacagaaaaacaactctg
gaaaagtagtgggctattgttttaggcccaaaggctactgtcttattggcaggacagtatttttgaattggatcaacagagtccatttattacaaaacttat
gtttttgtagttctattttaggaaatgtctggtttttctgattccaaaagttgggataatttaatattcacttattgcatagcttagaataaatgtttcaagtactat
tattcactaacgaaggattaaaaagaaaaagaaaaaccatatgttataatagagggtgataagattccataagatgccttgttccactgcagtccacgt
atacagaggtgggacacgtggcatgatctcagcagcataaaagctaagccgccttctgagaagagcactgatcagtccagtcaaacccacaagctt

tgaccaaaaagaagaaaattccacagtcaaacttccatgtcagcttcgaagaaagtagctccacgaaacttaattgccaacaaaaacaaaaccagtc
ctttgttcctcttcgttccctcgttcttctccaatttttccctctctctctctctctctcttttcttgttcttctttttccttgttaatataaataagccctcattctgaa
acttctttcttcacaacttcttccagtgacttcccttttcccaacaacgaaaaagaaaaactaac

>LcWRKY29 - Up_Stream_Len 2000

tcacatctctccatgatcacaatggatcagttcccctcccctggtgcctttgcctgtattgggttgggatagggagcatttggaattaactcgatcttcata
aaatcatttgttgctgactaattcgacctcttcttgcaactggcagtggcgttttggtatggaggggtctgcgtcttcttttcctccacatgatcactggtaa
ttgttcctaaaagtgtaattttaaggcaacttttatgaaatcaacctttttgggatgaaattaaaattaatggatgtttacattatgtccaaagggggtgatag
atcaaacgatcgacttttgacatagtaattgataccttatctacttaaatatattcatattaacaatattgttaaattttatttttataacaaattaaaagtttgtac
cacttcatagatggattgtttaatactgtcaaactttttttgtaagaaaaaaacaacaataatcaaatcaaaagcttttattcttcttggtttctttttccacatca
cgtgtgaaagcgttctccttttcaactttcaatcccattcattcacactgtttttattattattgttattattattattttgaagggtgtttttattatatttttggtctgt
tcacttattagattttatatgccaactgctcgaaatctatttcccggttttattattattattactattttaatttaactaaaccatttctttatttttgactcgttccgtt
gggaaataagctagtttcttaatggattaaattaaaagagtagtcttcaagcttttgaatttgtgtttatagtgtcttaaatttctaaaacatgaatagatatct
tttttttttctaactcaaaatatgaacatatatctaaactttcaattttgtgtctaatataggttattgactttagatttatttaacaagtttgtgaattttcaattttttt
cctacgtatctttaacatatttaacatttttttaaaattaataaatctattcaatataaaatttatttttttgtatttcataaatcctaaattttcaattttgtgtttaatcga
taaataaatttaaaaaaatagctaatagactaagagtctatgagacgcaaaattaaaagtttaacgatttattagacataacattgaaagtttagagatgtac
taaacatcttttaaaattgaaaagaactttttaaatgcaaaattaaaatgtttaaaattttattagatgttccttaaaagttgaaaatcttattaaacatacctttta
aaaatatggaatttaagcttgtcaatcataggatggaagataggtgcgtaatccgaaacgattcggacatagaaatacaacgttggcactccacaaga
aggaaatagagaaggatttctatgatgccatgtcatcttggtaatggtattgtcctttgtaaattaaaagtttttttaaaattcttttgcagatggctggccctg
gcctgataatattattaggataaaaaaccaaaaggtgccctcaatctccgagagaatggtatggcgtggggagggtattttcgtgattttgtttgaagca
aagagatgagaagaaagaaatatatatatatatatatatatatttaaaaaaaaaaaaaaaaaaaaaaaaaaaaagaaaaggcgacattggaaatattaaag
taaaggcaactggagggtgacgagggtggcctgacctcaccagccttacctcagtcctcatcaccattgtttagtccactcgcgcgttgttccgttcct
ccaatcaaagccgtccatcacgcgcgtgcattccaaacataggtaaaggcacgttggttttacatttcctctttgtgtggaaaatttgcagtgggagtttc
cttatatcttccttgtatttgcattgaaatgaagacaaaatacaattgcctctcttccatccacataagaaggcgttggtgtcgtggtagagagtgcagca
agcgagagagat

>LcWRKY30 - Up_Stream_Len 2000

tatgaactcatatttgtttctactttttaagtatttgagtacatgtagggccttctcataactggctcctttcattatctgttgggcaattgtgaagctccacaaa
gtaaagtgaaaaaaaaaaaaagaaaaaagaaaaaaaaaaatagtagaattttgagggcacagaaatttggtgcacccatagaacatgagagttgttaa
ggaaagagctcactgtcgttagaaaaacagaacagagactgagtatagtgtcgtgtcttgaagtgaattgtcgtgtatagttgaccttcaattaaacagt
gaaatttccactaagcccaatgagacagagtttttatgcttttgaggtgtgggaaaaacagtgacagtgaggctccaaataatatccaaagcatcaact
tcagttttaatcctcaaaacagtcacctctcactacattctcttctctttctttcactttatttactttctttttgactccaaatttgttactattttttttgttttcttgttttt
gttgaatagtgaatcaaagaatgtttttcttatggtcttgctttggacatttatcttctaatgtccactgtcccttgtgttcttttagttatatataatctaaaacca
acaaacaagtgaattacaagtactgggtcttggtgatttgagacactatgtttaggaaagcttttgagatcaggaaatcaatacaataagtactttcaaat
gaagagagaatatttgaatttagaatatctgccttcatacaaaatatgttaatagctaaagttgataaatttggagtaaatttaaggtttaaatattattttggt
accagtacttttggtttcaattcattttggtacttgtgctttcaaaatatccattttgaatcctatactttcatttttgttttattttggtccatgtacttttaaaagatg
actaatttggtctcttcattttcaatttctttctcatttttttctatcataattttaagatgacacttcatcacttactaaatttcttgaaatgtagtaataaatttgatt
aaagagcttccatggtgtataaaatttatacttaaattttacaaataggggctagattggttacttctttagaagtataggtcctgattaaaatgaacaaact
gtcaaaagtacaaagaccaaaatgaagccaaaaagtacatggaccaaaatagtttttgaacaaaaatttaattactctgaatatataatctaaaggaaa
actttgggttgtcatggaaaatgggccaagtaaagaaagtatagtgtttttatataaaattttacatgaaactttgaaataattatattagttacaacatgata
caagtaatttggtacaaaatgcttggaaagtatagtgtttttagatcaaatgtttttggcttttaatttctaaatggttttgctatgctaagagatcatattttgta
attgacagagttcttacataaaataaggcaaaacaaatacaaggagagcattgcatgtagacttgtgtagaaggaaaaagaaaatgcaaggtgtaat
aacaaataaggtaagtattcccaacgatataaagaatgaaaatcagtgatttaagtaattaaaagaaataaagcacagaattaagataatgatttaatgg
tcaactttagtactctacctcgaaattctgaccagttaaaaaagggaaaagaagaagaagaagagtaaaaaaaacatggcaaaatggaaaaagggt
aaagtagttttggtgttgggatacgtaaaggaagcatctagctattctagtatatataaaatagccacccaaattccttttgtcaccctattcttcaacttctc
aacccatcaccaaaaaaaaaaaaaaaaaaatctctctttctcatccttcaaatccaccctctttcccttttcccacatcggaaacaaggaggagaaaagc
catagccaaaagacaacgagagaagaagattagagaaaa

>LcWRKY31 + Up_Stream_Len 2000

aattaactctaattttccattctatttttaaatcaaaataatttttttaggatccatttcctatacaatttggatttctttctttagtttataatgaatatctatttggaa
aatttatttttgcgctataatattttatgttataaactcatttaatttttttatcaccattaaaatatattataaattacataatattgaaaaataatgattataccaaat
ttataatttaaaacatattcataaattaactaatgataatttagttttaactaaatatttagttgctaaagtgagcataactcaactctaattgacacgtaccctc
aactatgaggttcaaggtttgaatccgtcaccctcaaatgttatcaaacttatacgtgtgtatatatatttatttggtaactacaactattttaattgtttataaat
atattatcaaatactctttcaacaatttttttaaattgagattcaatttttgagtaggctacttaacacattaagaaaattgtctaaattaatgtgagacgtcaaa
ctctcacaatactaaaaataaaatgttgtatcataaaatattcatttgtcacatctataaatacttgggaaaaaatcatttttaaccctgaactttgggggttat
atcaattttaaccttaaactttcaatttcatcaaattgaactctgaacttaacaagttgtatcaattttaaccttgaactttcaatttcatccaattgaacctcaa

acttattaggttgtatcgactttaaccccaaacttgcataagtgttacaatgaatacctttggttaactttccgtttgaaccaccgtacgtaattgattatttctt
cttttttttttcttaattcacattgcacctgaattggtttcttataaataaaatattgaagaaactttattttagatgactttttcttgttgttgagaactttgtgagg
gctttttgctgttatctaagattacgaaagcaaagaaactttggaggttttttatcatcaaatatttgctggtttttcttgttgctaggaactttgtgggattttt
ttccttcttttactgcttttttttccttgctaattagaagaatgtacaaatttagaaaacggaaataaagaaatttcaagtttttttatttctcatatttgctggatat
ttcttgttgccaataactgtgattttttttttttgtgaagaatagtctaggatacttgatttcttaaagagtttttcgaacaaatttcttacaaaacactaactcag
gtgcaatgtgaattaaaaaaaaaaaaaaaagatattgtcaattacatgttttaatagcaattcaaatgaaaagttgaccaaaggtctacattgctacattta
tgcaagttcgaggttcaaattgatacaatttgttaagtttgaagttcaatttgataaaattgaaagtttagggttaaaattgatacaacctgacaagttcagg
agtcaaaattgattttttccgtaaatacttcgatttagtttgatttgacaagctcttcttttaattattgaagtataatcgtactaattcacacccaaaggagga
aaaaaaaaaaagaaaaagaggaaaccaaatgatttaaaccaaacgaattcaactttaaaaacatagaaaccaaattttaatttttaacatataaagaaa
gaagtacttaaagaaaaaattcgttaattaaaaaaagaaaagtggtccataacagtacaataaagtgtacaccatacgtcacgatggtgacatcacca
tactcactccaaatccgccacgtgtcccaatgaagaactttcaaagcatcttcgtagtaggaagctgccatgaaagagagagaaagagaaaaaaata
tatttttaaaaaaagtagattaatcaattaatatctcctattataaaaactcatccagaccttacaaccagctgcctgtttcacaccaggtccatagtttccc
a

>LcWRKY32 - Up_Stream_Len 2000

Tcttttgtttttaattaaaacaaataggataagatttttttttttaattttttttctttttattttttttaattaaaacaattacgaaatttaatttccacttcttgattttttt
atcttttattttcttaattaaaaaaattatattttaactaaattaatttgtcttaaatttatttttaattattaaaacaatttatataaaatttatttttttttattttttataaaa
acaaaaaggataagattttttttattttttatcttttattttttttaattaaaacaattaggaaatttgatttccactttttttattttttattttaattaaaacaattacgaaattt
gatttccactttttttatttttttatcttttatttttttaaattaaaaaaaattagattttaactaaagtaattatcttaaatttaaaggagtattttagtttttttttttaattttct
taatcaattattatagataattattaaaatcactatgtttttttttcttatttttcttataatggtttttaaatttggtagggattacagatctataagctttaatattt
tggctttaatggtaacttaatataatatcaaattaggttatctcatattcaaactcttttgatgtgatataatatatataaaattataaatacatgtagcaagaat
tttaaaattttacaaaaataatataatcaaactcaatttaaatttcaaatttaacattgattgcagtaagaaagattaaatttatgctatattcataaaaaatta
aatttatgctctattcaatttaaatttcaaactaaaaaaaatacaaacgaacaattggaagaaattgaaaaatacctgaaccaacagatttttttttttttaaa
aaaagaaccaacagaattcgtgtagatttttttataagaattttttttttccaaaatttcataatgtggtttcctatttgtttttattaaaaaaaaaggaaaattaaa
aaaaaaaaaaaaaaaaaaaaaaaaaaaaaaaaaaagaaccaacagaattcgtgtagattttttttaagaaaagtttttttctcaaaatttcataatgtgg
tttttaaatgtggtagggattacagatttgataattgttttaattaaaaaaacaaaagataaaaaaatttaaaaaaaaactaccctatttgttttttttaaaaaag
aaaaaaaaaaccaaccgtgtagatttttataagaatttttttttttcaaaatttcataatgtggtttcctatttgtttttattaaaaaaaatttaaaaaaaaaaac
caacagaatttttgtagatttttataagaaaagtttttttttttcaaaatttcataatgtggtttttaaatatggtagggattacatattttttttataattttcttaat
catttattatagattattattaaatttactacgtttgtttttttttccttatttttcttttttatagtaaaatattttttttcaaaatttcatattcgttttaaattttttttttt
acagattaccagtttttaatttttgtatcttttatttttttaattaaaataattagagatttgaactaaagtaattatcttaaatttaaagggggtattttagtctaaaa
atgtttttcacatatccttgctttttaatatatattaattaataattaataatatattaatatattatagatatatagtaagatatatatattgcttaaaaaaaaataa
aataaacataaaataattttctaaaagaaatggtagtatgagttatttttattattaaaaattgtcttttgaagtccacaaagatgaaaaaaaaggtgggttgt
tatgtatattgattaggtcatacctgaattttctctaataaccactcaaattgaaggttaaaaatagagagaacgaatccatagaagattgtggaatgaag
ttttcctctgagaaagaaaaaaaaacagagagcgc

>LcWRKY33 + Up_Stream_Len 2000

gttatgggttattttggactacttcgatgtataaggagctgacgaggacaaccccgacacgagccagaaaataggaccagaaaactgactccggagg
tgaaacaggccagagggtcgggccaaggtcggagggactgggccgaggtcgaggccgaccactcgagccactcgcgtgggccgagtcctttc
gcctccatccggtccctggtgcccttggccgccccagttccacttggttcagtctgaatcgcctccgaatgtcaaaaaaccctagaggaggaacatgt
atttaaatccttcttcaccactgaagaaagggatcccgaactctattctctaactctcctcttactcttgctctcttgcttcccattgttctacttgctgacttaa
gcatcggaggcagtgtgacaaacaccacaccggtgtgcaggtttattgctttgtaggccatgtcttccccccctcttatacaaatttactgtcggtggcac
gtaaaggtcagatgagttctgtctgttcagattttgccatcaacataaaacatttacaattatttattataatttttttttttacatatatggtgacaatggtttaaa
aaaaatcataactcaataaaatgtttatgaatatgtatctttataaaattaattagaaattaagcataatttaaacaaatagttataaacataggatctttttaa
aaagaaaaatgtactaattatttattgcttaaataataataaatcaatttgcttttatttttatatgatagcccttcataaaccaaaattagttataatctaggaat
attcatcctgacaatcaagagttaacataatattataaaaagataacaagaaaaagaaaaagcaagcaacctatatattgatttaagccctttgttaataa
ttaatatattgatcaaccaacataatgattaagttaaataatatgtttgagtccatttattccttgtacttgaacaacttcataaagtttcaaatttgtgtttattta
atatacatactttaaaaacattctaattcacttgcaataaactaaatttagatctcctcaaaaaaaaaagaaaaaaaacaaactaaatttagatagaatatt
aacgaagctgaaaataattaaaattacaaggtaatccagttatttgcaatcaataaatacataatctaattaccatttttttttcacaaacgggtgagagat
ttgatcttctaacctcccggtgaagaaacttatgtcggttgaactatgcttttgttaactaccacttcttgttctatatttaattaatattgaaattaatcatccca
cgtaggttaattaataactatcctacaaagtacaatttattatttttaaaaatatgctaggttagttaaaaaaaaaaaaaaaacaaatacttggaattagtttt
aagaaaaatggtttttataaaataaattaaaaagttcaaattaacatttctttaaaagaaatataatgaaaaaaaaaaactaaagaaaagcatatacataat
ttaaaaaaaaaaattaataggaggacatatccttaattctaagttcaaaatttctcaatttgaaacaacaattcaatccaacctcaaaatatatatttttttaat
tttctctttcatttaaagatatagaagatagaatgttctaccacaaaatattaaaaaatatatatatcataactcaaataaaaaatatctcccacatcttaaaa
aaagagaaaaaaagagaaaaaaaagaaaaaggaaaaagaaaaggtaaagttttaataaagggccccatccccagccccaggaggcagcagag
caatggtcaaatgagaagaccagacagagaggatcaatccagtgttgtttttaagaaccccttttcttctttttgtttgtttatattaatgccctttttctcttct

ccatttcattcaatttatttccattttccattttcccaattccccccttcccttcc
>LcWRKY34 + Up_Stream_Len 2000
ttggacacattattattattattatttattctatattattctatattatatgattcttccttttcttcatgaatcatgatgatcatattagataccatgtttctatcatctc
ccccttttcccatttctccttttttcttttaattaaatatccttctaatgttttatctacatcaatcccgagaagctacactatgtttttactcagataaccatatattt
aaatctaaacttgtatggtattaaagttttgatataagattgaatttatcgtaaccctatctgcttaagcgtccggtggtgctattttattgaggtaagaatatg
gtttgtatggctagtagttttggtatttttcactatatttgatgtgtagagttgttgggatatattaatactttgatttggtgtgtttactgattggatctgcaaatg
gcaacacgaaacaacatagatctaactatgaatctatgatatagaaggtgtttatgagaatgccgccatattatttgattcatatcatttcttcactttaattt
cataaagggcatccattgtgtccttttcatgtcatatcaatttggtcttgagcttgtacctttttgtagtacacaatcccttattcattccctccattttattacct
caaatgaatgccgttgcaagagtgaaagttggaggagtgaaagagagtgcacgcgagatagatctagaatcccattttcaataacttttcttttctttcc
ctccaaattccaattcaatctagccatgattgttttcgtcttcaagatttataacaaaccgcttttaagtcttaaaaaagcttttcctcttctactaataaaatac
gcaacacatcacgaaaacgaaaagcaaagaatcacagtcaatctgagcataatttagaattgtgaaacaccgatctgaagattcgaagttcaaatttt
cacactcacatacttaaacgtgaaaagaaaaagagacgggtcatagcaatgtactacgacaaagcaaaatgcacataaataaccacaaattcatag
caatgatgagtgctgcaacaaataaaagttggaaaaaagtacaaaaatgtaaggaagaatgcagagagtttcaggcctaaagattcaatacgaatga
tcaaatttgatcaaagtccaatccatgagcgtgacagaggaacagtttcattcatctatattctttagtttgcaaaccatagacttcaaaaaaaaaaatgta
atcttttttttaacttatttttttttcttgaaaaataattgaacttaaaaaagaaacagagaaattagaatctcatggacgtctagcggctttataggtaaatga
cacattgaaatggcagtgacacatctctgcctaactctgatattttagtattgcaaaagcagaaaattttgacccctaaaccatattcctatttcactattat
gacttctcagaaaatcttcggcttaattcgttaactatcgatatcgatgataatatcgaaatattcattagtgacaatattgaaaatgtgcaatatatatagg
cagagggtatggaaattataaaattcttttacattttttatgatttggttggtatattacaccccttttaaattggttttaaaaacaatggaagctcaattatttaaa
ttgtgtacaaactaatatgtttgacccaataaagttctttttgttaatattatttccattaaattaaaattaaagaaaacatcatattttattttttaaaaaaatctca
cctttagaaagtaagaagtagatctagtaagcaaaaacccaccatccaatttaagacacatctctacttcatccctttgtctctataataggacatagatcta
agggcaaagttcactttttttacacacaccccaaaagaagaagaagcagaagaagaagaagaagaagcaaaggatctaaattcagcatctcatctttc
tgctttggtcttctaattccttttttcca
>LcWRKY35 + Up_Stream_Len 2000
aaagatttctcagtgttgcagctcctgcaactcatcctatccaggccagcaggcaagacatttctcagttgcagcatgcacccccgaacgccgaaac
aggtcttcttttacgggcggtagatttgacgcgacacggttccacgacgaaggcttgtgtgttgatgcaaatggacacatgtttgaaattttgaacttgg
tattactgctgctgcttctttgagtagaattaggagtgagtttcaagtttttgactttgtccactttcaacatttctgttcattgagtgaattacaaaatgaaga
aaactttccctcagggtgttggatcattttcttttgcagtcaagtccagaagtttatttatggattggtccccccttcaatatcattttataatgatcatcattttga
tttaaaatttaattttgttctgacaatttaaatggtgtgtttgagttgtcaaggattgtcttattctgtttcttgattttcatatttcttggtgttcgaattttagaataa
tatggttagttatcaactaagcagtgctttattggtatatatatttgttgaattttagtgaaatgttaaaaaaataaataaaaagaaagtagctttaaaattttgt
ttttttgaaaatggatgtacgaaataaataaattatcagcaaattgtcttattttttgggaaaaagaatccattagtttatgttggtcaaacatacataactttc
aaatcgattcacctgcagggacgagatgaagccacgtggaagcaagcggtgctgcttgccttccttttttttttaattcaataatatatttagagggacg
ggtggttttcctctaattaagtgcattaaaataacataatatatcgaaatatatatcatttcgatgataattaacatgtatatttgataaagaggtcagaagttt
aaattttgtcaacttcaaatgttgtttaactcaaaaaaataatgaataagacatctattatcattttgaaagtagataataataaaaaataaaaaaaaaaaaa
aaaacaacaacaacaattaattataaaaatttggttcttcaattttttttaattttttttttaatttttttcaatatatatatatatatatatatatatatatatattttattta
tcaatactaggttcaagaaaaggaaaattgagtgatacctttttagtctataagatttaagataagtttctattttatttcaaaaaaaaaaaaaaaaaaaaaag
acaagtttctatttaatctttaaattttaaaccctctagatcctaaatttgagatagattaaaagcatgatattgaatatttaaaattaaattaaacgttgaagg
ggtaaaatacttatttttataatattatttaagattatgtggcaaaagtatgatcttacctctcctctttgcttcttacttagctctgtcttaactcttgtcttaatg
actcttcctctcttttcttggtattttattttattattttttgttacttcttatcacaaatcagctcaatgaataagatatttattactatcacaaaaattaaggaacaat
taaactatgacaacatgtgagagtggagaactcgaaccacatgccttctggtcattaatacaagaattatgtcagttaaactatgctcatgttggcaggt
gtctattatatcgtatgtcatcaatttgattataacaaaatgcaattttctttgtaatggtatcatcatcatcatcttgaggctcaagcaggctccggtcaaag
tcagagcatttgaccaagacgaaattcaaaaataaaaataaaataaaattcaatataaacaagttcaaaaaatttcttccccacgcggtccaactccaa
atccccacctaaacttctcagaaatctccttcccttctccaaatttgttttctttttacctttctgtaatgtgataacataatccactttcttcttcttgaatccaac
tgaacggtt
>LcWRKY36 + Up_Stream_Len 2000
atgatatgtttgcatgtgtttctattgttttttttttcacgtatatgttcaaatacatatacataagcaacaatacatttgcaaacccgtcattttctatctataaat
ttaacgaaaaaaatgtcttcaaaggttgttaacttttttttttttagtacatcaacatttgggggtgggggattcgaacctctgacctcatggtcgagagtacat
gtcaattaccgctgagctaagttcactttggccaaaggttgttaactaaaaccaacgtttcaggttttttgtttttttttagtacatcaacatttgaggatggg
gaatggtcgaaggtacatgtcaattaccgctgagctaagttcactttggccaaaggttgttaactaaaaccaacgtttcaggttttttgtttttttttagtac
atcaacatttgaggatggggaatggtcgaaggtacatgtcaattaccgctgagctaagctcgctttgacacgttttgggggtatttttgataatttaattg
atattttattaccgatactatggggtagtaaattagcaatatataaatcatagcataaaggaatgttagaagcggaagcttgtgcgttaccaaacaacgg
ccatagaatatgctcttagctgttatcaactttgccgggaacttcatcggacgcctctcagccggagcaccaatcactgcttctcaactgtaaccatttca
attccttctctctctctctctcttcttcttcatcttcctcacggcggcctctttctttttcctttttcttcatcggagcttgaatttgacttccatatctcgcactttc
cgctcctcctccactgacttcgctcctctctgcagctccgattcgccggctcgaattccgatgccggactcgtaattggcggccgcgactgttttctgg

cggaattctgtgcttccacgtgaaattactgcacctgtgtgcttttctagggcttgaaacttcgaggcttcggctctgcggtggaggtcgaggtcgagg
tctttttcctttctcgtgtttatttaacttttgaactcactataatcgttgtaatttcggaaaaattgtatgtgattatggctcttgacgaatatttttctttttaaacc
tatgttcgtatgttctgtttctgaaattgaagtcggaatgttgtaatttggatcttaatgattcagatcgttactggtgtctggagatctaatcactcagctcg
agctgagaatacttgaactgcatattttggatgaaattagtagcgaattgtatttctgctctttataagcttactaggagtgagagttttacttcgagtacga
tagtttttgttcgcctcgtgaatttcatcgaatggtgtgcaagtggattgttgtgcagtgaactcaatttccatgtatattcttctttttttataaaaatatcttg
gtcattaattgagaattaagattaactcgtaatttgaataatgtaaggaaaagtagtattctaaatctacactgacactgttgttcccatttttgggtccttgg
gctaaatggaaatttccactattcatgagtataaatacctttttatattcgaaacgagacattgcttgtcgttataatgcatgagtgtgagatgctgagtatg
tatgaaatcttcaaaccttctttttgactatggctctgaagtaaaattgaattgcttgttttaaaaagggtatccacaaattattttattttccattcacataatca
ttctcgaataatgataaattgtttgttactatctggcatctggattttcggttatatggaggcaaacaggtgcgaattgactttaagtaaaacaaaatttgttg
catttcttatcaggtttcagtatccagctggacttggcgtggcattcagttcctggatcaatcaaagtgtgaagaataagagttgtttagagctctctggct
tatgatctaagctcacttgtgcat

>LcWRKY37 + Up_Stream_Len 2000

Aatattttgtcgaagatataatataatgttgtaaccagttgtgctaatctttagttttgaagaagcacaaactttattagttatttaaacttaaagttacaaattt
gattcatggagctacctatatgatataaaagtaattttttttccccatagcttcaggatataatgaaaataatttacaactaaacaaaaaatatattttctctca
aaattttcatactactaattataagaaatatctaaagatcgctcaaaatgataaattattattattattactattattttgaaatgtaattcaatgaaaactttcaa
cactgttttaaagcatgcaagtatgtaatagaattaatcatcaaaattatggtttagttattttcgaaaatataaaaattatggcttattaattattatgttattatt
ttagttcaattatggcttgagaaagagacaaagttcataaacaaccaagtttcatcaaattttaatcaaagaatgattggttgatttgaaacaaaaaagaa
gagatttaaatttataccaaattcaatgtatacgcaactcaaaccaaaaggttacatcacattaacttggtattcgttcaatgtagtctaaaaatgaattaaa
gcagccaaaaatcaaacatcacaaattactaacaccaaacgaaacaaaaatatgaaatccatacaatttaggccccgtttgataaccatttggtttttgg
tttttggattttgaaaattatgcttgttttctcctaaatttcctaccatggttttcgttctagttaaggttgcatttgaattcctagccaaaattttaaaacaaagac
aagtttttaaaactatttttttcccaaaattggacttggtttttgaaaacaaagtagatgattcatactaaaatcaataaacttataggtggaagtatgtgtg
tataaacataattttcaaaaaccaaatggttatcgaacggagcctaaattttataatttccaaattacaatttcatctatgtaattcaataaaaattctgtggg
agaatgtgaccaaagtcccacatttgctagacaagggaaagaccatggatatataagagaggacaagttctctgttggtatgagacattttggggtga
accaaaaacaatatgcccaaagtggacaatatcataccatcgtggagatttatgtcgtgtcctcgttccttaacaaataactaacatagggtgtgtttaat
ttaatttttttaaaaaatcattttgaaaaaaacattatcagaacacgacacattagtaacactaggaacgacgatatttcacaacgtttgaccaaattgaaa
atcatgatcataccaaaaggaaaccatatcataccctcaactaaaagatcagaagttcaaatctccgattaagatatcaaaattaggagacccaatgaa
aaactctaaaaccatatagaaaagatctataatatattcacctcaaaagaaaattaggccaaaatgttagaagagttgaaaaaggagagagagggag
agagggtccatcttttttttgtttggtccaatgaatgtgttttgtttgaattaaaatttcttccacataacacccaggcacataagataagaaaaattggtttt
gtttttggtgagatttgaaagaaaaagaaggaagactagtgggatatttaaattgaaaatatatagaaataaaaaaggaataattttgaagatgtagtttt
gaagtagtgtttggtcttgtttgtcaagtcttaccctccaaaattatagccgagtccattgaaaccgtttgaaagaggtgtgtgttaaattaaaaattaaaa
gtgtttcggatttatcattttatggccaaacgaagatcaagtggactctcctttgattcacaatcagatctccattttctttctttctttctttcactttcatttccct
taaccctactgatcaactctcttccgcctccgcctccgcc

>LcWRKY38 - Up_Stream_Len 2000

taaaacatcatttttcatcttaaagatttatggtttgatcctctacccacaattattgttggaaaaaagaaaaaaaaaatcaaaagacaggccccgtttgat
aaccatttcgtttttgtttttggtttttgaaaattgtgtttgttttctctcaatcttcctacaatggttttcatatttgttaaggagacatttgagttcctagccaaat
tccaaaaacaaaaacaagtttttgaaaactacttttttttttttcaaaattttgcttggtttttgaaaacatgggaaatgatgtagataaaaaacaaagaagct
tgtgagtaaaaataatgtttatagacttaattttcaaaaacaaaaaacaaaatggttatcaaatggggcctaaataaacaaataattgaacttttattttttaa
aaaatcaaataacaataaaatcaggtccctcatccttatcgcctttacctaaccatcttacctttgagattttgattcctgcatggagcaacaacgccgca
aatcagtgaattgctctcaacgcttgaacaagtctatatatatatatatatatatatatatatatatatatatatttgaaataaaagttatatatatatatatatt
tttgagttgaagaaatagtctctaaaataaacagataaaacttttaccaaataacaagaaaaaaactattccctattaattagcccataaaaaaaaagtaatta
ttcgaatacatattcaattatttaaattaaaactctccgtcacggtgtgaactgtgagagtgtgtaatccggattccgattaggtttcaaactggaaaagga
aattaacacaatacgacaaacaaattaagtagacaaatacaataatacaaaaaccctttgcttttgtttttttattctgatggaatacttgatccatttgttaaa
ttacagttttagtccttttaattttgtgtctacaaagtcttcaaactttaaaagaattgtccaattaatttttgaactttcaactttgtgtctaaccgatctctaccc
tgcataaatattagtctctaaactttaagttattgttatattcaaatttctaaaaattaactaatctattaaatataaaattagattatatgtctaataaaaccataa
actttcaagtaaaggtggtgaataaatcaattacctaactagacacgagattgatgttattaaatactctatggagttcaataagttgttaataataaaattg
aaagttcaaagatttattggacactttataaaatttaaggactgaatggatacaaatctgaaacttcagtgactaaggtcccgtttgataaccatttggtttt
ttgtttttgtttctgaaaattatgcttgttttctcctaaattccctaccataaatttcatccttgttaagaatccatttgaattcctagtcaaattcgaaaaacaaa
aataagtttttgaaaactatatatatattttttgttttcaaaattggacttggtttttgaaaacaagaaaggaaattgataataaaacaaaaaaacttataggt
ggaaatagatgtgtataagttaattttcaaaaaccaaaaaccaaaaaccaaatggttatcaaatgagacataaactttaatttaacctaatctttctaataat
tccggaactccacaaaaccttacagtgcagccaaccgtacggaaattaaaccctcacccttaatcctcaactaaaacattataatttagtgcgtatttgc
ttgcacgtccttaataaacaaacatagtttgccaatctgtcaccatgactactctttgtattaaacctcatctaattctctcactcctcccctctcccaatctct
ccggccaaatcccaactccctttctctctctaatctcagccgtacgattgatcctcatcacacaatttccggccaccggagtctctcaatctcatccactt
ttcatcgccgttccgccc

>LcWRKY39 + Up_Stream_Len 2000

tcaggtgatctcaaattacgactatatttttattttctacttttatgaacggagtattaatcattcacctaataatcataaaaatagctatattcttctaaaagtga
atcataccatagatatatatatcatttaatcatatcaaatgccataaaaaaaaacaaaactcgttgatccatatcaattcttgattaatattaacattagatttc
ttgaacactaattaagctccctcaaatcaaatgaagaaacataatctaacaataatattctaaatagttgattaggtctatttagtcatgtccaatcaaagtc
aacttgattaggagcccttctttttcttatccctccttttccctctttcttttcatgtttggtttaaatttaaaatattataataaatcataaatctcgggcttggaat
attatttatagcagtcacaataatttgttttctagcaatatttccctagctacacaatacatatataacttatatatacaaaaaaaaaaagaatgaagctttttt
agatatatagagacataaatagaggtatactaaagtaatttggagaaaccttttaaaatcaaaacccgacaagaaaattattgaataaaagctaatgaa
atataaaaaaagtttaaattacggagcgagaataatgggaagtgatttaaatttatgagtttttttttttttttttttccttgaaaaaaaaattagggttttttttat
gactactgagatgacgtgagggttggatcagcaaaggaagaacaccacacagagttttggtgagaaaggaaataaatgaggatgataaaataaaa
ataaatgaaataaatagaatcaactcccacgtgccgagacaacggtcaaacgccggtgccggtttcgtcacacgtgtcgggacataacccttgtga
gagaagaaaatggaaggaattttagtacattataagttggaattgttgactaaaaataatattagtaattacttccatactaacgactgcgactctcatttttt
ccattttcaaaatcccgttttcatatataaccttctttgtttctttcacactttttttcttcttttggaattataccatttaccgcttggttgatggtcaaatttcgtaa
ctgcctaacttccacgtgacactagtagtgaatttgttaaagagagggggaagactgacacatgcaaacactgcacaccgacgtggtgtagacaatc
tataaccgtaggtctcggagttgtacctgtgattttagtgctctgaatttcgatccccccttctcgagtgacctctgccggtatttattcttcttccccattagg
gtttacttcggagctccgtgagactgcccaggatcaacgtataacgagccccgggccttcacatatgtcgagcccgagcctctcctcggcctcggta
atgggccgaggccgaggaacataacatgggggggcacagactcctttatctttccctaactgtattgggccgctccttctgtattcgggtccgttaattct
ggatctactgaaccgaatgattgctggtgattttttactcataacaccttttatcaaaggtttttactgcaattcctacgattaattaaatattaaatgttgtaat
tgtagttggttgagacttttagagagattaacttaatgggtatttagtattttaactgttgataaattgacccagtaaaaaaaaaaaaaaaagaaaaaaaga
aaatagccgatatcttctagatctaaagcatgtaattatttaaataatataacacaatattttaaggggctgtcaattcatgtgtaatgaattaacaaagaaa
aaaaaaaaagcgagtggagagtagccaaaagaaaaactctcaaacggcggagaaaagagaaaagaaaaccaggaaagtttggggtttttttttgca
gggatctaaaaaccctaatttgggtttgatcaaattggatatctttgttca

>LcWRKY40 + Up_Stream_Len 2000

gaaataaaaattgataatttcatagcgggttcggaatacctttagaaaagattgtttttctaaacaaaattaatgtttttataaatattttttaaaaaaatttaattt
tatgtctaatagattccaaaattttaaatatgtctaataagtttctagactttcgattttatatataataagtcattgaactttcaattgtaacaacttcctagcgc
gagtggcgacaggtctgaagccgacgtcgtcggaatcgtccgtcgttgacgatgatttagggatttttttataactcttccacgctggaagaggaagtt
ttttttggagggatgagtcttttccctcgtgtctggcctctctcgttgaggatgatacaattgtaactaccattttctaattaatgcagttattccagtttcaaa
aaaaaaaaaatcataaaatatgtgcttataatcaagaggtgatcatgaattcgaatcctccatctgttgaactaaaaaacaaatagataatcaatttgaaga
gagatatcatttgcatgagaataaatgtgttaacattaaaaagtacgtacaaacgtgccaaagtgagtataacttaacggtaattgacatgtacggaga
agaggttcaaatccccccacaccaatattgttgaactaaaaaaaagtaggtacaaatgtggttcatgctttcttagccaaccacgtgtttcacccatacaa
aactttattgagaaaaccaaaaaactcccacgccaatgactcaaaacacagatttttgaaaataaacatgaaaaatcacagttgtatttttatgtttttagt
gcttttggatgtaaccaattttagagattgaattctaattctaaatgtatttaataatacatttataaatcataaaaatagttatagttatcaacgtggaatttag
attattgtggaatttaaattatcataaattaatttatacacatgtcttatgttatggattttatataattattattttgcaatatcatataatttataatatactacatta
tacatttttttttaatttaaaaaaaaaatgaaatgattttataatatgaaatactatacttaacaaagtttttacctttttttaaatgttgcattttaaaatttaaaattcaa
aatttggatacactaaaaacataaaattgttgttttaagactttccactactttggatcttggaaatctaattaatatacagttttaagagacaaatctataaaa
aagaatatatttgttgaaaacataaaacataatatagatcgaaagagacaaatttgaggctgctttttctttacatgtcaccaaaggagataaaatagggt
ttgtaaataattgaaaagcttaacaaatgaataacacaacaaaagaataaacataaatggaaggcagctagggagacgattcaaaccaaacattctcga
gttttattagatttatatatagatacggatacctcttaattgtcaatattttaatagagtacgtagagaaattataatcggattaagtatcgtatatttgttattcg
ttgagatgtcagaatgtgactgaataatttaaacgatgtgatttatcttcgctatttactaaatgatgcgaattggtttttatcttaggttttaccaattgcatac
gaaagggatccttaattttatgtgataattaacttgatttccatgtttcaatgagtttatgaatttaatctcgagagagagagagagagagagagaaggtggg
tttgaaattgaaaaggagaatgatgatgataaagccgccagccaggcagcattatttccactaagtgacgaaaaccccattggtttgtttgaccaaatt
aaaaccaaaatctgacatttttcgtgccgtctacgacacatactcttcgcaaaatcacttataatttatatataattcctcaaggacgaccctcttaaataaa
ttgaaattctatattattattctctgatctgta

>LcWRKY41 + Up_Stream_Len 2000

Aacattcatgttgttacaatatctgaagaggcaaatagttgacattcaatttaaatggtatatgtcaataacaaaaggagttgactattgaaaatttaagtt
agataaaacaatagagggatacaatgaagaactagagttttcgagtacggggaggaacaagttcgagagagcctgatttgagggattagaattttta
ataatagaaagaggtgttaaaaggaaatttgatactttcttgtttttagttcaacaatatttaggagtgagaagattcgaagtttaacttttgatcgagaatg
catgtcaatcaccacttagctatgctcatcttgataaatgacaatttgatacctaataaaaaaaatcacctcctttttgcatacgaggcggattataaacct
atccattttcttcacaaaatcaaacaagaacttgccttttctaccttttaatacaaagtacattcttaccactatcctatttaccctaatcactaaaaatctagct
cttcaagccaaaattctctttaccctaaactcaattgcttcggccccgtttgataaccatttgatttttggattttggtttttgaaaattaagcttataaatccta
cttccacctacaagtttctttgtttttctaatacatttctttccctgtttttaaaaaccaagcaaattttgaaaactaaaaaaagtagttttcaaaaacttgttttttg
tttttagaatttgactaggaattcaaatgtgtcattgacaaatatgaaaaccatggtaggggaattggtagaaaacaagcataattttcaaaaaccaaaa
acaaaaaaacgaaatggttatcaaacggggccttcattattaatttgttataaacatatcacatagatttaatactttttttttaatataacaataaaattattgtg
gatgagaggttcaaactttccatttttaagaaggttgcaaatcactcaactatttaattatactcgctttggcacttaatgccaacttattaccaagtaagaa

aaacatgtcaagtaataaatatgtcataattttgtaataatattttagaagatttaaccaaaatattgaaactacaaaaaaaacttttaaagtacataaacta
caagtacagagtagctaaagtgaacatagttcaataataattgatatgtactctcgactaagagatcagatgttcgaatgtccccaccctcaattgttgat
tacctaaacagacaaaatttacttttaactttttgtatttataatctttcaatattgaaaatttaagggagagaaattaaagagaaaactttaaaatcttagg
cctcatttgatagttttatgtttttaatttttgaaaattgtgcttgttttctcaccttttttatacaatgattttcatatttgttaagataacatttgaattcttagccaaat
ttcaaaaataaaaaaagaagttttcaaaaacaattttttttttttttttagtttttaaaacttggcttgtctttttaaaacggtaaaaagtagagagcagaacaaa
gaagtctataaactataggtaaaaatagtttttataaacttaatttttaaaaatcgaatgattatcaaacgggataagtataaactttaaatttttttttttttttt
tgaaaatagtataaactttaaacttaaggacaaactaaaggggagaaaaatggtctctcctcatcaccctcgatgaaaagataatttaaaatatttttgttt
aagaaaagagagaagaaaatgtaaaatttaaaatttgagaattgtaaaagtaaaaaaagaaaaaaaaaaatcgtcagctacgtggactttccactgaa
tatgaagatagcacgaaatgttccgaaagccaaaagccccaaatacggaactttgtaagagagaaattcagagaaacacacagatacagattattac
cagagagaagagaacacagcttcccttcatca

>LcWRKY42 - Up_Stream_Len 2000

caaaaccttactttcttttttcttcttttcttgttgacgattgctacatcaattccatttctctctctcctcttttttttcttatggctaaaattttagatttttgtccc
tagatttttaggattttatttatttacttactttatgctttactaaatttctaataaattattggttaaaaatcacttttggtccctaaactttcatgaaactaacgatt
tagtccctgtaatttcgatttcataacgatttagtctctgtactttcaattttgtaacaatttagtccctgaactttaataagtaactatttagtccctatactttca
aatttgtaacaatttaatccttgttgcaaaaaaaacatcaaaattaaatgttgatttttattatttaatgattgaattaaactttgtagcttattaacatattgagtc
ccaaattagtttattaatctatctaggtaagaaatttcattaaatatttaccttaatttttcacgatagggactaaatcattacaaatttggaagtatagggact
aaatagttacttatattaaagttcagggactaaattgttacaaaattgaaagtatagggactaaatcgttacaaaattgaaattacaggcgttacaaatca
aagttcatggacaaaattattattttcatgaaagttcagggaccaaaagtgatttttaacctaaattatttatatatttaccatatattcaacagtttcttaaattt
tcaatttttatatggaaaatattttgctctatattagggatctccttacatatcttattaaaatcttgacaacgaaagagaaaaagtagaaaatgtgaaaactt
ttttatctctcttagttgtcaagattttcatggtggtatgtagagatgggtatgcaacaaactattaatgttctttttatattcaataagtccaagtgttagatttt
agtgatttagcacgtttatatggataaattatttaataagatcgttataatataactgcatttttaaaaccattcaaacttgtttgccttctaataataacatattg
gatataaaatcgatgtagttttacaatttatatgtcttgcaaaaattttaaacttttctctttgaaggagatactattttttttttcaaaatttaaagagtatgtata
attaaaatgtaaaattatgacaattatttattttaaaaaatatatacatctctcttttacatttctctccacttctagtaaaaaattcatgctcacatcctccataa
atcttacacagttatcgtgtcaaactgagcataatttaacaacaattgatatgtaccttcgatcaaaagatcaataaaaaaatttaaatctttcgtcaatgttg
ctaaactaaaaaaaatattatagacaattttttttaatacatcaacatttgagggtgggagattaaaagctctgacctcttggtcaagagtacataacaaat
tgaactatgttcgttttgacaaaatattataggcaattatcatatatgctagataaatattaattaaattatagtgaacaagtataacatattatattaaaatttta
ttttaacgtatgagaactaaataaacacgacaaaaagatctagagacgttcaaaataatacacgaaactaaatcaataaaacaaagaaaaaaaattta
gggacctatcaaacaagttacgaagtacagcgactaaatagaaacaaaccgacccgtaatttaaaattcaacatatattccaatcaaagatgaaccat
tttccgtggcgattacggcaccactcgcagctatatatttactgttcgtcctcttcaatttctccaaaatctctcctttctcttcttctcctttcttctttcatcgcc
ggcgccggcgaaatcgaaacagagattacacaaattcgtgttcgggtttcggccggagcgaaattcttgttcgatttttagatcttaagttttttga

>LcWRKY43 + Up_Stream_Len 2000

Taaaaaaatatggaaatgttggtgaatgttttgctctttacatgtagcataatatgtaaaatctaactcatgtcatgccaggctgattaatatatatatatata
tatatatatatttctttcttaagagtggactatacccaaattattatggaagaacctcccttggatttggactcgcttgaatttgaagatattattagaaaactc
aatttaattaacaagtagaaaaaatgccaaggcagacaagtaaattcaaataaataaatcttatccaagaaatgggaaaacataaattaaacaaaaaa
acaaaaagtcatattaaggtaatatcattcacaaccattattatttagattgagttataggtttgatccttaaacttttaaagttgtatctatttagtctcgtattt
aaaatatttttttttttattgaatctctaaaactttcgagattgcatatatctagtgtttatatatattaaagaaatactttaatagactatgaattttcaattttgtatc
taaaaaatctttgtcattgactctatttattatcacttccatagcaaaacatatgttatggaaattaagctacctgtacttcttaaataatataatcatcacaata
cacaagagtgttaatttgatacaatcataaacatgacttattaaacataaaattgaaagattaaatacctattgatttttttaaaaccatacaaactaaaccga
cagccttaaataaagttcataaaccaaatatgtaagcctcgtttgataaccatttgatttttggtttttgaaaattaagcttataaacacactacttctacctcc
aagtttctttgttttgttatatgcattcctccatgctttcaaaaaccaaatcaacttttgaaaactaaaaaaagtagttttcaataacttgttttggtttttggaatt
tggttaaaaactcaaatgtgtcattgataaagatgaaaatcatagttcttataggtaaatttggaccatcctgaaagtcagcagtttgacgaaaacaaca
gaatgcccatcatgagatacaatcaaaagccataccacctcagagacagagggccctcgaccgacccttggcacaagcccgaggatcgccaaac
ccttctgattcctggtccctagggtccaggccgagcccaattggaccgaatcactcttgaaggcaaaagaaaaccctagaagacaacttaggtataa
acatccttttcacatctggagaagggttcaaaactttaattcttgacacttcttgcttcttgtttgctgatttaaacatcagagtgagtgtggcgagcaccac
aacggtgtgcagtctttattggtgttgccgatcacacatttcccttcaaacaaatttattgtcatgtcacacatgaaggtcaggtgagttttctacccgaatt
tttgcatcaacaatagtaatgaaatcaagaaaaaacaagtataattttcaaaaaccaagaatcaaaatccaatgattatcaaacgggaccataataaaa
caattatttaatatgatagaatatttttgcgtccatgttgtgggagatattaaacgagaaaaaaaaaaaaagagttggaaaaaaatggaattcaaagataa
ctgccggatagaagacgacacacgagtggccaaaacacatgagaaaagaggtaataaaaagacaaagggaccaaactctatctatcgaaagaaa
gaaaaaatgaaaaacaaaaaaaaagaaaaaaaaaaaaaaatggaaaacagcgtaataagttggaaaaatataagtcagccgccattcagtcaaacc
cacgagttttgaccaaaacacagtcaacaatcacttggtttcctcgaaccatcctcttccctttcttcttcttcttcttcttcaataccctcttcctttcccttctt
taactttctctttctaccatttctcttcacaatctctcctctttctctctctctcttctctc

>LcWRKY44 + Up_Stream_Len 2000

Gtaggcatgctcaaacttttgggcttggtccttggaatggccgaggcaagccaagctctttctggagcttcctttcctttggctccgatcggttctttgtg

gcctgatttgtgctagatccgtgtagctacatcctattcctctattacaggattggagcaaattgacctataacaagttctactatttttttttaaaaagggg
caatgtcgtataaacaattaacttagaaaaaaactaaagaaaacacacatgaaatcaatcaaatgctattccttcttccttgttcttttttgttactatggag
ttaaacatttttctcaaaattaatttttatttttaaaacagacaactatgttaaatcaatgcttctaaagagcagtgttaagaaaagtttattctaatttcccccct
aattttggaataaatattttagatattttagaaaataaaagcaattacctgataaacaatttaaagctaaatttatcgttaggtcctaaagaatttagtctttaa
cttacactataaagtatcaattttaattacattaaaaggtataaaaaaaaaaattcaatgtaactcctactaaataacatttattataattataattaaataaag
aaatagtaacataacacttctaaaaaaaaaaaaaaaaaagaaagaaagaaatagtaacataacacttaaaattttatttaactatcaattcaaccccacttt
attaaaaaagaaaaaaaaaatggatagggtaaaaacaagttgaatttttaataaagtacttaaaatgtaattttctagtcttgtaaggtgaaatagatcctt
ctctcactaccaagaaaaacatttaaggcttagtttggatcggaattgttgtaacttctaaaataattgctgttaattttgtttttaagaaaatcagttgtactat
gaagtaaattagtgtttggtaaattttagttttatagatgaaaaagcaggttgaggtgtttggtaaattaaaattctaaattgttacagcttaactgtatttttc
aataaataaatttaatgttgatatttaatttcaaagttttataagataatttcaaaattattatacgataattatttttttgaaacgtataaaataattattttaaattt
catatgatttagataaaatatgaaaggataaaagaagaattttaaatatttgttagttacaccagattttcataaaagcaaaatctcaaagctactcttaaca
gtttttgcaaatgattcaaaaattgattttccttaaattattaaagaataagtttaaccaaacacaaaagacagttaaaatttttctgcaatcactttttacaact
tcaaaatcaatcccaaacgggcataactcatagaaattattatttttaactagcaaaatatctgaatatgcacttaatataattgaaatatcatgatacttcg
agaggatataactgaaatggaaagaataattgatcatattttagttattattttatataacatgaaaaaaaaaaaacaaaaaacaaaaaattgtcaaacgt
gccacaaaagtttagaccaaaacatttttaatcaaactgacaaagacacaaataccctcccaaaaaaaaaaacttgcaataatttcttccttatatccac
aaggtaattgacatattattttagtcataaattcaattaataagaacaagtcctacttatttttcatgtgacgtactttaattttttttttgtagactatagagaga
cgtaagtattgcttaataaataaatggacatcataaatgtttttttaaaaaaaaaaaaaaaaaagaaagaaaaaaaaaaaaaaagagttgtctttataaat
gatatcaccctcttacttgccgtttacgaatattaatcaaacttcaaagtattattgaagaagaacctctcatctctagccgccatttctctctctctctctc
aataccaaattaccaattgagag

>LcWRKY45 - Up_Stream_Len 2000

tgtatttttgtctaatcagaaatccacacaacttctttacaaaaacaaaagtgtgtgtgtatatatatatatatatatatattaggattagaaagcatcatttattta
agttttgttgaaaacaataaaagttattgatggacatatacactttaatttttgttaaaaaaaaaaatctaaattaacagtactcttaaattccaattatacgta
cacgtatggttcacaaaaattggaatataaatcaaaataatattgattccatattatcctttgcttatttatgttggagagctaattcattatttttttttaaatcaa
ggaataaatacatcatttagaaagcttattctacgggaaaagcatttttaggttgagtaaagaataaaaatgattatttaaaaaaaaaaaaaaaaagaata
aaaatgattgaaatagattaggtagaatctacctgactattcaaaataaaattaaatcactctattactagcttattgcaattattttagtctaaaacaaaaaa
gttatttcactttattaattactctatttttctcattattggtttattgattatcaattaattgtcccccgtttgtaatttttctaatgtatgattgggttctaaaggaag
tgacggagtaattttccgatcttacaataatataattctatttcttatttgatccatctaagcataacttaattattaatacatttctagctatctaaatgtctacg
cttttgtctctaataagagtaataataaactataagttaatttgattatattaaattaagttttaatgatttatatgcatgtgtacatgtttaaatatgacttcaaga
ttcaaccttatcactcatttctcattttcaattttcttttcactccttatacttcttacaaatttcttttgtcattttaagtatctatactcaagatatttaaatgaaatgt
aaactattttattaaaacatttcaaataactaattaactttttccttttaagaaaaaaaatatgaaaatagctcaacaattaatgcacctaaattttcaaataaa
aatttaataaaggagggaactcgaacttctgacattcagtcgaggattaacaaattaacattaattttaatgtttaaatctcattttagtccttgaacttttaag
tttgttttattttagtccttaaactcttaaaacgttcattttggtccttacactttcaaatttgttctattctagtccttgaactttcaaaatgtctattttagtctctaa
cttatttgacttgaaatcatgctcatattaacaatctaatacacatactcaaagctatgtcattaaatttgttaaaattgaaataaaagtaaggaccaaaata
gtcatttttttgaaagttcaaggactaaaatagatatttcaaaagtttagagaccaaaataaaacaaactttaaaatttagggactaaaacgaatattttgaa
agttcaagaaccaagatagaataatcttaaaaatttaaagaccaaaataggatttaatccttaatttaattattttataggataaaccaaaccacacaaaaa
gaacatgatcataaattcacaatttatatacacacatatataatataaataaaataatttttttttttattaaaaaatctattgtcttttttttttgcctacgtgtacac
ggccattaagaatctagtttacactggaaccggttaaccggtcaaaactccgcgtttcagaagtctccttttccaccagtcctctgtcatttattgcgaac
accgatccagtcgctcttgtttctatgcccactgtttaccggcacctgtcggtcaccatcctaggtgggaaaacttacaccccaagctgttctccaaaat
cccaaacactcttttctttttctcttacactctccaacaaaagaatattattattattattatgtcttaaacaaaaaaaaaaaaaaaaattccgatcgatccca

>LcWRKY46 + Up_Stream_Len 2000

Gttttcatccttgtaaacaaaacatttgaattcttgtcaaatcccgaaaacaaaaacaagtgtttgaaatatttttgtagttgtcaaaacttgacttggtttttg
aaaacatgcgaaaaatgttgataacaaaacatagagatttataggtgatagtagtgtttataagcctaaatcaaagactagatagttatcaaacagaact
tgaaataatagtaaaaaaaaaaaagggttaaattacgagtttggttcctaaactttaggtttatgtctatttgatctctaaactttagaaggtatctaataggc
cgttgaactttcaataggtttctgaactttaaaaagtgacgaatatatttttaaaatttcaactttatgtatctaataggtccttaaaattttaactttgtgttgttt
aaaaaaaaaaaaaaattaactttgtgtataataggctattgacttattcaactatttaaaaattcatggaactactagatacaaaattagaagtttaaggtttc
atcaaacattaaatttagttttatagcttatagattaattaatttttcaaaactccaaaaatgtcaaagacatattaaatacaaaagtagaagtttaaaaactta
ttagacatttctaaaagataggaacctattaaacacaaaattgaaaatttagaaatctattaattatcaaacactttttaaagtctaaaaactagataaaccc
aaatttaaaagtttaccgactaaatttataatttaaccttaaaaaaaaaaaagtaagtggtagaaaaaagaaaaaaaaaatctaaaaaagaggaaaaaga
ttccaaaaataaatagaaaaagtttcacccaattctttaccaaccattaccccaccaataaaatcaccacctcatgtctccaataaaattgataggagcg
ctccagttttcactatgtccagtccccttctagttctaccgtaatttcataatatacatttttaatttaaaaaataaaaataatgactctttttgtctgtacgatca
cgagcttggattgtagcgtgtggaccccgcacgcttatcaaaatcaacctctttttttctttctctctcctctttttttctgtcatttaacaatttgaatttgggcctt
tgacattttccaaagtcaacccccaaaaaatccaatctttcttcaaatcaagaaataatcaattgggtattttaaaaaaaacatttttttaatagaggggagg
aattggaaaaagaaagaatagcatagagatgctctcctccaccccacctgtctctttctctctctaaaaaaaaagttaaattaccatttcaatccctacactt

tagaactcattctattttagtctctgttcttttaaatttaatctatttactttcaatgaatcttaaaatcagttcttatgaaaaattataattgtttccattctaaaaaa
tcattgtggaaatgttttcaaaatttatggagaagactaatagcattttttttaagttacaaataagtaataagaagtcaacatgaacatatctcaactgata
ctaatactaagactaagcggtctgtctatggttcgaatctctcacctccatatgttttaaaaaaattaataggaacgaattttaagatttattgaatagagac
caaatttatatcgaagagattaaaattattaatacatagaaaaagaaaaaaaaaaggggggcaaagggaaaggcaaaggccacaactttctctctct
agctagcacttggatttgtgaacacaaaacataaacacctcacttttctctttctatctctccattttggtttctatatataattctttttcatactcattactcatt
acacccttatcaccaaaagtcagcgccttcttttgtctctatttctttctttttctctctctctctctcagaaaaaaaactgatcaagaagctgagacaaag
gtacagattttttcagagcagagttttgtct

>LcWRKY47 + Up_Stream_Len 2000

cgtggatgttagacgagattcatgccttgagaccattcaaggacgccgctcatatattggccgagaaggaggattggcctcctctatatgacattgctg
ctcttaaaaataaccaggtattctgttcaaatgattctacatttccaagtgagcaaaaatgagcttgtatttctcatacattgtcttctctgatgttgcttgtcat
gacgagcattaggattagcttgggacttttctgttgacaaaagaataacatgttttctcgataccgatcacaggttccggtcgcagctgcggtttattacg
aagatatgttcgtaaacttcaagctggccatggagacagcttcccatattgcaggaataaggctgtggattactaatgaatttatgcattctggtctgcgt
gatgcagggccccaagttctggatcacttgatgggattgttaaatggaaagaagcctttattctgaggtttttctgatcttttgttgcttttcttcataattatt
ggattgagttgttgccattgccataagctttttcattttctcctcaataagcttgttggttgttagtgtgagatcaggagagtgtaaactggtttatcctttctc
ctagataagtattttttttccattaattataacaaataagtaaataatgttttaatagctaaattatgttcttatgccatgattaatgatgttaattacgtgggact
aagtagaagtgaagttcaaaattactttttttttcctattttgtggggtttttttttcttcttcttcttcttaaattgatgagatggagattaattattcagataatcgt
tttggatttttggaagacacaattttgtatgtcttaaattataaagaatttatgtcaatttaagtataattcagtggataagacattaatgattatctcgaaagtc
gatggttcaatccttaatcttgaactaacaaaatttaaaagaaaatctaccaaaggaatcagccaaatgcaatgttaaatgttgtgaagaagttgtttaac
tcccttggatggttggtcaacagtattttattgatagttgtatggttaacagaaaataacttatttagggtaactttttttgaccgggttattcttaatttaatctc
gaatttaattaatttgaagcttacccaacctaaatatagtacaaaagtaggtacttaaggtttttttacgaatctttcaatctcatagtgttgaataaacaatatt
tggttgcataacgtaaacatctcccattgtatttaataagtccttaaactttcaattttgtagctaataagttctatttttttaagcttttgaacttttaattctgtgac
tgctgagtagtaggttattgacacattcgatatttttttaaaatctgctaacttgttagacaaaaagttaaatgctactcttaataagtatatattttaaaagact
gttggatttattaaacacaaaattgaaagtccgtcaagttataagacatttttaaatttcaatcatcaaagatacaaaattaaaaatttaaatatttattaaaca
ctttttaaagttagtgtacctatttgacattgaagaccatgagaattcatgaactcaactaaactcgtaatttaaccaaatatatatcaccatttaaaatttctc
caatcaaagtaagacaaggaaatatggatttaaaaacaaaaaaaggaaaatacatgactatggatacactatcaaaatcttgacaactaggagagat
aatttttttttttttctctcacgctgtatacatactagtatgcagtagtaagaaatatttcactttttaaaaaaaaaaggaaaaaaaaaatgaactatggtttaaa
gtggtggttgaagtagttgaaatatagttggcttcccccatctctctcactctttataaactccaaatctcaaacttccccatttcctctttcatcatattccca
acaaacccacaaagaa

>LcWRKY48 - Up_Stream_Len 2000

atagagggaaatgataaatgtcccaccttttcaattcaaggatatatgtccttttgtttgtgtcagcatcctagtaaaattaccattttactctttactctaactt
cactctctcttgctactctcgctgcgctccaacctcccgctcctagcttctcgcgctctctctccaatctctcacactctctcaaatttctctctccaatctctt
gctccctctccaaccttacctctagcttctcgcttcttgctctctctccaatctcttagtctctctctaatttctctctccaatgtcactcttaaatcttgctctcg
ctccaacatctcgctctttaaatgtcttaaatgcgcattttcataaaaagagtaaaatgataattttactaggatgctgacgtaagcaaaagggcatatatc
cttgaataaaaaggtaggatatttttcatttggcctctccaaatgggtcatccatgcaattgtttcttaacattttatgcatattctcaacactcttcttcatttg
taaaagtttgagcttaaacatttggctctaataccatcttaagcataggttatgaagtatttaattttttttttagagttcaacaacatgtaggtgggagagag
aggattcgagcctttgacctcctaattgagggtataaagtttttattagaagaaaaatattaatttgatctaaattgattcacctctattttttttttttttactttaa
aaaaattaaggtttaaataattgatacaattattagtttgggttttaattgatacaaattagtatttgtctctttattttattcaacatatataaagttagaacgatt
cgaacctttgaccttttagtcaaagacatatgtccttaactagttcaactatgttgagtttggtcaaggacagacaccaccttgacctgttatattgaactat
acttaggttagatatttagactcttgtatacatatattagggttcaaaaccaaccacgtcgagacttgcggataaggaaaatatttaaaaaattcaatatatat
tttttttccctttgaagaagtccaaattcgaggaaatggggttgattgagactcaagagagaatgctaaaagagtagagaagaaatgaaaaagtttgaa
aggcccacattgtgttagccatcaatgtgtgtgcgacgtaaccaaagggtttatgccttacttcatcaaatgacatcactccttactccatctctcttcaat
aatatttattcatcttcatcgtcaactaacaacaataaataaataaataaataaataaataaataaataaataaatattcatcttcctcaatcaaattcaaagtcagc
tcaacacgaacaagaaggaagctcgaattttcaagcctctttccgcgttgttccacttcaaaggatcgatggcttttgaaatggtcagcaaaatttgttct
ttttggttttggccctttctctctcacttcgatgttccctctccaagggttaccctaccaaattacctcatatgtttttgtttcattaaattaatctcagcctactc
ggtttctcacagtccgagtcagtcctttttttaaaaaagtaaaataaaatacaatcaatctctctttcatagcccttcttacacgtcctttttttctctctcttcaat
tgatgacatctttataagcccttctcttccaaacccatttcccatagctcttcaattctctcaaagtttggagctttcttcacttttgctcatttgggttcttagaa
attgggatttgggagtgacccatttagtgaaacttaaattttctttgtgggtttttggccaattttaaacctttattgaatgaccatatttcttttaggttattattc
tttcgagattgttattattattattgcattgtttttttggggttttggctgatctcaactattgggccgccgccatttcttgtttgggattgaatttgggactttggg
atcgat

>LcWRKY49 + Up_Stream_Len 2000

ttgcaatttaacttcaatgctatttattattttgccattattactatcctcctttcatgcggcctcatgttctatcatttttatccatgtatctttttattacccatgtca
ctgtcaataaaagtatttctttgatccttcatgtaacgtataatttatgttcacatccatataagggttttacggtatatatcaattccctatgaaccattcaaaa
actaaagtaagcatagttcagcggtaattgacatgtactctcaactaacatatattattttctccttaccaataaaaatataaaataataataataacattga

aagaatataggaacaggtttctttatttgactacttctaaatatgacatgaaagttaaaataacataattcaatgccaaccagttcaagtttaaccatctata
taaataacataatctcttttggtctgtttctcacatacattgtcgtttgctgctgataaaagtatctttcccaaaaaaaaaaaaaaaaaaaaaaattaacaat
ctatacgataaacattaaaaaagtatatatacatatttacttgaacaaaattgttccaaaaagaatgagaagtaaattctttgatgggataaaatgcatcatt
atactgacctaaatatggggaaaatgagataacgggagagatgaaagtaattaatgttctttttgaaaataaaaatgagttagttggggaaagaaggg
cttccaacctctattatatgggtgggcatgtcaatgtgtgtccctctactactagatttagattacctaaaacatactcttcttatgacactcattgtcaatata
attatcaagacaaattacatcacattattatattcaagactgacaatcctaaagtatgggcaaagtttaatgttgcacttctttttagaaggttacctatcaag
tgaggttagggagtaatgggccgatagttgattcatattatataaataaatattataatattggaggttgttaagcagatgttacaacttattcaataaaata
tggaatcacaaaagataaaggtgacattttgaaaccttgctttatacatgaagaatacaagtaatcaattgtgaaacgaacactttttgtacaaaaaatat
atattgaaataaacaattgaattgggcaatatagaatttaatttaggatatatataattaaccgttggttaaatggatgtggattggattgaatgaagcggtt
tttttgttagaataaaaacgaatgaagagagaatataaaaagaaaagaaaaggtggggacaaaaacggagactttgggttatagttaaagtatgatag
aaaaaatgtagaagtgagaaatagatggtttggatgaaaaatggttaaaagagggtaaaataagaaaagaagattcgaatattttcaatagcttttaga
gtacgaacaaaagaagtggcgtagacccgcattatggagcagcagctggcatatgatatgttttaatgggcagtgcgactgtgcaagtttctcgtctt
gttttcaagatgtacataattgtactgtgcaaacggggaaaaagagagtgagaaagttattggaaatgaagaagctagacatataaataaagaaatgc
atttaatttaatttatatatatatatagaatacaaggtaataagcagctaagaaacaaacgttgcgtttgtacagtagtagagagagagagtagaagtggaa
gacaattttgaaaacattccttttcgcatttgaagtcgttgacccgaacagcgccctgacctgatcgaattgtgtgtgagacacgagagcaaggttgttg
cttttaacaaagtgccctaaacaattaagcgaacaacaacaaggagaagaaaacagcagccaagggatctttctttctttctttctttctttattttctttat
ttttctttctctctttcttcctgcctttctttgttataatcatattgtgaaagacgaata

>LcWRKY50 + Up_Stream_Len 2000

Ttggctgcataccggtatatagggatccctacatattggtatgcaaccaagttttttcccaaagaaatttgttttcttaactctacccaactctttgtttagt
ccaaggacatacaacatccaattgtgaggtctgcaaatttattaaccaattacaacactctattgtgggtcccacaaacttattaacttctattccaatactt
catcgtgggactcacaaacttattaacttctactccaactcttaggacaaaacaattccagatttcataactccaactccttagaccaaacaaatcgtaag
gtttgtggagatcaaagacaagacatacattccctgttcctcccccgacattaaatggagaagaaaaaatttctacctcataggcaaaaatttgtcatct
ctatttactacttttcaaaaatgcatttttttttttttatattaaaactctgccacgtgctcccatctaataaaaaagtatcatagtttgtttaatcaatcagtattc
aaatcactcaattattctaattgctatttttaaaagaaaaaagaaaggtttttaaagttgagattagaagcctaaacgtaaggtttggtttaaaaagaaaac
gtcaaagtggcatcatcacttccaagcaaatgcaaatgcaaatgtgggagacgaaagcaaaggagtaatctttacgcgcttatgcccttctttctttaat
atttctttaatattcccttttgacttttagtatttgtattctcgactgcggttcattttgatcttaaatctctattttaagaaaaattattagaagagcacggttttata
ccattgattgtaggggagctagaaggttacatgtcaattaccgttgaactatgctcactttggcaagctttcgctatttcatccaaaggtttaggagtagta
acgatcacattaaataattttcccctctttttttttttagttcaacaactatttggaggtgagagatttgaacctctgacctcttggtcgatgatacatgccaatt
actactagctatacccgctttggcaaataatttttctcattttagtatactttcagttttttgttcattttagtctctgtactttcaaaaagtttgtttccgttcttgatt
ttggttcctgtttacaaagttttagcacaaattttatacacaacgaaaacatttcagtacgaagttatgactacatttcaagagattcattgagtgtaccgtg
ataaaataaaataaaaatggaccaaacacaatcgtttctcgaaagtgcagagatcaaaatgaaccggaactaaacggggagtgaaagctctatttaa
acctttagttaatcatttcatcattccttatcgtattctacaactcactcacatcttgtcacactgttttgatcaaacttgaaaagcattcactttttttatgtgattg
ccctgtttttaaaaagtggaattggtcaacaattggacttaaaatctctcaatcaatttacaagcaatgtatctacgaccgaagactggtctcctttgaaga
aactccatcgatctcagtcaaactctctccccttcgttttctttggtttcaaaaagattctcagtctcagcctctttttaaattgcttcctccttcatcaacttca
caattcacacttctcttttcttccctctttcaccatttctgtttcttttcatcacccaaaacttctgcaaatgcaagtctaaaacctcctttttttcaactttttctca
caacccacttcatattttggctttttttccatcatttttttgggtgagcttcccttgaaagatcctctttggtttgggactttaatgtcaagttctgtttagctgcatt
gtgcacatatataagctagcttcctgttagttttgtatgtgcattttctggctggcttgcatttgttcatagctattcttcttcaaagttcagaaattttaagtctc
cacctttggaa

>LcWRKY51 - Up_Stream_Len 2000

Aaacagcccacttcattaaatagctagagtctaaagtatactattttttttagaatattacataaaattaagaatatatatataataattagaaggaaaaaaa
aaaaaattgagaaacatgggtgtagagttatgaggtcttcgagttgaccatttcaagatcggatcccaaggtttctctttctcatgggatttcgttaaaca
ctcatatttaattggagttgatgatgttgagtttgcattctttttttatattattttatttcctcttctatttttctttttttcttttttttttatttttttattttttataatgtgaagtc
caaacaccataaataatgacagtgatgcaacagctaattataaaatttaatccccttccaataaataagctaagctaggtttcaacctttaacaaacatta
attaacacaagaggctcttagattaaagaatttgatttgcaatatcggttacgatctaattggacaatgtatgagaagacgtgtttaagtttatgtaatgtgt
tgtttcttttgtgattaatattaatggtcgtttagtgggtatacgaactaaaagtttttagattttagattagattagggttttcttctttttttttttcttccgaaatttg
tttgaattatttgtaggtttctaattttacaaaaaaaaaaattaaaaaatacacttgtttgctaaccatttcaattatagaaaggattattagtgttatttttttaaaa
ttctatccaatactttaaacataagagctttgttacggattttgtttttaattctcaatttttagaatagaagcaatatgaactcaattagcttttttaatatttttatt
attacttctatctaaatttttcttttaaaattatagtcaaaattaaaagagaatagtttccaaaaatgtcgacttatatttcgaaagtttcttgcgaagtagaaga
caaaaattgagaaattatttgcataacattattttttgggataaaaaggaaaaaagaaacaaaattgtcatctttttttttttttgaaaaaaaaaaaaacaaaatt
gtcatctagtaggaaagataattttccgaataagaaatttccatttttttttttcaaattattaatctaagtatagtttaatggataaagacactaaaaattatcttt
tcctaaaggcggaggttagaactctcaacttaggttgtatttgttgagtgtttttttctttttttagttctatatttgtaggagtgagtgattgaactatcgaccttt
aaaatggctattaatgtctttatatattttttataaaaatcaaaccaattgtctcgaatttgtgcttcaaaagatgtttaatggcttggacacccaactccacgg
gctacaattagctgtaataaattgttggggctgcttaattaattaaaagttaggataatacggctgctaaagtttctcctaattatcacatagttgaaaaaaa

ttaaaataccttcaacaaaaaattaaaaaaaaaaaacaaaataaaatagagccaaacccatgttttcatctcgaatcaattatatctaaaaaaccaaaat
atagctgccacaacgtagctgtctcttagtaactgcgagaacagagagcaggtataaaatttgtctctctctcgtaataaccattcgacgcaagttcaaa
atgcatctcgtaatttctctatcaaacatccaacggagatgtaaaactagcgatcgcactgtccgatctcggtctaataatttatgatctaaaagagatta
gggtttggcctaaggggtaaattaataaagggaccccattaaagtcaaagccgagatgcggccttgtgtgtgtaataatagtttttcattgagagacga
gagaattgactttggtgcctcaaaaattgtattaataaccaaacaaaatggagagagacagactttgtcttcttcaatttggacagtggagaaagaaag
agagagagaaacaaaa

>LcWRKY52 + Up_Stream_Len 2000

aaggtaaaaattgtaccacttgttgaaagtttaatttcatgaaattgaaatttagaataagatatcttgcagttgtcgagagaacaggaaggaagggaaa
aaaaaaggtaaaattttattttatttttttccttaaacgttttaaaatttaatagggagaactcattgttgatggtcaaatctagacagggaaaactcacctggc
tttcacgtcccaccaacggtaaatttgtttgagaggggggaaagacgtggcctgcaagacaagaaaaatctgaacaccggtgtggtgcttgtcacacc
gcctccgatgcttaagtcagaaaacggaatgaggagagtaagagagcaagaggagagagtcgagaatagaattcgggatcctttcttcagtgacg
aagaagggtttaaatacctgttcttgctctaggatttctaggctttcggaggcgattcagaactgaacccagtggaaccggggcggccaggggcagc
agggaccgaacggaggggaacggactcggcccaagcaagtggcccgaggccgaccatatgggtcgggacaaggcccggtccctccgaccttt
tggcccatgtcgcctccggggtccatctttagatcctatttttggcctcgtgtcggttgtccttatcagctccttgtacatcgaagtggtccaaaattacc
tagaacactcatgaacaacatgtaattgactaaaatcatcctaactgaagagtagataaggcataaaaagggtgaataatgaaactttttcaattgccaa
caagaacataactcaactgtcataaaacttgtactaatagtcgagagatatgtgattcgaattccccccacatattgtaaaaataaaaattaaaaaataat
aataataataattaaaataataattaaaaaaaaaaacttttcaattgcatcatatcgtgatactattattatgtcatatcaaataaaataattaagccgaatg
agaatgaccaaatttcaaacttatgtaaaataaataaacgaaaaaacattaattttgaccacaaatttttctaagactaaggtctaagatttaatttatgttgt
atcaaggaaaaataagtaaaagaaaaacatgtatgagaagaaatggtctcaacacttggaatttggtgatgcaagaaatccaagtcagcagtcttatg
tggaagagctgtccactcacaatcataagctttagattttaaaaaacaaagagcaaaagcacatgtttactataccacaacgcggatataagctttgaa
aattcttggtctactctccaagcttgaactttgactttccacttctctctctatctcaatcatggaatttcattctacacaaatcgcagccgtcggatctagag
agagaaagagaaacccttttggaagttaaatccaagtgggagtggacttggtcagcttttgaacttggacttccacactaatcttagtctccactgctcc
aatgaccaaagaccagcctatcaaaagaaagagaaagggagaaagcaactgggtttgtctcagtcaaagtttcccaacttctaactgttcaagaaatt
tttaaaaatgaaacaaagttcttttcctttttgaattccttataaaaacccaagactggttcttcttaaacccaaagctctcctcatctccctttcaatctctctc
tcttcttcttcttcttctctctctctctctcactattccacttcagctttaacaaaacacgaatatacccagttaaccaaattacctatctggctacttccatgttt
gtagtctctggttctttaccatttgggtcttccacttcctctagcttctgcttcttcttcttagatagaaagcttctgcttcttgaaaaaacctacaaattcaaac
agagatgtactgaattcttgttcttcctaccatgaactagcaccgtagaggttcagttcattttatgaaactaagaggaattgaaa

>LcWRKY53 - Up_Stream_Len 2000

cgtcaagcaagcaaacagcacagagaagctggtcggcctcatgccaaggccgagacagaccatccaccttcaaaaggccaggtatgaattagtc
cttcttactcgaaaaatgcaacgcttgaagtaataaatgcacatgtgaagtgggggctgggaatagggataggttaaggcccttccattcctttgttgta
atcatcatgcaactctcccattgttttccagtaaggactgatatatgaatgcagggggctaagtgttgagctaacaggggcctgaggccaaggccgat
cgtagcctcctctctcatttttcttttgcagaaattaacttttcaatgcaggattgtgaagtcttctgtgttttcctatttttaattatggataagccctggatctg
gaatgaagcactccgaatgaccaagtacaggggtctcactggataacaaagaagtcatacatgagtcagcacgagacaagagtcgtactctcaaa
ccgggcttaaagagctcagcttatagcattaagtcgcacctgaatcctcaattcaagagtggggacttaatgttataggtaattttggaccacacagat
gcacaaggagctgacaaggacaatcggacagaggtaggaccagaaaactgacccagagaaagaacggatcgaagggtcggccatgtatggt
cagcctcggcaagaggccgaggccgaccatccggcccgcgcggacttggacacccccctttcggtcccagatgccccgaatcgccccggttttg
cccggttcgtcctgaaacacctccgaattcctaaaaatcctagaaggacgagcaggtatttaaacccctcttcatcattgaagaagggttccgaaaact
ctattctcaacttcttcctttactccctatcttctcctcggttttctgacttaagcatcggaggcggtgtggcctacaccacaccggtgtgcagcggttttgg
ctggtcttgcaggtcacgtcttccccagtttctacaaattcactgttggtgtcaagtgaaggttaggtgtccggattttggtatcaacagattttttattttttg
tcttggtttttttgaaaattaagatcataaactacttctatctataagtttctttattttattatctaccttcttcccatgttttaaaaaaactaagtcaaagattgaaaa
ctcaaaaaaagtagtttttatttttgtaattttgttaagaattcaaatgtttcattaacaaagataaaagcatggtacagaaagttgagaaaaaacaaccaa
aattttcacaaacaaaacaccaaaaagattaaatggttatcaaacggacctatatatttcatattttttttaaaactctagtaaggattttagagaacaaata
gacataggaaaaaaaaagcacaaatgacatcaaaatatcaaagtaaaccataaataggcattaaaacatgggacttcaatgaattgaagtgaaatgc
ttgatgggtcaattaaaacattatatccataaaataatagttggataagatatttaaaaacatgaccctttgactgcaaccatacaatgaagatagaatatc
ccagaaattagctcttgtacaattacttgtaacaattgaaattaaaccaatttaatttggaaaaggttttaatttttccaaactaccacaacttcttttcctcttg
aggccgtttaacaacatcatcaacaacatctcaaatcacaagccatcaaatatacaaagagagagagagagagacaaagacaaatttttcttaaaatttg
gaaaaaacatagcttgaaagttgaaactaagttaaacccaacaaacacttaccaactaattctgttcaactgccctgccccaaaaacaaaaacaccat
ctttgtttctctctccaaaaacatataaaagttttgattttgagttttggtaaaaccctagtaataatggatggtgacattaacaataataatccacat

>LcWRKY54 - Up_Stream_Len 2000

Taagcatctcaaaaagtacttttcctttgatatttatttttgttaaatagcctttagctttaacgtgaggaaaactatcaaaccattaaaaaacaataataata
actaaaaagtaccgaggacgaagaagaagaaaaagactaaattcatcgttatttatgaacgagataagaattcacattcaaatataactttacaataac
atcattctagtatccatttcacataaaaacgcacccgaataacacctaacacacgtcaaatttataaatgaaatcgtaggtcgaatggcttgaattccata
ctccctagattagggttaaaagtttttgtccctttttgttattaatgagagaaactagattagggcaagcctaaggctcaacaagttgaaggtggcaagtt

gcaaaagaggactttagattattttaatttattttttgggtgtttgacttttaggaaggttcttgataatataggattgggattaggcagtgggtttttttgactg
aaaaaggtaaggaaagtcggaaagcaaaagctctgtatttgagcattattgaaagattggaagtatatataggcaatgggaagcaagcgtgggaaa
ggccaaagggagggggagcaacctatatatgatgttgatctctccctctcatccactttttgagatatgttttatccatatggatcgattattcgatatggg
acttttcattggtcgatgtctcgatataaacaaagggttgagagtaggctgataaagatattcgagttactgtgatctatgttaacttaattgagatttttga
actttgagagtggtgtgatttaatatagcatcggaacatgaaactctaagtttaaatttctaaatgttatttcctctaccatttatgttcattattgtccaaggtt
cgacttcatgtgttaacattatagtttaggtgtggaaaagttcgctaacctatatttcaacctaaatttttgtatttaaaggagtaaatgtgtgtttgcaagga
ctatgagtgactcaaagtaatttttttgaaaagttttggtagtgtttggttaaagtttttagggaatagtaaaaagtgttttggactaattaaaagagattttaaa
aattttcgatgataagaaggtcaaatttaaaaagtattgacatggtacttctaaaggaatcgtttaattaatttttttaagaaaatactggtctaatttagtgttt
tttcacttaaaatcattcattttctttcaaaagacatgtcaaacgcactaaagggttgtttgggagaaacaagttcaaattctacagtgtccacctactaact
aggatattaaaatatcattaatagacaacgcatcaagaaacatcttaaccttgaaaattaattaatcacgaaaaaatcgatttttgctctctcaaatagggt
aatagatgagatgaaaagaaaaaaaaaggacccttttatcttcaaagtcattattgagatatttaagaggaaagcttgttaacatggacaacaagaatcat
caacaactctgtccactcattaaaaaaaaaatagtaataatacttcaaagaaaaagttttgaaaataacataagattatattaatattatcaaaagaaacaa
caatgagagatttctcttcttcacacattaaactagttacaaacaactgaattaaaaaaaaaaaaaaaaaaaaaaaaaaaaaaaaaaaaaaaaaaaaaaaaa
aaaaaaaaaaaaaaaaaaaaccactttttttaaacccgttgacatttcacaaagtcaatggaaatacagattaaagcggcaaaaagccccaaacaac
actttcacaagatcccatttctccaattatttaaacaaactttgggcaaatttaatttcctcaaagctcaaaactttattttcaaagtcagctgatccctcttctt
aatttccactgtgtgtgatctgcaaatttttcttctctcttttgaattatttgattttcc

>LcWRKY55 + Up_Stream_Len 2000

ggtcattaataaaaatttgcactggactccatcgagcccaatgcttcttaaccttcttacactcccaaagagcatggaggactgtctcatctcttttcctgc
acataggacaacatgggtcacaagagatatgtctttttatgatgttcatcctacagggtaaaatatctaaactggctctccaaacaaagatcttgattttgt
ttggaatttttagattccagagtagcttccaccaactggaaatagatgaatcagatgatgatgattctccctggatctcttccatagcaagcttgtaccca
ctccgaacagagtatttgcctcgtttatcatagtgccagaaaaaactgtcagggtgttgggcacctgcaataggaatctcctggatttttactacatcatc
ctgcataaaaagttctgttagaagatttacattccatgattgactactagagaaaaggcttttgactcggtagtcagtcagcgggcgtttgagagagaa
gtttaaaagtcttcatccttggaatccacttgtcctcatagatgtcaattgtttccccgttcccaatcctccagcgaccacctttcagcgctagttctcggcc
ccataaaatgcctcgccatacatatgatggtctgccccccagacctgcattcaaaaattcacaattaggatagtatctcgctttcagaacttgtgccact
aaagattgaggtctgtgaagtaagcgccaagtgtgtttcgccaacaaagcttgattgaaaagccttatgtctctgaaccccattcctcctctgtcctttgg
tttggtcattgctccccacttcagccagtgaatttttctccctagttcagagctaccccaccagaaattagccatcatcctattcagctcgtcacaacaac
cctgtgggagtttgaagcaagagagggtgtacgttggaatggcctgagccactgcctttataagagtttccttgccagctgatgaaaacatctttccttt
ccaaccttgcaaaaccttttggaccttacttttaatgtaattcaacggtgccttcttgttcctagacagagaagacggacaaccgagatactttccatggt
ctgctactattttaaccttcaaaatactggctaattcgctccggatgttaactgcagttccaggcccaaacgctattgctgatttttttatagttgataatttgg
cctgtagcctcctcatagacttttagaagtctttgtaactccaaaaattccttcttgcgcgtttttaggaaaagcaaactgtcatctgcaaaaaagagatga
gaaatagtcggtgtcgttcttgcaatccgaagacctgaaatattctgagagatctcaacctttgttaagaggctagataaaccttcagcacacataaga
aataaataaggtgataacgggtcgccctgtctaagtcctcgagtgggcctaaaggtttcagaacagatgccatttaagtttacaaagttgataaattaaa
ttattacataatttaaaatatatgaacgaaatcagtggatactaaaaatcattactttgataaaagtgtagaaacaaaaaatgattaagagagagagaaa
aaaaatctattaaaataaaaagaagaaaaatgaggagagggaaaaacatttaaaaaatgaaaaaaggcaatgtcgcaccgatcggcaacttggatc
caaagccaaaagtcaccaaagtctctcacagtcacagtcacagtcacagtcctccacttcttcttcttcttcttctctctctctctctcactctctctct
ctctattacagagcttaccctaaatagactgaatccaaaatccaagacgacaatccacagcccaaaactgccggaactcccttctctcgaaatgacctt
tctgccctcaccctcttccccaattaccccttctccctcctcctccttctttgacttcctttcttccttctctgtctctcctc

>LcWRKY56 + Up_Stream_Len 2000

accaaaacatgcctttagtaattcaaataatttttgatgatatgaaaatcacgttaaaaagtgtaaaaccaaacatgaaattgattttaaatatttaaagatat
gtttcgagatgatttcaaatatgacaaaagcaattttacaaaatcacttccgaacatgcatttactttctgaaaaattttcgtcctcaattcccatttctcaaat
aaaaatataaggtaggtacgagaattgaaaattcccattctcgaccgagcccgaatccaacctttgccagttacccattctaaaattttgacaggaaaat
caaataataaaatgaggaaagaagtaaaatttaagcaacaaagagaagaaagaaaaatttaggggggaaaaaaaaaagataaaagcaaataataag
gaaggattagttgtaacaccttcccccatttgaaagctatagcctacaggtgttaggccattgtacagttgaagacctaaaagatctattacattatttttgt
attattacaattttaattttaaaaaaatgaatattattgctgattttaaggcaccaaatcaactctattattgtctattctgcaaaataaccaattattgtaataac
tagatttagagtagtgctttttttttttttttttttttttttttttttttttttttttttttttttctttttcattttgattttgaccatacagcttagatgtgttaatggaattagtata
aagtgtaagaagataaccaaaatgggccccttaaaaaaagatagatttttcaaaaactggaatgatttagttacaaaataaaatacccaaaaaaaaaa
aaaaaaaaaaccaacaaattgttagatacaataatactcattgtaaaatctaaaaaagaagctttggaaaataaataaatttaattaaattttgtttttttgtttt
ttgtttttttaaaattcaaaaatctgctttcactttttttgcttttgtttgttgaaattcaaagattccatccatttcctgtcgtccaaacacaatccctctccttccctt
ttcctcgaccattttcttctttctgggttattagaacagtgaaagttttatctttttttgtaaatttgtttggagctcaagaacagagttatggccattgttcatcaa
ttgggttttgaccagaatcaccaaaaagcgatttgagagagaaggaacaaggaagaaggaggtgggggtcactgtttttttccccccttcttccctactc
tccccatttccttcaatccctaaaatcaacccaccaaaatgatccataaacctttgttttccgatcataatttcgtttaatctccgattcagcttctgggatttt
gcccccttttgatttgatttgattttccatgtccgaacccgtttcttcttcttcttatctgatttgatttttgatttccttgttccttcgacctccaaatcactcaggt
aagtgattggactggttttgatttttggcaatttttgtttcgtttgttttgagtttcttcaattctgttctgtttctggaatttcgttttgttggcttctgtgtggaaat

gcttagatcttcacgggatcttcggatccgacactgttttggagggtttagtttcatgttttctgtcgattttcgtcttcttctttgtttgtttctggggtttgatg
ataaggttaaagaatcgaaaatcaaaatattcacatagagacaataagaaatggaaattacaacaatgaaaagaaaagacatacatgttcatgttttgtt
ctgctgtttctagctttttttcagctgagtttgttttggctctatgtcgtttgagagctgggattgttcatgcatgctcaaattccagcttccaatgtgaatgcta
atttgttgcaaagaagatcagattatcagattgttatatggttttcattgtttcccttcaggatttgtcgacattgctacgagattttcgagcttgatcgttcg
agttgta

>LcWRKY57 + Up_Stream_Len 2000

ggactaatttgacacaaccctgaaagttcagggactaaaaaaaacgtagacaagttgagctttgaactagataaatccatggtttctattaaaaaattga
ccacaaacaaagacaaccataaaaattcaaaagaccataaactattaaaattctgaatacatattaaattataattataattaaaaaaaaaaaaaaacaag
aaaaatagcctccattcccactacgtttaaaagtcacaagcttaacaatttaaatccataaaataatgacgactaaaaagtaaattattaatagcaattga
ctcgattaagattgaaagagttttataataatatatatatatatatatatatatattttaaatactcaataatcaaccattccaatcttacttatttttctatataaatat
aaataatttaatttatttcaagttatgaagcaacttgataatatttaataaatattctcatgattactcctaatccctcaattaggtcaaaaggtttcgagaaag
ttatcgcttactttattaaaagttgcattgcaccacacaataattaattatttaaaaaaaaaaatccgaaatatttttctttaattggcaccagctaatttccatac
tcttttaatgctttcaacacgtggcagaaaattcaaatgcccagccaattattaaataaggggatggaaaaagtcgtggtcaactcttttggtgaatctcc
accgtcggatcatcgccaatgaattcgacggttctggttgaatctaccgaaaataatacatcttttaatttaagtttggtcttaaaatttgcagatcattccat
ttttatgtacgagtttctaaggcccgtttgataaccatttgattttttatttttgatttttgaaaagtatacttgttttctcctaaatttcctaccatgactttcattata
attaatgttgcatgtgaattcctagtcaaaattggacttcgtttttgaaacaaggtagaagattaatactaaaaccaagaaatttataagtgaaagtatgtgt
gtataaacttaattttcaaaaacaaaaaccaaaaaccaaatagttatcaaacagagcctaaaatatttaattttgttattgaatatttacgtcaaaatgtgtat
tcaggttttttaatttgtaaaaattaaccatttaattttttttttcataaaaataaaaaattttagttcttatctttaaagtcacagaggtagtagtttagattaaatatt
cacggagtcgggtataaattcgttaaataataacactaactaatttttttatttttagatttaagactaaaaataataaattaaaaaaataatagcaaccaaaa
taaaatagaattaaaattttagatggaaaagaaatttgaacaataatttaatattataaagtttagtcaacatttggaggttaaattacgagaataatagaag
agctccacgtgatccccacttttggtcagctttctttatcaaaacacagtatggttcgtcgttttgtgcagttctatatcggtattttctcgcgatattttgaag
atatatcgcatccgaaatggagattttctagaagatgaaaattttaaaaaaccaatgtcaagtcaaaaaacgcaccgttttatcgtcttttacagcgagta
ctgtttggtaacgtacgtcgtcgttttaaaggatttggtcttctctcttctgatttttttgtttttttaaccctttcacagtatgccccactctgtacgtctctgaacg
aatctccaccgttggatcacgaatccactttgactcccaacctgcgtacccggtcatccggtcacctcgagcgccattaaatcatccccaacctccaa
ccttcggcttactttctccgaatactaatccttctcttctcttcaatcttcttcttcttcgatgatcttattcttgtagttcagatttttcatctctgtttctatttgcttt
gctttaagctcttaact

>LcWRKY58 + Up_Stream_Len 2000

Cttttctttcagcaccaacaaaagaaagttatgacgtgacaaaagtggtattgaattaaaaactaaaggtatcatacatttttgaaatttatctttaatttctt
cagtaccaagccaatggtattaaaaaaaaaaaaaaaaaaaaaaaaggataacctcaacttcaagggtaaaacgtataatttttttgggtctaaaatatttca
acaaatcaattcatacgaactttttatgaattaatttaaaaaagtaagactattttgcatgaacttatctttttaaaaaaaaaaaaagtgtccaattaatgaaatc
aatatatggtccatagcgaaccacgaactaacaatagcgcttctatatgtacagtcccaaatgagaagaatcgtatttaaaattaaattacaaatttggc
cttcaactttgagagtgttgtatcctaaatttaaaataaatcttatcctgtctctaaactttcaatgttgtatccgatacatcattgcattatatatatataatatcta
gtacatacttaaacttctaatttcatgtccaatgaatttttttaacttaaaaaagtatttctcatatgttagatatatatttattagactttctaagacttatttgagac
aatcaaaagtagatctataaagattagttgagaatgttgtctgcctaccaaagacgagtaagatatgatttcaagtctccccattcaaatcaacatccttt
gtcctccaaatctggtagaaattttcacaactcgatatagacttgaagttggtgttcatacaaaatttctagggacagtctatttgtgcttcagatattccac
acgagttttactataaaccccatcaaatgtgaattcattagacatttttttaaagttcccgaaacctattaaacataacaccaaaagtttaagaacatatttttg
agaagttagtagacacaatactaaaaatttcaggaaaacttctaatttaggggctttgtgaacccaacatgaataaaaacaaaattgtcacataataaaa
tattttgcaaaaattgcacaaatataaaaatatcaatctatacattaatagtgtactttatattatatatatcaaattatcaataacatattttttttttttttgaatca
atcaataacatattttatcaatatgtataaagaaaaaatacgagaaaaaagtatcatatttaaaaaattattttttttaacatacaacgaaatttgcaaatata
acaataaatttcatcactcattataatttctcgatattttaactccctacaaaattagtaagaaaaaaaaaaatttgattgcgcataccgatatgtaggaatctc
cttacataccacaattgaaatgttgacaactaagagagataaaaaaaagcttttcatttcttacatttctctttctcagttgtcaaaattttaatgggataagg
agatctcggtatgcagtcaaatattattaataaaaaaaaatcgaaaaaaaaaaaatcaaacttctctctttcccattcaaaacataatgagcaaaaaagt
catttcattttcaaaagcgcgtgggaccccaacacaccccaagtcaaccattccactccactttcaaagtcaacataaccacaaaacaaaaattaaaa
agcaagaaaaaataaaaataaaaaaaagtgatgttttatgaatgaatataatgaaggctggactcagaaaagaattccaaaaccgacttccagaaga
atgggacccaacacacagacaaagacatagactctttatatgcaatttttgctttttgcctctttttttattttatttttttattttatttttctctctatatataagtttctt
tctttagtcacttaattttctgcaaagtccttcttcttcttctccaaaaacccccatttttcttctttctttttttcttcttaattttcaataatcttaatcttttatttatttatt
tttaatctttctgatcttcaata

>LcWRKY59 + Up_Stream_Len 2000

aaatctgaattcccaactccagacttttacagaattactattgttttgtcggattctctctgatcactgacttaggcatcggagcgtgtgtggcaagcacca
caccggtgtgcatttttctctggttttgcaggtcacgtcttctctaacttctacaaattcactgttggtgtcacgtgaaggtcaagtgagtcatctgtccaaa
ttttggcatcaccaatttgtttcctttaagaggtaaagaaagaaataataatttggcaaagatttgatgaaaataattggaaaaaagtttatttgttattaagt
ggattttaataacagatcaaatctagcagcacttgattgggctgtctcaattaatgctaattctgcactcactacaaaatcttatggatgaatctcatttcca
accttaatttcttcacttaatactaaatcttcctcttcatcattctctccactcgacgagcttcctatgccatttccctatttttagcaacctattcatcgacagc

cacccactgatttttcttgatatttgcatcaatccacactcaagcgcatccttatacaaaattattttaatttattaagaaccaattttcaattttttttttttttttta
aagcagttctaggaagtcttaaattctttgtgtaaactgcaggattaacactcattcgatgattgtggacttggtaagggtaaaaatggatttggtgaaaa
tcaagtaataatcatccttttacacagcttgtttgtgccaaaagctatgagctacagtaccattcaacgctttgtttaagggaaaaaaaaattaatgccacta
gatgtttgcaccaaacgaagcctccttgttttaatcaaatcgtcttgctttaattaatataataaatcaagagtattctttttcggattggacctccaagaga
gcatctcaattttaaaatcctgaaaagtcaattcaaatttctgtttgggttcgtccaattattaaaaagggtaaaagaaaaaagaaaacttaggatccaatt
cactccaaaacagtacactgtactactacacataacatatggaaaatgttcaatgtttgtcaaacccaaaaggagattctaaaatttcagttcacacatttt
tctttttccaaattgcagaaatgcgcttccttttcttttccttgttgaagtagaagaaaaagagagagctttttagtttttttttcccattctttgaacgttgttct
cctcaagaagacttcgtggttctcgtataatgtgggcccggaaaagcaaagcccaatgagaaaccgaaaggaaagaagaaattgaaatttgaaaaa
taaattgttgaaggaagaaaggaatccaaggtgagtcgttggtggagagtatgatggaggaggttccggtatccggtaaaaaagatgggaaaccgt
atgggacccactaaaccccacctcctcactcaatgacttttgcattaaaagctgttaaaggtcaactctatcccactgtctccaactaatatcttgtccttt
atctctctctctcttcccgattttaccgagtcctcaatctaacggctattctcaacctttttgaccttatctatcccatctcatctctaatctctttacttcccac
aaatttaaaacccctccccaaccggttcaccctctaaatggccctttcctaaacaggagggtcaatgtgctgacaatcccccccttacgtggacctttctc
attggtttcactctttgacggcagatggaccagaatcgacagctcagttgcagttgcccgctgccttgcatagagagggcatcactttgactccaatcc
tttcccccaattctttttcatctttcctcttctctttttccccctttttgctttccctcttctctcctctccttttttcttttttcttttcaaattctcatcttttctccatataaa
tactctcctccttctgcccccccttctgttttcat

>LcWRKY60 - Up_Stream_Len 2000

caatgggaaaaagcaaatggccaaaaggagtctcttgtttcgatgtaacgtatggtcacttacaattaatgatataaccaaaataattatagtgatgaca
tgaactatatatatatatatatatataaataataggcaattatggaaaaatttaaaggaagagttgactattattattattaattcgatcgaaaactgacatttttat
aaaagaaatatcctatttacatacatatatacaataacatgggaaagtgagagattagtctctcaactaagatatctttttttagttcaacaacatttgaaag
tggagaacgggattatatttcaattaaaaacgttttcttttatttatttattttagtatgaaagtggcttaataaataggataatgggttgaaaagaaaaagg
ataatgaattcctcaaagaaaaaaattaataataatgacttggcatttagttgcatttttttccccatccccgtggtccctacgatcttagcctcttccttcaaa
atcaaaggaaccattaattgaagttcaccaccccatccactgatttggtgccaactggctctataaggtccactctgtcctatattttgtttcttttccgttttt
gcccctctcgtctcattgaaattacaaatccaacctccgacctgcgagtgggtgggtggttgatattttaaaaagtattgtacagcttattcctggttgttg
tgttgtgttgtgcttcactgcttctcacccactcctcatttaaccgcttccaactaaaaaaataataatcatttttccattttgtctaaagtgaataaatcttcaa
ataccatcacttttaccatctttttatcaaatttaagaccaacaaaatactctttttattagttatggaatggatcaagagcccatcgacaagtccacaatgc
acatagactagctcgtaggcttaccaaaatagttaaattaaggagacacagttgatgagacacatcacgagtaacctactaagggtccgtttggaata
cattttgatttaaatgtttttcaagaatgcatttttttgaaaaacattttgtttaaaaattgtttaaaatttaaacactcacatgtttggttgcattacctcataagtgt
ttttatacacaagttattgtatactaaacactctttttaaaaaaaagttggttttaagtgtttcttccaaaatggtttatttatcaatctcattttctaacaagtcatttt
tagtggttgccaaacactagaaatttttttcaaaatgattttttttcaaaattaaacacttgaaaagttaaaccaaacacaccctaagtttgaagagtataaat
agaaaaaactcatctcttatactactctagcttagcttgataccaccttaatcggagcgtaacatcaccagaccaactccactcttcgattagactcacgt
acatcagattcttgcatccacatgacccaaatcgaaactaactttaacatgagaaacatcctagtttgagctcaatgaaagcagtaagaattacaggttt
taacatgggttggaccttgttttaggtgtaccgaatgagtgaattaatggccaattttctccctcgcttccacttggcgaaacccatttttcatatgctgcta
gagtctgtgtttgtgagagaattaacaaaaagaaaatatcttggaggttaattttgtcatattcatggctttaatgtggtggcgtacttgtcttgttcaatcg
gagctataagctcagagattatagaacatggaaacaaaaaggggattaccaaatttttttctttggattaatttaattatccctttttcctttttcgtgtgttgcg
gcgtggactttttttttttcccatttctttcagctcagtttcagatcccgagaccaaaacccaggaaccgaaacttttggtatattcgagaaagagctgattt
gattgttttcggttggtttttgaaatttggtttt

>LcWRKY61 + Up_Stream_Len 2000

gagacaaagtttagcccttcctccacacacactattacaaaaggagaaaagaaaaaaaaagtctccaaaagttgcaaatgccaacacaatttcccaca
cacacaaaagtctggctgagctttttgtatttaactaactctcttctttggaataatctcaaataattacctagcaatttccagtgttaaatccattgatatgtt
gattaaactctattataatcttctctgtccatcaaaattttagctacctagaaaaagaataataataattaaatgtcatgatatgattactcctaatagctaaca
actccatgcaagttcaatatatatatatatatatattaaattttgtctctatcgtgtaacttaatgtgtaaaatggaaaaacttttgtagcgttgttccaacccta
tcgaataatttcaagagaaagagatatggtgtgaacctttttgaatatgaatccaacagttgtttgaaaaaacaacaaaatttaaagtttatgataaaaagc
caaatttagacataaaatttgaaagttttagggtatgtttggtttaacctttcaagtgtttaattttgaaaaaaattctagtgtttggcaaccactaaaaatgac
ttattaaaaaatgagattgagaaataaaccattttgcgagaaatacttgaaagcaattttttttttaaaagggtttttaagtatataataacttgtgtataaaaac
acgaggaaatgtaaccaaaacatttaagtgtttaaattatgaaccatttttatacaaaatgcttttttttgaaaaacccttaaactaaaaggtattcaaaacg
ggccttaagtctcgtttgataaccatttagatttcgctttttgaaaataaagcttataaacattacttccaccaatgagtttctttgtttttgtaatctactttttcta
ctaaatgttttagaaaacgaagctaggctttgaaaactaaaaacaaaaaaataggttttaaaatcttatttttgtttttagaatttgattaagaattcaaatgtt
tctttaacaaagataaataaaaataaaaataaaaataaaatcttaaagaaattttatgataacaatcttaatttttaaaaataaaaaataaaaaatcaaataa
ttatcaaacgtactttaagaactaaaatcttgaatgggaaaatttttagcactaccgcagtacgaatactcctcctattttattccttatcatgtcatcctcctt
tgaaaaatgatgtgagatatatttataaagataaaactatagtgtgaataaagagagtatgatacactaattttctctattacgaagtagccacgttgcagt
gcaagtggctgcttcggataatttctcccttgtaacaagtcgacctaagacgaaatgcaattactttgccacgtaagagagagagagaaaagtaaatgaa
aaccaaaaaccaagttgcaagttgtgaaagaaacaaaaggaaattcaaaatttagaacaagcccaacttgaatagaaacaaaacaaaacacatttga
tagcatagtagtactaattaattcttttaccaattcccagtccaaaacacacacaaacactgttgaaattgaaaccaaagtaaaaggaaatgggaaacta

tagaatttttcattttcttcttttaatttcttccatagtctttggtctaaattcccatcaaaattatatttaaaaaaaaaaaacccttcaaaccccaaaaaaagaag
attctttttcaatgggagaagaaggccaaagtcttcctctagacccaacccccaatttttgttgaaattctccgtcgaccatgaccaggtctctttttccca
ttttttatttaattcattcttgtaccgtattcatcaaacatgtcatttaatattcaatcaaaactttaccttatatttttcattcatacatacatagatacatatatatat
atatttgctgatcttaaagtttcgagatttgtct

>LcWRKY62 - Up_Stream_Len 2000

atggcttagcgactgtacttctatatataactcgatcttgagtgtttcgttggttaactttccatgtaatttattagtgaaattcactagaaattctctaattattc
aacggtatagtttaattaaaaaatgtgactttataattatttaaccaaatataagaataaaaaatggtttttttttttgaaaagataagaataaaaaatggtta
aattacaaatttggttcttaaactttcaacgttatatcattcattcatatatgttaaaaagtttgtaataattgtcaaacaatggcagatccaaaactttatagc
agtaggggcacaatttcatatacggttaaataaatttagtatatgaatgtttaaaagttgttctgggagaattcgaaccatagacctcacggtcattaatac
aagttttatgctaattgagttatgctcgtgttgacaattgtctctttaatcatgatatgtttaaacatttctattcagtccttgaagtttcaattttactttataaact
cagccactatacattaagctaattgcaagtaagttcaagatagtattaaaaatttgaaacttaatataaactttttaaaatcgatagatccacaccactctaa
aaaaaaaaattaaaaaacaaaacaaatttataatttaactagaaaacaaaaaaaaggtcctaaaactatatttcaacttcactctaaaataaaataaaaat
aaaaataaaataaaatagtaaaaccaacagctgtaagcctgtattttgcttttagaaaatattaatggcagcctcttccactatccaaagcatcacctttcc
gcaactgtgattacgtcagcatgcttacgtcatcatatctatcgctttcccaagtttcttcctctcaccttccaccccattcactacgatacttattgtctttctt
tccttttctcttaataattatttttctttttttattattatttaaaattacaaatttagtatttaataggtttatggaaaatattatgataaaaaaagatctaaaagacat
tccataagcctctaaattttgaattttgaattttgaatgataattatctacaaatagataagtagaaacttcgataccgtgttcaaaatttaacttctctcccaa
tagtaaaattataataatggtcaatagtcctactgattcgtcagaaggtaagtattttcattcaatcttatatttactagatttttaaactttcaattttgtaggtc
atggagaaattcaacatttttttaatttttcgattttattaattataaaattggaatttcaaaagatttattagacctatttaaaatttaaaaacttattaaacaaac
aaaaaaaaaaaaaaaatccagaatgaataagatatttgtatggttaagaacttataagtctcttaaactttaaaagcattcaacaggtctctaaactttcagt
tttatgtcaataattctttaaattttcaattttgtaactaatacgtgatcaacatattcaaaattctaaaaattaaatgatcttttagatataaaattaaaattcatgt
ctaatagaattttaaactttcaatttcgtattcagtagatacgtgaatttttaaaaatgtcaaataggccaaggacctattatacataaaattgaaaattcaga
tacctattcgataaaaaaattgaaagtttaagaaactatcatacatttttttaaaatttagggatctattagacataaaattaaatgttcatgaatttattgaatatg
aaattaaaagttcgaaaataatctatcaacagggactaaacttgtaatttaacgaaacttgtaatttaatactatatatatatatatatatatatataatatgtgt
ccctctattaaaatctcatcgccattgtctggtatttcatttacatccaaaatcggagagaaccaaatcgtgtggtttagactttagagatagaatcataac
