# Supplementary material for: Identification of WRKY Family Members and Characterization of the Low-Temperature-Stress-Responsive WRKY Genes in Luffa (Luffa cylindrica L.)
Source: Plants (Basel). 2024 Feb 28;13(5):676. doi: 10.3390/plants13050676 (PMC10935285; doi:10.3390/plants13050676)
Supplement: Supplementary file 1 [file plants-13-00676-s001.zip › Supplementary File S5.pdf]

>LcWRKY1 Chr01:35317600-35321483 probable WRKY transcription factor 57 isoform X1
ATGGACCCCGATCCTCCGCCCGACTTCTCCCCCGCCGATTCCTCCACTGATTGGACCAACTCACTCGCCTCCG
CCGATCCTGCTTACTTTTTCTCCGCCGATAGAGAATCCACCATCCTCACTGAATTCGGATGGAATTTCCACTCC
CACGACCTCCAACCTCCCCGATTCCTCCACTCCGACAACCCCGATTTGCCCGGAACCTCCGCGCCGCCGATC
GACGACGGCGTCAGCGGCGGACTGCAGAGCCCCGACGCTCCTGCTACGGCCGGATCGCTTAACGATGCAG
TCGCCTCGAATCCTTCATTGTCGTCGAGCTCCAGCGGAGAGCCGCCGGAGAAACAGCCTGAGATTCCGCGG
AAGGTCAAAAAGAAGGGGCAAAAGCGAATCAGGCAGCCACGATTTGCTTTCATGACCAAAAGCGAAGTTG
ATCATCTTGAAGATGGCTACCGCTGGAGGAAATATGGACAAAAGGCTGTCAAGAACAGCCCATTTCCTAGG
AGTTACTATAGGTGCACAAACAGCAAATGCACTGTGAAGAAAAGGGTAGAACGGTCGTCTGAAGATCCAAC
GGTTGTGATAACAACATATGAAGGCCAACACTGTCATCACACCATTGGATTCCCTCGAGGTGGACTGAACAT
GGCGCATGAAGCTGCTTTTGGAGCTCAATTTTCACCTCAAATGGCACAGTTTTATTACCCTGAAGCTCAGCC
ACTGCCCAGAAATAACCCTCCCAATGCCCACCAGCCGCAGAATTTGCCAACAATGGCATCATCCAATGCAGC
GTTTGAACAGAAAGAGGCAGCAAACTCACAGCCAGTGCCTTCGGATGAAGGCTTACTTGGCGATATTGTGC
CACCAGGAATGCGTAGAAGATGA
>LcWRKY2 Chr01:37098283-37100632 probable WRKY transcription factor 40
ATGGAACCTTCATCAACCATCAATACGTGTCTCGACCTCAATTTCAATCCTCCGCCGTACACCGCCGACGAATC
TCCGGCCACTAACACTGTTACTCAACTCAAAGAAGAGGCTCCGGCCGTTCTTGCCGAAAAGTTGAATCGGAT
GAGTTCGGAGAATCAGAAGCTGAATCAGATGCTAGGCCTCGTCGTTGACAGTTACAACGTTCTGAAAGATC
AGGTGATCGATTTGATGATTAAATCGAGGAAGCGAAAGGCAGGATGCGATGATTGCAACTTCAATCGGAGC
GGATCTGGCGCTAATGCTTTTACCGATCAGTACTGCGGCTGCTGTAGCGACGACGATTCGTGTCATAAAAGG
CCGAGAGAAAGCAGTAAGCCGAAGGTTATGAGAGTTTTGGTTCCGACGCCGATTTCCGATGCGAGCCTGGT
TGTGAAGGATGGATATCAATGGAGGAAATATGGTCAAAAGGTCACTAAAGACAACCCGTCGCCTAGAGCTT
ACTATAAATGCTCCTTTGCCCCAAGCTGCCCGGTGAAAAAGAAGGTGCAAAGAAGTGCTCAAGACCCATCCT
ATTTAGTGGCTACATACGAAGGGGAGCACAATCACATGAAACCTAATTCTGGAATTGAGTATCAATTAGTTGG
ACCAATTCACTTGGGTGGTTCAAAGCTGGATTCTTCAGTTCCCTCACCCTCGTCTTCAGTCAAATCTCCATCA
ATACCTTCTGTTGTAACCTTCGATCTAACCAAATCCCAATCCACCGACACTGTGAAAGCTTCAGTCCCAGAAC
TGGAAAGACCCTCATCAGCTCCATCCCAACAGATCATAGTTCAACAAATGGCTTCCCTTTTGACCAGAGACG
CCAATTTCACCAGAGCCCTCGCCACCGCCATTACTGGAACCATGGTAGAGAAAGAAATCTGGCGATGA
>LcWRKY3 Chr01:37109189-37111044 WRKY transcription factor WRKY76-like
ATGGGTTCGGAGGTGGAATGTCTCCGAATAGAGCTCGAAAGGTTGAGGAAGGAGAATGAAGCTCTGAAGT
TTATGCTTAGAGTTGTGAGCATGAGGAATTTGGTTTCTCAAGTGGGTGCTTTTGTAGAACATCACAATGATAT
GAACCAAAATTCAAACATGAGGGCGAGAGCAGACCTTCAGGTTCCGCTCGCTCTCGGGGCATCTTCCACGA
CCCAAGCATATGTGAGAACTAACTTCAAGGACCCTGCTTTGATGGTGAAGGATGGATATAGATGGAGAAAAT
ATGGGCAAAAGATTACAAAGGATAATCAATCTCCTCGTGCCTATTTCAGGTGTTCTTCTCCGGGATGCCCCGT
GAAAAAGAAGGTACAAAGAAGCTTGAAAGACAAGTCTATGGTCATAGTCTCATACGATGGCCACCACAACC
ATGAAAATGACTCTCCTTCTCCAGTTTCAACCTCTGAACAACTGTCTTCGTCTCCGTCTTCTCAAACCGTCATC
CACCGTTTGCCACAGCCGCCGCCTCCGCCACTTTTAGAGGAGAGCAACCGTGTTGTTCAACCAGCGTCGCT
CGATCTCGATCTCGATCTCGATCTCACTCTTTCCAGAGGCAACAAAGAAGATAGAAAAAAACCTTCCAATTTG
AAAGAAGAATTGGAGGGAGAAACCCTAAACATAATCAAAGATTGTGCTGAATCTTTGACTCAAAACTCTGG
TTTCACAGTGGCTTTGGCTGCTGCAGTTGCTCGTTCCATTTCTGACCAACCTAGGTCAATGGAGAGTTGA
>LcWRKY4 Chr02:886468-887701 probable WRKY transcription factor 27
ATGGCTGCCGGAAACGACGACTGGGACCTCTCCGCCGTCGTCCGGAGCTGCAACTCAGCCTCCTCCACTGA
CCCAACAGCCGCCGCCGCCGCAGAAACCGCCTTGTCTTGTCTCGCTTCTTTAACGTTTGACGACGATCCAAA
CGACGTCGCATTCTCCTTCTCCGACATCCTTCAGCCCCAACAACAGCCCAACGGCTTCCATGAATTACACCAA

GCCTTCGTCTCCTTTCTCCCCAACCCCGCCGCCTCCGCCGCCGTCACCACCGCCTTGCCGGAAATTCCCAACC
CCACTCCGAGTCGCCATTTTCGGCAGGGAGTTAAACCCATTCGCCCGCAGCCGGATCCCGGGGCGGCGGC
GCTGCAACCGCACCACCGGCAGCCACCAAAGAATCAACAGAAGAGAACCGTATGCCATGTTACTGCAGATA
ATCTCTCAACAGACATGTGGGCTTGGCGTAAATATGGGCAAAAGCCCATTAAAGGATCTCCATATCCAAGGA
ACTATTACCGTTGCAGCAGCTCAAAAGGGTGTGGAGCAAGAAAGCAAGTTGAACGGAGCAACGCCGATCC
GGAGACTTTCATCATCACATACACCGGAGATCACACCCACCCACGTCCAACTCACCGGAATTCCCTTGCCGG
AAGCTCCCGAAACAGGTCGTCCTCGTCGATCACCACCAAAAACACCACCACGGGAGATCCCAATCGGTCGT
TGACCGCCACCGCTGTCATCGGCTCTTGCTCCTCTCCGGCAGCCTCTCCGATGACACCGCTCGATGGCGGTG
GTGAGAAAGAGGCGGAGATGTTTGAAGATATGGTGATCGATAGTGACGATGATGACGACGATGACGATATT
CTGATTCCGAACTTGTCTGTGAGAGACGAAATCTTCGTCGGCTTTGAGGAGCTTGGTTGTAGCCCTAGAAG
GCGGCCATCGTAA

>LcWRKY5 Chr02:4395721-4396925 probable WRKY transcription factor 27

ATGGAGGTTTCTTCTGATCATTCTCTCTCTAAAGCCAAACCCAGAAGAAGATGATAATCTTTCAGCTCAAC
TCACACCAGAAGCAACTTCCAAAAAAAGAAAGGTCCTCCAAAAGACTGTGGTTACTGTAAAGATTGGGTCT
GGAAAAGCTGCAATAGGGATTGGGAAGATGAAGAATGAAGGTCCACCTCCTGATTTATGGTCTTGGAGAAA
ATATGGCCAAAAACCCATCAAAGGATCTCCATATCCAAGGGGTTATTATAGATGTAGCACAACAAAGGGCTGT
TCAGCAAAAAAACAAGTTGAAAGATGCAAAACAGATGGTTCAATGTTCATCATAACATACACTTCAAGCCAC
AACCATCCAGGCCCCAACATCTCCACCCTCCATTTGGATCAATCCCAAGAAGAAATTCAACCACAGCCGTTG
GATCAAGATCAAGAGCAAGATCAGAATAATCTTCATCCAAATCAAGGGCTAGAAAAGCAAGATCAAGATAAA
AATGATGAAAAAAACAGCATTATTCCAAGTGATGATGAAGAAGAAGTAGAAGAAGAAGAAGAGGAAATTG
AAGAAGAAGAAGAAGAAGAGAAAAAGGCAATGGAATGTTTGGAAGAACCAAAAGTTTCTTCATGCTCTCA
TGAGCTGATAAACTTATCAGCAACAAACAAATCAGAATTGGAAAATCATGACCATTTCTTTGATGAGCTTGAA
GAACTGCCCATTCCTCCACCTTTCTCAAGCTTGATGAGATCAAGCTGCTATTTTTTTGATGAAGTGATTAGGA
TTCCTGCTGCCCCTTCTTGA

>LcWRKY6 Chr02:4472340-4476225 probable WRKY transcription factor 23

ATGGAGAAGAAGAAGGAAGAGATTATAAAGATGGAGGACACTAGCACGAGCGGCGGCGGCGGCGGCGG
CGGTGGTGGGTATTTGTCATTTTCCGATAATATGATAGCAAATAGTTTCTTGGATTTTTCTGATGGTGAAAAAT
GCTCTGTTGGGTTCATGGAGCTGCTTGGTCTCAATAATCATGAGTTTAAGGATTACTGTTCCGAGGTTTTCAA
TCCTCCGGCCACCCCCAATTCCTCCTCCGTCTCCTCCGCCTCTAGCGACGCCGTTAACGACGACCCCACCGCC
GCCGATCACAAGGCTGATGATCATGACCTACAACAACAACAACAACACAAACCCACTAAACAATTGAAGGC
AAAGAAGACGAATGGAAAGAGAGAAAAAGAGGCAAGATTTGCATTCATGACGAAGAGCGAAGTTGATCAT
CTAGAAGATGGATATCGATGGAGAAAGTATGGCCAAAAAGCTGTCAAAAACAGCCCTTTTCCTAGGAGCTAT
TATCGTTGCACTAGTGCAGCTTGTAATGTAAAGAAGAGAGTTGAACGATCATATGCTGATCCAACCATTGTGG
TGACCACCTATGAAGGCCAACACACTCACCCGAGCCCGGTCCTCACCCGGTCCGCCCTAGGCATTTCCGTTC
CACCACCGCCCTCCACCGATATATCGGGAGGAGGGTGTGTTGGCATTGCGGCCATGCCATGGCTCAAAGCTA
GCAACAACTCTCACGAAGGCAACATTACAGCAATATCTCATAAGTACCTTCAAAACCCGACCTTCTTCACTGC
CCAACATATGGATGCCGATTACAACCGCAACCTCATCGGAGCAGCGAACATGGCCGGATTCCTTCAGGAGA
AACGATTCTGCAACCCAAATCCTGCCTTTCTTGCAGACCATGGGCTGCTTCAAGATGTTGTTCCTCCCCACAT
GCTGAAACAGGAGTAA

>LcWRKY7 Chr02:6294188-6296022 probable WRKY transcription factor 40 isoform X2

ATGGCTGCTTATTCCCCTTGGTTGGATTCCGTCGACACTTCTCTCGATCTTAACATTCATCCTCTCAGATTCTCC
GGCGAAGCTCCGAAGGAGAGGAATTATTTGGATGTTGAGAGGAAGGTTTCTGTGAAAGAAGAGACTGGT
GCTTTAATGGAGGAATTGAAGAGGGTGAGTGCAGAGAACAAGAAGTTGACTGAAATGTTGACTGTTGTGT
GTGAAAATTACAACACTTTGAGAGGTCATTTGATGGAACAGATGAACAAAAATGGAGAAAAGGAGATTTCT

TCTTCAAAGAAGAGGAAATCAGAGAGTAGCAACAACAATAATATAACTGGAGTTAATGGAAATTCTGAGAGT
AGTTCAACTGATGAAGAGTCTTACAAGAAACCAAAAGAGGAAACCATTAACAAATCTTCTAAAACCACTAGA
ATTCAAGTCAAAATTGGAGCTTCTGATTCAAATCTGGTCGTTAAAGATGGATATCAATGGAGAAAATATGGCC
AAAAAGTCACTAGAGATAATCCCTGTCCTCGAGCTTATTTCAAGTGCTCTTTTGCTCCTAGCTGCCCTGTCAA
AAAGAAGGTTCAAAGAAGTGTAGACGATCAATCGATTCTGGTCGCCACATACGAAGGCGAACACAACCATC
CAAATCCATCTCAGATGGAGGCTACATCCGGCGCCGCCCGCTGCGTCAGCATGACTCCGGCGGCGCTCACG
GCGGCGGCGGGGGGATCCTCGTCGGCGGCGGCGGCGGTTTCGCTCGACCTGGCAAAGCCCAAATCAAGC
ACCGAAGGTAAAACCACCTCCAGCCCGAGATTCGAGTCGCCGGAACTGCAGCAATTTCTCGTCGAACAGAT
GGCGTCTTCGTTAACCAAGGACCCCAACTTCACGGCGGCGCTCGCGGCGGCGATTTCGGGAAAAATTTTCC
CGCATTAA

>LcWRKY8 Chr02:11911386-11912916 PREDICTED: LOW QUALITY PROTEIN: probable WRKY transcription factor 48

ATGTTTGTTAATTGTATGGAGGAGATTGAGAAGAAGAAGAAGGAAGAGTTAAAAAAAAGAGAGAATTTGA
ATTGGATGGGAAATTCTGGTATGTTATTCTCCGATTCGATTCCAAGTGGCATGTTCGATTTCCCACTGGACTCC
ACTGAGTATTCTTCCTTTAAGCCCTCCACCTTCTCCGATTTGTTGGCCATCCAAGATTACAATCCTTCTTTATTC
GACCTCTTCTCTCCACCGTCTCTTCCTCCGCCGCTGCCTGAATCCTCTGAGGTTTTGAATACTCCTCCCACTCC
CAATTCCTCCTCCGTCTCCTGCTCCTCCAACGAAAGACCCTTGGACGCTGATGAAGTCGATCGGGATAACTC
GCCTTTCAACAAACAGTTGAAAGTGAAAAAGAAGAATCAAAAAAGGGCAAGAGAACCGAGATTTGCGTTC
ATGACAAAGAGCGAAGTGGATCATCTGGACGACGGTTACAGATGGAGAAAATACGGTCAAAAAGCCGTTA
AAAACAGCCCTTATCCGAGAAGTTATTACCGTTGCACCACCGCCGGCTGCGGCGTCAAGAAGCGAGTCGAA
CGATCCTCCGACGACCCTTCCACCGTCGTCACCACCTACGAAGGCCAACACACCCACCAAAGCCCAATCATG
CCACGTGGAGCCCTCTCCTCATCCATCTCCCCCGCCGCCGCCGCCTTCACCGCCACGCCGCCGCTTGTTTTCC
CACAGTCGCAATACCCCCACCACCACCAACAGTACACGTACACTCCGGCGCCGCCCATGGGATTTGATCCGG
TGTTCCATAGCTTGGGAGAAGAACGGCGTCGTATCGGCGGATCCTCTTCGAATTCCGATTCTTTCAAAGACC
ATGGGCTTCTACAAGATATGATCGTACCCTCCTCCTCCTCCTTACACATCCCCAAAGAAGAGTAA

>LcWRKY9 Chr02:45569374-45571776 probable WRKY transcription factor 21

ATGGAAGAGGTTGAAGAAGCTAACAGAGCTGCCATAGAGACCTGCCATGGAGTTTTGAATCTCTTGGCTCA
TCAGCCCCTTCAAGACCAAGTTCAGTTGAGCAACTTAATGGTGGAAACTGGAGAAGCAGTTTTCAAGTTCA
GAAAAGTGCTTTCTCTTTTGAATTCTGGCTTTGGTCATGCAAGAGTTAGAAGGTTTAACAAGATTCCTCCTCC
TCTCCCCAATTTGCCCCAAAGGGCCCTCTTGGATTGCCCAAATTACAGGCCAGATCCTCCAAGCAAAAATCTT
CACCCTTTTCAGCCTGGTTTGAATGGTAAAGTTTCAATTTTTATGGGAAACCCAGATTTGGAATTGGGTCAA
AATGACAAAAACTCCCTCCAAATTCCCAAACAAGCAGCTCCATCTTTAAACTTCAGCTTCCCTCAACAACAAC
AACAGCAGCACCAACAGAGGCTGTTGGCTCATCAGAAACAGATGAAACAGCAAGCTGAAATGGTGTTTCTT
AGGAGCAACAGTGGGATGAATAATCTGAATTTTGACAGCTCTAATTGCACATTGACAATGTCATCAGCTAGAT
CTTTCATTTCCTCCTTGAGTATGGACGGTAGCGTCGCCGACGGGAGCTCGTTCCATTTGATCGGACCGTCGTC
GACGTCGGCCGATAACAAGAGGAAGTTCCCTGGGAGGGGAGATGAAGGGAGCTTGAAATGTGGAAGCAC
TGGTAAATGCCATTGCTCAAAGAAGAGGAAACATAGAGTGAAGAGATCAATCAAAGTGCCTGCTATAAGTA
ACAAACTTGCAGATATCCCTTCTGATGATTATTCATGGAGGAAATATGGGCAAAAGCCAATTAAGGGTTCTCC
TCATCCGAGGGGTTACTACAAATGCAGCAGCATGAGAGGTTGTCCAGCGAGAAAGCACGTCGAGCGGTGC
CTAGAAGACCCGTCGATGCTTATTGTAACGTACGAAGGCGAACACAATCACCCGAAAATGTCGACACAATCT
GCACACACTTAG

>LcWRKY10 Chr03:37041515-37049862 WRKY transcription factor SUSIBA2-like isoform X1

ATGCAGTTCATGGAGGACTATGGCGACCACACAAGACCCTCCACTTCTTCAACCCCTAAATTTGCAGACGAT
GAGGTTCTTGTCGATGCCAATCCTGGAAGCTTCGCCCAAAGAAGACCTTCCAAGAGAGGGAGAAATGGGT

TTAATGCACCCAAGAACAATATGCCCACGCCCAGGTTTCGTACTACCAGTCCCCCCTTGGCTTCGCCCATGGT
TCCATCTCCATGTCTCACTATTCCTCCTGGAATCAGCCCAACTGTCTTGCTTGACTCCCCAATTATGCTGCTCAA
TACACAGGATCTGCCATCTCCAACCACTGGCACTTTTCCCATACATCCAATAAAAGATGAGCATTCATTGTTGA
ATCCTGTAATGCCTGAAGATGGAAGTTGCCACGGCAGTGAGGATTCCTACTTCAGGTTCGCACCTCATGGGG
ATTCTCTTCAAAATCTTTTGAGGCTTGAAAATCAGGAAGCTGACATCGATTATCAAGCCTTTGAACCAGAGA
AAACGCTGATGGATTTTGAATTTCTTGCCGATTTTCCAAAAGAGGCTTCTGTATTGAAATATGAAGTCGCTTC
TTCCACTGATAATGATCTTTTTGATGGCAAGATTGTTAATGACAACTGTGACGATATGGAAAGTTGTCTTACTT
CTATAGCTAATAATCAATCGTCCATACCTGAAGAATCAACAAAAAGAGAAGACATTGAGACGCAGCACCCTTT
AGAAGGCGAACAGAAAGGGTCTTACATTCCTATGGGAATCTTAAGGACATCAGAAGATGGATACAACTGGA
GGAAATATGGACAGAAACAGGTCAAAGGTAGTGAATATCCAAGAAGCTACTATAAATGTACACACCCAAATT
GTTTAGTAAAGAAAAAGGTGGAGCGATCTCTTGATGGTCAAATAACAGAAATCATCTATAAGGGTGCTCATA
ATCATGCAAAACCTGATCCCAATCGTCGAGCTGTGCTTGGATCAGTGCCAACTCCAGATGATACTCCAGAAAT
TGGTGAAGGTGGTGGAAACCGTGCCAAAGTTGAAGCTGGGCTGACATGGAGAAACACTCAATATGGTGTT
AAGGATATCAAACTTATCTCAGATTGGAGTGTTGGTGGTCTAGAAAGGACGTCCTCAGTATCAGTTGTGACT
GAGCTTTCTGATCCATTGTTAAATCCCCAAGGGAAAACTGTTGGGGCTTTTGAATCTGTGGGAACTCCAGAG
CTTTCATCCACACTTGCTAGTCATGAAGATGGTGGTGGTGGTGATGATGATGATGATCTTACAACTCAGGGAA
GTATTTCAGTTTGCATGGAAGCTGATGATGTCGAACCTGAATTGAAAAGAAGGCGAAAAGAGGGCAGTTCA
ATTGAGACAAACTTGGCTTCAAGGTCTGTACGTGAACCAAGAGTAGTTGTTCAAATTGAAACTGAAGTTGA
CATACTTGAAGATGGGTATCGATGGCGGAAGTATGGGCAAAAAGTTGTCAAAGGAAATCCAAATCCAAGGA
GCTACTACAAATGCACGAGTGCAGGCTGTTTAGTAAGGAAACATGTTGAGAGAGCATCACATGATCTGAAAT
GTGTTATTACAACATACGAAGGAAAACACAATCATGAAGTGCCTGCAGCAAGAAATAGCAGTCAAGTAAACT
CAAGTAACGGCAATGCACAACCTTCTGCTTCTCATGTTCAACCGAACATGGGTTTATCCAGAAACTCGAACG
TCCCAAAGTCCGAAACACAAATTCAGGATCTAGCTGCCCAGTTTTATCCAAAACCTGAATTTAATCATGACTAT
CAGAGGTCTGGTTGTTTTGATAGTTTTAGCAACGATATGAAACTTGGAGCTCCTTCATTCTGTCAAATGAAGT
TCCCACCATTGCATAACACCTTGCCTTATAGCTCCTTCGGATTAAATTCAAAACATACTGCAACAAGTATTTCT
GGTTCTTTGGCATCCATGGTCCCGGACTTCCCGATTTCCTTGCCCTTGAACCAAAACCTTTCAGCTGCTGGTT
TTGATTTTGCCAATGGAAGACCAATACCACCATTTCAGGTTTTCCTGGCTGGTCAGCAGCTGAGAGAGACA
GATAGATTCCTCACGCCCAAACAGGAGCACGATGAAGAAAACATCTGTGCTTCTTTCCAACCAATTGTTGAT
AGTTCGAGTGGATCTTCATCGTCATCGGTCTCATCGGTTTATCAACAAATCATGGGAAATTTTCCATAG

>LcWRKY11 Chr03:49118873-49123457 probable WRKY transcription factor 72 isoform X1

ATGGAGGCTGAACCTCTGCCAACCAAAGATGATAGAGTTCAACCAATTGGAGGAGATCATGAAGAAGCTGC
TGCCAAACAAGATGTTCTTCACAAGGTGACTTTTATGGCTGACTTGGAAAAGCCCTCCATGGAACCCCTCTC
AGTCGCATCAGCTTCTTCAACTTGGAAGGAAGAGGATGATCATCACCATGAACAAAGAATCAAAATGGCGA
AAGTTGAAATGAGCCAAGTGAGAGAAGAAAACCAGAGGCTAAAAAAGAGTTTGGATCAAATGATGAAGGA
TTACGAGAGCCTGAAAAGGCAGTTCCACGACATAACAACTCAAAGGGAAGGGAAAAAATCCACACAAACA
AGTGCGAGTACCATTAACAATGATGAGGTTGAAGAAGTGGACGACATGGTTTCACTCACATTGGGAAGATT
CTCAAGCTGCGATCAAAACAAGAACAACACTTCCTCAGAAAGAAAATTGGATCATAAACTGTTGGAGCTGA
AAACCCCATCAAATAATGATCACATTCAAAGCCCAACTGATAGTGAAGCTAAAGATCAGGAAGAAGCCGGA
GAGACTTGGCCGCCGAGTAAAGCGTTGAAGGGTTTGCCAGCTCCGGCGACCGGAGAAGATGAAGTCTCTC
AGCAGAATCCTCCCAAGAAAACTAGGGTTTGTGTGAGAGCTCGGTGTGATACTCCAACGATGAATGATGGAT
GTCAATGGAGGAAATATGGACAGAAGATTGCAAAGGGAAATCCTTGCCCTAGAGCTTATTATCGTTGCACTG
GTGCACCTTCATGTCCAGTTAGGAAACAGGTACAAAGGAGTGTTGATGATATTTCCATACTAATCACCACCTA
TGAAGGAACTCACAACCACCCTCTTCCTGTTTCAGCCATGGCCATGGCTTCCACGACCTCCGCGGCTGCCTC
GATGCTCCTCTCGGGCCCTTCCTCCTCCTCCACCTCTCGGCCCGGACTTAACCCTTCGTCGTCGATCTCCACC

GCCGCAAACCTCCATGGAATGAACTTGTACCTCTCCAACAACACCAACACAAAGCAATTCTACTTGCAAAAC
TCTTCAATGCTATCTTCTTCTCTAAACCACCCCACAATCACTTTAGACCTCACTTCAAACCCTCCCTCAACTTCC
TCCTCCTCCTCTCCTTTCCAATATAAAATCCCTTCAACTTATCCCCAAAATATCCCTTCACCAGCCTTGATTTTG
GCTCTTCTCAACCCAACAACTTCATGTCTTGGAACAATAATAATAACAACCAACCTTATAATATCACCAAAAAT
GCAATAGGAATAGCATCATCAGACCTTTCCAAACAGCTCCCTCTCCATAGTAACATCTATCAAGCCTGCCTTCA
ACATTTTGCCAAACCTTCAACTCCGCCGCTCCCGCTCCCGCCGCCGTCGCCTCTGCCCGACACGATAGCGGC
AGCAACCAAGGCAATTACGTCGGACCCGAGCTTCCAATCGGCATTGGCGGCCGCCCTCAGCTCGATCATCG
GCGGCGAGGGAATCCAGCCTGTGTCTAATGTGTGTGGATCAAGTATGGGGTTTCAGGGTCCTCCATCGTTGA
TATGCTCCAGCTCCAAGAGTCCTTCTTCATCTCCTGGTGATAGTAGAGACAATAATACAAAATGA
>LcWRKY12 Chr03:51487311-51488955 probable WRKY transcription factor 11
ATGGCCGTCGATCTCGCCGCCTTTCCGCCCAGCTTCGACCATCAAGCCGCCATCCAAGAAGCCGCCTCCGCC
GGTTTACAGAGCATGGAGCATCTAATCCGCCTCTTATCCAAACAACAATCCTCTCCGACTCTCAACCACCCCC
ATCTCGACTGCTCCCAAGTCACCGATTTCACCGTCTCCAAATTCAAGCGACTCATCTCCCTCCTCAACCGGAC
CGGCCACGCCAGGTTCCGTCGCGGCCCCTCCCCTGTTTCCGATTCCCCCAATCCCGTCCTCAATTCCCTCGAT
CCGCCGCTCAAATCCCCCAATCCGACCTCCACAGTCGATTTCCTTAAACCCAATCTCACTCCTCCGCCCAAATC
GCCCGATTCCAGGGCTCCCACTGAGTCCACCACGACGTCGTCTTTCATGTCTACCGTCACCGCCGACGGCAG
CGTCTCGAACGGGAAGCTCGGTCTCTCACTCTTCACGACTCCGGCGGTCTCCTCCGGTAAGCCGCCGCTGTC
GTCGAAGAGAAAGTGCGAAGATAGCTCTCGATTGGCTTGCAAGATCTCTTCGAAGCCTTGCCACTGCGCGA
AGCGAAGGAAATCTGGAGATAAGAAGACGGTACGAGTTCCGGCGATCAGTTCTAAGATCGCCGATATACCT
TCCGATGAATACTCCTGGAGAAAGTACGGCCAAAAGCCCATCAAAGGCTCGCCCTATCCCAGAGGGTATTAT
AGATGCAGCACCGTCAAAGGATGTCCGGCGAGGAAGAAAGTGGAGAGGGCTCGTGACGATCCGGCGATG
CTCCTCGTAACTTACGACGGCGATCACCGTCACCCGCAGGCCACGGTGCCCAGGGCGGTGACCGATGCCAG
AGTCGGCTTCGTTTCCCAGAAGTGTTGA
>LcWRKY13 Chr03:52697842-52703679 probable WRKY transcription factor 32
ATGGCGGAGCGCGAAGGCTTCGAAACTGACCGACTCGGAAGCTCGAAGGCTACTGCGGGACAAGAGGAT
GATGAGGAAGAAGAGATGGAAGTTTCGGAGGATGAATCAGAGGTGGAGTTGGAAGAAGGAGGAGGAGT
GAGTGAGTTGCAACCTACTGAGTTGAGGGCCGGTTCGTCGGTTAGTGAAGCTGCGGTGAGGGGTTCTCCT
TCTGAAACCCTAGCGGCGCCTTCTGCGAATCGGTCCTCGGAGAACGGTCAGTCTGATGGTTTGCCTGTCAAT
TCTTCTGCGCAGTCACTCGAAGGAGCTGAATTGAAGCAAGCTCCATCTTCCCGCAATGAGCCTCTGGCTGCT
GAAGCAACCCAGACAGATCAGGTGCAAGAACAAAGCCAACTTCAGGTGTCAACATGTAAAGGAACTGATT
CGGGTCGATCGCCAACTTCAGTTACCCAGTCTATCTCATCCTCCACAAGTCCAAGTTTGTCCGAACATAAACT
GTCACCTAAGAAGGTCCATAAAGAATGTAAGCCAGAACCAAGCCAGAAAAGTTCTTCCGATCATAAAACTGC
ATTATCTGTTCCCAATGTTAGGACACCTGCTTCTGATGGTTACAATTGGAGGAAATATGGTCAGAAGCAAGTT
AAGAGTCCCAAGGGTTCACGTAGCTATTACAAGTGTACATATTCTGAATGTTGTGCTAAGAAGATTGAATGTT
GTGATCACTCAGGCCTTATAACAGAGGTTGTTTACAAGAGCCAACATAGCCACGATCCACCTAGGAAGATTA
ACAACCCCAAGGAAAGTAAGCTTGTGCCATATGTTGAGCCTGTAGTTAAAAAAATCATTGCAGAACATTCCA
GAAGAATAATTAATGATTCAGATCCTCCTACGCCTTCAAAAGAAACTGTACGGGAAACAGCTTTAGTCCTTGA
AAGAAAACGACAGCACTCAAATGACTCTGATGGAAACGATGAATTTAAAATTAAGGATGAGAATGACAATG
ATTCTGAGACAAAACAAAAAGTAAAGAAAAGCAGTGGGGGATATTCAGGTACTCCCTTAAAACCTGGAAAG
AAACCCAAATTTGTGGTACATGCAGCGGGTGACGTGGGAATCTCGGGTGACGGATACAGATGGCGCAAGTA
TGGTCAGAAAATGGTGAAAGGCAATCCTCATCCTAGGAACTACTACCGATGTACCTCTGCTGGGTGTCCAGT
CCGTAAGCACATCGAATCAGCTGTAGAAAATCCAAATGCAGTGATTATAACATACAAGGGAGTTCATGATCAT
GACATGCCTGTACCGAAGAAACGACACGGTCCACCAAGTGCTCCTCTTGTAGCTGCTGCAGCTCCAGCCTCC
ATGAGCAATACACAACCGAAGAAAACAGATGCGGTTCAGAGCCAAATTTCTTCAACACAGTGGTCTGTGGA

TGCTGAAGGAGAGTTAACTGGTGAGGCCTTGGACCTTGGAGGTGAGAAGGCAATGGAATCCGCTCGAACA
CTTTTGAGCATTGGATTTGAAATCAAGCCTTGCTGA

>LcWRKY14 Chr04:41393674-41395779 PREDICTED: probable WRKY transcription factor 49

ATGGAGTCGTTAGCAGCCATTCCCTGGTCAGAGACTTACAACGCCACCTCCGAAGACGACCTCCTCGGCCTC
ACTGACCTTCTCCACGACGACGCCTCTCCGCTCTTCCTCCTCCCCCAAGACGTCGACGACGACCGGCCCACC
AAGCTCCCCGTCCCCGGTGGCCCGGCATACTTCGGGCCGACGATTGAGGATATTGAAAATGCACTCTCCACT
GCCCCGAGATCAAAAGACTTGCAGTCCAACACCCACATTTCTCATGCTGTTACGGGGTTTTCGATTGTGGAG
AGGGGAAGTTTGAATAAGGTTGAGCATAAGTATAGCCTAAGAATCAAGAGCTGTGGTGGGAATTTGGTGGC
TGATGATGGTTACAAATGGCGAAAATATGGTCAGAAGTCTATCAAAAATAGCCCCAATCCTAGGAGCTATTAC
AGATGTTCAAATCCAAGATGTAGTGCAAAGAAACAAGTGGAGAGGTCCATAGAGGATCCAGACACCTTCAT
CATCACCTACGAAGGCCTCCACCTCCACTTTGCTTACCCATTCTTCCTAATGGGCCAAACCCCACAGGCCCAA
TCTCCAACCAAGAAGCCCAAGACGATCAACCCAGACCCCGAAGCCCAATTTCACGAAGCCCACAAGAGCCC
AACTTTCATCTCTCCAGGCCCACTGCCACCCGACGACCCAAAGGAAGAGACGGGCCCACAAGGGTTGCTC
GAGGATATGGTCCCTTGGATGATCCGAAATCCATCGACCAATAACAACACTCTATCGAATTCGTCCTCGTGTTC
ATCCTATCGGTCGCCTCCAACGTCCCCTCCATCACCGTCCACGTGTCCTACCTTCTTAGCTTCGTGCTTTTGA

>LcWRKY15 Chr04:45650053-45652243 PREDICTED: probable WRKY transcription factor 12 isoform X2

ATGGGATCTCCGGCTTCCGACGAGATAATGGTGAACCAGTTGGTGAGTCTACTGCAAGCCGAGCACGCCGC
CGCCGCTATTACCACCAATTTCAATTTCTTTTCATTCCCTGAAAACACCACCGCCGTCGACCTCCACCAACAAT
CATCCCTGAAACCCTTCTTCTGTACGAGTACGGAGCCTCCTCATTATTTGTCCCAACAATCTCAGCCGCCCCTT
CTCTTTTCTCTCAAGCCGCCCTCTCTTCTTCACTTTCCTTCCTCCCTCCCCTCCACCACCGCCACCTCCTCCGAT
CTCTGGGATGTGGGGGAGGGGTTTGTGAGGGGGAGAAATGGGAGCGGAGACAGCGGTGGCGACGAGG
ACCGTAAGCCGCCGCGGTTGAGGGTGTCGGCGACGGCGAAGATGAAGAGGATAAAGGGGAGGAAGAAA
GTGAGAGAGCCAAGATTCTGCTTCAAAACCATGAGCGATGTGGATGTTCTTGACGATGGTTACAAATGGAG
AAAGTACGGCCAAAAAGTTGTTAAGAACACCCTCCATCCCAGGAGTTACTATCGTTGCACACAGGATAATTG
TAGGGTAAAGAAGCGAGTAGAAAGATTAGCGGAAGATCCAAGGATGGTGATAACGACCTATGAAGGAAGA
CACGCTCATTCACCATCTCATGATAATTTAGAAGAGACAATGGGTCAGTTACCGTCGGGTCACTTTAACAATT
TCTTCTGGTAG

>LcWRKY16 Chr04:45775210-45777257 probable WRKY transcription factor 51

ATGAACATGAACTCCCCTCAAAACCCTACCTTCTTCAACCACCATCAATTTCAAGACTCATCCTCCTCATTCAT
GGATCTCCTCGATTTCTCGGGTTACCCGCTACCCGATTTCTGCCTCGAACCCGAGACCGCGACGTTGTCGGA
GCCGATAATCGACGGCCGGAGCAGATCCATGGAAGCAACGTCCACGGACAATAATACCATGGATGGGTGGT
GTGAAATGAAGGGTGTGAAGAGAAAAAAAGAGAGAGGAGGTGGGTGCAATAGAATTGCATTTAGAACAA
AGTCGGAATTGGAGATCTTGGATGATGGCTTCAAATGGAGAAAGTATGGCAAAAAATCTGTCAAGAATAGC
CCTAATCCAAGGAATTACTACAAATGCTCAAGTGGAGGGTGTGGAGTGAAGAAGAGAGTAGAGAGAGACA
GAGAAGATTCAAGCTATGTTATAACAACATATGAAGGAATTCACAACCATGAAAGCCCTTTTTTGGTTTATTG
CAATGATCCAATATTTCATCCTCATGCCACTTGGCCTTCCTCTTTGTACCATTCTTCTCCTCCCTATTCTTCCACT
ACTACCCTTTGA

>LcWRKY17 Chr04:47142779-47144474 probable WRKY transcription factor 15

ATGGCCGTGGAGCTCCTGCCCCACGCCCATCCACTCTCCTCCACCCACAACATGGAAGCCAACGCCGTTCAA
GAAGCCGCCTCCGGCCTCGAAAGCGTCGAGAAGCTCATCCGATTGCTCTCCAATGCCCACCAACAACACCA
CTCCTTGCCTTCTATTCAATCCCCCAATTCCAATTCCAATTCCGCTCTCGATTTCCCCACCGACTGCCGCGCCG
CCGCCGATGCAGCCGTCTCCAAGTTCAAGAAGGTCATTTCTCTCCTCGGCCGGAGCCGGGTCGGCCATGCC
CGCTTCCGAAGAGCTCCTCTCCCTCTCCCCCAAACCCCCCAAGTCCACTACGCCACTCCGATCCAGCAGATCC

CGCCCCATCGCCACCACCCCCCCGGCGACCTCGATAAGAAGGAGCCTCTGACCACCAGCGTTAATTTCTCCT
ATTCCTCCGCCCATAACTCCTTCATCTCGTCGTTGACCGGCGACGGCGACAGCAAGCAGCCTTCCTCCTCCTC
CGCCTTTCAGATTACCAACCTCTCCCAGGTCTCCTCCGCCGGAAAACCCCCTCTCTCTACTTCTTCCCTCAAG
AGGAAGTGTAGCTCCGATAACTTGGGATCCGGCAAGTGCGGCGCTGCTTCTTCCTCCGGCCGATGCCACTG
TTCCAAGAAGAGAAAACTAAGAGTGAAGAGGGTGGTGAGAGTTCCGGCGATCAGCTTGAAAATGGCGGAT
ATCCCACCGGACGATTACTCATGGAGAAAATATGGTCAAAAGCCAATTAAAGGCTCTCCACATCCCAGGGGA
TATTACAAGTGTAGCAGCGTGAGAGGATGCCCAGCCCGGAAACACGTAGAACGGGCCGTAGACGATCCGG
CGATGCTAGTGGTGACCTACGAAGGAGAACACAATCACACCCTCAGTGTACCCGAAACCTCCAATCTCATCC
TCGAGTCCTCGTAG

>LcWRKY18 Chr04:51709386-51711938 WRKY transcription factor 2-like protein

ATGGACAAAGGATGGGGTCTAACCCTTCGTGATTCCGATCAGTCAATTGGGTTCTTCTCAAACAAGCCACCG
CCGCCCACTGTCAACTCCTTTCAGAGAATGTTTCAAGGTATTGAATTTTCCGGAAAACTTGGCCGGACTGAC
GACGACAACCCCGCTCCGCTGCCCTCCGATGAGAATCGGTTGGTCGTCAACGAGGTGGACTTTTTCTCCGAT
AAGAAAAGGGTGGTTGATGATACCAGGGAGGATCAAGACTCAAAATCTTCCATTAATATTATCGCCACCGCT
GTCAATAAAGACGACAAGAATTTGACCGCTCCCAGAACTGGCTTCAATCTTGTTAATACTGGTTTGCATCTGT
TAACTGCCAACACCGGAAGCGATCAATCAACGGTGTCTGATGGGATTTCATCAGATGGTGAAGATAAACGA
GCCAAGAACGAGCTAGCGCAGCTTCAAGTGGAGCTCCAGCGTATAAACGCAGAGAATCACAAGCTTAGGG
ACATGCTAAGCCATGTGAGCAACAACTACAATACCCTACAAATGCATCTCTTGACCTTAATGCAACAGCAACA
GCAACAACAAAACCAGGCCACGGAACCCGCTCATGAACGAGAGATTGGCGAGAGGAAATCGACGGAAAA
ACATGAGGTTGGAAGAGTGGTAGTACCGAGGCAATTTATGGATCTAGGACCAAGTGGGAACACAGCCGAA
ACCGATGAATTATCTCATTCATCATCAGACGAAAGAACTCGTTCTGGGTCTCCATTGAACAACGCCGAAACCG
GCTCCAAAAAGAGCACTGGAAGAGATCACGAAATCGCTCCTTCCGATCACGAGAATTCAAATTTCAGAGAT
GGCAAAAGATCATCCACCGCCAGAGAAGAAAGCCCCGAATCAGAATCTCACGCTTGGGGTCCTAATAAAGC
CCCTCGATTGAACTCCTCCAAACCACTCGATCAATCCACCGAAGCCACCATGAGAAAAGCCCGTGTCTCAGT
CCGTGCTCGATCAGAAGCTCCCATGATTTCTGATGGGTGTCAATGGCGAAAATATGGGCAAAAAATGGCCAA
AGGAAACCCATGTCCACGGGCGTATTATCGATGCACAATGGCAGTTGGTTGCCCAGTTCGCAAACAAGTTCA
ACGATGTGCTGAGGACAGGACTATATTGATAACAACTTACGAAGGCAACCACAACCACCCACTTCCGCCGGC
CGCGATGGCGATGGCGTCCACCACGACGGCGGCGGCCAGCATGTTGTTATCCGGGTCCATGTCGAGTGCGG
ACCATAATCTAATGAACCCAAATTTATTGGCTCGAGCCATACTCCCATGTTCCTCAAGCATGGCTACGATTTCA
GCTTCGGCTCCGTTTCCAACCATCACATTAGACCTCACTCACACCCCAAACCCACTGCAGTTCCAAAGACCG
GCGGCGACGCCGTTCCACGTACCCTTCGCAGGCGGGCAACCACCGTCTGCAGCCGCCGCCGCCCAACTACC
GCAGGTTTTGGGGCAGGCGTTGTACAATCAGTCAAAATTCTCGGGGCTACAGCTGTCCCATGAGATGGGAG
CCAATTCCTCTCATTTGGGTCACCCCCAAATTGCACAGCCGGCAGCCGCGTCCCAGCCCGGTGGTGCTTCTT
TTGCCGACACGTTGAGTGCCGCCACCGCCGCTATCACCGCCGATCCCAACTTCACCGCCGCGCTGGCCGCC
GCTATCTCCTCCATCATCGGCGGAGCTCATTCAAATAACAACACCACCACCACCACCAATACTACTTCTACAAC
AACAAACAACAACAACGGAAGCAACAGCAGCAAAATTAGCAGTTTCCCTGGAAATTAA

>LcWRKY19 Chr04:53830845-53832838 probable WRKY transcription factor 28

ATGTCAGATGAAATGTTTAGAGACGTTTTTTTCTCGGACAGTTTCCGAGATGGTTGGTTGGGTGATTTAGAA
TCAGGCATGGATGAATACGACGAGTCTCTGGCGAGAGCTTTTGGCGTGTCGGGTTCTTCGAATAATAATGAA
ATTTCGGGGACTCCGATGAATTCTTGTGGTTCGTTATCGTCTTCTGATGCTGGAGCTGAGGAGGATGATTCTG
GAAAGGAAAAGGATAAACAGATCAAAGAGATGGACGATGATTCTGGACAGACTTCTAAGCCTGCGGCGAA
ATCGAAGAAGAAAGGAGAGAAGAAAGAGAGAGAGCCGAGATTTGCTTTCATGACGAAGAGCGAGGTTGA
TCATCTTGAAGATGGATATAGATGGAGAAAATATGGACAGAAGGCTGTCAAGAATAGTGCCTTTCCCAGAAG
CTACTATAGATGCACAACTCAGAAATGTGGAGTGAAGAAACGGGTAGAGAGATCGTTCGAAGATCCGTCCA

TTGTGATTACGACTTACGAAGGTCAACACAACCACCCAATCCCAGCCACACTGAGAGGAAATCTGTCGGCC
GCCGCCGCGTTTCCACCGTCCATGCTGGCACCAATGCCAGTGGTCGGTGGTGGCGCCGCGTTCCTCCCAGA
ACTCTTGACCAATACTTCCGGCAACAACCAGGCCGTCGGCGGCTCCGTTTATTCACAGAGCAGCTTCGGCTA
TCCTTACAACGGGCGGCAGCCGGAATATGGGCTTCTGCAGGACATTTTTCCAGCTCCGTCGTCGTTCTACAA
CCGGCAGCCGTGA

>LcWRKY20 Chr04:54892243-54896234 probable WRKY transcription factor 14

ATGTGTAGCTTGTTTGAGATGGAGAATTACCAAGGTGATTTGACTGATATTGTGAGAGGAAGAAGCACTTTT
GGTTGCAAAACAGAAGACCCCTTTTCTTCAGAGTGGCAATTTTCATCAGAAATGGCCATGAATTTCTCTTCCT
CTTCAGAATTACAAGAACAAGCTGCAACTGCAAGAGAAGAAGAATACAGTTTTGGGGATCCATTTTGTGCT
GCAGCCATGAGAGATCCACTTCTTCAAGAGCTTGATATTTGTGGTAATAATAATAATAATTCATCTTCTTCTTCT
TCTTCTTCAGCTTTTTTCAATGGTGGTGGTAATTTAGAGGACAAAAGTGGTGGTGGAGGAAGTGTTTTTGGT
GTGAGCAGCTGCCTTCATGAAGACGAGCTTATCAAAAGGCCTTGCAATATATTCTCTCGCATGCTTCAGATCT
CACCAGCAACAATTACAAACAACAAATTCCCAATTTCAGCTTCTCCTTGTGATTCCCCTTTGCTAATTCCCAAT
TGCAATTCTCCCACACATCCATTAATTCATGCTAATAATTCCGACCTTCATCATTTTGTTGACAATAATCCCTCCG
CCCTTCAGATCTCTTCTCCTAGGAATAATCCAGCTGGTATCAAGCGAAGGAAGAGCCAAGCAAGGAAGGTG
GTTTGTGTTCCTGCTCCAGTGGCTGCCAGCAGCAGGCCTAATGGGGAAGTTATTCCTTCTGATCTCTGGGCT
TGGAGGAAATATGGTCAGAAACCAATTAAAGGTTCTCCATATCCTAGGGGCTATTATAGATGCAGCAGCTCAA
AAGGTTGTTCAGCCAGAAAGCAAGTGGAAAGAAGTAGAACAGATCCCAACATGTTGGTCATTACATACACA
TCTGAGCACAACCATCCATGGCCAACTCAAAGAAATGCTCTAGCAGGCTCTTCAAGGTCCCAACAATCAAAA
AACAACACACCCAATTCCTCGAAACTCTCCTCCACAGCTCAGCCTCAAAAGCCAACGACGACAACTCAAGA
GGGATCGGACAAAACTAAGGAAGACCAAACCAACAACAACAATGGAACAAGATCCCCAGCAGCATCAAAT
AATGTCAAGGAAGAGGCTGTTGAAGAGATCGAGAAACCGTTTGAAACAGAAGAAGGAGACCAATTCAGT
GAAGGGTTTCAATATAGAGTGGCAATGGGAAGCAACAACAATCAAAGTGATGATTTTTTTGCAGATTTGGA
AGAATTAGAGACCGACCCATTAAACCTGTTGTTCACACAAGGCCAAAAATTAGACCAAATTAGCAAAGGGG
GCGGCTTGGACGACGTCGTTGCATTCAATAATCTCTTCGATTGGGCAGCTGAAAACAACAACAGTAACAACA
ATTCCAACAATTCATTTGAGGAACAACCACCACCTGCTACTGCTAGCAAGAGAGGTTTTTATTAA

>LcWRKY21 Chr05:4760592-4764317 probable WRKY transcription factor 48

ATGGAGGAGCTGGCGGCATCCGATTCGTCGCGATGTCCGTTTCCCTACGCTTTTGAATGCGCTGAGGACGGT
AAGAGTCGTCTAGGGTTTATGGAACTTCTTAGCATCGATCAAGATTTTTCGTCACAATTTGATATGTTTTCGAC
ATCGTCTTTATCGTCTTCCCTTATTCCAAATCTCAATATAGACTCCGATAATTTGGAGATTTGGAATCGATGGCC
TGCGACGCCAAATTCGTCGTCGATCTCGTCCACGTCGAGCGAGGTTGTTAATGATGAGTTGACAGAGCTGAA
TTTAGAGGGAGGACAGAAACAACATCAGCAAGAAACTGTCAAAACTGACAAGCAATCAAAAACAAAGAAG
ACGAATCATAAGAAGAAAGAGCAAGAACCACGATTCGCCTTCATGACAAAGAGCGAGGTTGATCATTTGGA
AGATGGATATAGATGGAGAAAGTACGGCCAAAAAGCGGTGAAAAACAGCCCTTATCCTAGGAGCTATTATCG
TTGTACTAGTGTAGCATGCAATGTAAAGAAACGAGTGGAGAGATGTTTGAAAGATCCAAGCATTGTCGTTAC
AACCTACGAAGGCCAACACACTCATCCTAGCCCTGTTATGGCCCGATCAACCTTCTTTCCTCCGCCCATCTCG
GCCGCCCTCTACGGCGGCTCTCCATCGTTCACCACCGCCACTGTCTCCACCGCAACCCCGTCTCTCTTCCACT
ACCAAAATGCTCATAATTCAGATTTTATTAGCCACCCGAATGGCTTCATGGCATCGTCGTTTCATCACGAGCG
AAGACCGTGGAGTGTTGCCTCCTACGATCGAGCTACTCATTTTCTTGCTGCAGCTACCGATCATGGGCTGCTT
CAAGATGTCGTCCCTACCAATATGATGAGTTGGCATGGAGAGATGGTTTGA

>LcWRKY22 Chr05:7688892-7691373 WRKY transcription factor 21-like protein

ATGGAGGAGGTTGAAGAAGCTAATCGAGATGCTGTTGAGAGCTGCCATAGAGTTTTGAATCTGTTGACTGT
GGCGTCGCCTCAAGACCACTCGAAGCTCAGGAGTTGTTTGATGGCGGAAACTGGACAGGCTGTGTTTAAG
TTTAGGAAGGTTTTGTGTCTTCTTGATTCTTCTGGGTTGGGTCATGCAAGGGTCAGGAAGAAGAAGGTCAA

GAGCTTTCAGTTTAATTCCTCTTCCCCTTTTCCTCTTCCCCAGTCCATGTTTTTAGAAACTTTTTCCCCTAATTG
CAGAATGGACCTTCTCCAGGGGAGGAATTTGCAGATGGGTCCTCTTTCTTTGGGGAGCCCATCTTTGGAATT
GAGCTCAAATGGCAAAAGTTGTTCAATCCAACAACAACAACAACAACAACCTCAATCTGTTGGTCATTATCAT
CATCACTTCCTTCAGAACAGGGTGTTGTTGAATAGTAATCCTAATATTAATCCTAATGCTTCACAGCCTGAAAT
GGTGTATCTTAGGAGCAATAGTGGCATTAACTTGAATTTTGATAGCTCTAGCTGCACACAACACACAATGTCA
TCCACTAGGTCTTTCATTTCTTCATTGAGCATTGATGGGAGTGTGGCTAACTTGGATGGGAGTGCCTTTCATT
TGATCGGGGCGCCCCGGTCTTCCGATCAGAATTCGCACCATAAGAGGAAGTGCAGTGGGAGAGGGGAAGA
TGGGAGCGTTAAATGTGGAAGTACTGGGAGATGTCACTGCTCTAAGAAGAGGAAACATAGAGTGAAGCGG
TCGATCAAGGTGCCTGCAATTAGTAACAAACTTGCAGATATCCCTCCAGATGATTATTCATGGAGGAAATATG
GCCAGAAGCCAATCAAGGGTTCTCCTCACCCAAGGGGATACTATAAGTGCAGCAGCATGAGAGGTTGTCCA
GCGAGGAAGCATGTCGAGAGGTGTCTCGAGGAACCTTCAATGCTCATTGTTACATATGAAGGTGAACACAA
CCATCCCAGAATACCTTCTCAATCTGCAAATACTTGA

>LcWRKY23 Chr05:13724246-13726258 PREDICTED: probable WRKY transcription factor 53

ATGGAGTTTGTGGGTATAATCCAAGAGCTCAACCAGGGCAAACAGCTCGCCCAGCAGCTACGCAACCATCT
CCGTCCTTCTCCTTCTCCTTCCTCTTCCTCCGATGGCATTCTCTTGATCGATAAGATCCTTCGTTCCTACGAAAA
CGCGCTTTCCGTTCTCGCCGGCGGCTGCGGCGGCGACGAAGAACTCGCGGCTGTGAAATCCGCCGTTGCTC
CGATGAATGCGGCTGTGAAAGAGGGAGACGTGTCCAAGAAGAGAAAGGTAATGGCGAAATGGAGTGAGC
GGGTTAAGGTTCCTTCTGGCTCCGCCATTGAAGGTCCTCTTGATGATGGCTTTAGCTGGAGAAAGTACGGCC
AGAAGGACATTCTTGGATCCAAATTCCCCAGAGGTTATTTTAGATGCTCTCATCGGTTTTCACAAGGATGTTT
AGCAACAAAGCAAGTTCAAAAATCAGACAATGATCCAACAATGTATGAAATAACCTACAAAGGAAAACACA
CTTGCAACAGAGTCCTTCATTCAAACACACCAAAAGAAGAACAGAAGCCATTATTGCAACACCCAAATCAAC
CAAAACAAGAACAAAAGCCATTGCGGCAACAACACGAACACGACAACCCTTCATGCTTCACCTTCAGCTCC
GACGCCATTCGGGTAAAATCCGAGAAGTTAGACGACGTCGACGATGGCCTCTCTCGACCTTTTCGCACCTTG
TCGTCGTCGTGGTTTGGATCTGAAGTTCGAGATGATCAAAGCCCCTTTAGGGAAAGTGAGTTGTCTCCGACA
TTTGAATCGAGCGATATGTTTGGATTGTGTCGTGATGGGGTCCGAGATTGGGATTTCGTAACCGAGTTCGTT
TCGGTTCCGAATTCAGTCACTAATATTTCCATTGGTGACTTGGAGGAGTATTATAGTTTTGATAACTTGGAGTT
GTTCTGTTGA

>LcWRKY24 Chr06:2919058-2922682 probable WRKY transcription factor 72 isoform X2

ATGGAGGCTGCTGCTGCTTTTGGTCGTCCAAGGCCTGTCGTCAAGACCGAGAAGCCCACCGGAGATGTCGA
CGGGGACGGGGATTCTCCTTCGAAACAACGTCTTCTCGTTAAGATGGGGAATAATTATGGAAAACAAGAAG
ATAAAACAAGCAATTCATCTGATCAAAAGGATTTGAGCTGCACCAAACCTCAGGAAGATCAACTAGAATCCG
CCCGAGCGGAAATGGGGGAAGTGAGAGAAGAAAACCAGAGATTAAAAATGAGTTTAAGCCAAATCATGAA
GGATTACGAAGCTCTAAGAACGCAATTCCTTGGAATCGTCAGACGAGAAGGGAAGAAATTATCGGAAGATG
ATGACAAGTTGAACAACGAACAACACCACGACGACGATGATCGAAACCAAACTGACGAAACAGAGCTCGT
TTCACTAACGTTGGGGAGGTTTCCGACGACGGAGAAGAATAAGAATAAGGCGGCAGACGAGAAATTAGAT
CAGAACAATAAGAGTTCTCAAGGCCGAGAGATGATTGGCGAGGAACAGGGCGTTAAAGAGACTTTATCGC
TCGGTTTGAACTGCAAATTCGAACAAGAATCGACTGTCGTTAAAGAAGTTGATTCTCCGAATCGGAGTCTGA
CAAATAGTTTCGATCATGAGGCGAAGGAGGAAGCTGGAGAGACGAGTTGGCCGCCGAGTAAAGGGGTAA
AGACGATGAGAAGCGGTGATGATGATGTTGCGCCGCAGAATCCGCCCAAGCGCGCTAGGGTTTGTGTTAGA
GCGCGATGCGAGACAGCTACGATGAACGACGGATGCCAATGGAGAAAATACGGGCAAAAGATAGCGAAAG
GAAACCCATGTCCACGAGCATACTATCGCTGCACAGGCTCACCAACTTGTCCAGTTCGCAAACAAGTCCAAC
GATGCGCCGACGACATGTCCATCTTAATCACCACCTACGAAGGCACCCACAACCACCAACTACCCGTCTCCG
CCACCGCCATGGCCTCCACCACCTCCGCCGCCGCATCCATGCTCCTCTCCGGCTCCTCCTCCTCCTCCTCCACC
TCCGCCACCGCAAATCTCCACGGCCTAAACTTCTACCTCAACAACCCAACAAACAACAACAATCCCAAACCC

AATTTTTACTTACCCAACAACTCCTCCATCATCTCTTCAACTTCTCCAACTCATCCCACCATCACTTTAGACCTC
ACTTCAAATCCTTCCTCCTCTTCCACTTCCATCTCCGCCCATCTTGGAAAGTTCCCGACCGGATTCTCCAATCC
TCGGTACCCTTTCACGAGCCAGCTCGACTTCGGATCTTCTAGAAACAATGTGCTGTCGTGGAATAACGGCCT
TCTTAGTTACAACAGAAATAACAACCCCACTGCCACGTCGACCACCAATGCAATCTACCAAAACTACATCCAG
CAACAGCAGCAACGGAACAATAATCTTAATCATCACGGTGTCGTCGCTGCTGCTGCCACGTCACTGCCACAT
CAGCAGCAGCCTTTGCCCGACACCATCGCCGCCGCCACCAAGGCCATCACGGCCGACCCGAGCTTCCAGTC
TGCTCTCGCTGCTGCTCTCACGTCGATCATTGGCACCGGCTCCGGCGCCGGTGTGATTAAGTCGTCGGCGAG
AGGTGAACAGCCGTTGTTTCAATTGACGTCGACGGCGGCGAATAAAGGGAATGGATGTGGGACGAGCTTT
TTGGGTAATATTGCGGCGGCGACGGCGACGACAACGAAGAGTAATTCGCCGCCGGCCGGGAATGTGGTGT
TTGTGCCGGCGAGTTCGCTGCCGTTCTCGAATCCGAAGAGTGCGTCGGCGTCTCCGGGTGATCATATTGATC
TTACCAATTGA

>LcWRKY25 Chr06:7989644-7991320 probable WRKY transcription factor 65

ATGGATGCTTCCTTCAACAATAACCACCAATTCTATTCCGGCGACCCCTTAGAAGACAGTGACGAAAAAGCC
CCTGATTCTCCTCCTCCTAATTCCACCGCCAAGAAGGGCAGAAGAGGGATGAAGAAGAGAGTGGTTTCGGT
GAGAATTAATGGTGACAGCCCGAGGAATTCTTCTGGCTCTACAACTCCTCCTTCTGATTCTTGGGCTTGGAG
AAAGTACGGCCAAAAGCCCATCAAAGGCTCCCCGTATCCGAGGGCTTATTATAGGTGTAGTAGCTCGAAGG
GATGTCCGGCGAGGAAACAAGTAGAGAGGAATCGGCTTGATCCGACGATGTTGGTAATTACATATTCGTGTG
AGCATAATCATTCCGGTCCGGTTTCGAGAAACAACAACAATAATAATAATAATATTCATCATCATCAAGCCGCC
GTGACGAAACCCGGTTCGCCAGAGACCGTGCCGGTTCAGGCGGAGGCGGAACCGGAAGTGGAAGAGAA
ATTTGTGGATATCGGGGAGGAGTCGTTGATAACGGGGGACGAGTTCAGTTGGTTCGGGGAAATGGAAACG
ACGTCGTCGACGGTTCTCGAAAGCTCGATTTTCTCGGGGAGGTCGAGTGGGGTGGGAGATTCGATTTCGA
GTGACGTGGCAATGCTTTTTCCGATGGGGGATGATGACGTGGACGAGTCGCTGTTTGCGGATCTTGGGGAG
TTGCCGGAGTGCTCGGTGGTGTTCCGGCGCGGCGGAGGCCGGGGATTGGCGGTGGAGGAGCAACCGGCT
GCAGGCCAGCGGCGGATCACTCCCTGGTGCGGAACCACCACCTGA

>LcWRKY26 Chr06:11318747-11322452 PREDICTED: probable WRKY transcription factor 14

ATGTTCTGCAGTTTGTTTGAGATGGACAACAATAGTTATCAGCAAGCCGGTGATTTGACTGATGTTATTCGAG
CCGGTTCCGCCGCCGCTGGCCTCTCCTCGGAATTCTCTCTCGACCCTTCTTCCGGCGACCGGCGGCTCTGGC
CCCACCTTCCCGCCGATGACCCTTCCATGAGTTTTGGAGATCCGTTGTTGTTTTCGACTGGTGCTGCTCGAGA
TCCGTTTCTTCATCCTCATTTCTCTGCCTTCAATTTTGGTTCTGACGGCGGCGGCGGTGTTGGGGGCTTGGC
GGCGGCCGCGGAACGGCCGTGTAATATATTTTCGAGGATGTTGCAGATCTCTCCGAGTTCGGATGGTGTAAT
TAGTTCTCCTCCCTGTGAGTCGCCGGTCGTCGGGTGTTCGCCGAGCTCGACTTCGACTAAAGCTTTGGCTTG
TGGCGGCGGTGGCGGTGGCGGTAGCATCGTGATTTCGAGTGGTGGCTCAAACCCACTTTGCTCAATGGATA
ATTCCGGATTGCAGATCTCTCCTCCGCGAAATCCAACCATTAAAAGAAGGAAGAGTCAAGTGAAGAAGGTG
GTGTGCATTCCGGCGCCGGCGCCGGCGAACAGCCGATCAAGCAGCGGCGAAGTGGTTCCTTCCGATCTATG
GGCATGGAGGAAGTACGGACAAAAACCCATCAAAGGATCTCCATATCCAAGGGGATATTACAGGTGCAGCA
GCTCCAAGGGCTGCTCGGCCCGAAAGCAGGTCGAGCGCAGTCGCACCGACCCCAACATGCTCGTCATCACC
TACACCTCGGAGCACAACCACCCATGGCCCACTCAACGCAACGCCCTCGCAGGCTCGACCCGCTCCCACCCT
TCCCGAACCACCGTCACCGCCCATAAAACAAGTCCGAAACACGAGCACACCAACGAGAACCCGACAACCGC
AGCTGTAAAGGAGGAAGAGATGGATGAAGACCAGACGACAGAGAAAGCAACAGCAACAACAACAACAAA
CGATCATAAGAAGGAAGGAGATCCTCAAGAGTTCTCCTACGACTTAATTTTCGCCGAATTTGAAGATCAGAT
AAACGACAATCATTATCATCATCATCATCATTCGGCGGACCCTCTGAACAACAATCTGGTGCTGTTCAGTAGCC
ATGGTTTTAGCACAGGAAATGATCAAGAGCGCAGTAAAGATCCATTTTTGGAGCTCTATGATTGGGCAGAG
AATTCAAATGAGAGCTTGTTCAAAGAAGCCAAGGGAGGTTGA

>LcWRKY27 Chr06:46758360-46762744 WRKY transcription factor 44

ATGGACAACAAAGCAGCAGAAAGAGTTGTTATTGCCAAACCGGTCGCTTCAAGGCCAACATGTTCCAGTTT
TAAGTCATTCGCAGATATCCTTGCATGTGCCTTCAATACTTCTCCACCTAAGACATCGTCTGAGACCATGGTTT
CTGCCATTAGGCCGAAAACAGCGAGGTTTAAGCTGAAGGATAATCCTGCTCCATCACCCAGGGCTAAAATAT
CAGAAACACTGCCTGGAACGACCTCTCGTAGTTCATCTGAAAATCTAACCTTATCAGACAGCAAATCCACTGT
TTTATTCAAACCCTTGGCAAAACATGTATCGAAAAGGACTGTCTCTCAGCTGTCACTCATGGGGAACACTAAT
TTGCAAAATCATTTACCACCACCACCAGTCGAGGCCCGTATCGAATGTCTGAACCAAGGTAAAGACAATATCA
AATCTGCACTGAACTCTAATCTCCCCCGGAACATCACATCTGCTGTTGAAAACAGTCAATCCATTGAAAGCTC
AAGAGTGACATTGAATTATAGTAAAGAAGATCCAACTTCGCTTTGTCCTCGAATTAGTTGTGCTCAGCCTTCA
TATGACGGGTATAACTGGCGGAAATATGGGCAAAAGCAAGTTAAGGGAAGCGAGTATCCACGGAGTTATTA
CAAGTGCACACATCCAAGTTGTCCTGTCAAAAAGAAGGTTGAGAGATCATTGGATGGGAAAATTGCTGAAA
TTGTTTACAAAGGTGAGCATAACCACCCAAAGCCTCAGCCTCTAAAGCATAACTCATTGGCAACACAAGGGG
AAGGGTCGATATCAAATGGAACTGCACGAGATACTAATTCCGAATTATGGCTTCATTACCTCAATGGACAAAT
CGAAGGTTGTGAAAGTAGGCTCGAGAATCAGATCGACAAAACTTGTCAAGGAAGAGTCACACAATCCTTTG
TTCCGGTTGCAATTAGAGAAGTCAATACTGGATGTGGAATCTCGGATAATTCATGCGGTCTAAGTGTAGAATA
TGAAGAAGGAAGCAAAGTACTTGTGCCCATGGGTGATAAATTGCGAAGTAAAAGAAGGGATGGCAAAAAT
CCAACAAATGAAGCTGATACATCAAGTGAAGGTGTGAAAGAGCAGCATGCCATGGCTCGAGGTTGTACTGA
TATCGAGATTTCTGGCAAAGGCATTCGCTGGAGGAAATATGGGCAGAAAGTTGTGAAGGGAAATCTATATCC
TCGAAGTTACTACAGATGTACTGGCCTCAAATGCAAAGCACGCAAGTACGTCGAACGAGCATCCGATGATCC
AGATTCCTTTATAACTACTTATGAAGGAAAACACAACCATGGTATTTCACTAGGAAATGCAAATCCTCTAACTC
CTGAAATGGAATAA

>LcWRKY28 Chr06:48356425-48359744 probable WRKY transcription factor 26

ATGAATAACATAAACCAAGCGATAAATGCACTCGCTGGCGGATCTTCTGACAACAGAACCAATAATTTCGCCA
TGGAAGCTCCGAAATTCAAGTCTCTTCAACCTCCTTCTTTCCCCTTGTCTCCTTCTTCTTACCTTTCCGCTTTCT
CTTCTGGTTTAAGCCCCACTGAGCTTCTCAATTCCCCTCTCCTCTTCTCCTTTGGTGTTTTTCCCTCTCCCACTA
CTGGTGCCTTGAACTCGAGAAATGATTTCGATGGTGGTGAGCAGCAAGAAGAAATGAAGGGCGATTTTAAA
AATTACTCTGATTCTTTGTTTAACCCTCAAACAGGATCCTCTGTTTCGTCTTATTTTCAGTCGTCTTCTTCCCCA
CTGAATCCAGGCGGTTTGTCGTGTGATGAAAGTGGAGCGAAATCTGAATTTGTTGCCTCGGAAATGGCGGC
AGCTCAAACGAAGCAGATTTCTCAGCTGCCTCTTTACAACAGAGAACAGCATAAATCGGAGAACGATGGAT
ACAACTGGAGAAAATATGGGCAAAAACAAGTGAAAGGAAGCGAAAATCCTCGTAGTTATTACAAGTGCACT
TTTCCGAGCTGCCCAACGAAGAAGAAAGTTGAAAGATCTTTAGATGGGCAGATTACTGAGATTGTTTATAAG
GGAAGCCATAATCACGGCAAGCCACAGCCGACGAGACGGTCCGGTGGCTCCGCCGTTTATGATCCGGCCGC
GGCGGAATCCGCCGTGCTGCAGGAGGATTCCTCCGTCTCGGTCGGGGATGAAGAATTGGAACCCAATTCGC
CGTTTAGCAACTCGGTGGACGACAATGAAAATGAACCGGTAGCTAAAAGATGGAAAGGGGAAAATGAAAA
TGAGGGATTTTCTGGAGGGGGAAGCAGAACAGTGAAAGAGCCAAGAATTGTTGTTCAAACAACGAGTGA
AATTGACATATTGCCTGACGGCTATAGATGGAGGAAATACGGACAAAAAGTTGTCAAAGGAAATCCAAATCC
GAGGTATACCTCTCACTCTTCAATGTTTCATCTTTTCACAGTTTTTGCAAAATGCAACTTTTTTACCTTTTCTCT
TCTTTTTAAGGTTAAATTCTATTTTACATTTAACTCCAATTCCCTCTCTACCCTTTTACAATTTTTAATAATTGTCT
TCCTAATTTCAATTCCTTTAAACTCTTCCATCTTATTCTTACCTTTCGGTTAA

>LcWRKY29 Chr06:48619644-48620522 PREDICTED: probable WRKY transcription factor 70

ATGGATTGCTCCTGGCCTGAAACCACGCCCTTCGACCGAAGAAAAGCGGCCGACGAACTGCTTCGTGGCCG
TGAATTCGCCCAACAACTCGGAGCGCTTCTCCGGAGGAATTCGAGTTCCGGTGGAGCAGCCTCCCAAGAA
GATCTGCTCTCAAGAATCCTAACCTCTTTCTCCAAAACACTTTCCATCTTGAATCGCTGCGACTCCGACGACAT
AAATGGCTCTATTGTGGATTCTCCCGACGATGCTAGAAAATCCGAGGAATCTGGAGACAGTTGCAAGAATAA
TGATCGCAGGGGTTGCTACAAGAGAAGAAAGAGCTCGCATAGCTGGGCGAGAGAGAGCTGCAGCCTGGT

GGACGACGGGCACGCGTGGAGGAAGTACGGGCAGAAGGTGATTCTAAATGCCAAGTACCCTCGAAACTAC
TACCGGTGCACGCACAAATTCGACCAGGGGTGCCAAGCCACAAAGCAAGTCCAGCGAGTGGAAGAGGATC
CCCCCAAGTTCCGTACTACTTATTACGGACATCACACCTGCACCAATTTCCTCAAAGCTTCGGAGATCGTACT
GGGTTCTTCCAATTTCAACGATTCCTGCGGAGTGCTTCTCAGTTTTGACACTCCGACCGTCGCACAGGACAA
CTTCTTTTTGCCCGCAGATGATGACGTCAAGAAGGAACTTGCAGTCTGTTCCCCCTCCGATTACATGAGCGC
CGGCCCCTCGCCCGACCATCTCTCCGAGGTCATCATGGGTTCCGTCGACTTTGAGGACGATGTTTTACAGTT
CGACTTTTGA

>LcWRKY30 Chr07:9134090-9136383 PREDICTED: probable WRKY transcription factor 13

ATGGGTTCTAAATCCCAAGTCCTATTGAACCCACAAGCTTTGTTTGAAGATCAAGCGCCCTCATCAGAAGCA
GCAGCTCATTCTCAAATGGGATTCTTCTCTTTCCCTTCTTCAAACTTAACCTTTTTTCAACTTCCTTCAATGCCC
CAAACCCATTCTTCTTCTTCTTCCCCTCCATTTGAGCCTCCCAATTTCTCCACTACTTCCAATATCAATATTAATA
ATAATACCCTCTCTGAAACCCTACTTTCTTCTTCTCTTTTGCCCCTCAAGCCTTCACTTCTCTCCCCTTCCTCTCA
CTTTGCACCTCAACACCTCCTCTCCTTGCAAACTTCCACTCCAAATCTATGGCCATGGGGAGAGGTTGGAGA
GAGATTGATGAGTGGGAAGAGAAATGAGTATCAATTGGGAGTGTCAACAATGAAGATGAAGAAGATGAAG
GGAAGAAGAAAGGTGAGAGAGCCAAGGTTCTCCTTCAAGACCATGAGCGATGTGGATGTTCTTGATGATG
GTTACAAATGGCGAAAGTACGGACAGAAAGTGGTCAAAAACACACAGCATCCTAGAAGCTACTATCGTTGT
ACACAAGATCACTGTAGGGTAAAGAAACGAGTAGAGAGATTAGCTGAGGATCCAAGAATGGTGATTACAAC
TTATGAAGGAAGACACATTCATTCTCCTTCTCATGATTCTGAAGACTCTGAAGCTCAAACCCATCTCAACAATT
TCTTTTGGTACCCTCTCTCTCTCTCTCTCTAA

>LcWRKY31 Chr07:10143214-10149894 probable WRKY transcription factor 51

ATGGCCAGCTTCCCTAACGATGATCCTAACCCTAACCCTGACCCTAACCCTAACCTTAATTACACACATTTTCC
CCCAATTCTCGATCCCAATTCCTTCTTCGATTTCGAGCTCTCCGATTTTCTTGTGTTCGGTGACGATGAGAACG
TCGATCAAACTGCATCGACCTCACCGAGCATCGCCTCGTCGGAGAAGATTACTGGGGTGGATAGTGGCAAC
AGTTGCTCTGCAGTCGACAGCGGATCTAGCACCGTAGTTTCATCGGGAACGTCGACGTCCTCCATAAGAAGC
AAAAATGAAGAGAAGAAAAGGAAGAGGGAGATGGTGTTTAGGGTTGCATTCAGAACCAAATCAGAGCAG
GAAATTATGGATGATGGCTACAAGTGGAGGAAATATGGGAAAAAGTCTGTCAAGAACAGCCCAAATCCCAG
AAATTACTATAAATGTTCAAGTGAAGGATGCTGTGTGAAGAAGAAGGTGGAAAGAGACAGAGAGGATGCA
AGCTATGTGATTACAACTTATGAGGGAGTTCACAACCATGAGAGCCCCTTTGTTGTGTATTACAATCAAATGC
CATCCTTTGCCTCTACTCCTACCTAA

>LcWRKY32 Chr07:27913508-27914770 probable WRKY transcription factor 13-like

ATGGGATCATCATCAGAGATAACAGTGAACCAGTTGGTGTTGAGGAGTCCATTGCAACCCCAACCTGCCGCC
GACGCCTCATCCTCCTCTCCGATAATTCAACAATCTCTTCCTAGGAATGTTGAGGAGAGCGACAGCAACGATA
ACGGCGACCACAATGTGTGTAAGCGGCAGTTGAAGGTGTCGACGAAGGAGATGAAGGTGAGGAAGAGAG
AAGGGAGGAAGAAAGTGGCAGAGCCAAAAATCTACGTCATAATCAAGAGCGATGTGGATGTTCTTGAAGAT
GGTTACAAATGGAGAAAGTATGGCCAAAAAGTTGCTAAGAACTCCCTCCATCCCAGGAATTACTATCGTTGC
ACACAAGAGAATTGTAAGGTAAAAAAGCGAGTAACAAGATTGGATAAAGATCCAAGAATGGTGTTGACACT
CTATCAAGGAAGACATATTCATTCAATTCCATTACAAGAGACAATGGGTCAGCCACCGTCGGACCATCTTAAC
AATTTCTTCTGCTAA

>LcWRKY33 Chr07:32119638-32121520 probable WRKY transcription factor 15 isoform X2

ATGGCGGTGGAGCTCCTCACAGCCTTCACCAACGCCCAGCTCTCCGCCCCAATGGACCAAGACTCCGCCGT
CCAAGAAGCCGCCTCCGGCCTCGACACCCTCAAAAAGCTCGTCTCATTACTCTCCCACTCTCCCCCTTCCAAT
CTCGACTCCGATTGCCAAGCCGTCGCCAACGCCGCCGTCTCCCACTTCAAGAAGGCCATTTCTCTCCTCGGC
CGCTCCCCCCGAACTGGCCACGCCCGATTCCGCCGTGCCCCTTTGGATTCCTCTAAAGTTTACAACGCCACTC
CCATTCAACAAATTCCTCCTCCCCTTGAGCGCCGCGAATCGGCCACCACCATCAATTTCTCTTACTCCTCTGCT

CCTAGCGGCTCCTTCCTCACCTCGCTTGCCGGTGCCGACTCTGAAATTAAGGTCCAACACCAACCCTCCTCTT
CTTTTCAGATCACCGATCTTTCCAGAGTTTCTTCTGTTGTCTCTAAGCCTTCTTCTGGTCTCAAGAGGAAGTG
TGGCTCTGAGAATTTGGGCTCTGGGAAGTGCAGTGGATCCTCTGGCGGTCGATGCCACTGCTCTAAGAAGA
GAAAAATGAGGTTGAAAAGGGTGGTGAGAGTTCCAGCCATAAGCTCGAAGAACGCGGATATTCCTCCTGAT
GATTATTCATGGAGGAAGTATGGACAGAAGCCAATAAAGGGTTCTCCATATCCAAGAGGTTACTATAAGTGC
AGCAGCTTAAGAGGATGTCCAGCTAGAAAGCACGTCGAGAGAGCCTCGGATGATCCATCTATGTTAATAGTG
ACTTATGAAGGCGACCATAATCACTCTCAATCAGTTGCAGAAGCCTCAAGTCTCATCCTTGAATCTTGGTGA

>LcWRKY34 Chr07:42704310-42706648 PREDICTED: probable WRKY transcription factor 12

ATGTCAAATGAAGATCATCAGAAGGATTCTTGTTACCAAAAATTTGATCCTTTCACTCACAATTTCCATCATCA
CAAACCCTTTGAAGCTCCTCTTTTAAGTCCCTATGATGCTTTTGATCCCTCTTATGTGGGGTTCTTCTCTGATTT
CTTACATGCCTCTGCTGATTATGACTACAACAACATCCTCTCCGCTGGCCTCGAGATGTCGTGCTCGTCGTCCG
AAGTTATTTCTCCAATCGACGAAGCTTCGAAGAAGTCTATGGGCGTCGGAGAGTCCGTGGTGACCGGTGAG
CACCCGTCGACGCCGATTTCCTCAACGGCATCTTCGTCCGATGAGGCTGCGCCGGGTGGTGGTGATTCTGCC
AAGAGTGGGGAAGAGGTGAAAGAGTTTGAGGATAAAAGTGGAGAAAATTGCAAGAAAGGGAATGGACG
AAAAATAAAGAAAGAGAAACGTGAGAAAGGGCCACGATTTGCGTTCTTGACCAAGACTGAAATTGATCATC
TTGAAGATGGATATAGATGGAGAAAATATGGTCAGAAAGCAGTCAAAAACAGCCCTTTTCCAAGAAGTTATT
ACAAATGCACAACTCAAAATTGCAGTGTGAAAAAACGAATAGAAAGATCAACCGAAGATCCATGTTTTGTTG
TGACAACGTACGAAGGGAAACACAACCACCACTGTCCAATTACACTTCGAGGCCAGAATGCCGCCGGAGTG
TTGCCGGCGTCGGCTTCACCGCCGTTGCTTCCCGGATTAGCTCCGGCGAACTTCTTCTCCGGCGAGGTGTAT
GAAGGGGTTGATCTGCTGCAACAATATTATCAGGTTCCTGATTATGGGCTGTTGCAAGATTTGATTAATCCATT
ATCATTCAATCGAAAGCAAGAGCCATGA

>LcWRKY35 Chr07:43494758-43498837 WRKY transcription factor WRKY24-like

ATGGAGTTTTTCTTCTCTGCTTCTGTTGAAGATCCCACCTACGGCCGCCGGAGACTGCCGGAGCGTACGGCG
CCGCCCATGTCTAAGCTCATTTCCACGGAAAATCCGCCATTGTTGCCGGCCCTGTCTCCGCCTCTTTTTTCTCC
TTCTTCGTTCTTCACTATCCCTCCTGGGATCAGCCCCACCCAGCTTCTTGATTCCCCTGTTTTTCTTAACCCTTC
TAATATTCCGCCATCGGCGACTACCGGAGCAATTTCCGCCGGAGACTTGACCGGAAACAGAAACAATTCCG
GCCACCACCAACAGAACATCAAACAAGAACACAACAACTTACCTGACTTCTCATTTTCTCGTACCCATCCCAC
AAAATCATCCTCAATGTTTCAATCATTTTCAACACAGCAAAACCGGCCATGGAGTTCTCAACCCGAACCCCCC
GTCAGTTTCCCGCCGGAAACCACCATCTCCGGCGGCTCCAAACGGGATCACCGCCCGCAATCGCAATCGAG
AAACAGAGCATCCGACGACGGGTTTAACTGGAGAAAATACGGCCAAAAACTGGTAAAAGGAAGCGAAAAT
CCCAGAAGCTATTACAAATGTACACATCCAAATTGCCCTGTGAGAAAGCAAGTCGAAAGATCATTGAATGGT
CAAATTACAGAAATCGTTTACAAAAGCAAACACAACCATCCAAAACCAGAGTTCACAAGAAGATCGTCATCT
TCTTCTTGTTCTTCTTCATCTTCATGGTTATCGGAAGCTTTGCCTCAAACGATGGCGGCGGAGCCAAAGCATC
CGGCGGCCTCTGATTCCATGCCGACGCCGGAGAACTCTTCGATTACGATCGGCGACGATGAATCTGATGAAG
CCGATGCAAAAAGATGGAAAAGTGAGAGTGAAAATGAAATGGTGTCGGCGGCGGGAGGGAAGACGGTG
AGAGAACAGAGAGTTGTGGTTCAAACGATTAGTAATGTGGATAAATTGGACGATGGATATTGGTGGAGAAA
ATATGGACAGAAAGTGGTTAGGGGAAATCCAAATCCAAGAAGCTACTACAAATGCACGTACGCAGGCTGTG
GAGTGAGGAAGCACATAGAGAGAGCATCCCACGATTTAAGGGCAGTCATCACTACCTACGAAGGCAAGCA
CAACCACGAAATTCCGGCAGCCCGTGGCGGCGGTGGCGGCAGACCGGTACTCAATAATCACGGCAGCAGC
AACGACGGTGCATCGAACAACGGCAATGGCGACCGACGACCGAGTTCAGAGATCTCTGGTGGCGATAATG
GATTATCAAACATGTTGTGGAGGACCAAAGAAGAGGCAGCCGATGACATTTTCCAGTCGTTATTAGACTGA

>LcWRKY36 Chr08:4389112-4392520 WRKY transcription factor 1 isoform X2

ATGGTTTCTTCAGGGGACCAAGTAGATAATGAAGTTGATTCTGATAGATTGGAGCATGAACAGAGTTCAGAT
AGCCAATCGCAAGCGTCTCAGGAGGATCCTGGAGGAACTAATGCATCAAAATCAGATATCAAATGCACGGC

AGCTGCCTCGAAAACACTTGAAGAAGCTGTCAACCTGCCTGACGTTACAATCGAACAAGTGGATCGCGGAG
GTATCTCTAATATAGTGTCTGAGAAAGTGACCCATAAGCCAACTACTGCGGACCAGGACTCACATCCTGATTT
GAAAGTAAGTATCACCTCTACAATACGAGAGAAAGTGTCAGAGGATGGATATAACTGGCGGAAATATGGTCA
GAAACTAGTAAAGGGAAATGTCTTTGTTAGAAGCTATTACAGATGTACGCATCCAACGTGCATGGTGAAAAA
ACAACTAGAGCGCACTCATGATGGGAAAATTACAGATACTGTTTACTTTGGCCAGCATGATCACCCTAAACCT
CAACCTCACATTCCAGTTGCTGTTGGAGTTGCCATGGTTGAAGAAAAATTAGATGAACATGCTTCTAGAAGT
TCTCAAGATAAGACCTCCATTGCGCTTGGCCAGACACCTCATCAAACTGGGCCGGTTGATACACTTCAACTG
TCATCAGTCACGGCCAGCGATAATGTAAAAGATGAAGCTTTGAAAAGGTCTAGGATTAATGATGAGGATGAC
AGTGATGATACACCAGACTTGAAACGAGAGAAGAAAAGATGTAATATTGACGTGACAGTAGCAGACAAGTC
AACAGTTGAATCTCGTGTTGTTGTTCAAACTCCTAGTGAGGTTGACATTCTCAATGATGGATACCGCTGGCG
GAAGTACGGACAGAAATTAGTGAAAGGCAATCCAAATCCTAGGAGTTACTACAGATGCTCAAGTCCTGGAT
GTCCAGTTAAGAAACATGTAGAGAGGGCGTCTCATGATCCAAAAGTGGTGCTTACCACGTATGAGGGTCAG
CACGACCATGATATGCCTCCTGCAAGGACAGTGACGTTGAACTCAGTTGGGGCCGCAGCAGCCCATAGTGA
TGAGATAAAAACAAAATCCGTCGGTAGTTCGATTAGCCATGATACGGTGGTTCATGCTAGTAAGGATCCTCGA
AGCGATTCAAGTTCTGAAGGCAAATTAATTGAAAAGAATGGCACGTCGAATGCCACAGGAGCAAGTGATGG
CATTGTACTCGATATGGTGGTAAATTCCAGTCCAGGAGTTGCGAGTGGACAAAATAAGCAGCTCAAAGTAGC
AATTGAAAGCTAA

>LcWRKY37 Chr08:6315882-6318558 WRKY transcription factor 3-like protein

ATGGACGCCGCCCCTTCCCCTCCGCCGCCCACCCGCCACTTCCCGGTCAACCTCAATTCCACCCTCCACGACT
CTGATCCTCCTCCTCCTCCCCCTCCTCCGCCTCCTCCCCCCTCCTCCTACGAGATGAACTTCTTCGCCGCCGAC
GATAAATCCCGCGTTCTTCTCGCCAAACTCCCTGCCTCCGACAACCTCGACTTCAACGTTAACACGGGGTTG
AATCTCTTGACGACGAATTCGAGTAGCGATCAATCGATGGTGGACGATGGAGTTTCTCCCAACCAAGAAGAT
AAAAGAGCCAAGAACGAGCGAGCAGTTCTTCAAGCTGAATTGGAGAGAATCAACGCGGAGAATCAACGG
TTGAAGGAAATGTTAAATCAAGTGACGAGCAATTACCAAGCGCTGCAGATGCACTTCACGACGCTGATTCA
GAACCAGAAGGCGGGCGACGCCGGCGACCCAATGGACGAAAAGGCCGGCGGCGGCGGCGGCGGAGGT
CAAGAAAAGGTCCGGCACGGCGGCGGCGGAGGGAATAACAACAGTAATAAGCTGGTGCCGAGGCAGTTT
ATGGATCTTGGATTGGCGACGAATACGGACACGGATGAGCCGTCGATGTCGTCGTCGGAAGGGAGGAGCG
GCGAGCGGTCGCGGTCGCCGGGAACCACCGGAGAAGTGGCGTCGTCGAAACGGCATAGCCCAGATCAGG
GCTCGAATTGGGGTTCGAGTAAAGTCCCTAAATTCAGTTCTTCGTCTTCTTCTTCGGGTAAAGATGTGGATCA
AACTGAAGCTACTATGAGAAAGGCCAGAGTCTCCGTTCGAGCCAGATCCGAAGCACCCATGATCACAGATG
GATGCCAATGGAGAAAATACGGACAAAAAATGGCGAAGGGAAACCCTTGTCCTCGAGCTTACTACCGCTGC
ACCATGGCCGCCGGCTGCCCAGTTCGAAAACAAGTACAAAGATGTGCAGAAGACAAAACAATTCTGATAAC
AACCTACGAAGGCAACCACAACCACCCATTGCCGCCGGCCGCCATGGCAATGGCATCAACAACATCATCAGC
GGCAAGAATGCTTCTATCAGGATCCATGTCAAGTGCCGATGGTTTAATGAACTCCAACTTTCTAGCAAGAAC
CCTACTGCCTTGCTCTTCAAGCATGGCCACAATCTCAGCCTCCGCCCCATTTCCAACCGTCACATTAGACCTAA
CACAAACCCCCAATCCCTTGTTCCAACGGCCCGCCGCCGGACACTTCCCGATCCCGTTCGCGGCCGGTCCGC
CCCAAAGCTTTCCACAGATCTTCGGACATGCGTTGTACAATCAATCGAAATTCTCCGGCCTCCAAATGTCAAA
GGACATGGAAGTACCGCCTCCTCCGCCGCAGAATCCATTGGCCGACACGTTGAGCGCGGCAAGTGCGGCG
ATCGCTTCCGACCCGAATTTCATAGCGGCGTTGGCAACGGCGATGACGTCGCTGATCGGCGGGTCTCATCAT
CAGAAGGAGAATAGTAATGGCAATAATAATGTGGATAACAATACAACTAGCAACTCCCAACAGTAA

>LcWRKY38 Chr08:7998714-8001062 WRKY transcription factor 1-like protein

ATGGACCGCCGTGTTCGTACAAACCCATTTCTCTCCGAGCAAGAAGATCCTGAAGCCACGTCGGACGACGG
CCTGCCGGAGTCCCCCTCCGACGGCAATGACTCTAAACCCGCCGCCGGCCCGCCGCCCAAAAAGAGTAGAC
GGGGAGTGCAGAAAAGAGTGGTGTCAGTACCGATCGGCGACGTGGAGGGATCTAAGAGCAAAGGGGAA

GCATATCCACCGTCCGATTCGTGGGCATGGAGAAAATACGGCCAAAAGCCAATCAAGGGCTCTCCTTATCCC
AGGGGATATTACCGATGTAGTAGCTCCAAGGGCTGCCCAGCAAGAAAGCAAGTAGAGAGAAGCCGTGTGG
ACCCCACCAAGCTCGTTATTACCTACGCTTTCGACCACAACCACCAACTCCCGGCCACCAAATCTCACCACCA
CCACCACCACAATTCCTCGCCGTCCTCCGCCTCGATCGCCGCCGCAGTCGCCGTCCCCGACGACTCGTCACC
GGGGAGCACTACGACGTCGTCCTCCACTTCCTCCGGCGACAACACGAACGCCACCCCGACGTCGCCGACG
GCGGCGAAGTTCGAGGAGGCAGCGGCGGTTTTCGCGAGTCAGCCGGAGCTGGAACTCGGCGGCGATTCG
CTGATGATAAAACCGTGCATCGGGGATTTTGGATGGTTTGCTGACGTGGCGTACGATAGAATCCTGGAGGG
CCCGATTTGCGGGGGAGGCGACATATTCGACGACGCCGATGTAATGGTTTTGTCGACAAGAGGAGATGACG
AGGAGGAGTCGTTGTTTGCGGATCTTGGGGAATTACCCGAAGGCTCGGTGGTTTTCGGCCGGCGGCGAGC
GGTCGAACCGGACGGACCGAACCGGACGTGTGGCAGAGTTTTAGACTGCTAG

>LcWRKY39 Chr08:8985724-8987601 PREDICTED: probable WRKY transcription factor 71 isoform X1

ATGTCGAATGATGAAGGGAAAAATAATCTGTACCAGCAGTACGATCCATTTCACTACAATCAGCTGGATATGA
ACCGTTCGATCTTCCAGCAAGAGTCGTTGGATCCAGCTTTCATGAGCTTCACGAACTTCTTCGACACGTCGTT
GGACTACAACAGCCTGTCGAGGGCGTTCGACGTGTCGTGCTCTTCATCGGAAGTGATTTCGGCGGTGGACG
ACATGTCGAAAATCAAGAAGGCGGCGGCGGCGGCTTCTTCAGGTGGAGGGAAAAGTAGTTTAAACAATAA
TAACCAATCGTCGACGCCAAATTCTTCAGTGTCGTCTTCGTCGAATGAGGCTGTAGCTGAAGAAGATTCAGT
CAAGAGCAACAAAGACGACACCAAAGGGAGCCAGAATAAAGATGAAGAAAAGTCCTCTAAGAAACAGAA
CATAGCAAAAAAGAAAGAGAAACGGCAGAGGGAGCCCCGCTATGCTTTCTTGACTAAGAGCGAGATTGAT
CACCTTGAAGATGGATATAGATGGAGAAAATATGGACAGAAGGCAGTAAAAAACAGTCCTTACCCCAGAAG
TTACTATAGATGCACGAGTCAAAAATGTTTAGTGAAAAAACGAGTAGAAAGATCATACCAAGATCCATCTGTG
GTGATAACTACATACGAAGGCCAACACAACCACCATTGCCCAGCTACACTCCGAGGACATTCTGCAGGAATG
ATGTCGTCGCCCTTTTACGCATCGGCATCGACATCGGTAACGGCAGCCTCCTCCGGGCCTACCCTCCCCCAAG
AACTCTTCTCGCATCTGCTACCGATCACCAACTGCCCGACCGACCCCGCGTCGATCATGTACCAAAATCTAAA
CCTCCAACAACACCTTCAAATGCCTGATCATTATGGTTTGTTGCAAGATTTGTTCACTCAAAAATAG

>LcWRKY40 Chr09:974949-976801 probable WRKY transcription factor 75

ATGGATAATTACCAAATCTTCTTTCCAGCTTCTTCGTCGTCTAATAACTCAGTGCCAATGGGCATGGAATTATTA
ACAGAAGAAGACCATCAGGAGGAAGAAGAAGAAGAAGAAGAAGAGGAGGAGGATGATGAAATTAAGAA
TAATAATGGAGGGAAATTGAAAGGAGAGACGAAGAAGATTAGAAAGCCCAGATTTGCTTTTCAAACAAGA
AGCCAGGTTGATGTGCTTGATGATGGCTACCGATGGAGAAAATATGGTCAAAAGGCTGTTAAGAACAACAA
GTTTCCCAGGAGCTATTACAAGTGTTCGCATCAAGGATGCAAAGTGAAGAAGCAAATCCAACGGCTAACAA
ATGACGAAGGCGTTGTATTAACAACCTACGAAGGAGTTCATTCCCATCCCATTGAAAAGCCTCAAGATAACTT
TGAACATATCTTGACCCACATGCAAATTTACCCTTCCTTTTAG

>LcWRKY41 Chr09:3028755-3033619 probable WRKY transcription factor 3

ATGGCTAAGAAAGACGACTCTGCAAGAGCTCCACTCCAGCGCCCCACAATTACTCTGCCGCCTCGCCCTTCC
ATCGAGGCCTTTTTCGCCGGCGGACCCACTGGGGTTAGCCCTGGCCCTATGACTCTCGTCTCCAGTTTCTTT
GCCGACGCTGCCGTTGACTCGCCCTCCTTCTCCCAGCTTCTCGCCGGAGCCATGGGCTCTCCCATGGCTATG
GGCTTTCTCGGAACTGGCTCCACCCCCAATTATTACGCCAAGGATGGAGGCGCCTCCGAATTGGAATTTGGT
CTCAAACAATCCAAGCCGCTCAATTTGGTCGTCGCTCGCTCTCCTTTGTTCTCGGTCCCGTCGGGGCTCAGCC
CCTCTGGGTTGCTTAATTCACCGGGGTTTTATCCTCCTCAGAGTCCATTCGGAATGTCGCATCAGCAGGCATT
GGCTCAGGTTACTGCTCAAGCGGCACTGGCACATTCTCATATGCATATGCAGCAAGCTGAATATCAACATTCT
TCGGTACCAGCTCCTACGGAAACATTGACACGCGATCCATCATTTACTCTTGATGAAGCATCTCAACAGTCAA
TATTACCCTCCACATCAGACACAAAAAATCTCATTGCAGAATCAACAGAAGTCTCTCACTCTGATAGAAAATA
CCAACCTCCTCCTCCCCATGCCTCTGATAAACCTGCAGATGATGGCTATAACTGGCGTAAATATGGACAGAAG

CTGGTTAAGGGCAGTGAATATCCACGAAGCTATTACAAATGCACACATTTAAATTGCCCCGTTAAAAAGAAG
ATCGAGCGCTCGCCTGACGGTCAGATTACCGAAATTATTTACAAAGGGCAGCACAACCATGAACCTCCTCCA
GCCAACAAACGTGCAAGAGATAATAGCGAACCAACTGGGTGTACAAATTCTTTGATGAAGCCTGAAAGTGC
ATCCCAAAATCAAGCTGGAATTTTAAACAAGTCAAGTGAAAATGTGCAATTAGGGTCTAGTGACAGTGAAG
AACAAGCTGATACGGAGATAACAGACGACAGAGATGAAGATGAGCCAAACCCCAAGAGGCAGAACATTGA
GTCAGGGACATCTGCTGTTGCTTTGTCACATAAAACACTCACAGAACCAAAAATCATTGTTCAAACTAGAAG
TGAGGTCGACCTGTTAGATGACGGTTATAGGTGGCGCAAGTACGGGCAGAAAGTGGTTAAAGGAAATCCTA
ACCCTAGGAGCTACTACAAATGCACTAGTGCTGGGTGCAACGTCCGAAAGCATGTAGAGAGGTCTTCAACA
GACTCGAAAGCTGTCGTAACTACGTACGAAGGCAAACATAACCACGATGTTCCTGCAGCTAGAAACAGCAG
CCACCACACAGCCAACAATACAGTGCCCCAGATCAAACCACACAAAGTTGTAGCTCAGAAACATCCATTACT
TAAAGAGATGGAATTTGGAAGTAATGACCAGAGACCTGCCGTTTTGCGGCTTAAAGAAGAGCAAATCACCG
TGTAA

>LcWRKY42 Chr09:46205239-46207860 probable WRKY transcription factor 75 isoform X1

ATGGAGAATTATCAGATGTTTTTTCCTTGTTCCGGCGGCGGCGGATTGTCGGCGGCTTACCATCAGGCCGAC
ATGTCGTCCGGCGGCGGGTCTGATATGTTCGGGAGTTTTCACGGCGGCGATCTGCAGGGAAGTGGGTTTTT
GGGGCTGAAGACGGAGGAGGTGGATGCGGCGGCGGTGGAGGGCGGCGAACAGTACCAGATCGGCGGC
GGCGGCGGGAAGAAGAAGGGGGAGAAGAAGGTGAGGAAACCGAGATATGCTTTTCAGACAAGGAGCCA
AGTTGACATTCTTGACGATGGTTATCGTTGGAGGAAATATGGTCAAAAAGCTGTCAAAAACAACAAATTTCC
TAGGAGCTATTATAGGTGCACACATCAAGGGTGCAATGTGAAGAAGCAAGTGCAAAGGCTAACAAGAGATG
AAGGGGTGGTGGTGACAACATATGAAGGCATGCACACACACTCCATTGATAAGCCAACAGACAACTTTGAA
CAAATCTTGAGTAGAATGCAAATTTACAGTACTCCTTTTTGA

>LcWRKY43 Chr10:403137-404714 WRKY transcription factor 1

ATGGCCTCCTCTTCCGGGAGCTTAGACACCTCTGCAAATTCCCATCCTTCTTTCACTTTCTCCACTCATCCTTTC
ATGACTACTTCTTACTCTGATCTTCTCGCCTCCGGCACCAACGATCCCCCTTCCTCTGCCGCTGCTCTCCGGGG
CTCCGGCACCGGAGTTCCCAAGTTCAAATCCCTCCCCCCTCCTTCTCTGCCTCTCTCTCCGCCTCCTCTCTCTC
CTTCTTCCTTTTTCGCCATTCCTCCTGGCCTCAGTCCGGCCGAGCTCCTTGATTCCCCTGTTCTACTCAGTGCT
TCTCATGTTCTGCCGTCGCCGACCACCGGGAGTTTCCCGTCTCAGTCGTTGAATTGGAAGAGCAATTCGGGG
TATAATCAGCAGAGCATCAAAGAAGAGAATAAGTTCTTGTCTAACTTCTCTTTTCAAACTCAATCGTCTAAGC
CGCCGCCGACATCTTTTCAGCCTTCATCCACCACAGCTCCGACGACTCAGGGATGGAGTTTCCAAGAACAG
GGAAAGAAACAGGACGGATTTTTGTCGGAGAAGAATATGGTGAAGCCGGAGTTCGGATCGATGCAGAGCT
TCTCGCCGGAATATGGGGTTGTGCAAAACCAGAGCCAGAACAACGGCGGCGGGGAGTTTCAGTCCGACTA
CGGCAACAATTACCCTCAACAATCTCAGACGTTGAATCGGAGGTCCGATGACGGCTACAACTGGAGAAAAT
ACGGCCAGAAACAGGTCAAAGGAAGTGAAAATCCGAGAAGCTATTACAAGTGCACCTTCCCCAATTGCCCA
ACGAAGAAAAAGGTCGAAAAATCCTTAGACGGACAGATCACAGAGATCGTTTACAAAGGCAGCCATAACCA
CCCGAAGCCCCAATCCACGAGGAGGTCGTCCTTGTCGTCGGCCGGTTCTTCCCACGCCATGGCGGCTTCAA
ATCCGGCCGCCAACGACATGGGGGACCAGTCATTCACAACCCAAGGAAGCGGCCAATTCGACGGCGTCGC
AACGCCGGAGAATTCCTCGATTTCAATCGGCGACGACGAATTCGATCGGAGCTCTCAGAAGAGCAAATCCG
GAGGGGACGATTTCGATGAGGACGAACCAGAGGCGAAGAGATGGCGAAGGGATGGTGACAACAGCGAA
GGGATTTCTGCAGCTGGTAGCCGGACCGTGAGAGAGCCGAGAGTCGTCGTTCAAACCACCAGCGACATCG
ACATTCTCGATGACGGTTACCGGTGGAGAAAGTACGGCCAGAAAGTGGTGAAAGGAAACCCGAATCCTAG
GAGTTACTACAAATGCACAAACCCAGGATGCCCAGTAAGAAAGCATGTAGAGAGAGCGTCACACGATCTAA
GGGCAGTGATCACCACCTACGAAGGCAAGCACAACCACGACGTCCCACCGGCACGTGGCAGTGGAAGCCA
TTCCCTCAGCCGTCCATTCCCGCAGAGCGAGCCACCGGCCGCCGCAATCCGCCCATCGGCGATGACCCATCA
GTCAAACAACGGCGGCAACAACAACAACAACAACCTTCTGCAGGGTCTGAGGCTGCAGCAATCTTCAGAG

AACCAAATGCCATTCACACTGGAAATGGTGCAAAATCCGAATGGATTTTCATTCCCGGAATTTGGAAACTCC
ATGGGAATGGGATCCTTCATGAACCAAACACAATCCAACGACAATTTGTTCAGTAGAGCCAAAGAAGAGCC
AAGAGATCACGACACGTTCTTCCAATCTCTCTTATGTTGA
>LcWRKY44 Chr10:34586277-34589174 probable WRKY transcription factor 75
ATGATGGAAAATTTCCCAAATTTTCGTTCAACTTCATCTTCTTCTTCAACCTCCTTCTCTCTCCAACAAATGCTG
ATTTCTCGAGCTTCCGAGATCGCCGGAATCCATGGAGGCCATTTCGGACAGACGCCCATCTCCGACGGGTTC
CCGTTGTTTCGCGACGTTTCGACGAACGACATGTCGGTCGATGCGACGAGAAGTACGAAGGACGACGTCGT
TTCGATGAGTAAAAGCTTCTTCCATGTTGATGGGTCAGCAGAAATTGGAGTGGATAGGTTGGAGAAGAAGG
CCGTAGCCGGGGGAGCGACGACGGCGGTGACGATGACGACGGGACGGGGAGATTATGATAAGAAAAAGA
AGACGAGAAGCCGGAGATTTGCTTTTCAAACGAGGAGTCAAGTTGATATTCTTGATGATGGTTATAGATGGA
GAAAGTATGGACAGAAGGCTGTGAAGAACAACAAATTCCCAAGAAGCTACTATAGATGTACACACCAAGGT
TGCAAAGTGAAGAAGCAAGTTCAAAGGCTAACAAGAGATGAAGGTGTGGTTGTGACAACTTATGAAGGCA
TGCATTCTCATCCCATTGAGAAATCCACTGATAACTTTGAGCATATTTTGAGTCAGATGCAAATTTACACTTCTT
ATTGA
>LcWRKY45 Chr10:38559010-38560834 probable WRKY transcription factor 48
ATGGAGAAGAAATTTGAAGACTCATTGATGGGTTCTGAAAATTCCGCCGGATTTTACTCTCCGGCCCTGTTC
TCCGACGACTTTCCGGCCACTGGGTTTGGTTTTTCCGGTATGTTCGACATGCCATGTGACGATGATCATAAAG
CTTCTTGTTCTTCGAATTCCTTTGGAATTGGAATCCACGATCTTTATAATCCTTCTTCTTTGTTCGATTTGCTTTC
CACGGCGGCGCCGCCGCTGCAGCAGCCGCTTTCCTCGCCGGCCTCCACCGTGCCCGAGTCGTCCGAGGTG
CTCAATGCTCCGGCCACGCCGAATTCTTCGTCGGTTTCGAATTCCTCCTCCAATGAAGCTGCGGCCATTGAA
GAGGTCGACAAGAACAACAACACTGACAAGACTTCTAAAGTGTTGAAGCCAAAGAAGAAGAACCAGAAG
AAGCAAAGAGAACCCAGATTTGCTTTCATGACAAAGAGTGATATCGATCATCTCGACGACGGTTACCGGTG
GCGAAAGTACGGCCAAAAGGCCGTGAAGAACAGTCCTTATCCCAGAAGCTATTATCGTTGCACTACGGCAG
GGTGTGGAGTGAAGAAGAGGGTGGAGCGGTCATCCGGCGACCATTCGATCGTCATCACAACGTACGAAGG
TCAACACACCCACCAAAGTCCGGTGATGCCCCGAGGAAGCATCCGAGTCCTACCGGAATCCACCAACTGCC
TCGTCGCTAATCACGACGCCGCCACCCCCGGTCTTCTATTCCAACACAACACTCCCCAGCCCTTCATGTACAG
CCCTCCGCCGCCATTTCTAACGATCGACTCGTCGTCGGTGAGTCCCCATCCTCCGTCGTCCCTCCCACCGCGG
TCGTCTCGAGATTCGTTGCTTCGAGACCATGGGCTTCTGCAGGACTTGGTGCCATTGCAAATGAGGAAGGA
ACCAAAAGATGAGCAAAATGGATGA
>LcWRKY46 Chr10:45285901-45287860 WRKY transcription factor 22
ATGAAAATGGAAGTTGATTGGGATCTTCACGCCGTCGTCAGAGGCTACTCCGCCGTGTCTTCCGCCGCTTCC
ACCGCCGTCACCGCCGCCGTTCCCTCTTCTGACTTTTACTCTGTTCCTTCCATTTCCAATAACTCTATCCCTCTT
GCTTTTGGTAGAGACCCAACGAACCAAACAAACCATTTATTCTCTCTTCAAGATCCATTTCAAGGACCCAATT
GTAATTCCACTGAGGAATTGCATGAGCTTTTCAAACCCTTTTTTCACAAATCTCAACAGCCCTCTCCGCCGCC
GCCTCCTCCTCCGCCGTCTGCTCCGCCGCTTCTTTCTTCTCCGGCGGGTAAAATTCTGACCCATCAGAAACAG
CAGCAGAACACCCATCTCCCCAAACAGCTCCATTCTTCCCCTGTTTCTGCCCCAAGATCCAAACGCCGGAAG
AATCAGTTGAAGAAAGTCTGTCAAGTTCCGGCGGAGTCTCTGTCTTCGGACATTTGGGCTTGGCGAAAATAT
GGCCAAAAACCGATCAAGGGATCTCCATATCCAAGGGGATATTACAGATGTAGCAGTTCCAAGGGTTGCATG
GCCCGAAAGCAAGTGGAGAGGAACAGATCCGACCCCGGAATGTTCATAGTCACGTACACGGCGGAGCACA
ACCACCCGGCGCCGACTCACCGGAATTCCCTCGCCGGCTCCACCCGTCAAAAGCCCATCACGCCGACGCCC
GCCGCCGCAGAACCCGGATCCGGAAAACCCGACCCGAAACAGCCCGGTTGCTCGTCGGAAGACCAAAGTA
CGATCACGGAGAGCAAAGAGGAAAAAGAGGAATTATTGGCCGAAGATGAAGAAGACGACGACCTGGGGG
TCTCCGATTTGATCGTCAACGACGATTTCTATGTGGGTTTTGAAGAACTCGACAGTCCAATCGCCGACGACT
GCTTTTCCGACCAGTTTCCGGCGAATTTCGAGCTTCCGTGGCTGTTCAACAATGCCGCCGCCGCCGCCAGCA

GCATTTAA

>LcWRKY47 Chr10:45320615-45323824 PREDICTED: probable WRKY transcription factor 75

ATGGAGGAACATGACCAACAATTCCCACCTCCGCCGCCGTTTCCGCCGCCGTCATGTACGGACCCCCATGCA
GCCTCTCTGGAAATCGACTGGATCGCAGTTCTCTCCGGCCAAGTCACCGGAGACTTCTCACCGGCGGCAGC
AACCTGCGAGTCATCGGAAATGAAAACCGACGAAGAGAAGGGGAATTCGATGAGGAATGGCGGCCGCCG
GTGGAGGAGGGACGCCCGGCGACGGAGGTTTGAGTTTCAGACGAGGAGTGCCGAAGATATTCTCGACGA
TGGGTATCGGTGGCGAAAGTACGGACAGAAAGCTGTTAAGCACAGCCTCTATCCGAGGAGCTATTATAGGT
GTACACATGTGACATGCAATGTGAAGAAACAAATTCAAAGGCTATCAAAGGACACAAGCATTGTTGTGACCA
CTTATGAGGGAATTCACAACCATCCTTCTCACTTCCTCATGCAAACCCTAACTCCTCTTCTCAAACAAATTCAA
TTCCTTTCCACTTTCTAA

>LcWRKY48 Chr10:45669746-45672602 PREDICTED: probable WRKY transcription factor 46

ATGGAACACAAGAGTTTGATCACCGAGTTGACTCAGGGGAAGGAGATGGCGATCCAACTCAGAACTCATCT
TCAACCTTCTTCTTCTTCCCCAGAAGCTTGCTTTTTCTTGACTGAAATGATTCAGTCTTCGTTTGAGAAAGCA
CTTTTGCTTCTGAATTATTATTCCAACCCTTCTGATTCAATAATGGCTGCCGATCCATCGCCCAAAGTTTCTTTC
GTGGAAGAGGAATCCACCAAGAACAAGAGGCAGAGCAGCAAATCATCAGACGTGTCCAAAAAGAGGAAA
TTGCTGCCTAGATGGACTGAGGAAGTTAAGGTTTGCAGTGGAACTGCTCCTGAAGGCCCTCTCAATGATGG
CTATAGTTGGAGAAAATATGGCCAAAAAGATATCCATGGTGCTACCTTTCCAAGATGCTATTATAGATGCACTC
ATCGACATGTACGGGGATGTTTGGCGACGAAACAAGTACAAAGATCCGACAATGACTCGAACATTTTCGAG
GTGACATATAGAGGACGACACACATGTAACCAATCCTCTAACTTAGGATCCACTTCGATTTCAGCTCAAAATG
AAATCCCCGAAGAAACCAATCAAAATCAACCAAACCCCATTTCTGAAATCTTGTTCAATTTCGGAGACCAAC
AACATTTCAATGTCAAAACAGAGGATCATTTTGAAAATCTTTTCCCGCCCTTTTCCTTCCCCTCGTCGTCGATC
GGATCGGCAATTGGGGACGAAAATGTGTTCTATGAGCCGACGATGGAGCCGGCTTTTATTACCGACACCGG
CCTATCTGCGGTGACGGTGTCGCCGGCGGCATCGGAGAAGGCATGGGATTGGAGTTTCAACGACGGAGGC
GACGGAGGAATTCAGAGGGTTCAGTCGTCGGAGCAATCGGACATGACGGAGATTATTTCAGCGACCACATC
AGTGACCAATTCGCCGATCAACAATGGGGATTGGGATTTCTCGCTTGATAAGGTTGATTTTGACCAGAATTT
CCCTTTTGACTGTCTAGATCTAGATTTCATCTGTTGA

>LcWRKY49 Chr10:46586602-46588639 probable WRKY transcription factor 12 isoform X2

ATGGAAGGATCAGAAAGATCAGGGGTTCACAATTATCAAGTTCAAGTCTCCTTCCTCTCAAATGCACCGCAT
CCGCAACCCATCCACGAAATGGGATTCGTCCAGTTCGAAGACCACAACCAAGTCTTGAGCTTCTTGGCACCC
AACAACGCCGCCACCACTGCCACTGCACCCACCGCCACCATGCCCGCTGCCTTCCCATCGCCTCACGCCCAC
CTCGTCAGTAGTAGACCTTCTTGGAGTAACGAGCAGGTAGGAACACTGGATCCGAAGCCCGCTCACGACGA
GAACTGCACCGGCAGCGCAAGCGACGGCAGCAATTCATGGTGGAGGAACTCGAATGCAGATAAGAATAAA
GTGAAGGTGAGGAGAAAGCTGAGAGAGCCACGTTTCTGTTTCCAGACCAGAAGCGATGTAGATGTGCTCG
ACGATGGCTACAAATGGAGAAAATATGGACAAAAAGTCGTCAAGAACAGCCTTCATCCAAGAAGCTACTATC
GTTGCACGCACAGCAACTGCCGAGTGAAGAAGAGGGTGGAGCGACTGTCGGAGGACTGCCGGATGGTGA
TCACCACCTACGAAGGCAGACACAACCACTCCCCTTGCGACGACTCCAATTCTTCAGAGCACGAACCCTTTA
CATCTTTCTGA

>LcWRKY50 Chr11:858082-859913 probable WRKY transcription factor 53 isoform X2

ATGGAGAATTTGGGGGAGTGGGAGCAGAAGAATCTGAAAAATGAGCTGCTTAAAGGGAAGGAATTGGCT
AAACAACTCCAAATTCATCTCAATGTGAGGCCATCATCATCATCAATGGCTGCTTCTTCTTCTTCTTCTTCTTCT
TCTTCACATGATGGTGGTGAAATTTTGGTTCAGAAGATTTTATCTTCATATGAAAAGGCACTGTCATTGCTCTG
TTCAAATGGGATCCAAAGATCTGAATCTCCTTCCTCTCTTAATGGAAGTCCAAGGAGTGAAGACTCTGACCAT
GAACTAAGAAGTGCTTCCCGCAAAAGGAACATTCTTCCAACTTGGACACAGAAATTCCAAGTTAGTCCAGG
GATGGCCCTTGAGGGCTCTCTTGATGATGGCTTTTGCTGGAGAAAGTATGGTCAAAAAGGCATTCTTGGTG

CCAAACATCCAAGAGGCTACTATAGATGCACACACAGGAACCTTCAAGGTTGTCTTGCAACGAAACAAGTTC
AACGCTCGGACGACGATCCGACCGTCTTCGAGATCACGTACCGAGGAAACCATACTTGCAGCCAAGTCTCC
AACCTCAGTACTCCATCTGCAATAACACCAGAGTTTCAGCAACAAAACAATAGAATTGAATCAAATTTGGTGC
AAAGCCACCCTGCAGTTCATGACCAAAAGGTCCAAAACCAACAGACATCACCTGATGCTCTCTTGAACTCAT
GGGCATCTTTAAGAGTCATAACTGAAAACCTTGACACAACTCACGAACCAACATTGTTTCCTCCTTTTAGCTA
TGATCCCACATCAAACTACGAGGCTGCAGACCGTGTCGAGTCGACGTCGGCCGTCGACGTGAACTTTGCGG
AGTTTTCGCCTTCGTTTTTGTCCCCAACAACATCTGGTTCTGGGTTGAGCTATTTCTCTGCCTCCTCAAGTGG
GTTGAGTGAAGGATTTGTTGGGAATCAGAACTTGCAAGGAAGGAAATCTGAGTTATCTGAGATTTTTTCCTC
ACCAACTTCAGGTGTAAACTCTCAAACTCTTGGCTTGGAGTTCCCATTTGGTGGTCTTGAAATGGAGCCAAG
TTTCACTTTTGACAACACAAATTTCTTCTCCTAA

>LcWRKY51 Chr11:5134386-5139438 probable WRKY transcription factor 9

ATGGAGATTGATCTTTCACTGAAAATTGATCATCACAGAGAAAAAAGTCAGCAAAAAGATGAAGAAAACCA
AGAAGAAGAAGAAGAAAAAAACCATCAACTTCAAGAAAAAGAAGAAATGGGAGTTGAAGAAGAAGAAG
CTGAAATTGATTTGGCTGCTGCCTCTGGTTTGAAAGTGTTTTTGCCACACAACATTAATGCAGGAGAGATTT
CGGAGTTGCAGATGGAAATGGATCGGATGAAGGAAGAAAACAAGATGTTGAGAAAAGCAGTGGAACAAA
CAATGAAGGATTATTATGATCTTGAAATGAAAATTGCTATCATCCAACAAAACAATCTTCACAAAAAGGATTCT
CACAACTTTCTATCATTCCATGGAAATGAGAACAAGAGGCAAGAAGAACCAAAACCAGATCACCTCGAGCT
CGGAGAAACGGCAAAGAGAAGACGAGTTCGGTCGCCATCGAAGGAGGACGAAATGAGAGAGAGGCAAC
TAGGGTTATCATTAGGGCTTCATACAGACAATGATTTGGAACAAGAAAACCATAAGGAAGAAGAAGAAACA
AGAGAAAAGAACAAGGAATTAGATCCAACAACCAACTTCAATTCAATCCAAAACAAGCCACAAAGGCCTGA
GTTGCAAGGAATGGCACCCCCACAAAACAGAAAAGCTAGGGTTTCTGTGAGAGCAAGATGTGAAGCTGCC
ACAATGAATGATGGTTGCCAATGGAGAAAATATGGACAGAAAATTGCAAAAGGAAATCCATGTCCTCGAGC
ATACTATCGTTGCACAGTTGCACCGGGTTGCCCTGTTAGAAAGCAGGTCCAAAGATGCTTAGAAGACATGTC
AATTCTCATAACAACGTACGAAGGAACACACAACCATCCACTCCCTGTGGGAGCCACAGCCATGGCTTCAAC
AGCTTCTGCAGCTGCTTCCTTCATGTTATTAGACTCTACAAATCTTTCTCTTCCAAACCCTCAAAACCCTAATAT
TCTAAACTCGTCTTCGTATTCTCCGACCTTCCTTGGATCGGCAAACCCTAATGAACCCTCGAAAGGGCTTGTC
GTCGACCTCACAAGCAACAACTTCTATAGTCCGATGGCGAGCTCGTCGAGCTCGGCATTGGCTCAACCGTCC
AATTGGATGAAAACAAACAGCCATTCGTATTACCAAAGGAACTTTCAAGCTAATCTTTTGAGTCCCTTTGATG
GGCGGACTTGGAAACCGGCGGAGGATAATAAGCCACCATTGACGGCGGAGAGTGTGTCTGCCATTGCTTCT
GACCCTAAGTTTCGAGTCGCGGTGGCAGCTGCCATTTCGTCGCTCATTAACAAGGAGAACAACCACCCGAC
TGCACATCCGGTCGAACATTCTTCTTTCGGTCCCAATAAGGATGGCGAAGGAGGTGGTGGCGGCGGCGATA
ATGGTGGCGGAAACAAGAAATGGGTTGTCGAATCGCTCTCGACGAATGGTAACTAA

>LcWRKY52 Chr11:8150570-8155637 probable WRKY transcription factor 41

ATGGAAAGTGGGTGGAGCTGGGATCAAAAGTCACTCATTGGTGAGCTTATTCAGGGGATGGAGCTTACCAA
GCAATTGAGAGCAGAGTTGAGTTCAGCGTCTGCAGAAGAGAGCAGAGGATCATTAGTACAAGGGATTTTAT
CTTCATATGAAAAGGCTCTTTTGATACTGAAATGGAATGGACCAATGAGTCAGCCTCAGACTGTAGAACCAA
CCCCTGGTTTGCCAGGCTCTCCAATTTCTGTTAATGGAAGTCCTTCTAGTGATGACTCTGGTCGAGGCCTCAT
GGGCAACCAGGACCCCAGAAAGGAATCAAAGAAGAGAAAGACACAGCCCAGATGGACAGAACAAGTGA
AAGTGAACTCTGAGACGGGATTCGAAGGACCCCACGAGGATGGTTATAGCTGGAGAAAATACGGCCAAAA
GGACATACTTGGTGCAACTTATCCCAGAAGCTACTATAGATGCACTTTCCGTAATACTCAGAATTGTTGGGCA
GTAAAGCAAGTGCAGAGATCAGATGAAGACAATTCTATGTTTGAAATTACATATCGAGGAAAGCACACTTGT
TCCCAAGGGAACTATTTAGCCCAATCATGTCATTCACCAGAAAAGAAGGAAAACGACCATGACCACGATCAT
CATCAGAAGCAGCCTTTGCAAGAGAACTTACTCAGTAATCAAACTATTGAGAACATCGAAAAGCTTGAAACC
AAGGCATCTACCTTCTGCTTCGGCTCAACTTCTGTTGGATATAAGGACATAGTAAATGGTGGCTTTTCACATTT

AGCCATAGACACTCACACCGCCTTGGGAAGCTTTTCTCAGTCATTTATCTCCCCAACCACACCTGACTCAAAC
TATTTCACACCATCCTCATGCCAAAGGAGCAACGTGGGCGGGACTCATAATGTGCAACATCCAGAATCTGAT
GTCCATGAGATTTTCTCAGCCAACAATTCAGCTACCAATTCCCCTATCCTGGATTGGGACTTTCCATTTGATTC
AGATCAGATCAACCCGAATTTCCCATTTAATTCCTCAGGATTTTTCTACTAA

>LcWRKY53 Chr11:8595284-8596734 probable WRKY transcription factor 43

ATGGCAAATAGTTGCAGTAGTAGTAGTTCATTGATGATGATGATGAACTGCAGTACTGATCAGGTTATTGATG
GATCAGGATGTTGTGAAATTGATTGGGCTGGGCTTCTTTCTGGTTCTTGTTTGAGCTTGGAGATGGAGAAG
AAGGAAAGTGGAAGTGGGGCTGCAATGGAAACTCATGAGGATTATCATGGAGGGAAGAAGATCAGCAATA
AAGGAAAAATGGTGATGGGATCAAAGAGAAGTGCTGCAATGCCAAGGGTTGCTTTTCAGACAAGGAGTGC
TGAGGATGTTCTTGATGATGGCTATAGATGGAGGAAGTATGGTCAGAAGGCTGTCAAACATAGCACACATCC
TAGGAGCTATTACCGATGTACACATCATACATGCAACGTTAAGAAACAAATTCAAAGGCACTCCAAGGATCCA
ACCATAGTGGTGACAACATATGAAGGAATTCACAACCATCCATCTGAAAAATTAATGGAGACTCTAAGCCCTC
TTCTCAAGCAATTGCAGTTCCTTTCTGGAATTTAG

>LcWRKY54 Chr11:8608668-8610091 probable WRKY transcription factor 29

ATGGAGGATTGGGATTTGCAAGCAATTGTAAAAGGTTGCAGCAGCAGGCCAAATTCAGATCCTTTCTATTCT
AGTTTTTTGAGTGAAGAAGATGATTTTTCTTGTGTTTATCCTCAGCTCTTTGAAACCACAGCAACAACAACTT
CTTCTTCTTCTTCACACGAGTTTGAAGGATTTGGAAGATCGATTTATCCACTGATTCCTCATCCAAACAATTCT
TCCTCCATTTCTGATCTTCTCAGAGAGTTTAAGGAACCAGAAAAGCTTCATCAGAAGAAGCAAATCGCAGCT
ACTAAACAGAAACAAAGCAAGAAAAGTAGACAAAATAGAGTAGTGAAAGAAGTGAAAGCAGACAGCGTAT
GTTCAGATTCATGGGGATGGAGAAAATATGGACAGAAACCAATAAAAGGGTCGCCATATCCAAGAAGCTATT
ACAGATGCAGCTCCTCAAAAGGATGCTCAGCCAGAAAGCAAGTCGAACGAAGCCTCTCCGATCCCGACGTC
TTCGTCGTCACCTACACGGCCGAGCACAACCACGCCGAGCCAACTCGCCGCAACGCCCTCGCCGGGACCAC
CCGCAAGAAGTTCCCGGCGCCGGAGAACCCGAGCTTCGACGTGGTTCTCTCACCCAATAATTCTACTTCTGT
GGCATCCATTGAAGAAGAACAGCCCATGGAGGGTGTGGCAGAGGGGGAAGTCTTGATGAATATGCCATTT
GAAATCTTTAGTGATGATTTGTTTACTGGTTTGGAGGATTTGCTTTTTGGATGA

>LcWRKY55 Chr11:33909996-33920087 probable WRKY transcription factor 20 isoform X1

ATGGACCCCACTGACTCTCACTGGACTTCCACTGACCCCAACCTCGCCGATCCATCTCATGCCCGCCCACCCC
CTTCTTCTGAACCTTCCTCCGCCGCTGCTGCTGGCGCCAAGTACAAGCTAATGTCTCCGGCCAAGCTCCCGAT
CTCCAGGTCCCCTTGCATTACCATCCCTCCCGGCCTCAGTCCCACTTCCTTTCTCGAGTCCCCCGTTTTGCTCA
CCAACTTGAAGGTAGAGCCTTCTCCGACTACTGGATCCCTTACCAAGCTTCCAATGGCACATGACTCTTCTAG
CTCAGCTATTTATTCAATGACCACTATGGCTTTCTCAAATACAAATGCCTCAGATGAAGGAAAATCCAGCTACT
TCGAGTTCAAACCATATGTTGGACCGAATATGGTTCCTGCAGATCTGAGTCATAGGAAAGGTGAACAATCTA
AAGAAGTTCAAGTTCAAGGTCAACCTCTACCATTTACTGCTCCACCCATGACTAAAAGTGAGATCAATGTCAT
TTCAAATGATTTGAGTCGATCCACCCAGATGGATACGGTTGCCTCAGGGGCTAGTGTTCCTGAAGTTGATGG
AGAAGATTTCAACCATAATATGAACACAAGTACCAGGGTTCAGCCCCCCCAGTCTGATCCAAAAGGCAGTGG
CATTTCAGTAGCTTCTGATAGGTTATCTGATGATGGATATAACTGGCGGAAGTATGGACAGAAGCATGTTAAA
GGGAGTGAATTTCCACGTAGTTATTATAAATGTACACATCCTTCCTGTGAAGTGAAAAAGCTCTTTGAACGCT
CTCATGATGGACAGATAACTGATATTATCTATAAGGGTACACATGATCATCCTAAGCCTCAGCCAAGTCGGCGA
TATACTGCCAGTGCTTCTGTGAATGTTCAAGAAGATGGGTCTGATAAGCCTTCACATTTAACTGGCCAAGATG
ACAGGTCGTGCGGCATATATGCTCAGACAGTGCATACCATTGAGCCAAATGGAACTTCAGAACCATCCCTTG
CTGCAAATGATAGCATCGCCGAAGGTGCAGGAACAACTCTGCCATGCAAGAATCATGATGAGGTTGATGAC
GATGACATATTCTCAAAGCGGAGGAAAATGGAACTTGGTGGTTTCGACGTCTGCCCAATGGTTAAGCCAATC
AGGGAACCACGTGTTGTGGTTCAAACTCTAAGTGAGGTTGATATACTGGATGATGGGTATCGCTGGCGCAAA
TACGGCCAGAAGGTAGTGAGAGGGAACCCCAACCCGAGGAGTTATTATAAGTGCACAAATGTTGGATGTCC

TGTTAGAAAACATGTCGAAAGAGCGTCCCATGACCCAAAAGCTGTTATAACTACATATGAGGGGAAACATAA
TCACGATGTTCCGACTGCAAAAACTAGTAGCCATGATGTCGCAGGCCCATCAACAATTGCACCATCAAGGTAT
AGACTTGAAGAGAGTGACACCATAAGCCTTGATCTTGGCGTTGGGATTGGGACAGGTGGCGAGAATCGAT
CAAATGAATACAGGCAGGCATTGCATCCTCAACTGGTTGAAAACCAAACTCCAAATGGCAATTTCAACTTCG
AAGTGGTTCAAGAAAACTCAGCCCCAACCTATTTCGGTGTTCTCAATAGAGGTATAAACCAGCATGGATCAA
GAGAGAGTTTGAGCGAAAGCCACCCAATGGAGATTGCTCCGCTAAACCATTCGTCTCACCCATATCCGCCGA
GCATTGGGAGAATACTAACAGGGCCATAA

>LcWRKY56 Chr11:40122538-40127220 PREDICTED: probable WRKY transcription factor 2

ATGGGGAGGACTGATGATAATGTTGCTATAATTGGGGATTGGGTGCCTCCAAGTCCCAGCCCAAGAACCTTC
TTCTCAGCAATGCTGGGGGAGGATGTTGGTTCTAGACCTGCAATGGATACTACCATCAGTGATAAAACTGAA
GAGCTCTTTCTCAGGCCTCGAGAACATATGGTATCGGAAAACGCCCTTGCAAGAGGCGGGATCCCGGGCAT
CCATTCGAGTGACCGGTCGATGGATTTAGGTTCGTTCTCGGAGCAGAAATTCCGTGGAGGGCTTGTGGAAA
GGATTGCAGCTAGAGCTGGATTCAATGCTCCAAGATTGAATACAGAGAGCATTAGATCCACATCCACAGATC
ACTCGTTGAATTCAGATGTCAAATCTCCTTACTTAACGATACCGCCTGGTCTCAGTCCAACTACATTGCTAGAT
TCGCCAGTTTTTCTTTCAAATTCTCTGGCTCAGCAATCTCCCACAACTGGAAAGTTCCCATTTTTGCCAAATGT
TAGTAATCGGAGCTTGACGATGATGTCGGAGGCCAACAATAAAGGAAGCGACAACCCATTCAACGATAACA
GTACATCATTTGCATTCAGACCCGGTGTGGAATCAGGATCGTCCTTTTTTCTCGGTGCAGCAAGCAAAGCAG
CTTCTGCTACCATTCTTCCACAATCTTATCCAAGAATTGAGGTTCCAGCTCCACGTTCAGAAAATTCTTTTCAA
TCTCATCTAGTAGAACCATCTCTGTCTTTACCCCAAAATAGAATCAGTCATCATCCTCAAGTGGGACTCTCTAC
ATCATGCGCGGAGAGGGACGATGGGGGAAAGACCGTGTCAGACGATCAAAGACCCTTTGATTCTCTGTGT
GGAGGTGGTGAACATTCTTCACCATTGGACGAGCAACCGGACGAAGGGGAGCAAAGAGGCAGTGGGGAT
TCTATGGCTGGTGGTGGTTGCGGTGCTCCCTCGGAGGATGGATATAATTGGAGAAAATATGGACAAAAACA
GGTTAAAGGAAGCGAGTATCCCCGGAGTTATTACAAGTGCACACATCCAAACTGTCAGGTCAAGAAGAAAG
TCGAGCGATCTCACGAGGGCCATATAACAGAGATCATCTATAAAGGAGCACATAACCACCCAAAGCCCTCGC
CAAACCGGCGAGCAGCAATTGGACCTTCTGATTCCCAACTCAATATGCAACTAGAAATCCCTGCACAAGCAG
GCCAACAGAGTGCTGAAGGTCCTCTATGGGAAGATTCACAAAAAGGGATTCCAACTGGAGCTCCTGATTGG
ATGCATGACAACCTTGAGGTGACTTCTTCAGCATCCTTGGGTCCTGAATATGGCAATCAGCCTAACCCTTTAC
AGGCTCAAAATGGCAGTCATATTGAAACAGTTGAGGCCGTTGATGCCTCATCCACATTCTCTAATGATGAAGA
TGAAGATGATAGGGGAACACATGGCAGTATAACATTGGGGTATGAGGGGGAAGGAGATGAATCTGAGTCTA
AGAAAAGGAAACTTGACGCTTATGTAACAGAAATGAGTGGTGCCACTAGAGCTATCCGCGAGCCTAGAGTT
GTCGTCCAGACTACCAGTGAAGTGGATATTCTGGACGACGGCTATCGTTGGCGCAAATATGGACAGAAGGT
TGTGAAAGGAAATCCAAATCCTAGGAGTTACTACAAGTGCACGAATCCCGGCTGCACGGTGAGGAAGCACG
TCGAGCGGGCGTCGCATGACCTGAAGTCCGTGATCACCACGTATGAAGGAAAGCACAATCATGATGTTCCTG
CTGCTCGCAATAGCAGCCACATCAGTTCTGGTACGTCCAGTCCAGTAACCGTGCAAAACTCGACCGCAGCAA
TTCAAAGTCATGTTCACAGACCAGGACCACCCCAGCCTCAGAACACCATTCCAAGATTTGAAAGGCCGCCCT
TTGGCATTGCTGGAAGACAGCAGATGGGCACTGCCCATGCCTTCTCTTTTGGAATGAACCACCAGCCTGGA
CTGGGAAATTTGACAATGGCAGCAGTTGGCCAAGCCAAGCTTCCTGTTCTGCCAATGCATCCATACTTAGCA
CAAGCACACCATGTTAATGAAATGGGTTTCTTGTTGCCTAAAGGAGAGCCCAATGTAGAACCTACATCTGATC
TTGGCTTGAACTTTTCCAATGGTTCAACCGTGTATCAGCAAATTATGAGTAGGCTTCCACTTGGCCCTGAGAT
GTGA

>LcWRKY57 Chr12:44128923-44131108 WRKY11

ATGGCGGTCGATCTGATGAGTTTTCCTAAGATGGATGATCAGATGGCTATACAGGAGGCTGCCTCGCAAGGA
TTGAAGAGTATGGAGCATCTGATTCGTCTTCTTTCTCACAAACAACCTTCAAGCCATGTCGATTGCTCTGACC
TAACTGATGCAACCGTTTCCAAGTTCAAGAAAGTGATCTCTCTTCTCAATCGGACCGGTCATGCGCGCTTCCG

CCGAGGTCCGGTTTCCAATTCGACCTCCTCGTCGTCGTCCGGTTCAGTTCCGCAAAATCAGGCGACGAATCT
CACTCCTACGCCGTTTACTTCACCGGCCACGGTTTCTGCTCCGCCGTTCACCGCTCCGGCTACGGTCGCGCA
GCCGCAGGCGAAGGTTGCTGCGGCGGCGAACTTTCTCCAGTCGCAGCCTCAGAGTATGACTCTCGATTTCA
CCAGGCCGAATATTTTGAACTCTAACCCTAAGGGAACCGATTTGGAATTTTCTAAGGAGACCTTTAGCGTCT
CTTCGAGTTCCTCGTTTATGTCCTCTGCGATCACTGGCGACGGCAGCGTATCCAACGGAAAATTAGGAACGT
CGATCTTTTTGGCACCGGCGCCGACTGCTTCCGGCGGAAAGCCGCCGCTCTCCGCGGCGCCGTACAAGAA
GAGGTGCCACGAACACGATCACTCCGAAGATTTGTCCGGAAAATTCTCCGGCTCTACCTCGGCCTCCGTCAA
GTGCCATTGCTCAAAAAGAAGGAAAAATCGCATGAAGAAGACGATCCGAGTCCCGGCGATCAGTTCAAAAA
TCGCCGATATTCCACCGGACGAGTACTCGTGGAGGAAGTACGGTCAGAAGCCGATCAAGGGATCTCCCTAC
CCACGGGGATATTACAAGTGTAGTACGATGAGGGGATGTCCGGCGAGGAAACACGTGGAGAGAGATCCGA
ACGATCCGGCGATGTTGATTGTAACGTACGAAGGAGAGCACCGCCATACGCAGAGCTCGTTGCCGGAAAGT
ATGGCCGCCGGAGTATCTTTAGTTTTTGAGTCAACTTGA

>LcWRKY58 Chr12:45195090-45198986 PREDICTED: probable WRKY transcription factor 40 isoform X2

ATGGATATTTTCTTGGACCTTAATGTGGATCCTACTTCTTCTTATGCCAATCCAGCCATGGATGAGGGTCTTGA
TTCTTCTAAGAGAGAATTTGAAGCTGGAGAAATCTATTGGGATAAAGAAAAACTCTCACTAAGCTTGGCAAA
TAAGGGAAGTGATTCGAACTCGACATTGGAGGAAGAGTTGGATAGAAAAATCCAAGAGAATGGAAAGCTA
AGTCAGATGCTAAGAGTAATGTATGAGAAATACATGAATCTTCAAAAGCAAGTGATGTATTTGCTGAGCCAAC
AAAAGCAGAACTCAGAAATGGAAGCAGTTTCAAGGAAGAGAAAGGCAGAAGGAGAAGAAGACTATGAG
AATTTGGAAGGAATTTGCAGCAGCTCAAGAGATGAAGATTTCAACAGGTGGCTTAAGAGGCCAAGACTAA
ATGGAAACTCAAAGGTTTCTAAGGTTTTTGTGCAGAAAGATGCATCAGATCCAAGCTTGGTGGTGAAAGAT
GGGTATCAATGGAGGAAGTATGGGCAAAAAGTGACAAGAGACAATCCTTCTCCAAGAGCTTACTTCAAATG
CTCCTCTGCACCAAATTGTCCTGTTAAAAAGAAGGTGCAAAGAAGTTTGGAAGATCCCACAATTTTGGTGGC
TACTTACGAAGGAGAACACAGCCATGCCAGCCACTTTCAAACTGAGCTATCTTTAAGGTCCATCAATGGCGG
CAAAGGCAGTGCAGTCCCAGTCTTAGCCACCATCAAGCCGTCGTGCGCCACCGTGACGCTCGATTTGATTCA
CGAAGATGGGCTGTTCAAGAGTCCGAAAGATTACGCGTCGTCGGAGTCGGTGGTGTCGACGGAGGCAGCG
GTTTGGCAGGAGTTTTTGGTACAACAAATGGCCTCCTCTTTGAAGAAGGATCCTGAATTTGCTGGCATTGTT
GCAGGTGCCATTTCAGGCAAGGTTTTGGCAAACCAAACAAACAGAGAATAA

>LcWRKY59 Chr12:46827117-46830417 WRKY transcription factor 4-like protein

ATGCTCCTTCTTCACAATAATATGGCTGTCGAGCTTATGGTGGGTTTTGGGGATGCTGCTACTAATTTCACGCC
CAAGATGGAGGAAAATGCCGCTGTCTCTGCTGTTCAAGAAGCCGCTTCTGCTGGGATTCAGAGCGTCGAGA
ATTTCCTCCGATTGATGTCTCATACTCCTAATCAACATCACTCCGAAGACCATTCTTCCACTTCCACTGCTCCCA
ATGCTACTGCTGGATACGAAGCCGTCGCCGATTCCGTCGTCAACAAATTCAAGAAGGTCATTTCTTTGCTCGA
CCGGAACAGAACCGGCCATGCCCGCTTCAGAAGGGCTCCCGTTGTTACTACTACTTCCCCTCCGCCGCCGCC
GCCTCCGCCCAAGGTCAAGACGCAGCACCAAGATCCGAGTTCGTCGTCTCCGATTTCGACCCCGCAGATTC
AAGTAAAGAAACAGGAATCTGTCTCTGCTTTTAAGGTTTATTGCCCCACGCCTTCCTCTGTTGTGCGTTTGCC
TCCTCTGCCTCACAACAATCCCCATCAGCCTTCTTCTAATCCGCCAAATACCTTCCAAGCCCAGCAAAATTCTT
CGTCGGTGGTGCTAAAAAATGGGTCTGCAGATAGAAAAGATTCGACTACCACCATCAATTTCGCAGCCTCGC
CCCCTATTTCCGCTGCAAATTCGTATATTTCATCGTTAACCGGAGACACTGAAAGCTTACAGCCATCTCTGTCG
TCTGGATTTCAGTTCACCCACATGTCCCAGGTCTCATCCGCCGGAAAGCCCCCTCTTTCATCCTCCTCGCTAA
AGAGAAAATGCAATTCCATGGACGATTCCGCCATGAAGTGTGGCTCGTCGTCCGGCCGGTGTCACTGCTCCA
AGAAGAGGAAAAACAGGATAAAGAGAGTCATCAGAGTTCCCGCCGTCAGTTCGAAACTTGCCGATATACCA
CCGGACGATTACTCATGGAGAAAGTACGGCCAGAAACCCATCAAAGGCTCTCCTCATCCAAGGGGATATTAC
AAGTGTAGCAGTCTGAGAGGGTGCCCAGCACGGAAACACGTAGAACGTGCCTTAGACGATCCAACAATGCT

GATTGTAACTTACGAAAACGATCACAATCACGCCCACTCCACCGAAACACCTGCAGCGCTTGTTCTCGAATCA
TCATAA

>LcWRKY60 Chr13:2740565-2744212 PREDICTED: probable WRKY transcription factor 3 isoform X1

ATGGGTGATGATGAAGAACAGCTGCCACCGTCGTCAGCGCCGCCTCCGCCGTCGAAATCTAAGGCACTTCC
GTTGAGGCCAACTATTAATCTGCCGCCAAGGACCTCTATGGAGTCGCTCTTCAGCGGTGGGCCGGGTTTGG
GAAGCGGGTTTAGCCCAGGACCCATGACCCTCGTCTCGAGCTTCTTCTCCGATTCCGACGACTGCAAGTCTT
TTTCTCAGCTCCTCGCCGGAGCTATGGCGTCGCCGGTGGCTGTTCCTCCGAGGGCGTCTGAGCTCAAGGGC
CCTGCCGGTCTGCTCGATTCTCCGGGGCTTTTTTCTCCCAGTCAGGGACCTTTTGGAATGACACATCAGCAG
GCTTTGGCTCAGGTTACTATGCAGGCTGCACAAACCTATTCTCATAAGCAAATACAAGGAGAATCTTTTTCTT
CATTGGTACCACCCTCTGCATCCTCCTCAGAGCTTTTGACAACCTTTCCCGGCGAGAAGACTAAAGATCAGC
CGGTGCCACCTTCATTGCTGAACTCTGCGGTTGCATCCAAGGAATCATCGGATATCTCTCAATCAGATCAGAG
ATTGCAATCTTCTTCGTGTAATGTTGATAGGCCTGCTGATGACGGCTACAACTGGAGAAAATATGGACAGAA
ACAAGTGAAGGGCAGTGAATTTCCTCGAAGTTACTACAAGTGCACGCATCCGAATTGTCCTGTCAAGAAAA
AGGTCGAACGATCTCTGGAAGGTCAAGTCATTGAAATTATCTATAAGGGTGAGCACAACCACAAACGACCTC
AACCAAACAAGCGGGCAAAGGATGTTGGAAATTCTAATGGATGTTCAAGTATTCATGGAAATCCTAAGCTGT
CTTCTCAAGTTCAGAGTGGATATTTGAATAAATTGAATGATCAGCAGACGGCTGTGTCTTCCATTGCAAAGAA
GGATCAAGAATCGAGTCATGTTACACACGAACAGTTGTCCAGTACTAGTGATGGTGAGGGAGGGAGTGAGA
TAGAAACTGGAGTGAGTCGAAAAGATGAAGATGAACCTGACGCCAAGAGACGGAATACTGAGGTTAGAGT
TTCTGAGCCAGCATCTTCTCATAGGACACTCACAGAATCCAGAATCATTGTGCAGACTACAAGTGAAGTCGAT
CTATTGGATGATGGCTACAGGTGGCGAAAGTATGGGCAGAAAGTTGTTAAAGGCAACCCTTACCCCAGGAG
CTATTATAAATGCACAACTCCGGGATGCAATGTTCGTAAACATGTTGAGAGAGCTTCGACAGACCAAAAAGC
TGTCATAACAACGTATGAGGGCAAACACAATCATGATGTTCCAGCTGGTAAAATCAGCAGCCACAGTACAGT
CAACAGTAACGTTCCTCAGCTAAAATCACAAAATATAGCTACTGATAACAACACTGATCCTGGGAACTCTCGT
CAACAGCCTGTTGGACTTCTACGGTTAAAGAAAGAACAAATAACGTAG

>LcWRKY61 Chr13:11470918-11477873 WRKY transcription factor 55

ATGGCGAGCCAAAGAAAGTTGCACAAGGACGAGCAAGATCCGGCCATGGAGGACGCCATTTCTTTGATCC
TTCGTGGGTGTTCGTTGGCAAGAGAATTGGAGTTCAATGTTTTGAATTCCGCCAATAATGTTCATTTCCCTCA
GCCCCAGATGGTGGCTCGGTCTTGTGATCAGATTTTGAGTGTCTTTTCCGCCGCCAAGGACCGGCTGAGCG
GCTGTGAACGCCCCCCGTTGACCGCGGTGCTGCGGGAGGTGGGGTTGGAGGACTGGCTGAGGTCCTCCC
AAGCTATGGATCAGTTGGCGCAAATGCAGATGAGGTCGCCGCCACCGCCGCCGCCTTCCGCCGTGGAGGAT
TCGGGGAAGCTGGTGGGAATGGGTTTTGGTTCTTCTTCTTCATCGTCAACAAAACCACGAAGACGAAAAGA
TGATATGGAGAAGAGGACGGTGAGAGTGGCGGCGCCGCGCATCGGAAACACAGAGCTTCCGCCGGACGA
CGGCTTCACATGGCGGAAGTATGGACAAAAGGAGATTCTTGGCTCCAGGTTTCCCAGAGGCTATTTCAGAT
GCACTCACCAGAAGCTCTACCACTGCCCAGCCAAAAAGCACGTGCAGCGCCTCGACGACGATCCCCACACT
TTCGAAGTCACCTACCGCGGCGACCACACGTGCCACATGTCCGCCACCGCCCCCTCCGCTCCTCCGCCACCG
CCGCCGGACGTCGCTGGCCAACACATGGCCCAGTTCCGGCCCCCCTCCGCCGGATGGCTTTCGATGGAGTT
CGTCGGTCCTGCAAGTGGGTCGGGCTCCGGCAGCAGCAGCGGTGGTCCGGCCGCCGTCCGGTACGGAAA
AGACGTCGCCGATCAGTTTCCGGTGCTGGACATGGCCGACGCCATGTTCAACTCCGGCACCGGCGGTGGCA
ATAGTATGGACTCGATCTTCGCCTCTTATGTGGCGGAAGAACATCACCATCACAAGTGGGAGAAAGACGACA
AGAAGAATTAG

>LcWRKY62 Chr13:11493288-11497467 WRKY70

ATGGAATTGGCCGATAATTCTCGCCGTCGATTTCCGGCGGAAGCACGGAGGAGGATTACTTCGCCGCTTGTT
CGCGGCCGAGAATCGACGGTTCGGCTTCAGATCCTTCTCCAGTCGGAGACGGCGACGGATCACGACCGGC

GTGCTCTTGCCGCCGAGATCTTGAGCTGCTTCACCGAGGCAATCTCCGTTCTCCACTCTTCCGAGTCCGCCG
GCGACGAGTTTGGTTGTCCGGATCATTCTCTTTGCTCCGATCTGGATTCCGGCGACTCACGGCGGAGCCCG
GCGGGTAAAGATCTAGGACGTGGCAACAAAAGAAGGAGACTGGCAAACACGAAGATTGTATCATCGGCAA
TGATAGAAGATGGCTATGCTTGGAGGAAGTATGGCCAAAAGGCTATCCTCAACACGACACACCCAAGGAGC
TATTACAGATGCACTCACAAGTACGATCAAGGCTGTGGCGCCACCAAGCACGTGCAGAGAATGGAGGGCAA
TTCAGAAATGTACAAGATCACCTACACCTCCAACCACACGTGTGGCCCGACTTCTCCGATCGCCGCCGTCGTC
ATCGAACCTCCGCCCTCCGCCTCTAATTCTTACAACATTTGCTTCTCCGACGACTCTCGCAACGATCAGTTGGT
TGAGAGTACTGGTTCTTTCTTTTGGTCGAGCAAGAATAATAATGTTGCGAAGGGAGAAGAGACGACGACGA
CGAGTGGTGTGACAGATGAGATGGATCTCTGGTCGGAGTTGGATTTTGGGAGCTTGCAGGAGACGGCGGC
GACGCCAATGACGACGAACCAATACTATTTCTTGAATGGAGACGATGCAGATTCATCTCACATGTTTTGGGG
TGGATTTTAA
