# Supplementary material for: Identification of WRKY Family Members and Characterization of the Low-Temperature-Stress-Responsive WRKY Genes in Luffa (Luffa cylindrica L.)
Source: Plants (Basel). 2024 Feb 28;13(5):676. doi: 10.3390/plants13050676 (PMC10935285; doi:10.3390/plants13050676)
Supplement: Supplementary file 1 [file plants-13-00676-s001.zip › Supplementary File S6.pdf]

Table S2. Consistent sequences of the predicted WRKY motifs in *L. cylindrica.*

Motif	E-value	Sites	Width	Best possible match
Motif 1	1.2e-494	25	29	VLEDGYRWRKYGQKAVKGSPYPRSYYRCT
Motif 2	1.2e-524	25	31	VDILDDGYRWRKYGQKVVKGNPNPRSYYKCT
Motif 3	1.6e-440	25	41	SSKGCPARKQVZRSRDDPSMLIVTYEGEHNHPLPTSRNAMA
Motif 4	8.3e-414	25	30	SQGCNVKKRVERSSEDPSVVITTYEGKHNH
Motif 5	3.0e-383	22	28	IPDDGYNWRKYGQKPIKGSPYPRGYYKC
Motif 6	2.6e-177	10	38	HPNCPVKKKVERSLDGQITEIIYKGEHNHPKPQPNRRA
Motif 7	4.0e-115	25	15	KKVREPRFAFQTKSE
Motif 8	3.1e-080	12	28	GRCHCSKKRKSRVKRVVRVPAISSKLAD
Motif 9	4.4e-091	10	29	QGCPAKKQVZRSDEDPSMFEITYRGKHTC
Motif 10	3.4e-041	8	32	LQEELDRVREENQKLKEMLSQVMKBYNTLQMQ
Motif 11	4.7e-039	9	30	LPDTIAAAASAJTSDPNFTAALAAAISSII
Motif 12	3.3e-029	5	38	SPSPFLTIPPGJSPTELLDSPVLLSNSQVLPSPTTGSF
Motif 13	5.9e-028	11	21	CEALTDAIVSKFKKVJSLLNR
Motif 14	6.3e-020	10	25	MDQKSIIZELLQGLESAEQLRRLLS
Motif 15	6.9e-013	6	10	TGHARFRRAP
Motif 16	2.1e-012	8	13	DHGLLQDIVPPSM
Motif 17	2.4e-012	6	15	PKKARVSVRARCEAP
Motif 18	1.3e-010	3	18	IEKPTDNFEHILSQMQIY
Motif 19	2.5e-004	2	41	LMNPNFLARAJLPCSSSMATISASAPFPTITLDLTQTPNPL
Motif 20	1.2e-003	2	32	FYPPQGPFGMSHQQALAQVTAQAAQAHSHKQIQ
