# Supplementary material for: Identification of WRKY Family Members and Characterization of the Low-Temperature-Stress-Responsive WRKY Genes in Luffa (Luffa cylindrica L.)
Source: Plants (Basel). 2024 Feb 28;13(5):676. doi: 10.3390/plants13050676 (PMC10935285; doi:10.3390/plants13050676)
Supplement: Supplementary file 1 [file plants-13-00676-s001.zip › Supplementary File S9.pdf]

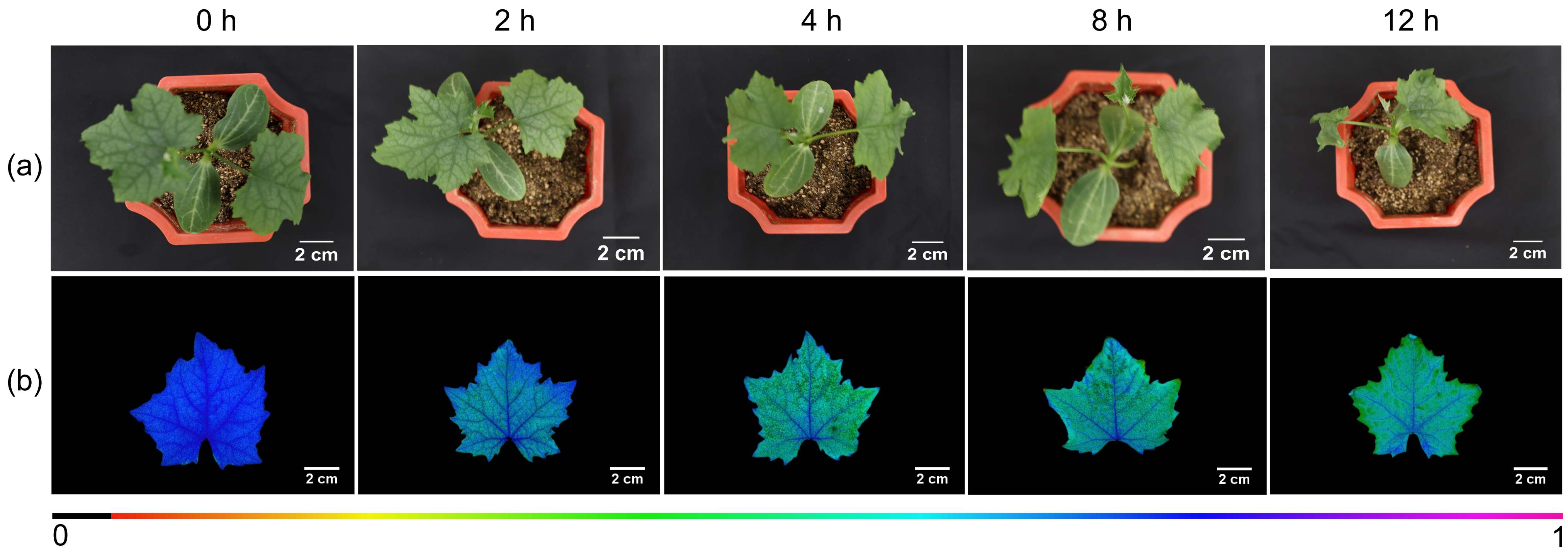

Figure S1. Effects of low-temperature stress on 2-week-old luffa seedlings. The Chlorophyll fluorescence imaging of two-week old leaves was performed by using a blue version of the max chlorophyll imaging system-IMAG-MAX (Walz, Germany), obtaining the maximal quantum efficiency of photosystem II (*Fv*/*Fm*): (a) The leaves of the luffa seedlings gradually wilted under low temperature (5°C) treatments (0h, 2h, 4h, 8h, 12h). (b) The response of *Fv*/*Fm* to low temperature stress, and the *Fv*/*Fm* values decreased gradually with low temperature treatments.
